# Integrated Analysis of Long Noncoding RNA and mRNA Expression Profile in Advanced Laryngeal Squamous Cell Carcinoma

**DOI:** 10.1371/journal.pone.0169232

**Published:** 2016-12-29

**Authors:** Ling Feng, Ru Wang, Meng Lian, Hongzhi Ma, Ning He, Honggang Liu, Haizhou Wang, Jugao Fang

**Affiliations:** 1 Department of Otorhinolaryngology Head and Neck Surgery, Beijing Tongren Hospital, Capital Medical University, Beijing, China; 2 Key Laboratory of Otorhinolaryngology Head and Neck Surgery, Ministry of Education, Beijing, China; 3 Department of Head and Neck Surgery, The People’s Hospital of Guangxi Zhuang Autonomous Region, Nanning, Guangxi, China; 4 Beijing Key Laboratory of Head and Neck Molecular Diagnostic Pathology, Beijing, China; University of South Alabama Mitchell Cancer Institute, UNITED STATES

## Abstract

Long non-coding RNA (lncRNA) plays an important role in tumorigenesis. However, the expression pattern and function of lncRNAs in laryngeal squamous cell carcinoma (LSCC) are still unclear. To investigate the aberrantly expressed lncRNAs and mRNAs in advanced LSCC, we screened lncRNA and mRNA expression profiles in 9 pairs of primary Stage IVA LSCC tissues and adjacent non-neoplastic tissues by lncRNA and mRNA integrated microarrays. Gene Ontology and pathway analysis were performed to find out the significant function and pathway of the differentially expressed mRNAs, gene-gene functional interaction network and ceRNA network were constructed to select core mRNAs, and lncRNA-mRNA expression correlation network was built to identify the interactions between lncRNA and mRNA. qRT-PCR was performed to further validate the expressions of selected lncRNAs and mRNAs in advanced LSCC. We found 1459 differentially expressed lncRNAs and 2381 differentially expressed mRNAs, including 846 up-regulated lncRNAs and 613 down-regulated lncRNAs, 1542 up-regulated mRNAs and 839 down-regulated mRNAs. The mRNAs ITGB1, HIF1A, and DDIT4 were selected as core mRNAs, which are mainly involved in biological processes, such as matrix organization, cell cycle, adhesion, and metabolic pathway. LncRNA-mRNA expression correlation network showed LncRNA NR_027340, MIR31HG were positively correlated with ITGB1, HIF1A respectively. LncRNA SOX2-OT was negatively correlated with DDIT4. qRT-PCR further validated the expression of these lncRNAs and mRNAs. The work provides convincing evidence that the identified lncRNAs and mRNAs are potential biomarkers in advanced LSCC for further future studies.

## Introduction

Head and neck squamous cell carcinoma (HNSCC) represents 4% of all cancer types worldwide, with 500,000 new cases estimated in 2008. Laryngeal squamous cell cancer (LSCC) accounts for about 30% of HNSCC with more than 150,000 new cases are diagnosed annually. The age-standardized world mortality rate of laryngeal cancer is about 2.3 /100,000 [1]. At present, early laryngeal cancers are mainly treated with laser resection, partial laryngectomy, or radiation therapy, while subtotal or total laryngectomy, followed by radiotherapy, are usually used in advanced laryngeal cancers [2]. In general, the early-stage patients retain vocal and swallowing functions after treatment. However, advanced patients often suffer from permanent tracheostomy and loss of natural voice [3]. Thus, to prevent voice disorders and dysphagia in advanced patients and improve the patient’s prognosis and long-term quality of life, it is urgently needed to identify novel biomarkers for early diagnosis and therapy improvement, and investigate the underlying molecular mechanisms in advanced LSCC.

Long noncoding RNAs (lncRNAs) are defined as RNA transcripts larger than 200 nucleotides (nt) without coding potential, which can interact with DNA, RNA, or protein molecules to significantly regulate gene expression and affect cellular processes [4–5]. It is known that lncRNAs play important roles in initiation and progression of cancer and may serve as potential biomarkers for early diagnosis, prognosis, and potential therapy targets in various cancers [6–11]. However, the lncRNA and mRNA expression profiles in advanced LSCC are unknown. In order to investigate the potential aberrantly expressed lncRNA and mRNA in advanced LSCC, we screened expression profiles in 9 pairs of primary Stage IVA LSCC tissues and adjacent non-neoplastic tissues by lncRNA and mRNA integrated microarrays. Using this data, we conducted in-depth integrated bioinformatics analysis to explore lncRNA and mRNA function and interaction, and performed qRT-PCR to further validate the expression of selected lncRNAs and mRNAs.

## Materials and Methods

### Experimental reagents and apparatus

Antibiotic solution (104 U penicillin, 10 mg streptomycin, 25 ug amphotericin B), dimethyl sulfoxide (DMSO) were purchased from Sigma Chemicals (St. Louis, MO, USA). Fetal bovine serum (FBS) and Dulbecco’s Modified Eagle’s Medium (DMEM) were obtained from Gibco (Cambrex, MD, USA). Trizol was purchased from Invitrogen (Carlsbad, CA, USA). MiScript Reverse Transcription Kit was purchased from Qiagen (Hilden, Germany), and SYBR Green Master Mix Kit was purchased from TaKaRa (Dalian, China).

### Patients and tissue samples

A total number of 39 pairs of LSCC tissues and adjacent non-neoplastic tissues were surgically removed between the years of 2012 to 2014 in Beijing Tongren Hospital, including 9 pairs for microarray analysis and 30 pairs for qRT-PCR validation. This study was conducted in 2014 to 2015. According to Union for International Cancer Control (UICC) and American Joint Committee on Cancer (AJCC), the advanced LSCC patients were determined as stage III and IVA, including lymphatic metastatic patients (S1 Table). All patients were diagnosed clinically and histologically by laryngoscope, CT, MRI and pathology biopsy, and never received radiotherapy and/or chemotherapy before surgery. The Epstein–Barr virus (EBV) status was not checked and the pathology was confirmed by at least two pathologists. The inclusion and exclusion criteria for patients’ selection were shown in S2 Table. All the clinical characteristics of the 39 patients with laryngeal cancer were shown in Table 1. One pair of cancer tissue and adjacent non-neoplastic tissue was obtained from one patient, which was used in microarray analysis or qRT-PCR validation. All tissue samples were stored at -80°C refrigerator within 10 minutes after the resection. Authors had access to information that could identify individual participants during or after data collection. All patients were provided written informed consent before their participation. The study was undertaken in accordance with the Institutional Ethics Committee of Beijing Tongren Hospital Affiliated to Capital Medical University and the ethical standards of the World Medical Association Declaration of Helsinki.

**Table 1:** Clinical characteristics of 39 patients with laryngeal cancer.

Characteristic	Advanced laryngeal cancer patients
**Age (mean±SD), years**	60±12
**Sex (no.)**	
Male	37
Female	2
**Tumor location**	
Supraglottis	12
Glottis	27
**Histological type (no.)**	
Squamous cell carcinoma	39
**Differentiation of cancer tissue**	
High differentiation	17
Moderate differentiation	15
Poor differentiation	7
**TNM stage**	
Stage III	24
Stage IV	15
**Smoke**	
Smoker	36
Non-smoker	3

### RNA preparation

Total RNA was extracted using trizol reagent according to manufacturer’s protocol, and then quantified by the NanoDrop ND-2000 (Waltham, MA, USA). The RNA integrity was assessed using Agilent Bioanalyzer 2100 (Santa Clara, CA, USA).

### Microarray processing and analysis

Agilent lncRNA Gene Expression 4 × 180K Microarray (Design ID: 042818, Agilent Technologies, USA) was used to test the lncRNA and mRNA expression profiling. The sample labeling, microarray hybridization and washing were performed based on the manufacturer’s standard protocols. Briefly, total RNA was transcribed to double strand cDNA, then synthesized into cRNA and labeled with Cyanine-3-CTP. The labeled cRNAs were hybridized onto the microarray. After washing, the arrays were scanned by the Agilent Scanner G2505C (Agilent Technologies). Random Variance Model(RVM) t-test was applied to filter the differentially expressed genes between tumor tissues and adjacent non-cancerous tissues according to the p-value threshold. P value < 0.05 was considered as significant difference [12]. The Hierarchical Clustering was conducted to analyze the differentially expressed lncRNAs and mRNAs. Four types of binary, agglomerative, hierarchical clustering were performed to assemble a set of genes into a tree, where genes were joined by very short branches if they were very similar to each other, and by increasingly longer branches as their similarity decreased. The microarray data have been deposited in Gene Expression Omnibus (GEO) database and are accessible through GEO accession number GSE84957.

### GO and KEGG analysis

Gene Ontology (GO: http://www.geneontology.org) analysis and pathway analysis were used to find out the significant function and pathway of the differentially expressed mRNAs in tumor tissues compared to adjacent non-neoplastic tissues. Two-sided Fisher’s exact test and *X*^2^ test were used to classify the GO category, and the False Discovery Rate (FDR) was calculated to correct the P-value [13]. Pathway analysis was conducted according to Kyoto Encyclopedia of Genes and Genomes (KEGG: http://www.genome.ad.jp/kegg/), Biocarta (http://www.genecarta.com/) and Reactome (http://www.reactome.org/). Two-sided Fisher’s exact test and *X*^2^ test were used to select significant pathways, and the threshold of significance was still defined by P-value and FDR [14].

### Gene-gene functional interaction network

Gene-gene functional interaction network was constructed based on the data of differentially expressed genes. KEGG database was used to analyze the functional gene interactions and Cytoscape software was used to build the network. In the network, each gene corresponded to a node, the nodes connected by an edge. The degree of a gene was defined as the number of directly linked genes within a network, which can assess the relative significance of a gene in the network. Thus, in a network, the more adjacent genes a gene connects the higher degree it has and the more important it is. Meanwhile the character of a gene was also described by betweenness centrality, which was an indicator of a gene's centrality in a network. It is equal to the number of shortest paths from all vertices to all others that pass through that gene [15]. Thus, degree and betweenness centrality were used as two indicators to identify the most important genes.

### CeRNA network

CeRNA network was constructed to discover ceRNA mechanism based on the differentially expressed lncRNAs and mRNAs. RNA transcripts could combine with miRNAs by miRNA response element (MRE), so we could find the competition relationship between RNA transcript in the process of combining MRE by predicting MRE and computing free energy. First, miRNA-mRNA, miRNA-lncRNA target relationships were predicted by target prediction database. Pearson correlation coefficient (PCC) between matched lncRNA-mRNA was computed based on their expression data. Then, the PCC between miRNA -mRNA, miRNA-lncRNA was computed. For a given lncRNA-mRNA pair, both mRNA and lncRNA were targeted by a common miRNA and co-expressed negatively with this miRNA. Finally, this miRNA-mRNA-lncRNA was identified as competing triplets [16– 17].

### LncRNA-mRNA expression correlation network

According to the normalized signal intensity of specific expression in mRNAs and lncRNAs, lncRNA-mRNA expression correlation network was built to identify the correlations between lncRNA and mRNA. For each of mRNA-lncRNA, mRNA-mRNA or lncRNA-lncRNA pairs, Pearson correlation was calculated to choose the significant correlation pairs and the correlation value cutoff was 0.92. The degree was calculated to measure a gene or lncRNA centrality within a network. While considering different networks, core genes were determined by the degree differences between two group samples.

### qRT- PCR

qRT-PCR was performed to validate the expression of significantly altered lncRNA and its correlated mRNA in an independent cohort of 30 pairs of LSCC tumor tissues and adjacent non-neoplastic tissues. Total RNA was extracted using Trizol reagent (Invitrogen) following purification with an RNeasy kit (Qiagen, Valencia, CA, USA). Total RNA then was reverse-transcribed to cDNA using M-MLV reverse transcription (Promega) according to manufacturer’s instructions. Quantitative PCR analysis and data collection were performed on the ABI 7500 Real-Time PCR System (Applied Biosystems, Carlsbad, CA, USA) using the primer pairs listed (Table 2). 18s served as an endogenous control for normalization. For relative quantification, 2-^ΔΔCt^ was calculated and used as an indication of gene relative expression.

**Table 2:** Primers used for qRT-PCR.

lncRNA or mRNA	Forward primer (5’-3’)	Reverse primer (5’-3’)
NR_003949	AAGCCAACTGTGTGGCAGAA	ATCCCATGCTAAGGCCCTCT
NR_027340	TGGTGACCTAACATGAGGCT	GTTGGGTGACACCTCACCAT
SOX2-OT	AACACCCTGATCTGGCATGG	ATATGGCTGTTGCCTGGCTT
MIR31HG-001	GAGGAGCGCTTTGTGTGAGA	AGAAGGCCCAGGCTATGTCT
HIF1A	TCAAAGTCGGACAGCCTCAC	ATCCATTGATTGCCCCAGCA
PIK3R1	GTGAAGCTCGTGTGTGGAGT	GAAGACAGGGCTCCACTTCC
ITGB1	TTCCGAACGTGAGGGTCGC	TGTTGAATTTGTGCACCACCC
DDIT4	TTAGCAGTTCTCGCTGACCG	CCAAAGGCTAGGCATGGTGA

### Statistical analysis

SPSS 20.0 software (SPSS Inc., Chicago, IL, USA) was used to do statistical analysis. All data were shown as mean ± SD of three independent experiments with each experiment in triplicate. The Student t test was used to evaluate the expression differences of lncRNAs and mRNAs between LSCC cancer tissues and adjacent non-neoplastic tissues. P<0.05 was considered as statistically significant.

## Results

### LncRNA and mRNA expression profile of advanced LSCC

**Fig 1:**
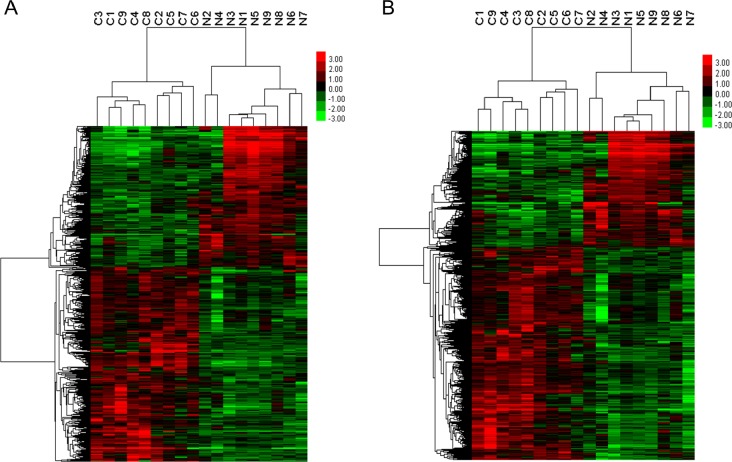
Hierarchical clustering of aberrant expressed lncRNAs and mRNAs detected in advanced LSCC. Red color indicates over expression and green color indicates low expression. Every column represents a tissue sample and every row represents an lncRNA/mRNA probe. C represents cancer tissues and N represents adjacent normal tissues. (A) lncRNA hierarchical clustering. (B) mRNA hierarchical clustering.

To understand how the lncRNA and mRNA were differentially expressed in tumor and adjacent non-neoplastic tissues, we employed hierarchical clustering analysis. We found 1459 differentially expressed lncRNAs (Fig 1A, S3 Table) and 2381 differentially expressed mRNAs (Fig 1B, S4 Table), including 846 up-regulated lncRNAs and 613 down-regulated lncRNAs, 1542 up-regulated mRNAs and 839 down-regulated mRNAs (fold change≥2, p<0.05). The differentially expressed mRNAs and lncRNAs perfectly distinguished tumor tissues (C1-C9) from adjacent non-neoplastic tissues (N1-N9).

### mRNA functional annotation and pathway analysis

**Fig 2:**
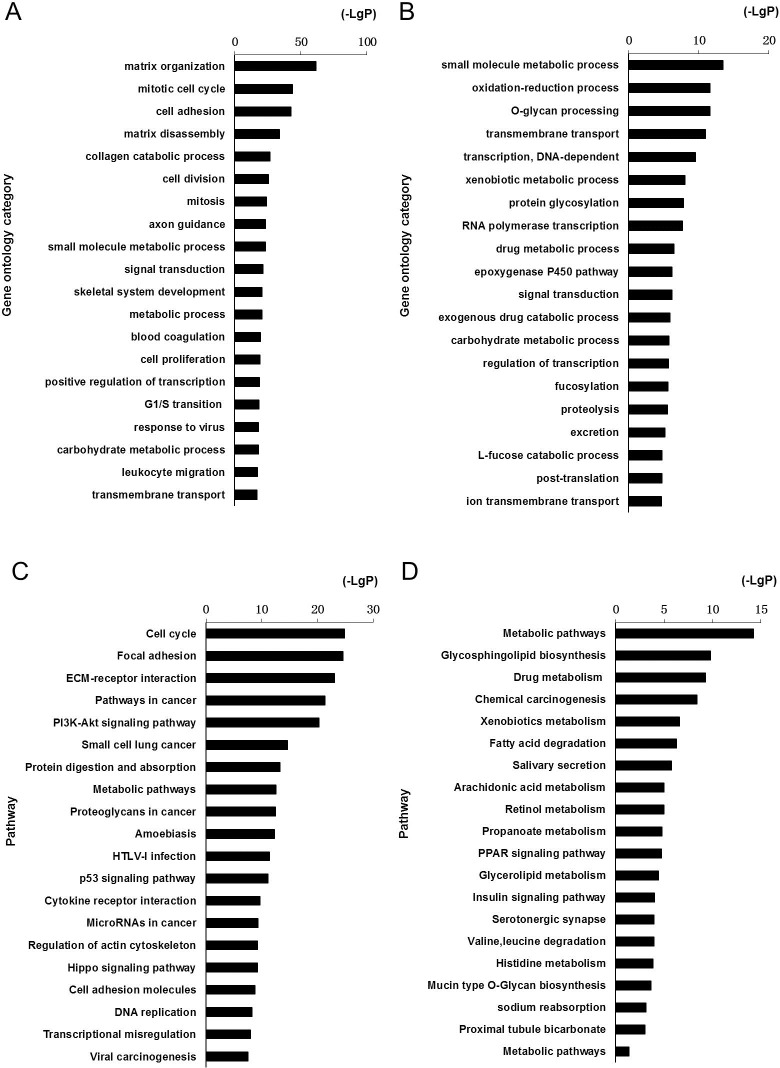
GO and pathway analysis of differentially expressed mRNAs in advanced LSCC. A total of 2381 differentially expressed mRNAs were chosen in GO and pathway analysis. The bar graphs represented the enrichment of these mRNAs. The value of (-LgP) was p value taking the negative logarithm with base 10. The threshold of significance was P value<0.05 and FDR was calculated to correct the P value. (A) Top 20 enriched GO terms among up-regulated mRNAs. (B) Top 20 enriched GO terms among down-regulated mRNAs. (C) Top 20 enriched pathways among up-regulated mRNAs. (D) Top 20 enriched pathways among down-regulated mRNAs.

In order to understand the biological processes, we performed Gene Ontology (GO) and pathway analysis. GO analysis revealed aberrantly expressed mRNAs involved in up-regulated GO function, including matrix organization, mitotic cell cycle, cell adhesion, cell disassembly, and collagen catabolic process (Fig 2A). Down-regulated GO functions included small molecule metabolic process, oxidation-reduction, O-glycan processing, transmembrane transport, and DNA dependent transcription (Fig 2B). Pathway analysis revealed aberrantly expressed mRNAs involved in up-regulated pathways, including cell cycle, focal adhesion, ECM-receptor interaction, pathways in cancer, and PI3K-Akt signaling (Fig 2C), whereas down-regulated pathways included metabolic pathways, glycosphingolipid biosynthesis, drug metabolism, chemical carcinogenesis, and xenobiotics metabolism (Fig 2D).

### Gene-gene functional interaction network

**Fig 3:**
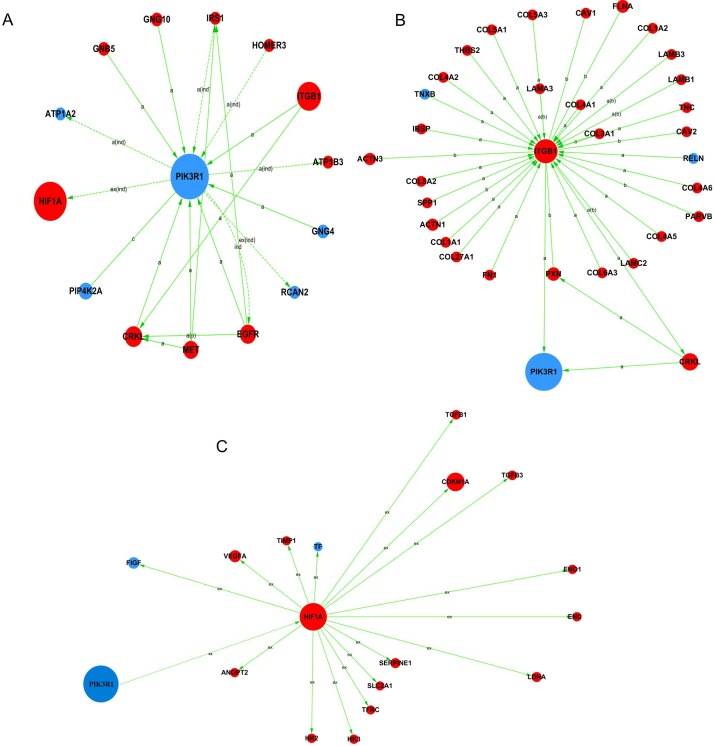
Gene-gene functional interaction network analysis of differentially expressed mRNAs in advanced LSCC. In the network, a node represents a gene, red color indicates up-regulation and blue color indicates down-regulation, the size of the node’s area represents the value of betweenness centrality. The nodes connect by an edge. The indicators a, b, c, p, u, m, inh, ex, dep, ind are abbreviation of activation, binding, compound, phosphorylation, ubiquitination, missing interaction, inhibition, expression, dephosphorylation, indirect effect respectively. (A) PIK3R1 signal network. (B) ITGB1 signal network. (C) HIF1A signal network.

Gene-gene functional interaction network was performed to identify the most important mRNAs. According to the network (S1 Fig), PIK3R1 was within the highest range of centrality, reflecting its main part in the network. ITGB1 had the highest degree value, indicating it connected the most genes and how important it was. HIF1A had the second highest value of betweenness centrality and the second highest degree value (Table 3, Fig 3). As a result of this analysis, PIK3R1, ITGB1, and HIF1A were determined to have important functions in the gene-gene functional interaction network.

**Table 3:** Core mRNAs selected by gene-gene functional interaction network.

Genesymbol	Style	Betweenness Centrality	Degree
PIK3R1	down	0.0201666	15
HIF1A	up	0.0161601	17
ITGB1	up	0.0098137	32
PLA2G2F	up	0.0094055	12
GATM	Down	0.0086179	3
CDKN1A	up	0.0082969	7
SHMT1	down	0.0081975	5
CYP2E1	down	0.0076066	14
GART	up	0.0062899	5
ALDH7A1	down	0.0059598	8
ALDH2	down	0.0059598	8
PPAT	up	0.0058419	4
MYC	up	0.0057823	12

Betweenness centrality ≥0.005. Betweenness centrality is an indicator of a gene's centrality in a network. It is equal to the number of shortest paths from all vertices to all others that pass through that gene. The degree of a gene was defined as the number of directly linked genes within a network.

### CeRNA network analysis

**Fig 4:**
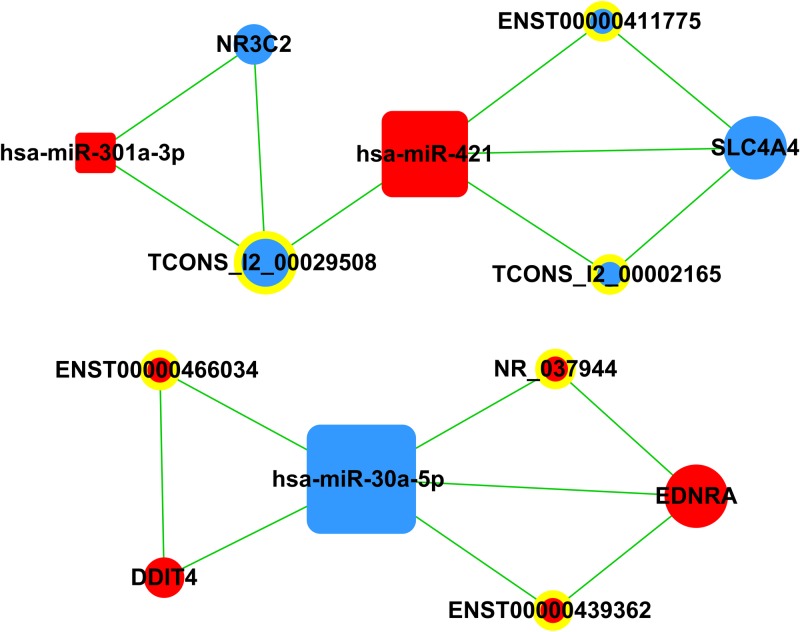
CeRNA network analysis of differentially expressed mRNAs, lncRNAs, and miRNAs in advanced LSCC. In the network, nodes represent mRNAs, nodes with yellow rings represent lncRNAs, squares represent miRNAs, and the size of the node’s area represents the value of betweenness centrality. Red color indicates up-regulation, blue color indicates down-regulation, and edges indicate target interactions.

A ceRNA network was constructed to determine whether the differentially expressed mRNAs and lncRNAs were involved in a ceRNA mechanism. The network analysis showed four mRNAs (DDIT4, EDNRA, NR3C2, SLC4A40), six lncRNAs (ENST00000466034, ENST00000439362, NR_037944, TCONS_12_00029508, TCONS_12_00002165, ENST00000411775) and three miRNAs (has-miR-30a-5p, has-miR-301a-3p, has-miR-421) involved in the ceRNA network (Fig 4). DDIT4 has been well-studied in cancer compared to other mRNAs and has-miR-30a-5p was with the highest degree in the ceRNA network. Thus, DDIT4, lncRNA SOX2-OT (also named as ENST00000466034), and has-miR-30a-5p were highlighted here.

### LncRNA-mRNA expression correlation network

**Fig 5:**
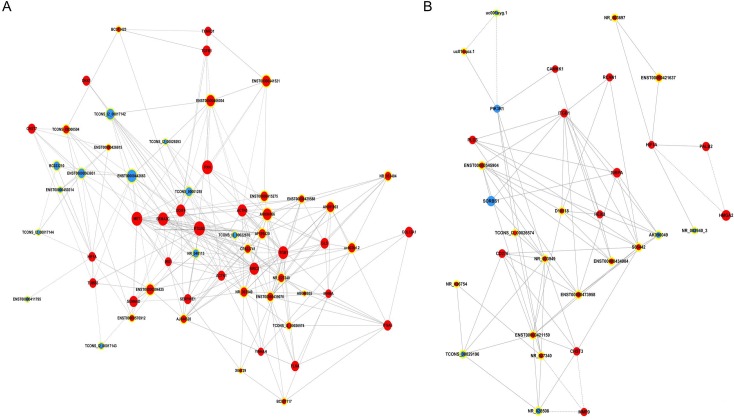
LncRNA-mRNA expression correlation network analysis of core mRNAs and their correlated lncRNAs in advanced LSCC. In the network, nodes represent mRNAs, nodes with yellow rings represent lncRNAs, and the size of the node’s area represents the value of betweenness centrality. Red color indicates up-regulation and blue indicates down-regulation relative to adjacent non-neoplastic tissues. The lines between nodes indicate a correlative relationship within the group, solid line represents positive correlation, and the dotted line represents negative correlation. (A) LncRNA-mRNA expression correlation network of core mRNAs in cancer tissues. (B) LncRNA-mRNA expression correlation network of core mRNAs in adjacent non-neoplastic tissues.

lncRNA-mRNA expression correlation network was built to identify the correlations between mRNAs and lncRNAs in cancer tissues and adjacent non-neoplastic tissues (S2 and S3 Figs). According to the core mRNAs selected above, we found that lncRNA NR_003949 was positively correlated with PIK3R1, lncRNA NR_027340 was positively correlated with ITGB1, lncRNA MIR31HG (also named as ENST00000304425) was positively correlated with HIF1A, and lncRNA SOX2-OT (also named as ENST00000466034) was negatively correlated with DDIT4 (Table 4, Fig 5).

**Table 4:** The correlation between lncRNAs and the four core mRNAs.

mRNA	LncRNA	Pearson correlation
PIK3R1	NR_003949	0.9535264
ITGB1	NR_027340	0.9681052
HIF1A	MIR31HG	0.9315686
DDIT4	SOX2-OT	-0.9386784

### QRT-PCR validation

**Fig 6:**
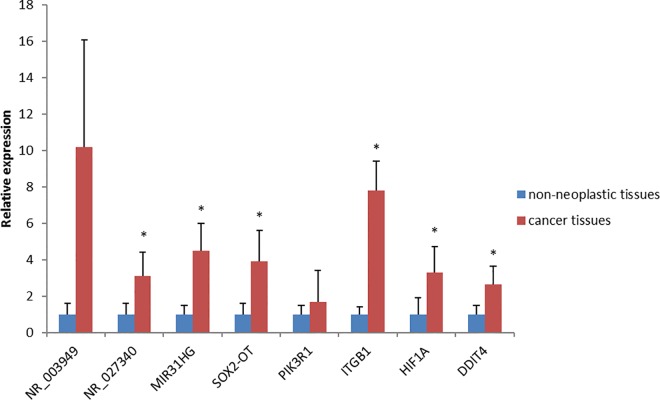
qRT-PCR analysis of relative expression levels of selected lncRNAs and mRNAs. The bars represent standard deviations, and the asterisks above the bars denote statistically significant differences from the control group, P<0.05.

To further validate the results of the microarray analysis, the expression of lncRNAs and mRNAs selected above were analyzed by qRT-PCR in an independent cohort of 30 pairs of LSCC cancer tissues and adjacent non-neoplastic tissues. lncRNAs NR_027340, MIR31HG, SOX2-OT and their correlated mRNAs ITGB1, HIF1A, DDIT4 were overexpressed in cancer tissues compared to adjacent non-neoplastic tissues (P<0.05), but there was no significant difference in the expressions of lncRNA NR_003949 and its correlated mRNA PIK3R1 (P > 0.05) (Fig 6). The results of qRT-PCR were consistent with those in microarray with the same trend, except for PIK3R1 (Table 5).

**Table 5:** The expression levels of selected lncRNAs and their correlated mRNAs in microarrays.

LncRNA/mRNA	Fold change(C/N)	P value	expression
NR_003949	2.99	0.000124	up-regulation
NR_027340	2.86	0.000122	up-regulation
MIR31HG	3.47	0.008458	up-regulation
SOX2-OT	2.70	0.032317	up-regulation
PIK3R1	0.43	0.001708	down-regulation
ITGB1	2.45	0.002554	up-regulation
HIF1A	2.61	0.001699	up-regulation
DDIT4	2.28	0.008304	up-regulation

C/N: cancer tissues/adjacent non-neoplastic tissues.

## Discussion

Genetic investigations have shown that at least 90% of the human genome is actively transcribed into non-coding RNA (ncRNA), indicating that ncRNAs may play a significant regulatory role in driving cancer [18–19]. The recent implementation of tiling arrays and RNA deep sequencing (RNA-seq) has made it possible to investigate the transcriptomes to an unprecedented degree, and tens of thousands lncRNAs are found to be uniquely expressed in specific cancer types [20–21]. In head and neck cancer, thousands of noncoding intronic RNAs were found to be involved in transcriptional regulation, may play an important role in HNSCC [22]. A lot of aberrant expressed lncRNAs contribute to the hallmarks of cancer by modulating gene expression in major biological processes of cancer [23–24]. LncRNA GAS5 suppresses stomach carcinogenesis by regulating p21 expression and then enhancing G1 cell cycle arrest [25]. LncRNA APOC1P1-3 inhibits apoptosis by decreasing α-tubulin acetylation in breast cancer [26]. LncRNA H19 promoted LSCC progression via miR-148a-3p and DNMT1 [27]. Our results demonstrate that 1459 differentially expressed lncRNAs and 2381 differentially expressed mRNAs were identified by microarray in advanced LSCC and may act as novel biomarkers for this disease. Among them, lncRNAs MIR31HG, NR_027340, SOX2-OT and their correlated mRNAs HIF1A, ITGB1, DDIT4 were selected with core function by bioinformatics analysis and further validated their expression by qRT-PCR in advanced LSCC.

In this study, mRNAs functional annotation and pathway analysis revealed that the aberrantly expressed genes were involved in biological processes, such as matrix organization, cell cycle, adhesion, and metabolic pathways. Previous studies have already indicated that these are all classical biological processes in cancer. For example, the extracellular matrix, a major component of the local microenvironment, is commonly deregulated and becomes disorganized in cancer [28]. Cell cycle, the process by which cells progress and divide, malfunction will lead to uncontrolled cell proliferation as a result of genetic mutations in cancer cells [29]. Adhesion molecules are well-studied to play a significant role in cancer progression and metastasis. Tumor cell adhesion in the vasculature of specific organs is essential step in the metastatic cascade, and determines the metastatic spread [30]. Reprogramming of metabolic pathway is a key factor in carcinogenesis, which can improve the ability to acquire necessary nutrients from a frequently nutrient-poor environment and utilize these nutrients [31]. In a word, these are all classical cancer processes validated by intensive researches. Our results demonstrated that the most aberrantly expressed mRNAs were enriched in these classical biological processes, indicating that the aberrantly expressed mRNAs selected by our microarrays are not only differentially expressed in advanced LSCC, but also have important functions in the progression and metastasis process of advanced LSCC.

LncRNAs exert function through modulating mRNA processing and post-transcriptional regulation [32]. In our study, lncRNAs NR_027340, MIR31HG, NR_003949 and their correlated mRNAs ITGB1, HIF1A, PIK3R1 are identified by gene-gene functional interaction network and lncRNA-mRNA expression correlation network. Integrin β1 (ITGB1) is a type of cell adhesion molecule that mediates mutual adhesion between cells and Extracellular Matrix (ECM). ITGB1 is often abnormally expressed in tumors and is implicated in aberrant proliferation, angiogenesis, invasion and metastasis [33–34]. Hypoxia-inducible factor 1a (HIF1a) is an essential transcription factor for cellular adaptation to hypoxia in cancer [35–36]. Intratumoral hypoxia is a common feature of solid malignancies, including LSCC. Indeed, HIF1a has been well studied in cancer progression and is implicated in the malignancy phenotype of LSCC [37–38]. Phosphoinositide-3-kinase regulatory subunit 1(PIK3R1) binds, stabilizes and inhibits the PI3K p110 catalytic subunit [39]. Previous studies show that PIK3R1 suppresses tumor cell invasion and migration by reducing PI3K/AKT signaling [40]. In this study, ITGB1, HIF1A, and PIK3R1 were selected as core mRNAs with important function in advanced LSCC. We found that lncRNA NR_027340, MIR31HG, NR_003949 were positively correlated with ITGB1, HIF1A, PIK3R1 respectively, suggesting lncRNA NR_027340, MIR31HG, NR_003949 may target ITGB1, HIF1A, PIK3R1 to modulate their expression in advanced LSCC. qRT-PCR further validated their expressions in an independent cohort of 30 pairs of LSCC tumor tissue and adjacent normal tissue samples. The expressions of lncRNA NR_027340, MIR31HG and their correlated mRNAs ITGB1, HIF1A were statistically different in the two groups, but there is no significant difference in the expressions of lncRNA NR_003949 and its correlated mRNA PIK3R1.

Some long noncoding RNAs (lncRNAs) play important roles in the regulation of gene expression by acting as competing endogenous RNAs (ceRNAs) [41]. CeRNA, a hypothesis about how mRNAs, long noncoding RNAs “talk” to each other using microRNA response elements (MREs) as letters of a new language. This ceRNA activity forms a large-scale regulatory network across the transcriptome, greatly expands the functional genetic information in the human genome and plays important roles in cancer [42]. DDIT4 has been well-studied in cancer compared to other mRNAs in the ceRNA network. DDIT4, lncRNA SOX2-OT, and has-miR-30a-5p were selected, which were supposed to involve in ceRNA mechanism. These findings were validated with qRT-PCR and were consistent with the expression trends in the microarray. However further experiments are needed to validate this hypothesis.

To date, very few lncRNAs have been characterized in detail in cancers. The lncRNA MIR31HG is located in 9p21.3 and 2166 bp in length, contributes to cell proliferation and invasion in breast cancer and gastric cancer [43–45]. However, ours is the first study to identify MIR31HG in LSCC. Sox2 overlapping transcript (SOX2-OT) is an lncRNA located on human chromosome 3q26.33. SOX2-OT plays an important role in regulating cell proliferation, and may represent a novel prognostic indicator for lung cancer [46]. Ectopic expression of SOX2OT reduces the proliferation rate and increases anchorage independent growth in breast cancer [47]. SOX2OT has also been identified in esophageal squamous cell carcinoma and concordant regulated with SOX2 [48]. Lastly, there are no reported functional studies on lncRNA NR_027340. Further studies are urgently needed to elucidate the molecular mechanisms of these lncRNAs in LSCC.

In conclusion, lncRNAs NR_027340, MIR31HG, SOX2-OT and core mRNAs ITGB1, HIF1A, DDIT4 are identified as potential biomarkers in advanced LSCC by integrated microarray analysis, and further validated by qRT-PCR. The data presented here suggest for the first time that these up-regulated lncRNAs and mRNAs may represent a new development in diagnosis and prognosis of advanced LSCC, and these targets may be used in potential lncRNA-mediated therapy as well.

## Supporting Information

S1 Table Demographics data of 39 patients with laryngeal cancer. (PDF)

S2 Table The inclusion and exclusion criteria for patients’ selection. (PDF)

S3 Table Differentially expressed lncRNA list in 9 pairs of primary Stage IVA LSCC tissues and adjacent non-neoplastic tissues. (PDF)

S4 Table Differentially expressed mRNA list in 9 pairs of primary Stage IVA LSCC tissues and adjacent non-neoplastic tissues. (PDF)

S5 Table The ceRNA relationship between miRNA, lncRNA, and mRNA in advanced LSCC. (PDF)

S1 Fig Gene-gene functional interaction network of all differentially expressed mRNAs in advanced LSCC. In the network, a node represents a gene, red color indicates up-regulation and blue color indicates down-regulation, the size of the node’s area represents the value of betweenness centrality. The nodes connect by an edge. The indicators a, b, c, p, u, m, inh, ex, dep, ind are abbreviation of activation, binding, compound, phosphorylation, ubiquitination, missing interaction, inhibition, expression, dephosphorylation, indirect effect respectively.
(PDF)

S2 Fig LncRNA-mRNA expression correlation network in cancer tissues. (PNG)

S3 Fig LncRNA-mRNA expression correlation network in adjacent non-neoplastic tissues. (PNG)
