# Supplementary material for: Integrated Analysis of Long Noncoding RNA and mRNA Expression Profile in Advanced Laryngeal Squamous Cell Carcinoma
Source: PLoS One. 2016 Dec 29;11(12):e0169232. doi: 10.1371/journal.pone.0169232 (PMC5199101; doi:10.1371/journal.pone.0169232)
Supplement: S1 Table — (PDF) [file pone.0169232.s001.pdf]

S1 Table: Demographics data of 39 patients with laryngeal cancer

sample	age	sex	tumor location	TNM stage	Differentiation of cancer tissues	Smoking status
1	69	male	glottis	T4aN1M0	moderately differentiated squamous cell carcinoma	smoker
2	52	male	glottis	T4aN1M0	moderately differentiated squamous cell carcinoma	non-smoker
3	72	male	glottis	T4aN0M0	moderately differentiated squamous cell carcinoma	smoker
4	48	male	glottis	T4aN0M0	highly differentiated squamous cell carcinoma	smoker
5	70	male	glottis	T4aN0M0	moderately differentiated squamous cell carcinoma	smoker
6	80	male	glottis	T4aN2cM0	moderately differentiated squamous cell carcinoma	smoker
7	45	male	glottis	T4aN0M0	highly differentiated squamous cell carcinoma	smoker
8	81	male	glottis	T4aN0M0	highly differentiated squamous cell carcinoma	smoker
9	50	male	glottis	T4aN0M0	moderately differentiated squamous cell carcinoma	smoker
10	72	male	supraglottis	T3N0M0	moderately differentiated squamous cell carcinoma	smoker
11	59	male	supraglottis	T3N1M0	moderately differentiated squamous cell carcinoma	smoker
12	60	male	glottis	T3N0M0	highly differentiated squamous cell carcinoma	smoker
13	60	male	supraglottis	T3N1M0	poorly differentiated squamous cell carcinoma	smoker

14	68	male	glottis	T3N1M0	moderately differentiated squamous cell carcinoma	smoker
15	37	female	glottis	T3N0M0	highly differentiated squamous cell carcinoma	non-smoker
16	69	male	supraglottis	T3N1M0	moderately differentiated squamous cell carcinoma	smoker
17	43	male	supraglottis	T3N1M0	poorly differentiated squamous cell carcinoma	smoker
18	65	male	supraglottis	T3N1M0	moderately differentiated squamous cell carcinoma	smoker
19	60	male	glottis	T4aN0M0	moderately differentiated squamous cell carcinoma	smoker
20	49	male	glottis	T4aN0M0	highly differentiated squamous cell carcinoma	smoker
21	70	male	glottis	T4aN0M0	highly differentiated squamous cell carcinoma	non-smoker
22	50	male	glottis	T4aN0M0	poorly differentiated squamous cell carcinoma	smoker
23	73	male	glottis	T4aN1M0	poorly differentiated squamous cell carcinoma	smoker
24	49	male	glottis	T3N0M0	highly differentiated squamous cell carcinoma	smoker
25	63	male	supraglottis	T3N1M0	highly differentiated squamous cell carcinoma	smoker
26	49	male	supraglottis	T3N1M0	moderately differentiated squamous cell carcinoma	smoker
27	70	male	glottis	T4aN0M0	poorly differentiated squamous cell carcinoma	smoker

28	75	male	supraglottis	T3N1M0	moderately differentiated squamous cell carcinoma	smoker
29	51	male	supraglottis	T3N1M0	moderately differentiated squamous cell carcinoma	smoker
30	52	male	supraglottis	T3N1M0	highly differentiated squamous cell carcinoma	smoker
31	51	male	glottis	T3N0M0	highly differentiated squamous cell carcinoma	smoker
32	47	male	glottis	T3N0M0	highly differentiated squamous cell carcinoma	smoker
33	46	male	glottis	T3N0M0	highly differentiated squamous cell carcinoma	smoker
34	47	male	glottis	T3N0M0	highly differentiated squamous cell carcinoma	smoker
35	53	male	glottis	T3N1M0	poorly differentiated squamous cell carcinoma	smoker
36	69	female	supraglottis	T3N1M0	poorly differentiated squamous cell carcinoma	smoker
37	59	male	glottis	T3N1M0	highly differentiated squamous cell carcinoma	smoker
38	71	male	glottis	T3N1M0	highly differentiated squamous cell carcinoma	smoker
39	72	male	glottis	T3N1M0	highly differentiated squamous cell carcinoma	smoker

The upper 9 patients for microarray analysis and the other 30 patients for qRT-PCR validation
