# Supplementary material for: Integrated Analysis of Long Noncoding RNA and mRNA Expression Profile in Advanced Laryngeal Squamous Cell Carcinoma
Source: PLoS One. 2016 Dec 29;11(12):e0169232. doi: 10.1371/journal.pone.0169232 (PMC5199101; doi:10.1371/journal.pone.0169232)
Supplement: S2 Table — (PDF) [file pone.0169232.s002.pdf]

S2 Table: The inclusion and exclusion criteria for patients' selection

**Inclusion criteria**	1. Subject is a male or female between the age of 21 and 85;<br>2. Subject is diagnosed as stage III or IV laryngeal squamous cell carcinoma clinically and histologically by laryngoscope, CT, MRI and pathology biopsy;<br>3. Subject is primary diagnosed as laryngeal squamous cell carcinoma;<br>4. Subject is conducted surgery in Beijing Tongren Hospital;<br>5. Subject provides written informed consent;<br>6. Subject provides written authorization for use and disclosure of protected health information;<br>7. Subject agrees not to participate in other clinical trial.
**Exclusion criteria**	1. Subject has received radiotherapy and/or chemotherapy before surgery;<br>2. Subject is diagnosed as recurrent laryngeal cancer and/or has remote metastasis;<br>3. Subject has genetic diseases (e.g., Neurofibromatosis, haemochromatosis, etc.);<br>4. Subject has multiple primary cancers (e.g., lung cancer following initial laryngeal cancer);<br>5. Subject has undergone any major surgical procedure or major trauma within 14 days prior to surgery;<br>6. Subject serum is positive for HIV-1 or HIV-2 antibody;<br>7. Subject serum is positive for Hepatitis B surface antigen;<br>8. Subject serum is positive for Hepatitis C antibody;<br>9. Subject has any underlying or current medical condition, which, in the opinion of the Investigator, would interfere with the evaluation of the subject.
