# Supplementary material for: Integrated Analysis of Long Noncoding RNA and mRNA Expression Profile in Advanced Laryngeal Squamous Cell Carcinoma
Source: PLoS One. 2016 Dec 29;11(12):e0169232. doi: 10.1371/journal.pone.0169232 (PMC5199101; doi:10.1371/journal.pone.0169232)
Supplement: S3 Table — (PDF) [file pone.0169232.s003.pdf]

ProbeSet	p-value	FDR	Fold change(C/N)	style
oebiotech_22211	0.008449	0.148	0.019	down
oebiotech_21140	0.000922	0.0632	0.02	down
oebiotech_15679	0.008338	0.146	0.022	down
oebiotech_02630	0.005005	0.116	0.031	down
oebiotech_05868	0.016778	0.199	0.038	down
oebiotech_27178	0.013143	0.178	0.042	down
A_21_P0012184	0.000623	0.0566	0.048	down
A_21_P0012777	0.013159	0.179	0.052	down
oebiotech_26793	0.00066	0.057	0.055	down
oebiotech_11045	0.000414	0.047	0.058	down
A_19_P00315529	0.001169	0.068	0.059	down
A_21_P0011814	0.001099	0.0668	0.069	down
oebiotech_19194	0.001673	0.0767	0.07	down
oebiotech_09407	0.001662	0.0766	0.079	down
A_21_P0005556	0.0002	0.0374	0.081	down
oebiotech_19383	0.000387	0.046	0.082	down
A_32_P150086	0.000768	0.0588	0.084	down
oebiotech_26529	0.001265	0.0698	0.085	down
oebiotech_00619	0.002429	0.0877	0.085	down
oebiotech_07639	0.003722	0.103	0.085	down
oebiotech_22954	0.000313	0.0419	0.087	down
oebiotech_04462	0.007587	0.14	0.087	down
A_32_P405902	0.001093	0.0668	0.091	down
oebiotech_03275	0.002226	0.0852	0.091	down
oebiotech_16761	0.000886	0.0625	0.094	down
oebiotech_18066	0.001367	0.0722	0.099	down
oebiotech_25432	0.000729	0.0585	0.1	down
oebiotech_04007	0.002495	0.0886	0.1	down
A_21_P0011907	0.002906	0.0932	0.1	down
A_21_P0014912	0.016806	0.199	0.1	down
oebiotech_12975	2.33E-05	0.0195	0.11	down
oebiotech_26791	0.000338	0.0438	0.11	down
oebiotech_26002	0.000667	0.0572	0.11	down
oebiotech_25706	0.000682	0.0579	0.11	down
A_21_P0012219	0.000755	0.0585	0.11	down
oebiotech_22453	0.001888	0.0799	0.11	down
oebiotech_23953	0.001899	0.0802	0.11	down
oebiotech_03982	0.002592	0.0893	0.11	down
A_21_P0003627	0.004673	0.114	0.11	down
oebiotech_00174	0.028521	0.249	0.11	down
oebiotech_10867	0.037009	0.28	0.11	down
A_21_P0012182	0.000404	0.0467	0.12	down
oebiotech_26404	0.000851	0.0613	0.12	down
oebiotech_26405	0.001209	0.0688	0.12	down
A_21_P0012183	0.001291	0.0703	0.12	down
oebiotech_25707	0.001413	0.073	0.12	down
oebiotech_26808	0.004234	0.11	0.12	down
oebiotech_21545	0.010483	0.161	0.12	down
A_21_P0014683	0.016877	0.199	0.12	down

oebiotech_07349	0.021879	0.223	0.12	down
A_21_P0008321	9.26E-05	0.0302	0.13	down
oebiotech_26402	0.00017	0.0364	0.13	down
A_21_P0012220	0.000554	0.0535	0.13	down
oebiotech_25508	0.000596	0.0554	0.13	down
A_33_P3340613	0.000648	0.0568	0.13	down
oebiotech_25645	0.000889	0.0625	0.13	down
A_21_P0013585	0.001096	0.0668	0.13	down
oebiotech_26794	0.001159	0.0676	0.13	down
oebiotech_25652	0.001667	0.0766	0.13	down
A_21_P0011169	0.004047	0.108	0.13	down
A_21_P0006276	0.004278	0.11	0.13	down
oebiotech_10918	0.000167	0.0364	0.14	down
A_21_P0013181	0.000187	0.0372	0.14	down
oebiotech_27111	0.000293	0.0413	0.14	down
oebiotech_20863	0.000334	0.0436	0.14	down
oebiotech_27779	0.000401	0.0466	0.14	down
A_21_P0010769	0.000809	0.0599	0.14	down
A_21_P0012181	0.000975	0.0645	0.14	down
oebiotech_25648	0.001126	0.0672	0.14	down
A_33_P3242453	0.001922	0.0806	0.14	down
A_21_P0011168	0.002759	0.0914	0.14	down
A_32_P68942	0.00383	0.105	0.14	down
oebiotech_23693	0.00983	0.157	0.14	down
A_21_P0014383	0.01255	0.174	0.14	down
A_33_P3282359	0.001129	0.0673	0.15	down
oebiotech_25705	0.00118	0.0684	0.15	down
oebiotech_25650	0.001233	0.0692	0.15	down
oebiotech_24113	0.003353	0.0993	0.15	down
A_21_P0003626	0.00393	0.106	0.15	down
oebiotech_26755	0.015109	0.19	0.15	down
oebiotech_12587	0.027457	0.246	0.15	down
oebiotech_05599	1.69E-05	0.0174	0.16	down
oebiotech_27592	0.000471	0.0502	0.16	down
oebiotech_02724	0.001012	0.0655	0.16	down
oebiotech_07965	0.002043	0.0829	0.16	down
A_21_P0011699	0.00209	0.0836	0.16	down
A_21_P0012217	0.002611	0.0894	0.16	down
oebiotech_20395	0.002854	0.0924	0.16	down
oebiotech_21798	0.006896	0.134	0.16	down
oebiotech_22507	0.023232	0.229	0.16	down
oebiotech_27398	0.000168	0.0364	0.17	down
A_21_P0013372	0.000278	0.0406	0.17	down
oebiotech_01607	0.000293	0.0413	0.17	down
A_21_P0010650	0.000491	0.0512	0.17	down
oebiotech_25359	0.001352	0.0718	0.17	down
oebiotech_27785	0.001473	0.0744	0.17	down
oebiotech_11463	0.001725	0.0777	0.17	down
A_21_P0001936	0.001854	0.0792	0.17	down
oebiotech_16232	0.002147	0.084	0.17	down

oebiotech_26807	0.002987	0.0941	0.17	down
oebiotech_23692	0.004583	0.113	0.17	down
oebiotech_26753	0.014698	0.187	0.17	down
A_23_P73848	0.0001	0.0307	0.18	down
oebiotech_25708	0.000253	0.0403	0.18	down
oebiotech_03947	0.000298	0.0414	0.18	down
oebiotech_27784	0.000907	0.063	0.18	down
A_33_P3312384	0.002704	0.0907	0.18	down
oebiotech_22855	0.002709	0.0908	0.18	down
oebiotech_05867	2.81E-05	0.0207	0.19	down
A_21_P0010553	0.000537	0.0528	0.19	down
A_21_P0010554	0.00111	0.0668	0.19	down
oebiotech_27783	0.001234	0.0692	0.19	down
A_33_P3283599	0.001339	0.0714	0.19	down
oebiotech_11414	0.003382	0.0996	0.19	down
oebiotech_26790	0.004213	0.11	0.19	down
A_21_P0009342	0.004553	0.113	0.19	down
oebiotech_22839	0.009247	0.153	0.19	down
oebiotech_14274	0.034968	0.273	0.19	down
oebiotech_21417	0.04063	0.291	0.19	down
A_21_P0001831	0.001641	0.0766	0.2	down
oebiotech_19385	0.002265	0.0858	0.2	down
A_21_P0002855	0.00227	0.0858	0.2	down
oebiotech_25578	0.004652	0.113	0.2	down
oebiotech_25094	0.005368	0.12	0.2	down
A_21_P0008432	0.01554	0.192	0.2	down
oebiotech_10981	0.021306	0.22	0.2	down
oebiotech_18669	0.00015	0.0355	0.21	down
oebiotech_25651	0.000494	0.0512	0.21	down
oebiotech_11568	0.000613	0.0562	0.21	down
oebiotech_25712	0.000816	0.0601	0.21	down
oebiotech_26035	0.003072	0.0954	0.21	down
oebiotech_25647	0.004338	0.111	0.21	down
oebiotech_21799	0.005642	0.123	0.21	down
oebiotech_16582	0.008141	0.145	0.21	down
oebiotech_12789	0.010059	0.159	0.21	down
oebiotech_27866	0.00035	0.0444	0.22	down
A_21_P0006588	0.001612	0.0765	0.22	down
A_21_P0010555	0.001784	0.0784	0.22	down
oebiotech_15084	0.003158	0.0967	0.22	down
oebiotech_15247	0.004237	0.11	0.22	down
oebiotech_26792	0.00475	0.114	0.22	down
oebiotech_18976	0.007503	0.14	0.22	down
oebiotech_02504	0.017463	0.202	0.22	down
oebiotech_11507	0.033839	0.269	0.22	down
A_21_P0010720	0.000648	0.0568	0.23	down
oebiotech_02977	0.000727	0.0585	0.23	down
oebiotech_26014	0.001223	0.0692	0.23	down
oebiotech_07399	0.003616	0.102	0.23	down
A_32_P181297	0.004892	0.115	0.23	down

oebiotech_21800	0.006249	0.129	0.23	down
A_21_P0006599	0.010046	0.159	0.23	down
oebiotech_13650	0.011801	0.17	0.23	down
oebiotech_09252	0.013234	0.179	0.23	down
oebiotech_12465	0.023862	0.231	0.23	down
oebiotech_16016	0.029234	0.252	0.23	down
oebiotech_10780	7.02E-05	0.0284	0.24	down
A_21_P0010551	0.000262	0.0403	0.24	down
oebiotech_18595	0.000413	0.047	0.24	down
A_21_P0002235	0.001745	0.0779	0.24	down
oebiotech_12278	0.001761	0.0781	0.24	down
A_21_P0005210	0.002115	0.0838	0.24	down
oebiotech_00176	0.002265	0.0858	0.25	down
A_33_P3215744	0.006455	0.131	0.25	down
oebiotech_10847	0.013575	0.18	0.25	down
oebiotech_23830	0.04303	0.299	0.25	down
oebiotech_00451	0.000477	0.0502	0.26	down
oebiotech_11501	0.000698	0.0579	0.26	down
A_21_P0007726	0.000797	0.0595	0.26	down
oebiotech_19388	0.001334	0.0714	0.26	down
A_21_P0012817	0.001541	0.0754	0.26	down
A_21_P0013637	0.001656	0.0766	0.26	down
oebiotech_11805	0.005712	0.123	0.26	down
A_21_P0012180	0.008658	0.149	0.26	down
oebiotech_03028	0.010914	0.164	0.26	down
oebiotech_02153	0.013796	0.182	0.26	down
oebiotech_08146	0.031151	0.26	0.26	down
oebiotech_24449	7.27E-05	0.0284	0.27	down
oebiotech_24399	8.53E-05	0.0299	0.27	down
oebiotech_02305	0.000179	0.0369	0.27	down
oebiotech_25447	0.002736	0.0913	0.27	down
oebiotech_27821	0.007013	0.135	0.27	down
oebiotech_06032	0.013501	0.18	0.27	down
oebiotech_02480	0.019146	0.21	0.27	down
oebiotech_20807	0.049414	0.317	0.27	down
A_21_P0014182	0.000265	0.0403	0.28	down
A_24_P281009	0.00068	0.0578	0.28	down
A_21_P0011843	0.00384	0.105	0.28	down
oebiotech_25315	0.005626	0.123	0.28	down
oebiotech_19384	0.005772	0.124	0.28	down
A_21_P0007451	0.007458	0.139	0.28	down
A_21_P0011930	0.009008	0.151	0.28	down
oebiotech_21763	0.009627	0.156	0.28	down
A_21_P0013045	0.011175	0.165	0.28	down
A_21_P0008881	0.016386	0.197	0.28	down
A_21_P0005731	0.016506	0.198	0.28	down
oebiotech_26754	0.037237	0.28	0.28	down
oebiotech_19799	0.000271	0.0403	0.29	down
A_24_P200854	0.001067	0.0663	0.29	down
oebiotech_25646	0.001485	0.0746	0.29	down

oebiotech_20705	0.001537	0.0754	0.29	down
oebiotech_27706	0.001889	0.0799	0.29	down
A_21_P0006571	0.003319	0.099	0.29	down
oebiotech_11702	0.003714	0.103	0.29	down
oebiotech_24871	0.006543	0.131	0.29	down
oebiotech_14433	0.007966	0.143	0.29	down
oebiotech_18967	0.014106	0.184	0.29	down
oebiotech_05662	0.018167	0.205	0.29	down
oebiotech_03004	0.01854	0.207	0.29	down
oebiotech_11534	0.023978	0.232	0.29	down
oebiotech_07409	7.88E-05	0.0291	0.3	down
A_21_P0001279	0.000223	0.0388	0.3	down
oebiotech_00656	0.000519	0.0521	0.3	down
oebiotech_24189	0.000548	0.0534	0.3	down
A_33_P3226395	0.001015	0.0656	0.3	down
A_21_P0014322	0.01424	0.184	0.3	down
oebiotech_12923	0.020793	0.218	0.3	down
oebiotech_01910	0.000126	0.0329	0.31	down
oebiotech_01719	0.000873	0.0621	0.31	down
oebiotech_22854	0.001639	0.0766	0.31	down
oebiotech_18623	0.002524	0.0887	0.31	down
A_33_P3299934	0.003458	0.1	0.31	down
oebiotech_25585	0.003685	0.103	0.31	down
oebiotech_12776	0.005405	0.121	0.31	down
oebiotech_06922	0.006087	0.127	0.31	down
A_21_P0012221	0.006323	0.129	0.31	down
A_21_P0009030	0.009577	0.156	0.31	down
oebiotech_06661	0.014569	0.187	0.31	down
oebiotech_13624	0.015076	0.189	0.31	down
oebiotech_02290	0.015826	0.194	0.31	down
oebiotech_25460	0.016168	0.196	0.31	down
oebiotech_26953	0.029071	0.252	0.31	down
oebiotech_26987	0.029271	0.252	0.31	down
oebiotech_02693	0.038911	0.286	0.31	down
oebiotech_25193	2.81E-05	0.0207	0.32	down
oebiotech_22010	0.000093	0.0302	0.32	down
oebiotech_23848	0.000193	0.0372	0.32	down
oebiotech_22495	0.000236	0.0393	0.32	down
oebiotech_22845	0.000786	0.0592	0.32	down
oebiotech_25576	0.001249	0.0696	0.32	down
A_33_P3249696	0.002026	0.0826	0.32	down
A_33_P3245290	0.002289	0.0858	0.32	down
oebiotech_08131	0.003279	0.0985	0.32	down
oebiotech_21648	0.003642	0.102	0.32	down
oebiotech_01880	0.004628	0.113	0.32	down
A_21_P0002289	0.005107	0.117	0.32	down
oebiotech_00156	0.007442	0.139	0.32	down
A_33_P3261710	0.009619	0.156	0.32	down
oebiotech_14847	0.010567	0.162	0.32	down
A_21_P0012871	0.027983	0.248	0.32	down

oebiotech_16160	0.033975	0.27	0.32	down
oebiotech_20146	0.03611	0.277	0.32	down
oebiotech_14582	4.74E-05	0.0245	0.33	down
oebiotech_18777	0.000261	0.0403	0.33	down
A_21_P0007164	0.000735	0.0585	0.33	down
A_21_P0011657	0.000884	0.0625	0.33	down
A_21_P0010646	0.00121	0.0688	0.33	down
oebiotech_27799	0.002502	0.0886	0.33	down
A_33_P3401307	0.010956	0.164	0.33	down
oebiotech_25345	0.015379	0.191	0.33	down
oebiotech_10842	0.020269	0.215	0.33	down
oebiotech_15697	0.03587	0.276	0.33	down
oebiotech_12613	0.045955	0.307	0.33	down
A_21_P0013636	0.00119	0.0687	0.34	down
A_21_P0010610	0.002553	0.0891	0.34	down
A_21_P0013696	0.003471	0.1	0.34	down
oebiotech_25709	0.005104	0.117	0.34	down
oebiotech_17201	0.008847	0.15	0.34	down
A_21_P0004543	0.009023	0.151	0.34	down
oebiotech_23960	0.00961	0.156	0.34	down
oebiotech_27806	0.010983	0.164	0.34	down
oebiotech_22499	0.013369	0.179	0.34	down
oebiotech_07007	0.01343	0.18	0.34	down
oebiotech_26013	0.016417	0.197	0.34	down
oebiotech_28182	0.021759	0.222	0.34	down
oebiotech_05641	0.025261	0.237	0.34	down
A_21_P0010833	0.049555	0.318	0.34	down
A_21_P0004255	0.000242	0.0395	0.35	down
A_21_P0000841	0.000367	0.0453	0.35	down
A_21_P0010651	0.000416	0.0471	0.35	down
oebiotech_02021	0.000789	0.0593	0.35	down
oebiotech_27852	0.00312	0.0959	0.35	down
A_33_P3279660	0.004886	0.115	0.35	down
A_21_P0003190	0.006333	0.13	0.35	down
oebiotech_11934	0.007509	0.14	0.35	down
oebiotech_17231	0.007672	0.141	0.35	down
oebiotech_18524	0.009107	0.152	0.35	down
A_33_P3260964	0.010018	0.158	0.35	down
A_33_P3259973	0.013689	0.181	0.35	down
oebiotech_15268	0.021145	0.22	0.35	down
A_21_P0012426	0.025521	0.238	0.35	down
oebiotech_19284	0.042939	0.299	0.35	down
oebiotech_03313	0.000145	0.0353	0.36	down
oebiotech_14541	0.000169	0.0364	0.36	down
oebiotech_00450	0.000197	0.0374	0.36	down
oebiotech_11569	0.000472	0.0502	0.36	down
oebiotech_25584	0.000571	0.0546	0.36	down
A_21_P0005546	0.001143	0.0675	0.36	down
oebiotech_02734	0.002675	0.0904	0.36	down
A_21_P0004451	0.003461	0.1	0.36	down

oebiotech_13461	0.004077	0.108	0.36	down
oebiotech_04590	0.004703	0.114	0.36	down
oebiotech_24641	0.02	0.214	0.36	down
oebiotech_04953	0.025612	0.238	0.36	down
oebiotech_11268	0.02602	0.24	0.36	down
oebiotech_17837	0.030394	0.257	0.36	down
oebiotech_11533	0.038237	0.283	0.36	down
oebiotech_16123	0.000274	0.0403	0.37	down
oebiotech_20311	0.000693	0.0579	0.37	down
A_21_P0013370	0.001042	0.066	0.37	down
oebiotech_24673	0.001126	0.0672	0.37	down
A_21_P0009023	0.001754	0.0779	0.37	down
A_23_P112452	0.004189	0.11	0.37	down
A_23_P96658	0.004236	0.11	0.37	down
oebiotech_15738	0.005546	0.122	0.37	down
A_21_P0014441	0.005686	0.123	0.37	down
A_23_P216935	0.006016	0.126	0.37	down
A_21_P0012241	0.006154	0.128	0.37	down
oebiotech_16517	0.006585	0.132	0.37	down
oebiotech_21377	0.006766	0.133	0.37	down
oebiotech_07339	0.017289	0.201	0.37	down
oebiotech_19253	0.017642	0.203	0.37	down
oebiotech_26829	0.019916	0.213	0.37	down
A_21_P0010760	0.021643	0.222	0.37	down
A_21_P0000822	0.035907	0.276	0.37	down
oebiotech_21166	0.040703	0.292	0.37	down
oebiotech_04518	0.0431	0.299	0.37	down
A_23_P251232	0.000182	0.037	0.38	down
A_21_P0002860	0.000996	0.0651	0.38	down
oebiotech_02251	0.00108	0.0667	0.38	down
oebiotech_07960	0.001229	0.0692	0.38	down
oebiotech_07736	0.001624	0.0766	0.38	down
oebiotech_19290	0.00276	0.0914	0.38	down
oebiotech_10801	0.003521	0.101	0.38	down
A_23_P364792	0.004308	0.11	0.38	down
oebiotech_04972	0.004545	0.113	0.38	down
oebiotech_17074	0.004958	0.116	0.38	down
oebiotech_07970	0.006303	0.129	0.38	down
A_21_P0011710	0.00828	0.146	0.38	down
A_21_P0013643	0.008627	0.149	0.38	down
oebiotech_26281	0.00884	0.15	0.38	down
oebiotech_16038	0.02073	0.218	0.38	down
A_21_P0013451	9.39E-05	0.0302	0.39	down
oebiotech_24766	0.000141	0.0348	0.39	down
A_33_P3679936	0.000944	0.0636	0.39	down
A_19_P00317631	0.001017	0.0656	0.39	down
oebiotech_10696	0.001198	0.0687	0.39	down
A_21_P0013644	0.002247	0.0856	0.39	down
oebiotech_20815	0.002741	0.0913	0.39	down
oebiotech_25501	0.003722	0.103	0.39	down

A_21_P0009394	0.003737	0.103	0.39	down
A_32_P221305	0.00375	0.103	0.39	down
oebiotech_11510	0.004221	0.11	0.39	down
oebiotech_21791	0.006297	0.129	0.39	down
oebiotech_13394	0.007601	0.14	0.39	down
oebiotech_16788	0.009386	0.154	0.39	down
A_33_P3241661	0.01121	0.165	0.39	down
oebiotech_21073	0.013461	0.18	0.39	down
oebiotech_25231	0.01507	0.189	0.39	down
oebiotech_24743	0.021252	0.22	0.39	down
oebiotech_21802	0.022082	0.224	0.39	down
oebiotech_20944	0.0227	0.227	0.39	down
A_21_P0007284	0.025666	0.238	0.39	down
oebiotech_22184	0.025894	0.239	0.39	down
oebiotech_24461	0.000004	0.0151	0.4	down
A_21_P0013600	9.2E-06	0.0158	0.4	down
oebiotech_11739	0.000563	0.0542	0.4	down
oebiotech_24334	0.000578	0.0549	0.4	down
oebiotech_09775	0.000653	0.057	0.4	down
A_21_P0001753	0.000799	0.0595	0.4	down
A_21_P0013635	0.001242	0.0694	0.4	down
A_21_P0014467	0.003059	0.0953	0.4	down
A_21_P0011478	0.004398	0.111	0.4	down
A_21_P0012868	0.005699	0.123	0.4	down
oebiotech_14912	0.010266	0.16	0.4	down
A_21_P0004187	0.015242	0.19	0.4	down
oebiotech_25753	0.022348	0.225	0.4	down
A_21_P0008654	0.045899	0.307	0.4	down
oebiotech_16530	4.57E-05	0.0239	0.41	down
oebiotech_25502	0.000143	0.0349	0.41	down
oebiotech_10157	0.00089	0.0625	0.41	down
oebiotech_11191	0.001256	0.0698	0.41	down
oebiotech_07315	0.001397	0.0728	0.41	down
oebiotech_16158	0.004095	0.109	0.41	down
oebiotech_19023	0.004526	0.113	0.41	down
A_24_P145529	0.005261	0.119	0.41	down
oebiotech_16656	0.009475	0.155	0.41	down
oebiotech_23532	0.01501	0.189	0.41	down
A_21_P0002500	0.032135	0.263	0.41	down
oebiotech_05299	0.035062	0.273	0.41	down
oebiotech_23543	0.039522	0.288	0.41	down
A_33_P3378383	0.049767	0.318	0.41	down
oebiotech_14791	9.47E-05	0.0302	0.42	down
A_21_P0008643	0.0002	0.0374	0.42	down
oebiotech_11800	0.000348	0.0444	0.42	down
oebiotech_22564	0.000782	0.0592	0.42	down
A_23_P350754	0.00128	0.0701	0.42	down
oebiotech_19592	0.001537	0.0754	0.42	down
A_21_P0001252	0.001638	0.0766	0.42	down
oebiotech_14266	0.00199	0.0819	0.42	down

oebiotech_20797	0.003433	0.1	0.42	down
A_21_P0000827	0.003807	0.104	0.42	down
oebiotech_09030	0.005445	0.121	0.42	down
oebiotech_16402	0.005582	0.122	0.42	down
oebiotech_01595	0.007936	0.143	0.42	down
oebiotech_20849	0.010225	0.16	0.42	down
oebiotech_18035	0.010606	0.162	0.42	down
A_21_P0003132	0.01072	0.163	0.42	down
A_32_P204795	0.0129	0.176	0.42	down
A_21_P0010924	0.016056	0.195	0.42	down
oebiotech_11428	0.018174	0.205	0.42	down
oebiotech_24094	0.023281	0.229	0.42	down
oebiotech_05123	0.031322	0.26	0.42	down
oebiotech_00763	0.031759	0.262	0.42	down
oebiotech_01683	0.039496	0.288	0.42	down
oebiotech_11475	0.000096	0.0302	0.43	down
oebiotech_21498	0.000173	0.0364	0.43	down
oebiotech_05380	0.000272	0.0403	0.43	down
oebiotech_10158	0.000356	0.0447	0.43	down
A_21_P0011890	0.00058	0.055	0.43	down
A_21_P0010306	0.001053	0.0661	0.43	down
A_21_P0013586	0.001263	0.0698	0.43	down
A_24_P119813	0.001357	0.072	0.43	down
oebiotech_01537	0.001375	0.0723	0.43	down
oebiotech_26314	0.001836	0.0791	0.43	down
A_19_P00808846	0.002826	0.0921	0.43	down
oebiotech_24347	0.004399	0.111	0.43	down
oebiotech_13340	0.004531	0.113	0.43	down
A_21_P0014619	0.005039	0.117	0.43	down
oebiotech_21423	0.006494	0.131	0.43	down
oebiotech_27851	0.007056	0.135	0.43	down
oebiotech_12889	0.008711	0.149	0.43	down
oebiotech_21195	0.011759	0.169	0.43	down
oebiotech_24882	0.012157	0.172	0.43	down
oebiotech_05418	0.012874	0.176	0.43	down
A_21_P0011895	0.013107	0.178	0.43	down
oebiotech_06469	0.013513	0.18	0.43	down
oebiotech_07732	0.015147	0.19	0.43	down
oebiotech_24881	0.020837	0.218	0.43	down
oebiotech_27837	0.021975	0.223	0.43	down
oebiotech_14342	0.024568	0.234	0.43	down
oebiotech_08127	0.027899	0.247	0.43	down
oebiotech_17813	0.049873	0.318	0.43	down
A_21_P0010340	3.91E-05	0.0226	0.44	down
A_33_P3325107	0.000084	0.0297	0.44	down
oebiotech_23653	0.001056	0.0662	0.44	down
oebiotech_02173	0.001103	0.0668	0.44	down
oebiotech_16718	0.001398	0.0728	0.44	down
oebiotech_20384	0.001838	0.0791	0.44	down
oebiotech_08587	0.002522	0.0887	0.44	down

oebiotech_00084	0.003139	0.0963	0.44	down
oebiotech_22012	0.003244	0.0982	0.44	down
oebiotech_27731	0.003368	0.0995	0.44	down
oebiotech_07848	0.004479	0.112	0.44	down
A_21_P0008799	0.004561	0.113	0.44	down
oebiotech_20709	0.006442	0.131	0.44	down
A_21_P0011980	0.007683	0.141	0.44	down
oebiotech_12872	0.008442	0.148	0.44	down
A_33_P3386150	0.00971	0.157	0.44	down
oebiotech_20977	0.009943	0.158	0.44	down
A_21_P0004803	0.012634	0.175	0.44	down
oebiotech_24674	0.012767	0.176	0.44	down
A_21_P0013079	0.015161	0.19	0.44	down
oebiotech_11439	0.021904	0.223	0.44	down
A_21_P0005955	0.022894	0.228	0.44	down
oebiotech_12417	0.025143	0.237	0.44	down
oebiotech_28104	0.025363	0.238	0.44	down
oebiotech_17246	0.026302	0.241	0.44	down
oebiotech_24865	0.027453	0.246	0.44	down
oebiotech_03526	0.03218	0.263	0.44	down
oebiotech_20023	0.035202	0.274	0.44	down
oebiotech_14722	0.044034	0.302	0.44	down
A_21_P0010757	0.000025	0.0203	0.45	down
oebiotech_25434	0.000177	0.0368	0.45	down
oebiotech_20976	0.000511	0.0519	0.45	down
A_23_P251268	0.001034	0.0659	0.45	down
oebiotech_18893	0.001453	0.0739	0.45	down
oebiotech_07183	0.001528	0.0754	0.45	down
A_33_P3260223	0.001715	0.0776	0.45	down
oebiotech_09700	0.002075	0.0833	0.45	down
oebiotech_16807	0.002307	0.086	0.45	down
oebiotech_26485	0.002428	0.0877	0.45	down
oebiotech_15392	0.003732	0.103	0.45	down
oebiotech_24757	0.004573	0.113	0.45	down
oebiotech_25107	0.004671	0.114	0.45	down
A_21_P0014247	0.004695	0.114	0.45	down
A_21_P0011197	0.006031	0.126	0.45	down
oebiotech_10160	0.006829	0.133	0.45	down
oebiotech_13788	0.009184	0.152	0.45	down
oebiotech_03415	0.010577	0.162	0.45	down
A_33_P3277883	0.016323	0.197	0.45	down
oebiotech_11053	0.021319	0.22	0.45	down
oebiotech_12484	0.024166	0.233	0.45	down
A_19_P00810009	0.028428	0.249	0.45	down
oebiotech_25366	0.030196	0.256	0.45	down
oebiotech_26345	0.032339	0.264	0.45	down
oebiotech_27225	0.041463	0.294	0.45	down
A_19_P00320242	0.042486	0.297	0.45	down
oebiotech_17261	0.044946	0.304	0.45	down
oebiotech_13753	0.046711	0.309	0.45	down

oebiotech_16923	0.000171	0.0364	0.46	down
oebiotech_01242	0.00022	0.0385	0.46	down
oebiotech_11452	0.000304	0.0416	0.46	down
A_21_P0008581	0.000913	0.063	0.46	down
A_21_P0011722	0.001571	0.0758	0.46	down
oebiotech_23925	0.002086	0.0835	0.46	down
oebiotech_16035	0.002657	0.0902	0.46	down
oebiotech_16565	0.002685	0.0905	0.46	down
oebiotech_27823	0.004074	0.108	0.46	down
oebiotech_14829	0.004421	0.112	0.46	down
oebiotech_19952	0.005009	0.116	0.46	down
oebiotech_09121	0.005815	0.124	0.46	down
oebiotech_00381	0.006418	0.13	0.46	down
oebiotech_14768	0.009294	0.153	0.46	down
oebiotech_14545	0.009855	0.157	0.46	down
oebiotech_17509	0.010218	0.16	0.46	down
oebiotech_19570	0.015407	0.191	0.46	down
A_21_P0002585	0.019035	0.21	0.46	down
oebiotech_15906	0.021016	0.219	0.46	down
A_21_P0014380	0.026412	0.242	0.46	down
oebiotech_02617	0.03425	0.27	0.46	down
oebiotech_13860	0.041773	0.295	0.46	down
A_21_P0012922	0.000039	0.0226	0.47	down
oebiotech_00241	0.000118	0.0326	0.47	down
oebiotech_25572	0.000347	0.0444	0.47	down
oebiotech_06572	0.000874	0.0621	0.47	down
oebiotech_25710	0.002743	0.0913	0.47	down
oebiotech_25649	0.003447	0.1	0.47	down
A_33_P3411744	0.003945	0.106	0.47	down
oebiotech_26821	0.005963	0.126	0.47	down
oebiotech_26700	0.006147	0.128	0.47	down
oebiotech_12870	0.006365	0.13	0.47	down
oebiotech_21904	0.007565	0.14	0.47	down
oebiotech_14012	0.00826	0.146	0.47	down
A_21_P0006412	0.008763	0.15	0.47	down
oebiotech_25988	0.008796	0.15	0.47	down
A_33_P3277367	0.009437	0.154	0.47	down
A_32_P10133	0.01144	0.167	0.47	down
oebiotech_03889	0.012602	0.175	0.47	down
oebiotech_12960	0.012764	0.176	0.47	down
A_33_P3275280	0.012881	0.176	0.47	down
oebiotech_19509	0.013186	0.179	0.47	down
oebiotech_19048	0.016676	0.198	0.47	down
oebiotech_11451	0.017808	0.204	0.47	down
oebiotech_02061	0.019054	0.21	0.47	down
A_21_P0008324	0.019276	0.21	0.47	down
A_21_P0013659	0.01969	0.212	0.47	down
oebiotech_21945	0.024626	0.235	0.47	down
A_21_P0003824	0.029696	0.254	0.47	down
A_23_P69941	0.03175	0.262	0.47	down

A_33_P3371115	0.037202	0.28	0.47	down
oebiotech_21080	0.040833	0.292	0.47	down
oebiotech_21229	0.041845	0.295	0.47	down
A_19_P00318183	0.042555	0.297	0.47	down
A_21_P0009266	0.04626	0.308	0.47	down
oebiotech_16554	0.000591	0.0553	0.48	down
oebiotech_02615	0.002049	0.083	0.48	down
oebiotech_16156	0.002122	0.0838	0.48	down
A_21_P0013057	0.002502	0.0886	0.48	down
A_33_P3335735	0.002891	0.0931	0.48	down
oebiotech_27825	0.003838	0.105	0.48	down
oebiotech_17928	0.007118	0.136	0.48	down
oebiotech_15428	0.008427	0.147	0.48	down
oebiotech_27227	0.009201	0.153	0.48	down
A_33_P3883985	0.010621	0.162	0.48	down
A_21_P0000539	0.011903	0.17	0.48	down
oebiotech_13202	0.013182	0.179	0.48	down
oebiotech_19009	0.013985	0.183	0.48	down
A_19_P00800681	0.015883	0.194	0.48	down
A_21_P0006149	0.016636	0.198	0.48	down
oebiotech_19360	0.020113	0.215	0.48	down
oebiotech_07611	0.020385	0.216	0.48	down
oebiotech_23126	0.022923	0.228	0.48	down
oebiotech_20898	0.023961	0.232	0.48	down
A_21_P0013568	0.025731	0.239	0.48	down
oebiotech_07000	0.027986	0.248	0.48	down
A_21_P0014332	0.027996	0.248	0.48	down
oebiotech_14112	0.031721	0.262	0.48	down
oebiotech_13728	0.035996	0.277	0.48	down
A_21_P0011768	0.038711	0.285	0.48	down
oebiotech_22575	0.043711	0.301	0.48	down
oebiotech_04666	0.000108	0.0318	0.49	down
oebiotech_25711	0.000313	0.0419	0.49	down
oebiotech_20892	0.000703	0.058	0.49	down
oebiotech_26997	0.001086	0.0668	0.49	down
A_21_P0001411	0.00147	0.0744	0.49	down
A_23_P433218	0.001615	0.0765	0.49	down
oebiotech_24457	0.001617	0.0765	0.49	down
oebiotech_21487	0.001658	0.0766	0.49	down
oebiotech_17469	0.002634	0.0898	0.49	down
oebiotech_13981	0.002901	0.0932	0.49	down
oebiotech_21521	0.003249	0.0982	0.49	down
A_21_P0000899	0.003517	0.101	0.49	down
A_21_P0014665	0.006665	0.132	0.49	down
oebiotech_08354	0.006696	0.132	0.49	down
A_19_P00801088	0.007218	0.137	0.49	down
oebiotech_26484	0.00771	0.142	0.49	down
oebiotech_27224	0.007886	0.143	0.49	down
A_33_P3217786	0.007936	0.143	0.49	down
A_21_P0011401	0.009605	0.156	0.49	down

A_33_P3263463	0.011255	0.166	0.49	down
A_21_P0000454	0.012823	0.176	0.49	down
A_33_P3255587	0.017961	0.205	0.49	down
A_33_P3408837	0.019212	0.21	0.49	down
oebiotech_20446	0.024525	0.234	0.49	down
oebiotech_22768	0.02516	0.237	0.49	down
oebiotech_14792	0.026802	0.243	0.49	down
oebiotech_26823	0.026817	0.243	0.49	down
A_21_P0000229	0.030587	0.257	0.49	down
oebiotech_26715	0.032743	0.265	0.49	down
oebiotech_26713	0.036084	0.277	0.49	down
A_21_P0014241	0.036782	0.279	0.49	down
oebiotech_25772	0.043985	0.302	0.49	down
A_19_P00807053	0.043986	0.302	0.49	down
oebiotech_19176	0.000704	0.0581	2.01	up
oebiotech_15035	0.002942	0.0935	2.01	up
oebiotech_15228	0.004005	0.107	2.01	up
oebiotech_11026	0.010668	0.163	2.01	up
A_21_P0012345	0.012819	0.176	2.01	up
oebiotech_21427	0.014886	0.188	2.01	up
oebiotech_19324	0.033033	0.266	2.01	up
oebiotech_11434	0.038945	0.286	2.01	up
oebiotech_09078	3.34E-05	0.0218	2.02	up
A_21_P0014790	0.002204	0.0849	2.02	up
oebiotech_22884	0.004402	0.111	2.02	up
A_23_P210164	0.004974	0.116	2.02	up
oebiotech_27098	0.009427	0.154	2.02	up
oebiotech_18935	0.013878	0.182	2.02	up
A_21_P0001601	0.021263	0.22	2.02	up
oebiotech_27081	0.022532	0.226	2.02	up
A_21_P0012536	0.034202	0.27	2.02	up
oebiotech_00875	0.044446	0.303	2.02	up
oebiotech_20072	0.045802	0.306	2.02	up
A_33_P3355926	5.74E-05	0.0256	2.03	up
oebiotech_06358	0.000235	0.0392	2.03	up
oebiotech_02536	0.000263	0.0403	2.03	up
oebiotech_22620	0.000344	0.0442	2.03	up
oebiotech_16771	0.000884	0.0625	2.03	up
oebiotech_05591	0.004033	0.108	2.03	up
oebiotech_01050	0.006358	0.13	2.03	up
oebiotech_04235	0.006839	0.133	2.03	up
oebiotech_10992	0.007193	0.137	2.03	up
oebiotech_19728	0.008629	0.149	2.03	up
A_21_P0014758	0.009756	0.157	2.03	up
oebiotech_12068	0.010318	0.16	2.03	up
oebiotech_00877	0.022483	0.226	2.03	up
oebiotech_12442	0.037284	0.28	2.03	up
oebiotech_19179	0.00052	0.0521	2.04	up
A_23_P99204	0.000719	0.0585	2.04	up
A_21_P0014871	0.001105	0.0668	2.04	up

oebiotech_14178	0.006639	0.132	2.04	up
oebiotech_14094	0.009409	0.154	2.04	up
oebiotech_22710	0.01029	0.16	2.04	up
A_19_P00321973	0.016645	0.198	2.04	up
A_19_P00315798	0.025505	0.238	2.04	up
A_21_P0014518	0.028393	0.249	2.04	up
oebiotech_03363	0.030408	0.257	2.04	up
oebiotech_07483	0.035006	0.273	2.04	up
oebiotech_20535	0.000272	0.0403	2.05	up
oebiotech_11328	0.000962	0.0643	2.05	up
oebiotech_11329	0.00225	0.0856	2.05	up
oebiotech_19612	0.005452	0.121	2.05	up
oebiotech_16348	0.005948	0.126	2.05	up
oebiotech_04688	0.011254	0.166	2.05	up
oebiotech_13663	0.024116	0.232	2.05	up
oebiotech_08038	0.025628	0.238	2.05	up
A_21_P0010486	0.032627	0.265	2.05	up
oebiotech_18187	0.033142	0.267	2.05	up
A_21_P0005951	0.036932	0.279	2.05	up
A_21_P0014875	0.037411	0.281	2.05	up
oebiotech_22756	0.048883	0.316	2.05	up
A_24_P687326	0.000271	0.0403	2.06	up
oebiotech_14523	0.000485	0.0507	2.06	up
oebiotech_20901	0.001098	0.0668	2.06	up
oebiotech_11335	0.002524	0.0887	2.06	up
oebiotech_21416	0.003024	0.0945	2.06	up
oebiotech_10773	0.003258	0.0983	2.06	up
oebiotech_11502	0.007704	0.141	2.06	up
A_19_P00317871	0.010146	0.159	2.06	up
oebiotech_25592	0.01198	0.171	2.06	up
oebiotech_12285	0.012094	0.171	2.06	up
A_33_P3378707	0.013073	0.178	2.06	up
oebiotech_15553	0.014813	0.188	2.06	up
oebiotech_04859	0.019953	0.214	2.06	up
A_21_P0013040	0.02033	0.216	2.06	up
A_21_P0010933	0.021003	0.219	2.06	up
A_21_P0001717	0.035525	0.275	2.06	up
A_21_P0000417	0.044258	0.302	2.06	up
oebiotech_16916	0.002611	0.0894	2.07	up
oebiotech_17500	0.010769	0.163	2.07	up
A_19_P00318600	0.038827	0.286	2.07	up
A_19_P00318768	0.041338	0.294	2.07	up
oebiotech_03010	1.95E-05	0.0191	2.08	up
oebiotech_07867	0.000207	0.0376	2.08	up
oebiotech_18323	0.001086	0.0668	2.08	up
oebiotech_02980	0.006862	0.133	2.08	up
oebiotech_20842	0.014033	0.183	2.08	up
A_21_P0011581	0.017318	0.201	2.08	up
oebiotech_28308	0.031677	0.262	2.08	up
oebiotech_19321	0.040022	0.289	2.08	up

A_21_P0008417	0.002002	0.0821	2.09	up
A_21_P0013712	0.002011	0.0823	2.09	up
oebiotech_10805	0.008703	0.149	2.09	up
oebiotech_17320	0.042502	0.297	2.09	up
oebiotech_14881	0.001249	0.0696	2.1	up
oebiotech_22701	0.002122	0.0838	2.1	up
oebiotech_14851	0.004363	0.111	2.1	up
oebiotech_08249	0.00491	0.116	2.1	up
oebiotech_27920	0.005105	0.117	2.1	up
oebiotech_11879	0.011229	0.165	2.1	up
oebiotech_22031	0.012565	0.174	2.1	up
oebiotech_04592	0.01355	0.18	2.1	up
oebiotech_08076	0.018537	0.207	2.1	up
A_19_P00804711	0.027743	0.247	2.1	up
oebiotech_16622	0.029556	0.254	2.1	up
A_21_P0006108	0.031964	0.263	2.1	up
oebiotech_11188	0.04037	0.291	2.1	up
A_21_P0011906	0.042457	0.297	2.1	up
oebiotech_22759	0.000049	0.0249	2.11	up
oebiotech_21546	0.000452	0.0492	2.11	up
oebiotech_18025	0.002321	0.086	2.11	up
oebiotech_12173	0.003464	0.1	2.11	up
oebiotech_14213	0.010293	0.16	2.11	up
A_21_P0008920	0.013771	0.182	2.11	up
oebiotech_21260	0.015976	0.195	2.11	up
oebiotech_05349	0.036808	0.279	2.11	up
oebiotech_26814	0.039763	0.289	2.11	up
A_21_P0012080	0.043833	0.301	2.11	up
oebiotech_05128	0.001698	0.0773	2.12	up
A_33_P3420909	0.007783	0.142	2.12	up
A_21_P0003375	0.014378	0.185	2.12	up
oebiotech_23906	0.037647	0.282	2.12	up
oebiotech_22592	0.041232	0.293	2.12	up
oebiotech_15199	0.001537	0.0754	2.13	up
oebiotech_10991	0.002451	0.0879	2.13	up
oebiotech_16161	0.00345	0.1	2.13	up
oebiotech_17888	0.007645	0.141	2.13	up
oebiotech_17505	0.016943	0.199	2.13	up
A_21_P0009560	0.022706	0.227	2.13	up
oebiotech_21677	0.022997	0.228	2.13	up
A_21_P0008957	0.047866	0.313	2.13	up
A_21_P0004810	0.000971	0.0645	2.14	up
oebiotech_11212	0.001383	0.0725	2.14	up
oebiotech_14028	0.012057	0.171	2.14	up
oebiotech_02128	0.030626	0.258	2.14	up
oebiotech_12862	0.000215	0.0381	2.15	up
A_19_P00317045	0.005337	0.12	2.15	up
oebiotech_14833	0.007937	0.143	2.15	up
oebiotech_26904	0.015108	0.19	2.15	up
A_21_P0012436	0.001169	0.068	2.16	up

oebiotech_12597	0.006399	0.13	2.16	up
oebiotech_21820	0.013302	0.179	2.16	up
oebiotech_19295	0.013458	0.18	2.16	up
oebiotech_19707	0.014344	0.185	2.16	up
oebiotech_01608	0.020124	0.215	2.16	up
oebiotech_09397	0.035456	0.275	2.16	up
A_21_P0014112	0.001201	0.0687	2.17	up
oebiotech_17166	0.003748	0.103	2.17	up
oebiotech_02518	0.007479	0.139	2.17	up
oebiotech_15210	0.007512	0.14	2.17	up
A_21_P0010798	0.017025	0.2	2.17	up
oebiotech_12316	0.017984	0.205	2.17	up
oebiotech_08960	0.02043	0.216	2.17	up
A_21_P0004566	0.024498	0.234	2.17	up
oebiotech_26141	0.027214	0.245	2.17	up
A_19_P00318175	0.044055	0.302	2.17	up
oebiotech_15735	0.000654	0.057	2.18	up
oebiotech_02089	0.004115	0.109	2.18	up
oebiotech_24818	0.006914	0.134	2.18	up
A_21_P0004476	0.00847	0.148	2.18	up
A_33_P3369029	0.008837	0.15	2.18	up
oebiotech_04167	0.009585	0.156	2.18	up
oebiotech_07422	0.009995	0.158	2.18	up
oebiotech_05302	0.019491	0.211	2.18	up
oebiotech_11332	0.047647	0.312	2.18	up
oebiotech_14206	0.001334	0.0714	2.19	up
oebiotech_11086	0.001838	0.0791	2.19	up
A_21_P0012077	0.002093	0.0836	2.19	up
oebiotech_26716	0.004821	0.115	2.19	up
oebiotech_00272	0.00813	0.145	2.19	up
oebiotech_05589	0.010372	0.161	2.19	up
oebiotech_20164	0.031635	0.261	2.19	up
A_21_P0013757	0.037785	0.282	2.19	up
oebiotech_05874	0.000694	0.0579	2.2	up
oebiotech_02415	0.002399	0.0875	2.2	up
oebiotech_27663	0.003532	0.101	2.2	up
A_21_P0007515	0.008233	0.146	2.2	up
oebiotech_01183	0.013354	0.179	2.2	up
oebiotech_16464	0.017835	0.204	2.2	up
A_21_P0010657	0.018191	0.205	2.2	up
oebiotech_04560	0.020504	0.217	2.2	up
oebiotech_02058	0.023142	0.228	2.2	up
A_21_P0007523	0.024318	0.233	2.2	up
A_21_P0012150	0.027397	0.245	2.2	up
A_21_P0011951	0.034666	0.272	2.2	up
oebiotech_20845	0.044035	0.302	2.2	up
A_19_P00319646	0.045148	0.305	2.2	up
A_19_P00322705	0.045419	0.305	2.2	up
A_33_P3334384	0.025193	0.237	2.21	up
oebiotech_12003	0.037711	0.282	2.21	up

A_21_P0014459	0.039229	0.287	2.21	up
oebiotech_17173	0.039813	0.289	2.21	up
A_21_P0008010	0.046915	0.31	2.21	up
oebiotech_01486	0.000324	0.0427	2.22	up
oebiotech_11336	0.007717	0.142	2.22	up
oebiotech_22230	0.014553	0.187	2.22	up
A_19_P00319528	0.020548	0.217	2.22	up
A_21_P0000727	0.042777	0.298	2.22	up
oebiotech_10636	0.000188	0.0372	2.23	up
oebiotech_26636	0.001408	0.0728	2.23	up
oebiotech_23782	0.006949	0.134	2.23	up
oebiotech_11257	0.007707	0.142	2.23	up
oebiotech_08229	0.009385	0.154	2.23	up
oebiotech_18543	0.00944	0.154	2.23	up
oebiotech_21174	0.018924	0.209	2.23	up
A_21_P0003941	0.021743	0.222	2.23	up
oebiotech_24473	0.027989	0.248	2.23	up
oebiotech_10876	0.036801	0.279	2.23	up
oebiotech_09054	4.22E-05	0.0229	2.24	up
oebiotech_21374	0.000389	0.046	2.24	up
oebiotech_21018	0.002318	0.086	2.24	up
A_21_P0014664	0.003502	0.101	2.24	up
oebiotech_26135	0.01068	0.163	2.24	up
oebiotech_00407	0.017999	0.205	2.24	up
oebiotech_26116	0.021148	0.22	2.24	up
A_23_P39881	0.023815	0.231	2.24	up
oebiotech_21413	0.000928	0.0632	2.25	up
A_21_P0011179	0.001312	0.0708	2.25	up
A_21_P0005739	0.002768	0.0915	2.25	up
oebiotech_16526	0.00316	0.0967	2.25	up
oebiotech_11902	0.023511	0.23	2.25	up
A_21_P0014319	0.026631	0.242	2.25	up
oebiotech_26875	0.029727	0.254	2.25	up
oebiotech_01769	0.032694	0.265	2.25	up
A_21_P0005297	0.033739	0.269	2.25	up
oebiotech_15801	5.35E-05	0.0253	2.26	up
A_21_P0008836	0.00078	0.0592	2.26	up
oebiotech_06468	0.000914	0.063	2.26	up
oebiotech_17710	0.002324	0.086	2.26	up
oebiotech_00965	0.00472	0.114	2.26	up
oebiotech_16245	0.005388	0.121	2.26	up
oebiotech_11116	0.014674	0.187	2.26	up
oebiotech_08967	0.046947	0.31	2.26	up
oebiotech_26892	0.007257	0.137	2.27	up
A_21_P0011966	0.011006	0.164	2.27	up
oebiotech_00408	0.011595	0.168	2.27	up
oebiotech_25354	0.021176	0.22	2.27	up
oebiotech_16320	0.026428	0.242	2.27	up
A_21_P0012095	0.037439	0.281	2.27	up
A_19_P00321124	0.048984	0.316	2.27	up

A_33_P3249654	0.001398	0.0728	2.28	up
A_21_P0012078	0.0028	0.0918	2.28	up
oebiotech_11082	0.007965	0.143	2.28	up
oebiotech_15263	0.017276	0.201	2.28	up
oebiotech_06132	9.13E-05	0.0302	2.29	up
A_21_P0008205	0.002821	0.092	2.29	up
oebiotech_13322	0.005385	0.121	2.29	up
oebiotech_17842	0.015342	0.191	2.29	up
oebiotech_04584	0.028061	0.248	2.29	up
oebiotech_27330	0.03125	0.26	2.29	up
oebiotech_12284	0.000283	0.0407	2.3	up
oebiotech_11728	0.000289	0.0409	2.3	up
oebiotech_25918	0.000724	0.0585	2.3	up
A_24_P506977	0.003586	0.102	2.3	up
oebiotech_14574	0.005597	0.123	2.3	up
oebiotech_17913	0.008771	0.15	2.3	up
oebiotech_12246	0.016566	0.198	2.3	up
oebiotech_22109	0.016718	0.199	2.3	up
oebiotech_01703	0.030973	0.259	2.3	up
oebiotech_03129	0.031121	0.26	2.3	up
oebiotech_19741	0.045275	0.305	2.3	up
oebiotech_12040	0.000889	0.0625	2.31	up
oebiotech_04204	0.001557	0.0757	2.31	up
oebiotech_26637	0.001655	0.0766	2.31	up
oebiotech_01779	0.001751	0.0779	2.31	up
oebiotech_11233	0.002409	0.0875	2.31	up
A_33_P3225630	0.007732	0.142	2.31	up
oebiotech_22639	0.010314	0.16	2.31	up
oebiotech_26745	0.012395	0.173	2.31	up
oebiotech_11914	0.014472	0.186	2.31	up
oebiotech_04796	0.028854	0.251	2.31	up
A_21_P0008160	0.034153	0.27	2.32	up
A_21_P0014425	0.039288	0.287	2.32	up
A_21_P0008913	0.001277	0.0701	2.33	up
oebiotech_21470	0.004724	0.114	2.33	up
oebiotech_11123	0.027271	0.245	2.33	up
oebiotech_20832	0.027875	0.247	2.33	up
oebiotech_15512	0.002573	0.0892	2.34	up
oebiotech_17879	0.010297	0.16	2.34	up
A_21_P0000338	0.013911	0.182	2.34	up
oebiotech_11271	0.016789	0.199	2.34	up
A_32_P214178	0.018587	0.207	2.34	up
oebiotech_18673	0.000285	0.0408	2.35	up
A_21_P0001958	0.002584	0.0893	2.35	up
oebiotech_11134	0.010776	0.163	2.35	up
A_23_P6708	0.013312	0.179	2.35	up
A_21_P0012287	0.015472	0.192	2.35	up
oebiotech_15717	0.00007	0.0284	2.36	up
oebiotech_22611	0.000128	0.0331	2.36	up
oebiotech_15779	0.000431	0.0481	2.36	up

A_21_P0011853	0.000477	0.0502	2.36	up
oebiotech_13479	0.014242	0.184	2.36	up
A_19_P00317420	0.027058	0.244	2.36	up
oebiotech_17784	0.027801	0.247	2.36	up
oebiotech_11548	0.028584	0.25	2.36	up
oebiotech_16027	0.035411	0.274	2.36	up
oebiotech_08176	6.26E-05	0.0266	2.37	up
oebiotech_15619	0.000153	0.0355	2.37	up
A_21_P0000543	0.001299	0.0705	2.37	up
oebiotech_28352	0.001474	0.0744	2.37	up
oebiotech_19912	0.002134	0.0839	2.37	up
oebiotech_24756	0.006372	0.13	2.37	up
oebiotech_20876	0.019721	0.212	2.37	up
oebiotech_06056	7.31E-05	0.0284	2.38	up
oebiotech_19417	0.000386	0.046	2.38	up
oebiotech_02000	0.00053	0.0525	2.38	up
oebiotech_10611	0.004307	0.11	2.38	up
A_21_P0008513	0.004838	0.115	2.38	up
oebiotech_08310	0.028404	0.249	2.38	up
A_21_P0001400	0.037126	0.28	2.38	up
oebiotech_17708	0.002319	0.086	2.39	up
oebiotech_14480	0.006387	0.13	2.39	up
oebiotech_20258	0.011563	0.168	2.39	up
oebiotech_26728	0.015189	0.19	2.39	up
oebiotech_19315	0.000182	0.037	2.4	up
oebiotech_13793	0.000193	0.0372	2.4	up
oebiotech_10418	0.00307	0.0954	2.4	up
oebiotech_06524	0.006661	0.132	2.4	up
oebiotech_04807	0.011216	0.165	2.4	up
A_21_P0004569	0.015896	0.194	2.4	up
oebiotech_11083	0.016951	0.199	2.4	up
oebiotech_02075	0.024314	0.233	2.4	up
oebiotech_19701	0.036256	0.277	2.4	up
oebiotech_24289	0.046174	0.308	2.4	up
A_21_P0013163	0.005048	0.117	2.41	up
oebiotech_10835	0.012669	0.175	2.41	up
oebiotech_11118	0.027122	0.244	2.41	up
oebiotech_20162	1.57E-05	0.017	2.42	up
A_21_P0005252	0.000241	0.0395	2.42	up
oebiotech_18897	0.007153	0.136	2.42	up
oebiotech_12476	0.01203	0.171	2.42	up
oebiotech_24241	0.012472	0.174	2.42	up
A_21_P0011949	0.032728	0.265	2.42	up
A_21_P0005949	0.033142	0.267	2.42	up
A_19_P00802154	0.000303	0.0416	2.43	up
A_19_P00321187	0.002311	0.086	2.43	up
oebiotech_05877	0.002443	0.0879	2.43	up
oebiotech_18641	0.003462	0.1	2.43	up
oebiotech_11006	0.003755	0.103	2.43	up
oebiotech_05577	0.004326	0.111	2.43	up

oebiotech_11279	0.004797	0.115	2.43	up
oebiotech_25565	0.028885	0.251	2.43	up
oebiotech_28007	0.035391	0.274	2.43	up
oebiotech_02027	0.000119	0.0326	2.44	up
A_33_P3311170	0.00128	0.0701	2.44	up
oebiotech_21737	0.004414	0.112	2.44	up
oebiotech_03561	0.006746	0.133	2.44	up
oebiotech_26651	0.007801	0.142	2.44	up
oebiotech_11326	0.020113	0.215	2.44	up
oebiotech_16443	0.008939	0.151	2.45	up
oebiotech_11526	0.019941	0.213	2.45	up
oebiotech_15520	0.034066	0.27	2.45	up
oebiotech_28053	0.00155	0.0755	2.46	up
A_21_P0006671	0.00289	0.0931	2.46	up
oebiotech_11804	0.003913	0.106	2.46	up
oebiotech_19565	0.011478	0.167	2.46	up
A_19_P00321075	0.018331	0.206	2.46	up
A_21_P0003085	0.030048	0.256	2.46	up
oebiotech_25382	0.049154	0.316	2.46	up
oebiotech_11496	1.27E-05	0.0158	2.47	up
A_24_P753476	0.00274	0.0913	2.47	up
oebiotech_26917	0.004853	0.115	2.47	up
oebiotech_10971	0.004919	0.116	2.47	up
oebiotech_12743	0.005613	0.123	2.47	up
oebiotech_04543	0.017023	0.2	2.47	up
oebiotech_08004	0.040371	0.291	2.47	up
oebiotech_05360	0.000639	0.0568	2.49	up
oebiotech_11150	0.002321	0.086	2.49	up
A_21_P0014433	0.018491	0.207	2.49	up
oebiotech_05226	7.8E-06	0.0158	2.5	up
oebiotech_01849	1.03E-05	0.0158	2.5	up
oebiotech_07139	5.69E-05	0.0256	2.5	up
oebiotech_12582	0.000481	0.0507	2.5	up
oebiotech_10912	0.000944	0.0636	2.5	up
oebiotech_07232	0.001851	0.0792	2.5	up
A_21_P0012286	0.003998	0.107	2.5	up
oebiotech_17517	0.007267	0.137	2.5	up
oebiotech_18747	0.000229	0.0392	2.51	up
oebiotech_12933	0.000462	0.0497	2.51	up
oebiotech_12299	0.003385	0.0997	2.51	up
oebiotech_25386	0.023402	0.229	2.51	up
oebiotech_25381	0.035107	0.273	2.51	up
oebiotech_25156	0.000275	0.0403	2.52	up
oebiotech_20279	0.02081	0.218	2.52	up
oebiotech_20200	0.045724	0.306	2.52	up
oebiotech_27684	0.000202	0.0374	2.53	up
oebiotech_19730	0.007465	0.139	2.53	up
oebiotech_24282	0.029193	0.252	2.53	up
oebiotech_28280	0.040536	0.291	2.53	up
oebiotech_11649	0.040583	0.291	2.53	up

A_21_P0009645	0.001648	0.0766	2.54	up
oebiotech_16768	0.003859	0.105	2.54	up
oebiotech_00347	0.008406	0.147	2.54	up
oebiotech_22139	0.011295	0.166	2.54	up
oebiotech_15114	0.049413	0.317	2.54	up
oebiotech_19844	0.00022	0.0385	2.55	up
oebiotech_21327	0.000625	0.0566	2.55	up
oebiotech_21415	0.002838	0.0923	2.55	up
oebiotech_25872	0.003019	0.0945	2.55	up
oebiotech_04534	0.00338	0.0996	2.55	up
oebiotech_16525	0.004102	0.109	2.55	up
oebiotech_06393	0.004124	0.109	2.55	up
oebiotech_27950	0.024152	0.232	2.55	up
oebiotech_11828	0.000415	0.0471	2.56	up
oebiotech_12072	0.00212	0.0838	2.56	up
oebiotech_23087	0.004705	0.114	2.56	up
oebiotech_22561	0.005657	0.123	2.56	up
oebiotech_00382	0.006728	0.133	2.56	up
oebiotech_11730	0.000593	0.0553	2.57	up
oebiotech_15986	0.002275	0.0858	2.57	up
oebiotech_10987	0.002928	0.0934	2.57	up
oebiotech_26916	0.003747	0.103	2.57	up
A_21_P0013078	0.019188	0.21	2.57	up
oebiotech_11148	0.025408	0.238	2.57	up
A_23_P55281	0.000333	0.0435	2.58	up
oebiotech_21535	0.000727	0.0585	2.58	up
oebiotech_14479	0.002383	0.0872	2.58	up
oebiotech_26051	0.010659	0.163	2.58	up
oebiotech_27366	0.002824	0.092	2.59	up
oebiotech_12622	0.00641	0.13	2.59	up
oebiotech_17379	0.000765	0.0586	2.6	up
oebiotech_00064	0.003583	0.102	2.6	up
A_21_P0012978	0.005673	0.123	2.6	up
oebiotech_16119	0.027806	0.247	2.6	up
oebiotech_28359	0.00105	0.066	2.61	up
oebiotech_05487	0.001621	0.0766	2.61	up
A_21_P0004807	0.007343	0.138	2.61	up
A_23_P70968	0.01297	0.177	2.61	up
oebiotech_03241	0.013674	0.181	2.61	up
A_33_P3376454	0.04043	0.291	2.61	up
oebiotech_06977	5.71E-05	0.0256	2.62	up
oebiotech_20104	0.000745	0.0585	2.62	up
oebiotech_27299	0.005214	0.118	2.62	up
A_21_P0001161	0.008597	0.149	2.62	up
oebiotech_19213	0.021664	0.222	2.62	up
oebiotech_11844	0.025361	0.238	2.62	up
oebiotech_17106	0.013733	0.181	2.63	up
A_21_P0012601	0.001655	0.0766	2.64	up
oebiotech_17172	0.003184	0.0971	2.64	up
oebiotech_23094	0.004246	0.11	2.64	up

oebiotech_23359	0.010629	0.162	2.64	up
A_21_P0009823	0.027137	0.245	2.64	up
oebiotech_05392	0.000164	0.0364	2.65	up
oebiotech_12027	0.000867	0.0619	2.65	up
A_21_P0006958	0.015342	0.191	2.65	up
oebiotech_11745	0.023055	0.228	2.65	up
oebiotech_11109	5.81E-05	0.0256	2.66	up
oebiotech_26535	0.001569	0.0757	2.66	up
A_33_P3221859	0.002424	0.0877	2.66	up
A_24_P15621	0.009003	0.151	2.66	up
oebiotech_26965	0.037967	0.283	2.66	up
oebiotech_16642	0.047917	0.313	2.66	up
oebiotech_02555	0.011726	0.169	2.67	up
oebiotech_04129	0.022402	0.225	2.67	up
oebiotech_26394	0.032238	0.264	2.67	up
oebiotech_14063	5.41E-05	0.0253	2.68	up
oebiotech_05375	5.93E-05	0.0257	2.68	up
A_21_P0008572	0.000755	0.0585	2.68	up
oebiotech_27349	0.001455	0.0739	2.68	up
A_21_P0003492	0.001753	0.0779	2.68	up
A_21_P0009886	0.004435	0.112	2.68	up
oebiotech_19281	0.018303	0.206	2.68	up
A_21_P0014129	0.046804	0.31	2.68	up
oebiotech_21700	0.000354	0.0447	2.69	up
oebiotech_07177	0.005912	0.125	2.69	up
oebiotech_10910	0.008333	0.146	2.69	up
oebiotech_15269	0.013242	0.179	2.69	up
oebiotech_19537	0.001978	0.0816	2.7	up
oebiotech_05779	0.009477	0.155	2.7	up
A_21_P0012343	0.015129	0.19	2.7	up
oebiotech_17295	0.032317	0.264	2.7	up
A_21_P0014434	0.000269	0.0403	2.71	up
oebiotech_05329	0.000932	0.0634	2.71	up
oebiotech_21379	0.001518	0.0754	2.71	up
oebiotech_28001	0.006784	0.133	2.71	up
A_33_P3418617	0.011833	0.17	2.71	up
oebiotech_14799	0.015812	0.194	2.71	up
A_21_P0006111	0.044663	0.303	2.71	up
oebiotech_12339	0.003874	0.105	2.72	up
A_33_P3234515	0.01185	0.17	2.72	up
oebiotech_12472	0.030152	0.256	2.72	up
oebiotech_24840	0.000756	0.0585	2.73	up
oebiotech_25522	0.002105	0.0837	2.73	up
oebiotech_27435	0.019742	0.213	2.73	up
oebiotech_26889	0.001783	0.0784	2.74	up
oebiotech_11972	0.011891	0.17	2.74	up
A_32_P128391	0.001843	0.0792	2.75	up
oebiotech_03249	0.002619	0.0895	2.75	up
oebiotech_00551	0.003745	0.103	2.75	up
oebiotech_04147	0.005665	0.123	2.75	up

oebiotech_01489	0.005885	0.125	2.75	up
oebiotech_27379	0.021064	0.219	2.75	up
oebiotech_14108	0.00042	0.0474	2.76	up
A_21_P0009265	0.013279	0.179	2.76	up
A_24_P418044	0.017981	0.205	2.76	up
oebiotech_25383	0.02975	0.255	2.76	up
oebiotech_14592	0.030518	0.257	2.76	up
oebiotech_25521	0.032316	0.264	2.76	up
oebiotech_03362	0.002316	0.086	2.77	up
oebiotech_08227	0.003526	0.101	2.77	up
oebiotech_07913	0.003105	0.0958	2.78	up
oebiotech_12549	0.000194	0.0373	2.79	up
oebiotech_07434	0.001809	0.079	2.79	up
A_19_P00321194	0.013715	0.181	2.79	up
oebiotech_16085	0.000282	0.0407	2.8	up
A_21_P0008526	0.00403	0.108	2.8	up
oebiotech_04131	0.022943	0.228	2.8	up
A_21_P0012185	0.028913	0.251	2.8	up
oebiotech_11889	0.000411	0.047	2.81	up
oebiotech_11468	0.002067	0.0833	2.81	up
A_21_P0001127	0.000494	0.0512	2.82	up
oebiotech_27312	0.009386	0.154	2.82	up
A_24_P453921	0.000565	0.0542	2.83	up
A_21_P0014132	0.001109	0.0668	2.83	up
oebiotech_11865	0.002103	0.0837	2.83	up
oebiotech_03677	0.008784	0.15	2.83	up
A_21_P0013832	0.01264	0.175	2.83	up
oebiotech_14678	0.045637	0.306	2.83	up
oebiotech_03253	2.08E-05	0.0194	2.84	up
oebiotech_26631	0.003754	0.103	2.84	up
oebiotech_20213	0.003721	0.103	2.85	up
oebiotech_20719	0.014706	0.187	2.85	up
oebiotech_21367	0.000122	0.0329	2.86	up
oebiotech_10997	0.000802	0.0596	2.86	up
A_32_P138042	0.001278	0.0701	2.86	up
oebiotech_03784	0.000382	0.0459	2.87	up
oebiotech_12324	0.001402	0.0728	2.87	up
A_21_P0001430	0.004087	0.108	2.88	up
oebiotech_25655	0.005887	0.125	2.88	up
oebiotech_15046	0.003236	0.098	2.89	up
oebiotech_21430	0.005305	0.12	2.89	up
oebiotech_21111	0.00943	0.154	2.89	up
A_21_P0000563	0.022941	0.228	2.89	up
oebiotech_04301	0.000209	0.0378	2.91	up
oebiotech_14575	0.007405	0.138	2.91	up
oebiotech_16338	0.00206	0.0832	2.93	up
oebiotech_06323	0.000012	0.0158	2.94	up
oebiotech_01928	9.18E-05	0.0302	2.95	up
A_21_P0014576	0.000984	0.065	2.95	up
oebiotech_01623	0.010798	0.163	2.95	up

oebiotech_16953	0.03294	0.266	2.96	up
A_33_P3351894	0.000804	0.0596	2.97	up
oebiotech_11967	0.001405	0.0728	2.97	up
A_21_P0000512	0.014755	0.188	2.97	up
oebiotech_21693	0.003393	0.0997	2.98	up
A_24_P339858	0.010492	0.162	2.98	up
A_21_P0012594	0.03012	0.256	2.98	up
oebiotech_20921	0.000124	0.0329	2.99	up
oebiotech_26533	0.002113	0.0838	2.99	up
A_21_P0004481	0.022082	0.224	2.99	up
oebiotech_05503	0.000039	0.0226	3	up
oebiotech_06555	0.0001	0.0307	3	up
oebiotech_01427	0.001205	0.0688	3	up
oebiotech_25851	0.02737	0.245	3	up
oebiotech_06431	1.61E-05	0.017	3.01	up
oebiotech_04553	0.000241	0.0395	3.01	up
oebiotech_16812	0.000578	0.0549	3.01	up
oebiotech_22721	0.029109	0.252	3.01	up
A_21_P0012249	0.03607	0.277	3.01	up
oebiotech_16860	5.86E-05	0.0257	3.02	up
oebiotech_19826	0.011079	0.165	3.02	up
oebiotech_23907	0.012559	0.174	3.02	up
oebiotech_24677	0.019985	0.214	3.02	up
oebiotech_06476	0.027638	0.246	3.02	up
oebiotech_28194	8.27E-05	0.0294	3.03	up
oebiotech_28423	0.000199	0.0374	3.03	up
A_21_P0005963	0.024877	0.236	3.03	up
oebiotech_16901	0.032286	0.264	3.03	up
oebiotech_22977	9.1E-06	0.0158	3.04	up
A_21_P0012326	0.001948	0.081	3.04	up
oebiotech_16962	0.000127	0.033	3.05	up
oebiotech_20789	0.003264	0.0983	3.05	up
A_21_P0014169	0.000004	0.0151	3.06	up
oebiotech_24792	8.06E-05	0.0294	3.06	up
A_21_P0009961	0.004971	0.116	3.06	up
A_21_P0013001	0.005138	0.118	3.06	up
A_23_P25150	0.005603	0.123	3.06	up
oebiotech_11927	0.032447	0.264	3.06	up
oebiotech_24410	0.000547	0.0534	3.07	up
oebiotech_11435	0.001725	0.0777	3.07	up
oebiotech_27952	0.026253	0.241	3.07	up
oebiotech_00280	0.000226	0.0392	3.08	up
A_21_P0004423	0.005766	0.124	3.08	up
oebiotech_16489	0.016587	0.198	3.08	up
oebiotech_01245	0.019771	0.213	3.08	up
oebiotech_14675	0.00546	0.121	3.1	up
oebiotech_27530	4.04E-05	0.0229	3.11	up
oebiotech_20331	0.002989	0.0941	3.11	up
oebiotech_27072	0.015746	0.193	3.11	up
oebiotech_14996	0.00542	0.121	3.12	up

oebiotech_23164	0.010654	0.163	3.12	up
oebiotech_10759	0.012019	0.171	3.12	up
oebiotech_26495	0.017812	0.204	3.13	up
oebiotech_01846	0.038556	0.285	3.13	up
A_21_P0012398	0.000446	0.0488	3.14	up
oebiotech_15768	0.001208	0.0688	3.14	up
oebiotech_02620	0.003411	0.0998	3.14	up
oebiotech_10855	0.00451	0.112	3.17	up
oebiotech_22660	0.0297	0.254	3.17	up
oebiotech_26646	0.014173	0.184	3.18	up
oebiotech_00915	0.023342	0.229	3.18	up
oebiotech_11766	3.14E-05	0.0214	3.19	up
oebiotech_19313	0.002912	0.0932	3.19	up
oebiotech_11010	0.002946	0.0935	3.19	up
A_19_P00805263	0.023107	0.228	3.19	up
oebiotech_28427	0.000283	0.0407	3.2	up
A_21_P0002733	0.000526	0.0523	3.2	up
oebiotech_12034	0.003662	0.103	3.2	up
oebiotech_00155	0.01577	0.193	3.2	up
A_21_P0007209	0.001751	0.0779	3.21	up
oebiotech_12455	0.005931	0.126	3.21	up
oebiotech_00390	0.014136	0.184	3.21	up
oebiotech_26058	3.16E-05	0.0214	3.22	up
oebiotech_04588	0.002567	0.0892	3.22	up
A_21_P0012288	0.002963	0.0936	3.24	up
A_33_P3877344	0.008691	0.149	3.24	up
oebiotech_13685	0.006013	0.126	3.25	up
oebiotech_11245	0.021517	0.221	3.26	up
oebiotech_26945	0.038075	0.283	3.26	up
oebiotech_22610	0.000265	0.0403	3.27	up
oebiotech_18905	0.00086	0.0618	3.28	up
oebiotech_11258	0.003225	0.0979	3.28	up
oebiotech_12067	0.003884	0.106	3.28	up
oebiotech_11228	1.43E-05	0.0165	3.29	up
oebiotech_04474	0.002771	0.0916	3.29	up
oebiotech_07926	0.037795	0.282	3.3	up
A_21_P0011963	0.010312	0.16	3.32	up
A_19_P00321206	0.004976	0.116	3.33	up
oebiotech_14285	0.046784	0.31	3.33	up
oebiotech_16308	0.004843	0.115	3.34	up
oebiotech_11675	0.018196	0.205	3.34	up
A_21_P0010778	0.002126	0.0838	3.35	up
A_19_P00317242	0.006637	0.132	3.35	up
A_21_P0011629	0.006884	0.134	3.35	up
oebiotech_17327	0.000349	0.0444	3.36	up
oebiotech_06516	0.000749	0.0585	3.36	up
oebiotech_26568	0.001749	0.0779	3.37	up
oebiotech_11783	0.00327	0.0983	3.38	up
oebiotech_19365	1.26E-05	0.0158	3.39	up
oebiotech_22829	0.019384	0.211	3.39	up

oebiotech_20773	0.002624	0.0896	3.41	up
A_21_P0009482	0.008812	0.15	3.41	up
A_19_P00322310	0.001731	0.0779	3.43	up
A_21_P0008730	0.0041	0.109	3.43	up
oebiotech_10736	0.004326	0.111	3.44	up
oebiotech_22526	4.19E-05	0.0229	3.46	up
oebiotech_15541	0.000368	0.0453	3.46	up
oebiotech_12258	0.001635	0.0766	3.46	up
oebiotech_16012	0.016044	0.195	3.46	up
oebiotech_16426	0.008458	0.148	3.47	up
A_23_P369485	0.015773	0.193	3.47	up
A_21_P0012069	0.000231	0.0392	3.49	up
A_23_P47941	0.000432	0.0481	3.49	up
oebiotech_13119	0.00088	0.0623	3.49	up
oebiotech_04010	0.014415	0.185	3.49	up
oebiotech_22976	0.000743	0.0585	3.5	up
oebiotech_22836	0.010367	0.161	3.5	up
A_21_P0014122	0.00177	0.0782	3.51	up
oebiotech_00659	0.002062	0.0832	3.51	up
oebiotech_17634	0.007086	0.136	3.51	up
oebiotech_16151	0.000237	0.0393	3.52	up
oebiotech_26632	0.004566	0.113	3.53	up
oebiotech_21395	0.018273	0.206	3.53	up
oebiotech_18459	0.000202	0.0374	3.54	up
oebiotech_13598	0.003364	0.0994	3.54	up
A_21_P0008064	0.000131	0.0334	3.55	up
oebiotech_04581	0.006908	0.134	3.56	up
oebiotech_11747	0.01795	0.205	3.56	up
A_21_P0014344	0.001949	0.081	3.57	up
oebiotech_13700	0.008899	0.151	3.58	up
A_33_P3576853	0.000589	0.0553	3.61	up
A_21_P0005617	0.020228	0.215	3.61	up
A_21_P0011275	8.62E-05	0.0301	3.63	up
oebiotech_19461	0.000752	0.0585	3.63	up
oebiotech_22089	0.000823	0.0603	3.63	up
A_23_P66682	0.014668	0.187	3.63	up
oebiotech_26133	0.020371	0.216	3.65	up
oebiotech_24670	0.004473	0.112	3.66	up
A_21_P0000491	0.002506	0.0886	3.69	up
A_23_P500998	0.002858	0.0925	3.69	up
A_21_P0001429	0.002994	0.0941	3.69	up
A_32_P169179	0.043161	0.299	3.69	up
oebiotech_25850	0.026564	0.242	3.72	up
oebiotech_13559	0.045487	0.305	3.72	up
oebiotech_27311	0.009985	0.158	3.74	up
oebiotech_11535	0.000149	0.0355	3.75	up
oebiotech_22687	0.009326	0.153	3.76	up
A_21_P0012411	0.013023	0.177	3.76	up
oebiotech_25823	0.000554	0.0535	3.77	up
oebiotech_11444	0.015961	0.195	3.77	up

A_21_P0008964	0.020706	0.218	3.77	up
oebiotech_11079	0.003554	0.101	3.81	up
A_23_P373119	1.6E-06	0.0112	3.86	up
oebiotech_07944	0.00031	0.0419	3.87	up
oebiotech_14645	0.000288	0.0409	3.88	up
A_23_P363316	0.002279	0.0858	3.9	up
oebiotech_22804	5.02E-05	0.025	3.93	up
oebiotech_21472	0.000507	0.0519	3.96	up
A_21_P0003199	0.000798	0.0595	3.96	up
oebiotech_22948	0.003208	0.0975	3.96	up
A_33_P3286151	0.004574	0.113	3.97	up
A_21_P0014880	0.01579	0.194	3.97	up
oebiotech_12042	0.001342	0.0715	3.98	up
oebiotech_10906	0.008104	0.145	3.98	up
oebiotech_22903	0.008525	0.148	3.99	up
A_21_P0014763	7.47E-05	0.0285	4	up
oebiotech_02580	0.02546	0.238	4	up
oebiotech_28278	0.002022	0.0826	4.01	up
oebiotech_13118	0.000144	0.0352	4.02	up
oebiotech_25853	0.00895	0.151	4.03	up
oebiotech_04864	0.000363	0.0451	4.04	up
oebiotech_22229	0.001668	0.0766	4.05	up
oebiotech_12846	0.001569	0.0757	4.07	up
oebiotech_12062	0.0025	0.0886	4.07	up
oebiotech_00744	0.0056	0.123	4.07	up
oebiotech_10909	0.002556	0.0891	4.11	up
oebiotech_21701	0.002576	0.0893	4.13	up
A_24_P124558	5.14E-05	0.025	4.14	up
oebiotech_20398	0.001693	0.0772	4.14	up
oebiotech_02481	9.4E-06	0.0158	4.24	up
oebiotech_22935	0.003769	0.104	4.26	up
oebiotech_11864	0.004006	0.107	4.26	up
oebiotech_00976	0.005557	0.122	4.29	up
A_21_P0000597	0.002116	0.0838	4.31	up
A_21_P0011948	0.009816	0.157	4.31	up
A_21_P0012328	8.67E-05	0.0301	4.32	up
oebiotech_22459	0.013451	0.18	4.35	up
A_23_P64808	2.57E-05	0.0204	4.36	up
oebiotech_26907	0.000108	0.0318	4.36	up
A_33_P3286146	0.001505	0.075	4.39	up
oebiotech_15653	0.002157	0.084	4.39	up
oebiotech_11907	0.019504	0.211	4.39	up
A_21_P0005363	0.024228	0.233	4.45	up
oebiotech_19837	0.039351	0.287	4.45	up
A_21_P0014418	0.001152	0.0676	4.46	up
oebiotech_26633	0.003729	0.103	4.46	up
A_21_P0012024	0.000675	0.0575	4.49	up
A_21_P0014104	0.00259	0.0893	4.49	up
A_24_P218805	0.010872	0.164	4.5	up
oebiotech_18352	0.000243	0.0395	4.51	up

oebiotech_21901	0.006775	0.133	4.55	up
oebiotech_20373	4.8E-06	0.0151	4.57	up
oebiotech_11008	0.000114	0.0326	4.58	up
A_21_P0007735	0.000587	0.0552	4.59	up
oebiotech_18394	0.000064	0.0268	4.6	up
oebiotech_00717	0.000168	0.0364	4.61	up
oebiotech_14199	0.000999	0.0651	4.63	up
oebiotech_18408	4.5E-06	0.0151	4.64	up
oebiotech_11487	0.006613	0.132	4.72	up
oebiotech_28173	4.7E-06	0.0151	4.73	up
A_21_P0005092	0.000397	0.0463	4.73	up
oebiotech_01179	0.004091	0.109	4.75	up
oebiotech_15473	0.001117	0.067	4.76	up
oebiotech_11101	0.000668	0.0573	4.79	up
oebiotech_16055	0.000386	0.046	4.81	up
oebiotech_27833	0.003697	0.103	4.83	up
oebiotech_15290	0.0033	0.0988	4.84	up
A_19_P00323040	0.025219	0.237	4.88	up
oebiotech_21360	0.000166	0.0364	4.89	up
A_21_P0013831	0.002287	0.0858	4.91	up
oebiotech_16974	0.00028	0.0407	4.97	up
oebiotech_00731	1.31E-05	0.0158	4.98	up
oebiotech_04146	0.001816	0.079	5.05	up
oebiotech_18371	1.78E-05	0.0179	5.09	up
oebiotech_13195	0.004782	0.115	5.12	up
oebiotech_11829	0.000707	0.0582	5.25	up
A_21_P0005627	0.001846	0.0792	5.25	up
oebiotech_03747	0.012797	0.176	5.3	up
oebiotech_26906	2.52E-05	0.0203	5.31	up
oebiotech_11490	0.002285	0.0858	5.4	up
oebiotech_26857	9.71E-05	0.0302	5.42	up
oebiotech_26392	0.000914	0.063	5.59	up
A_21_P0004444	0.000912	0.063	5.64	up
A_24_P77904	0.001552	0.0755	5.64	up
A_21_P0013869	0.00159	0.0762	5.65	up
oebiotech_18435	7.9E-06	0.0158	5.67	up
A_33_P3253304	1.55E-05	0.017	5.75	up
oebiotech_11684	0.010295	0.16	5.9	up
oebiotech_15502	0.000166	0.0364	6.03	up
oebiotech_16172	1.19E-05	0.0158	6.13	up
oebiotech_18544	1.47E-05	0.0167	6.17	up
A_23_P381368	0.000308	0.0417	6.17	up
A_23_P110276	0.010583	0.162	6.22	up
A_21_P0014553	1.33E-05	0.0158	6.3	up
oebiotech_26449	5.57E-05	0.0256	6.39	up
oebiotech_12651	0.000565	0.0542	6.71	up
A_21_P0011276	8.8E-06	0.0158	6.73	up
A_33_P3587376	0.00491	0.116	6.75	up
oebiotech_07770	1.32E-05	0.0158	6.78	up
oebiotech_18384	1.03E-05	0.0158	6.86	up

oebiotech_03505	0.000635	0.0568	6.92	up
oebiotech_15718	0.010892	0.164	7.04	up
oebiotech_26856	2E-07	0.0112	7.15	up
A_21_P0004445	0.000356	0.0447	7.15	up
oebiotech_18385	0.000256	0.0403	7.18	up
A_21_P0014938	0.000391	0.0461	7.19	up
oebiotech_11481	0.030549	0.257	7.24	up
oebiotech_15402	0.002607	0.0893	7.5	up
oebiotech_05542	3.15E-05	0.0214	7.51	up
oebiotech_16849	9.7E-06	0.0158	7.53	up
oebiotech_17024	3E-07	0.0112	7.74	up
oebiotech_03518	0.009401	0.154	7.75	up
oebiotech_14690	2.27E-05	0.0195	7.77	up
A_21_P0013667	0.002932	0.0935	7.78	up
oebiotech_10772	7.34E-05	0.0284	7.8	up
oebiotech_11399	0.000719	0.0585	7.81	up
A_21_P0000492	0.002214	0.085	7.85	up
A_21_P0010428	0.010305	0.16	7.87	up
A_21_P0000509	0.002912	0.0932	7.89	up
oebiotech_05506	8.28E-05	0.0294	7.91	up
oebiotech_10897	0.007879	0.143	7.92	up
A_21_P0000508	0.004053	0.108	7.95	up
A_21_P0000511	0.002941	0.0935	8.01	up
oebiotech_02557	5.9E-06	0.0158	8.12	up
A_21_P0004015	6.4E-06	0.0158	8.32	up
oebiotech_04359	0.008338	0.146	8.35	up
A_21_P0000507	0.004648	0.113	8.5	up
oebiotech_26905	1.03E-05	0.0158	8.87	up
oebiotech_04998	3.41E-05	0.0219	8.99	up
A_19_P00318487	0.00154	0.0754	9	up
A_21_P0014762	0.000093	0.0302	9.62	up
A_21_P0014928	0.000175	0.0366	9.72	up
A_21_P0003487	0.01206	0.171	10.19	up
oebiotech_26303	0.004276	0.11	10.94	up
oebiotech_16532	1.1E-06	0.0112	10.96	up
oebiotech_26068	1.4E-06	0.0112	10.96	up
oebiotech_11447	0.000631	0.0568	11.21	up
oebiotech_12063	0.000519	0.0521	11.25	up
oebiotech_14949	0.000191	0.0372	11.32	up
A_23_P143029	0.000285	0.0408	11.82	up
oebiotech_10778	0.000617	0.0564	12.02	up
oebiotech_26348	0.006568	0.132	12.02	up
oebiotech_18404	1.6E-06	0.0112	12.61	up
oebiotech_20436	0.010771	0.163	12.82	up
oebiotech_16630	3.91E-05	0.0226	13.26	up
A_32_P62963	0.000706	0.0581	14.01	up
oebiotech_19125	0.001594	0.0762	14.56	up
oebiotech_15267	0.000174	0.0364	14.84	up
oebiotech_20435	0.000201	0.0374	15.72	up
oebiotech_12418	9.47E-05	0.0302	15.92	up

oebiotech_26302	0.000251	0.0403	16.74	up
A_21_P0014892	7.7E-06	0.0158	17.82	up
A_21_P0014753	0.000539	0.0528	18.06	up
A_33_P3857239	0.000185	0.0372	19.63	up
oebiotech_12606	0.002546	0.0891	19.64	up
A_21_P0011578	0.000202	0.0374	20.89	up
oebiotech_04912	0.000314	0.042	25.27	up
oebiotech_11410	6.01E-05	0.0259	79.17	up
oebiotech_16306	1.5E-06	0.0112	121.79	up
oebiotech_12605	8.9E-06	0.0158	236.7	up
