# Supplementary material for: Integrated Analysis of Long Noncoding RNA and mRNA Expression Profile in Advanced Laryngeal Squamous Cell Carcinoma
Source: PLoS One. 2016 Dec 29;11(12):e0169232. doi: 10.1371/journal.pone.0169232 (PMC5199101; doi:10.1371/journal.pone.0169232)
Supplement: S4 Table — (PDF) [file pone.0169232.s004.pdf]

ProbeSet	p-value	FDR	Fold change(C/N)	style	TargetID
A_21_P0000003	0.0046135	0.113	0.0098	down	PRR4
A_23_P115202	0.0009123	0.063	0.011	down	CRNN
A_23_P17134	0.0031089	0.0958	0.012	down	MAL
A_32_P173662	0.0000005	0.0112	0.015	down	CRISP2
A_23_P45751	0.00151	0.075	0.017	down	CLCA4
A_23_P41145	0.0000477	0.0245	0.018	down	FAM3D
A_23_P2674	0.0012884	0.0703	0.018	down	KRT4
A_33_P3381821	0.0072682	0.137	0.022	down	CAPN14
A_33_P3318097	0.0021752	0.0843	0.023	down	TMPRSS11B
A_24_P228149	0.0047096	0.114	0.027	down	KRT13
A_23_P62709	0.0349266	0.273	0.033	down	SPRR3
A_23_P57118	0.0206273	0.217	0.036	down	TGM3
A_24_P208825	0.0003034	0.0416	0.038	down	MUC4
A_33_P3233040	0.000671	0.0574	0.038	down	SERPINB11
A_23_P99076	0.0119436	0.171	0.038	down	PRH2
A_23_P356494	0.0178764	0.204	0.039	down	SPINK5
A_23_P76291	0.0091876	0.152	0.04	down	PRR4
A_33_P3216570	0.000961	0.0643	0.042	down	MUC5AC
A_33_P3405474	0.0034204	0.0999	0.044	down	ENST00000450445
A_33_P3358208	0.006669	0.132	0.045	down	PADI1
A_33_P3226377	0.0086293	0.149	0.045	down	PRH2
A_23_P71379	0.000305	0.0417	0.047	down	PSCA
A_33_P3265783	0.0120976	0.171	0.049	down	STATH
A_33_P3391126	0.0223982	0.225	0.049	down	SPINK5
A_32_P161033	0.001337	0.0714	0.049	down	ENST00000433980
A_23_P4387	0.0014134	0.073	0.051	down	KRT24
A_23_P60990	0.0109335	0.164	0.052	down	C2orf54
A_23_P118203	0.00621	0.128	0.059	down	ZG16B
A_23_P94186	0.0115065	0.167	0.059	down	LYPD2
A_33_P3343316	0.0000161	0.017	0.06	down	SH3BGRL2
A_24_P209171	0.0001851	0.0372	0.06	down	SH3BGRL2
A_33_P3240693	0.0000149	0.0167	0.062	down	THSD4
A_23_P153301	0.0020479	0.083	0.064	down	CEACAM5
A_23_P129144	0.0028869	0.0931	0.065	down	MYZAP
A_23_P114713	0.0001926	0.0372	0.066	down	CYP4B1
A_33_P3275035	0.004567	0.113	0.066	down	MUC21
A_23_P94103	0.0001037	0.0307	0.069	down	SCARA5
A_23_P348028	0.0110559	0.165	0.069	down	IL36A
A_33_P3216150	0.0255091	0.238	0.074	down	PRB4
A_33_P3217845	0.0070735	0.135	0.075	down	TMPRSS11A
A_23_P106806	0.0286173	0.25	0.075	down	PRSS27
A_32_P191786	0.0028478	0.0923	0.076	down	ANKRD24
A_33_P3417281	0.0003624	0.045	0.078	down	MUC4

A_24_P109101	0.0066541	0.132	0.079	down	TMPRSS11A
A_33_P3308862	0.0058117	0.124	0.08	down	FAM22A
A_32_P167076	0.0122226	0.172	0.082	down	CAPN14
A_33_P3332625	0.0242959	0.233	0.082	down	PRB1
A_23_P26154	0.0058747	0.125	0.083	down	PLIN1
A_24_P183128	0.0011964	0.0687	0.085	down	PLAC8
A_23_P119562	0.0002613	0.0403	0.088	down	CFD
A_23_P8702	0.0325054	0.264	0.089	down	PIP
A_23_P420209	0.000492	0.0512	0.09	down	GCNT3
A_23_P202448	0.0010567	0.0662	0.091	down	CXCL12
A_23_P360316	0.0013776	0.0724	0.092	down	FUT3
A_24_P734953	0.0011548	0.0676	0.095	down	TRNP1
A_24_P307135	0.0010664	0.0663	0.096	down	TNXB
A_32_P204376	0.0024347	0.0878	0.096	down	ANKRD20A2
A_23_P369237	0.0114007	0.167	0.096	down	ADIPOQ
A_33_P3330911	0.000298	0.0414	0.097	down	BCAS1
A_33_P3423874	0.0081097	0.145	0.097	down	MYZAP
A_23_P108157	0.0000114	0.0158	0.098	down	TJP3
A_23_P84860	0.0006877	0.0579	0.098	down	FAM107A
A_33_P3364571	0.0011598	0.0676	0.098	down	TNXB
A_24_P239177	0.0002868	0.0408	0.1	down	MUC4
A_33_P3222228	0.0003126	0.0419	0.1	down	FUT6
A_23_P23783	0.0006267	0.0567	0.1	down	MYOC
A_23_P71300	0.0007224	0.0585	0.1	down	CCDC25
A_23_P118615	0.0022301	0.0852	0.1	down	ABCA8
A_23_P382811	0.0027712	0.0916	0.1	down	SNX31
A_23_P52480	0.0029261	0.0934	0.1	down	CYP2C18
A_23_P254507	0.0490702	0.316	0.1	down	HOPX
A_23_P121795	0.000031	0.0214	0.11	down	SORBS2
A_23_P80278	0.0016862	0.0771	0.11	down	SFI1
A_23_P130411	0.0018513	0.0792	0.11	down	SERPINB11
A_33_P3326075	0.0023687	0.0869	0.11	down	CYP2C19
A_23_P29394	0.0026817	0.0905	0.11	down	ATP13A4
A_33_P3254844	0.0032649	0.0983	0.11	down	CEACAM7
A_32_P83098	0.0064812	0.131	0.11	down	SCNN1B
A_23_P430948	0.0072555	0.137	0.11	down	ATP13A4
A_33_P3247057	0.0087906	0.15	0.11	down	C17orf109
A_24_P52887	0.0182303	0.206	0.11	down	ENDOU
A_33_P3408232	0.0261498	0.241	0.11	down	MUC22
A_33_P3268726	0.0086287	0.149	0.11	down	ENST00000507038
A_23_P98910	0.0009108	0.063	0.12	down	LRMP
A_23_P152906	0.0013727	0.0723	0.12	down	ALOX12
A_33_P3395688	0.0014354	0.0736	0.12	down	MYZAP
A_23_P23611	0.0018853	0.0799	0.12	down	AMY1C

A_33_P3340782	0.0069522	0.134	0.12	down	SPINK5
A_33_P3318668	0.0096546	0.156	0.12	down	C9orf169
A_33_P3363637	0.009821	0.157	0.12	down	BLNK
A_23_P103617	0.0098842	0.157	0.12	down	ANXA9
A_33_P3403474	0.024391	0.234	0.12	down	TMPRSS11BNL
A_23_P100583	0.0291326	0.252	0.12	down	LPO
A_33_P3395848	0.0322186	0.264	0.12	down	VSIG10L
A_23_P50269	0.0004297	0.0481	0.13	down	CXCL17
A_33_P3365193	0.0007857	0.0592	0.13	down	AMY1C
A_23_P166269	0.0026035	0.0893	0.13	down	FAM3B
A_23_P82000	0.0033152	0.099	0.13	down	TEAD3
A_33_P3301331	0.0050422	0.117	0.13	down	CEACAM3
A_23_P24332	0.0067676	0.133	0.13	down	MUC15
A_23_P130515	0.0073379	0.138	0.13	down	CEACAM3
A_33_P3232504	0.0168514	0.199	0.13	down	C9orf169
A_33_P3213086	0.0004506	0.0491	0.13	down	ENST00000442192
A_24_P140475	0.0000207	0.0194	0.14	down	SORBS2
A_24_P301837	0.0002576	0.0403	0.14	down	HRH3
A_23_P127978	0.0002623	0.0403	0.14	down	B3GNT6
A_23_P86021	0.00069	0.0579	0.14	down	SELENBP1
A_32_P70818	0.00084	0.0607	0.14	down	PAX9
A_23_P365218	0.0009248	0.0632	0.14	down	GPR110
A_23_P17420	0.0011036	0.0668	0.14	down	BCAS1
A_23_P87982	0.0022857	0.0858	0.14	down	ATP12A
A_24_P231104	0.0031436	0.0964	0.14	down	LEPR
A_23_P26511	0.0044386	0.112	0.14	down	GDPD3
A_23_P169437	0.0072467	0.137	0.14	down	LCN2
A_33_P3381378	0.0096324	0.156	0.14	down	PAX1
A_24_P293530	0.0113497	0.166	0.14	down	CYP4X1
A_23_P112482	0.0182575	0.206	0.14	down	AQP3
A_23_P1682	0.0289621	0.251	0.14	down	TMEM45B
A_23_P129835	0.0446728	0.303	0.14	down	PPP1R1B
A_33_P3347241	0.0006591	0.057	0.14	down	ENST00000420598
A_33_P3389599	0.0009002	0.0627	0.14	down	ENST00000442192
A_33_P3268507	0.0015805	0.0761	0.15	down	CEACAM1
A_23_P157136	0.0017193	0.0777	0.15	down	SCIN
A_33_P3248203	0.0020971	0.0836	0.15	down	FUT5
A_23_P170534	0.0033299	0.099	0.15	down	FUT7
A_23_P119634	0.0099016	0.158	0.15	down	UPK1A
A_23_P76488	0.0195621	0.211	0.15	down	EMP1
A_33_P3209591	0.0285061	0.249	0.15	down	AQP3
A_23_P166848	0.044225	0.302	0.15	down	LTF
A_23_P362694	0.019334	0.21	0.16	down	C4orf7
A_23_P311640	0.0000291	0.0212	0.16	down	AGFG2

A_23_P214267	0.0001545	0.0355	0.16	down	GPR110
A_33_P3725227	0.0002579	0.0403	0.16	down	COBL
A_23_P213857	0.0007603	0.0586	0.16	down	C7
A_33_P3275702	0.0009262	0.0632	0.16	down	FMO2
A_23_P502957	0.0012105	0.0688	0.16	down	CDH26
A_24_P8371	0.0014385	0.0736	0.16	down	SPNS2
A_23_P12767	0.0014399	0.0736	0.16	down	CYP2C9
A_23_P8801	0.002843	0.0923	0.16	down	CYP3A5
A_33_P3214825	0.0039218	0.106	0.16	down	LOC100652774
A_23_P13083	0.0075796	0.14	0.16	down	BARX2
A_24_P46130	0.008255	0.146	0.16	down	ACPP
A_32_P111639	0.0156635	0.193	0.16	down	CHST9
A_23_P218442	0.0305185	0.257	0.16	down	CEACAM6
A_23_P8253	0.0464264	0.309	0.16	down	RAET1E
A_24_P778906	0.0005768	0.0549	0.16	down	ENST00000435913
A_23_P419760	0.000923	0.0632	0.17	down	CRISP3
A_33_P3342305	0.0012833	0.0702	0.17	down	ABCA8
A_33_P3279590	0.0019309	0.0807	0.17	down	OGN
A_23_P82990	0.003905	0.106	0.17	down	OGN
A_33_P3273552	0.0131796	0.179	0.17	down	KRT83
A_23_P106906	0.0136615	0.181	0.17	down	PPL
A_24_P372012	0.0000248	0.0203	0.18	down	ICA1
A_23_P328545	0.0000853	0.0299	0.18	down	GABRP
A_23_P23839	0.0002802	0.0407	0.18	down	LGR6
A_24_P658584	0.0012259	0.0692	0.18	down	SASH1
A_23_P8142	0.0025085	0.0886	0.18	down	CLPS
A_23_P105651	0.002561	0.0891	0.18	down	PDZRN4
A_23_P133474	0.0039023	0.106	0.18	down	GPX3
A_33_P3400763	0.0092654	0.153	0.18	down	PLIN4
A_23_P258887	0.0135003	0.18	0.18	down	ALDH1L1
A_33_P3422777	0.0312411	0.26	0.18	down	PRH2
A_24_P14464	0.0448328	0.304	0.18	down	WFDC2
A_24_P239176	0.000782	0.0592	0.19	down	MUC4
A_24_P291658	0.0012559	0.0698	0.19	down	ADH1A
A_23_P126706	0.0015516	0.0755	0.19	down	ANGPTL1
A_33_P3369371	0.0029529	0.0935	0.19	down	GPX3
A_24_P64344	0.0048786	0.115	0.19	down	BLNK
A_23_P319783	0.006561	0.132	0.19	down	SPAG17
A_23_P218369	0.0101072	0.159	0.19	down	CCL14
A_33_P3398597	0.0153475	0.191	0.19	down	EPS8L1
A_23_P93141	0.0459857	0.307	0.19	down	GSTA5
A_21_P0012222	0.0016151	0.0765	0.19	down	C21orf81
A_23_P61042	0.0297109	0.254	0.19	down	ENST00000390539
A_23_P164436	0.0001196	0.0326	0.2	down	ASPA

A_23_P36018	0.0003205	0.0424	0.2	down	VSIG2
A_33_P3410806	0.000677	0.0577	0.2	down	CLDN10
A_33_P3324333	0.0007687	0.0588	0.2	down	ANKRD20A5P
A_32_P66222	0.001591	0.0762	0.2	down	ANKRD20A2
A_23_P87007	0.0016912	0.0772	0.2	down	HTR3B
A_23_P163306	0.0029122	0.0932	0.2	down	CGNL1
A_23_P392384	0.0104219	0.161	0.2	down	AIF1L
A_23_P103765	0.0202864	0.215	0.2	down	FCER1A
A_33_P3384795	0.0010768	0.0667	0.2	down	AK093443
A_33_P3213089	0.001107	0.0668	0.2	down	AK058117
A_33_P3384617	0.0067153	0.133	0.2	down	BX114012
A_23_P132515	0.0002699	0.0403	0.21	down	SIDT1
A_23_P162579	0.0002734	0.0403	0.21	down	HSPB8
A_23_P203540	0.0019172	0.0805	0.21	down	EHF
A_32_P114284	0.0024052	0.0875	0.21	down	IKZF2
A_33_P3303449	0.0042122	0.11	0.21	down	LEPR
A_33_P3378665	0.0042855	0.11	0.21	down	TC2N
A_33_P3315268	0.004482	0.112	0.21	down	KRT78
A_23_P164057	0.0100832	0.159	0.21	down	MFAP4
A_23_P214330	0.0218536	0.223	0.21	down	SERPINB1
A_33_P3302577	0.0444881	0.303	0.21	down	GPR133
A_23_P30098	0.0000232	0.0195	0.22	down	ADH4
A_24_P40626	0.0006897	0.0579	0.22	down	GREM2
A_33_P3420266	0.0009576	0.0642	0.22	down	ANKRD20A2
A_32_P159651	0.0009866	0.065	0.22	down	KAT2B
A_33_P3209279	0.0014742	0.0744	0.22	down	SASH1
A_23_P215913	0.0042472	0.11	0.22	down	CLU
A_24_P397386	0.0064175	0.13	0.22	down	LIFR
A_24_P190472	0.0075094	0.14	0.22	down	SLPI
A_33_P3368991	0.0104232	0.161	0.22	down	MPZ
A_24_P942969	0.0142303	0.184	0.22	down	FUT2
A_23_P351148	0.022592	0.226	0.22	down	SH2D1B
A_23_P42811	0.0317202	0.262	0.22	down	AGR3
A_33_P3276520	0.000203	0.0375	0.22	down	AK123704
A_33_P3406796	0.0230121	0.228	0.22	down	ENST00000511256
A_24_P106502	0.0000113	0.0158	0.23	down	PRICKLE4
A_23_P171074	0.0001021	0.0307	0.23	down	ITM2A
A_23_P415652	0.0003864	0.046	0.23	down	GALNT12
A_24_P120907	0.0007956	0.0595	0.23	down	PGM5
A_23_P54968	0.0010777	0.0667	0.23	down	ST6GALNAC1
A_24_P415150	0.0025005	0.0886	0.23	down	GNA14
A_33_P3369844	0.0026378	0.0898	0.23	down	CD24
A_33_P3260026	0.004151	0.109	0.23	down	AIF1L
A_32_P131031	0.0062507	0.129	0.23	down	MACC1

A_32_P183970	0.0071039	0.136	0.23	down	C15orf62
A_33_P3252781	0.0077305	0.142	0.23	down	PLAC9
A_23_P381714	0.0091874	0.152	0.23	down	CA13
A_23_P116037	0.0098447	0.157	0.23	down	TM7SF2
A_23_P394836	0.0110229	0.165	0.23	down	INF2
A_24_P183994	0.0143004	0.185	0.23	down	RASEF
A_24_P398147	0.0349277	0.273	0.23	down	NEBL
A_33_P3359771	0.0402819	0.29	0.23	down	ATP1A2
A_23_P128323	0.0443102	0.303	0.23	down	SCNN1A
A_24_P168925	0.0000533	0.0253	0.24	down	CHRDL1
A_23_P41217	0.0001035	0.0307	0.24	down	CD200R1
A_23_P159893	0.000618	0.0564	0.24	down	CHRDL1
A_24_P106624	0.0009157	0.063	0.24	down	MEOX2
A_23_P202520	0.0009571	0.0642	0.24	down	ABLIM1
A_23_P157914	0.001095	0.0668	0.24	down	MAMDC2
A_23_P431505	0.0013638	0.0722	0.24	down	XKR6
A_23_P137856	0.0021216	0.0838	0.24	down	MUC1
A_24_P123408	0.0036578	0.103	0.24	down	ABLIM3
A_32_P229618	0.0041474	0.109	0.24	down	DLG2
A_23_P356004	0.0065014	0.131	0.24	down	KCNIP3
A_24_P243749	0.0113085	0.166	0.24	down	PDK4
A_23_P216361	0.0148924	0.188	0.24	down	COL14A1
A_23_P118894	0.0150295	0.189	0.24	down	PRR15L
A_24_P270033	0.0151938	0.19	0.24	down	MPZL3
A_23_P67661	0.0237411	0.231	0.24	down	COX7A1
A_19_P00800061	0.0068379	0.133	0.24	down	ENST00000435913
A_24_P56363	0.0000331	0.0218	0.25	down	CAB39L
A_23_P318284	0.0000454	0.0239	0.25	down	GPD1L
A_23_P66767	0.0004753	0.0502	0.25	down	GGT6
A_33_P3330952	0.0024864	0.0885	0.25	down	ATP8A1
A_33_P3393170	0.0025347	0.0889	0.25	down	CAPN5
A_33_P3842551	0.0028078	0.0918	0.25	down	IKZF2
A_23_P252758	0.0043751	0.111	0.25	down	KEL
A_33_P3358735	0.0044062	0.112	0.25	down	PCSK5
A_24_P228302	0.0097286	0.157	0.25	down	CEACAM7
A_23_P213137	0.0134834	0.18	0.25	down	LNX1
A_23_P10121	0.0307192	0.258	0.25	down	SFRP1
A_23_P102919	0.0317432	0.262	0.25	down	B3GALT5
A_23_P36187	0.0336724	0.269	0.25	down	SYT8
A_23_P307844	0.038148	0.283	0.25	down	PHYHD1
A_23_P416965	0.0000748	0.0285	0.26	down	FAM149A
A_23_P13364	0.0000821	0.0294	0.26	down	NUCB2
A_23_P325690	0.0001796	0.0369	0.26	down	ANKRD35
A_24_P860797	0.0007532	0.0585	0.26	down	PAIP2B

A_33_P3328485	0.0009427	0.0636	0.26	down	IKZF2
A_24_P453497	0.0018509	0.0792	0.26	down	RBM20
A_33_P3411980	0.0034445	0.1	0.26	down	ABI3BP
A_24_P411749	0.0037094	0.103	0.26	down	GPR126
A_23_P415021	0.0050025	0.116	0.26	down	METTL7A
A_23_P254741	0.0053457	0.12	0.26	down	SOD3
A_24_P156501	0.0053888	0.121	0.26	down	EBF1
A_23_P216489	0.0070189	0.135	0.26	down	GNE
A_24_P363583	0.0096164	0.156	0.26	down	AGFG2
A_24_P394940	0.0100465	0.159	0.26	down	CYP2E1
A_23_P66599	0.0176725	0.203	0.26	down	VPS25
A_23_P311901	0.0242219	0.233	0.26	down	ATP10B
A_33_P3402313	0.0286533	0.25	0.26	down	SLC9A4
A_21_P0013571	0.0010125	0.0655	0.26	down	AQP7
A_33_P3277447	0.0000379	0.0226	0.27	down	SLC26A2
A_33_P3251562	0.0015325	0.0754	0.27	down	RSPO1
A_24_P272313	0.0024515	0.0879	0.27	down	C2orf55
A_23_P90944	0.0025571	0.0891	0.27	down	SCN7A
A_24_P15325	0.0025835	0.0893	0.27	down	XKR6
A_23_P8834	0.0027194	0.091	0.27	down	EPHX2
A_23_P103486	0.0035546	0.101	0.27	down	CYP2J2
A_23_P161135	0.0054655	0.121	0.27	down	LEPR
A_23_P78980	0.0056134	0.123	0.27	down	B3GNT3
A_33_P3220698	0.0084554	0.148	0.27	down	EPS8L1
A_24_P173823	0.0142241	0.184	0.27	down	PBX1
A_24_P407259	0.0216933	0.222	0.27	down	SOX21
A_23_P71946	0.0337534	0.269	0.27	down	BSPRY
A_23_P359870	0.0014568	0.074	0.27	down	ENST00000400102
A_21_P0013658	0.0021818	0.0843	0.27	down	PGM5
A_23_P150741	0.00072	0.0585	0.28	down	C2CD3
A_32_P43717	0.0010398	0.066	0.28	down	FAM189A2
A_33_P3335590	0.0079588	0.143	0.28	down	CCDC64B
A_23_P415706	0.010497	0.162	0.28	down	GPR133
A_33_P3367361	0.0133417	0.179	0.28	down	FAM107B
A_24_P308029	0.0174751	0.202	0.28	down	HSPB6
A_33_P3318449	0.0275087	0.246	0.28	down	RNF222
A_23_P69537	0.0297513	0.255	0.28	down	NMU
A_23_P31945	0.030003	0.256	0.28	down	IL33
A_33_P3266550	0.0331779	0.267	0.28	down	ABO
A_23_P78248	0.048784	0.316	0.28	down	KRT23
A_21_P0000084	0.0012645	0.0698	0.28	down	SLC25A21
A_24_P691826	0.0072814	0.138	0.28	down	THC2682885
A_23_P157766	0.0005771	0.0549	0.29	down	C9orf68
A_24_P910733	0.0005853	0.0552	0.29	down	CCDC50

A_33_P3383233	0.0018719	0.0796	0.29	down	NDRG2
A_33_P3348924	0.0019136	0.0805	0.29	down	LOC100128348
A_23_P160004	0.0026611	0.0902	0.29	down	UTY
A_23_P103971	0.0037624	0.103	0.29	down	CYP4Z1
A_23_P147388	0.0041281	0.109	0.29	down	KIF13B
A_32_P358887	0.0048711	0.115	0.29	down	SLC4A4
A_33_P3416331	0.0089764	0.151	0.29	down	SLC10A5
A_23_P411851	0.0111908	0.165	0.29	down	C14orf49
A_32_P197561	0.0119184	0.17	0.29	down	EBF1
A_32_P231568	0.0191497	0.21	0.29	down	RASEF
A_24_P37589	0.019745	0.213	0.29	down	ACPP
A_33_P3234882	0.0202885	0.215	0.29	down	PRELP
A_23_P96383	0.0428545	0.299	0.29	down	SRPX
A_23_P94501	0.0451445	0.305	0.29	down	ANXA1
A_21_P0010771	0.0007478	0.0585	0.29	down	ANKRD20A2
A_21_P0000128	0.0089299	0.151	0.29	down	LOC100129480
A_23_P72068	0.000011	0.0158	0.3	down	GMDS
A_23_P215419	0.000094	0.0302	0.3	down	ICA1
A_24_P343621	0.0002119	0.0379	0.3	down	ECHDC3
A_33_P3254708	0.0002347	0.0392	0.3	down	ARHGAP40
A_33_P3379492	0.0003856	0.046	0.3	down	LOC100131581
A_23_P212508	0.0012083	0.0688	0.3	down	TF
A_23_P64372	0.0016445	0.0766	0.3	down	TCN1
A_33_P3389342	0.0016626	0.0766	0.3	down	ARID5A
A_33_P3388453	0.0017771	0.0784	0.3	down	NANOS2
A_24_P71904	0.0021898	0.0845	0.3	down	HPGD
A_24_P914513	0.0023716	0.0869	0.3	down	BCKDHB
A_23_P152791	0.0036708	0.103	0.3	down	SLC16A6
A_33_P3281795	0.0055341	0.122	0.3	down	MGLL
A_33_P3251522	0.0062685	0.129	0.3	down	AQPEP
A_23_P500130	0.0076734	0.141	0.3	down	KANK1
A_23_P35414	0.0082039	0.146	0.3	down	PPP1R3C
A_23_P120281	0.0112024	0.165	0.3	down	EDAR
A_23_P92107	0.0185342	0.207	0.3	down	SLC15A2
A_33_P3336632	0.0255458	0.238	0.3	down	RN28S1
A_33_P3238941	0.0343776	0.271	0.3	down	ANKRD18A
A_23_P36531	0.0374326	0.281	0.3	down	TSPAN8
A_33_P3289121	0.0468005	0.31	0.3	down	C2orf40
A_24_P312325	0.0000498	0.025	0.3	down	ENST00000500944
A_33_P3402611	0.0022157	0.085	0.3	down	ENST00000417703
A_33_P3305763	0.0039106	0.106	0.3	down	ENST00000531549
A_21_P0013996	0.0041171	0.109	0.3	down	LOC100509121
A_21_P0012120	0.0312885	0.26	0.3	down	ARHGAP40
A_23_P217528	0.0008669	0.0619	0.31	down	KLF8

A_33_P3332215	0.0017566	0.0779	0.31	down	MUC1
A_23_P215111	0.0025085	0.0886	0.31	down	ATP6V0A4
A_24_P357465	0.0031788	0.0971	0.31	down	TP53INP2
A_32_P224522	0.0045969	0.113	0.31	down	SLC25A23
A_33_P3249746	0.0060098	0.126	0.31	down	CYP3A5
A_24_P372625	0.009668	0.156	0.31	down	RNF141
A_23_P218858	0.0131687	0.179	0.31	down	ABI3BP
A_23_P201808	0.0135469	0.18	0.31	down	PPAP2B
A_23_P122508	0.0154158	0.191	0.31	down	DPCR1
A_33_P3236703	0.0154277	0.191	0.31	down	ANKRD18B
A_33_P3263955	0.0176961	0.203	0.31	down	LCN10
A_23_P897	0.020225	0.215	0.31	down	C1orf116
A_23_P56978	0.0230623	0.228	0.31	down	PTK6
A_33_P3287967	0.0252516	0.237	0.31	down	ANK2
A_23_P169978	0.0347804	0.272	0.31	down	ZNF608
A_24_P317907	0.0391281	0.287	0.31	down	SORBS1
A_23_P56559	0.0396391	0.288	0.31	down	DHRS9
A_32_P218355	0.0410214	0.293	0.31	down	C6orf132
A_32_P167471	0.048362	0.314	0.31	down	CLMN
A_23_P156708	0.0002309	0.0392	0.32	down	TNXB
A_23_P61426	0.0006968	0.0579	0.32	down	MSRA
A_33_P3355281	0.0008283	0.0605	0.32	down	MIOX
A_23_P54681	0.0021291	0.0838	0.32	down	TOX3
A_33_P3389291	0.0022745	0.0858	0.32	down	TEX9
A_23_P7402	0.0029181	0.0933	0.32	down	PDZD2
A_23_P422831	0.0085118	0.148	0.32	down	FAM189A2
A_23_P382065	0.0089951	0.151	0.32	down	EMCN
A_33_P3400578	0.0125154	0.174	0.32	down	HLF
A_23_P37892	0.0209789	0.219	0.32	down	GPT2
A_33_P3370019	0.0120808	0.171	0.32	down	ENST00000390556
A_33_P3212092	0.0002534	0.0403	0.33	down	PDCD4
A_33_P3319625	0.0002956	0.0414	0.33	down	NFIA
A_33_P3381338	0.0010346	0.0659	0.33	down	TNXB
A_23_P53126	0.0011518	0.0676	0.33	down	LMO2
A_23_P250735	0.001368	0.0722	0.33	down	CBX7
A_23_P6980	0.0014061	0.0728	0.33	down	ZNF717
A_23_P424582	0.00159	0.0762	0.33	down	EGFL8
A_24_P268729	0.0035606	0.101	0.33	down	HEMK1
A_23_P91943	0.0046695	0.114	0.33	down	IL12A
A_33_P3420053	0.0046731	0.114	0.33	down	ZNF415
A_23_P350617	0.007164	0.136	0.33	down	KLB
A_33_P3244274	0.019116	0.21	0.33	down	RNF208
A_33_P3278755	0.0191979	0.21	0.33	down	SULT6B1
A_23_P101246	0.0192062	0.21	0.33	down	VSIG10L

A_23_P212241	0.0207949	0.218	0.33	down	CHL1
A_23_P124514	0.0221174	0.224	0.33	down	DLG2
A_23_P58266	0.0270807	0.244	0.33	down	S100P
A_33_P3301709	0.0408589	0.292	0.33	down	GNG4
A_23_P37127	0.0419334	0.295	0.33	down	FOXA1
A_32_P199551	0.0487592	0.315	0.33	down	SPTSSB
A_33_P3342481	0.0014429	0.0737	0.33	down	THC2723408
A_24_P341000	0.0028578	0.0925	0.33	down	ENST00000318245
A_33_P3299416	0.0000409	0.0229	0.34	down	GRASP
A_32_P190769	0.0000454	0.0239	0.34	down	C1QTNF7
A_23_P204879	0.0000884	0.0302	0.34	down	CAB39L
A_23_P100386	0.0005995	0.0555	0.34	down	IL34
A_23_P69497	0.0010972	0.0668	0.34	down	CLEC3B
A_33_P3336700	0.0013687	0.0722	0.34	down	SHROOM3
A_23_P142310	0.0019638	0.0813	0.34	down	MKNK2
A_24_P410797	0.0027076	0.0908	0.34	down	KALRN
A_24_P273742	0.0052778	0.119	0.34	down	TTC9
A_33_P3330503	0.008246	0.146	0.34	down	ALDH7A1
A_23_P102331	0.008901	0.151	0.34	down	SCN7A
A_23_P129064	0.0102307	0.16	0.34	down	GATM
A_33_P3279847	0.0105856	0.162	0.34	down	RAET1E
A_24_P27234	0.0110445	0.165	0.34	down	SOX5
A_23_P315836	0.0124975	0.174	0.34	down	BAIAP2
A_33_P3401902	0.0137312	0.181	0.34	down	ANKRD20A2
A_23_P76749	0.0139218	0.182	0.34	down	GALNTL1
A_33_P3780983	0.0146408	0.187	0.34	down	SLC34A3
A_33_P3411975	0.0165341	0.198	0.34	down	ABI3BP
A_23_P162211	0.0171459	0.2	0.34	down	MANSC1
A_24_P165423	0.0231607	0.229	0.34	down	RBP7
A_33_P3258801	0.0236441	0.231	0.34	down	SLURP1
A_33_P3403117	0.0253145	0.237	0.34	down	NR2F1
A_23_P372834	0.0268326	0.243	0.34	down	AQP1
A_24_P344961	0.0385023	0.285	0.34	down	AMOT
A_24_P325520	0.0001184	0.0326	0.35	down	SORT1
A_24_P414256	0.0004168	0.0471	0.35	down	CCDC72
A_23_P110167	0.0012347	0.0692	0.35	down	MGST2
A_33_P3324805	0.0018366	0.0791	0.35	down	LOC100130345
A_23_P77328	0.001879	0.0798	0.35	down	GCHFR
A_23_P373464	0.0032449	0.0982	0.35	down	AFF3
A_24_P7121	0.0042095	0.11	0.35	down	NSUN7
A_23_P171143	0.0063715	0.13	0.35	down	TSPAN6
A_32_P59678	0.0072204	0.137	0.35	down	C7orf46
A_33_P3390868	0.0082315	0.146	0.35	down	SYNPO2
A_33_P3377190	0.0093988	0.154	0.35	down	LOC100131662

A_24_P331830	0.0148832	0.188	0.35	down	KAZN
A_33_P3388646	0.0167757	0.199	0.35	down	ABLIM1
A_33_P3347397	0.0193304	0.21	0.35	down	AMOT
A_23_P41789	0.0239103	0.232	0.35	down	SLC27A6
A_23_P121657	0.0408421	0.292	0.35	down	HS3ST1
A_23_P319859	0.0417006	0.295	0.35	down	EYA2
A_23_P135381	0.0445102	0.303	0.35	down	SP5
A_32_P4792	0.0003996	0.0465	0.35	down	ENST00000344538
A_24_P19544	0.0000085	0.0158	0.36	down	STK39
A_23_P140029	0.0001482	0.0355	0.36	down	UBL3
A_24_P140666	0.0017517	0.0779	0.36	down	N4BP3
A_33_P3258723	0.0018076	0.079	0.36	down	RMND5B
A_23_P94736	0.0018303	0.0791	0.36	down	ST6GALNAC4
A_24_P112160	0.0019717	0.0814	0.36	down	UPK3B
A_23_P29067	0.0025712	0.0892	0.36	down	TMPRSS2
A_23_P170649	0.0026592	0.0902	0.36	down	C8orf84
A_23_P29975	0.0027027	0.0907	0.36	down	C4orf19
A_23_P81158	0.0037979	0.104	0.36	down	ADH1C
A_23_P158041	0.0038386	0.105	0.36	down	AQP7
A_32_P130630	0.0045853	0.113	0.36	down	SLC16A7
A_24_P309095	0.0057942	0.124	0.36	down	RELN
A_33_P3293432	0.006671	0.132	0.36	down	LRRC37A3
A_23_P167168	0.0109846	0.164	0.36	down	IGJ
A_23_P55682	0.0130875	0.178	0.36	down	ZSCAN18
A_23_P43763	0.0132205	0.179	0.36	down	PLLP
A_23_P211909	0.016441	0.197	0.36	down	PLS1
A_23_P160214	0.0200822	0.214	0.36	down	TTC39A
A_33_P3267248	0.0318503	0.262	0.36	down	TDRD10
A_23_P205531	0.039839	0.289	0.36	down	RNASE4
A_24_P97825	0.0478574	0.313	0.36	down	CCDC69
A_21_P0000102	0.0004555	0.0493	0.36	down	MTRNR2L2
A_33_P3309222	0.0024022	0.0875	0.36	down	ENST00000400831
A_21_P0013633	0.0065935	0.132	0.36	down	ANKRD18B
A_21_P0013675	0.0161932	0.196	0.36	down	ANKRD18B
A_23_P426944	0.000232	0.0392	0.37	down	PAX9
A_33_P3247838	0.0002964	0.0414	0.37	down	ZNF844
A_23_P380309	0.00375	0.103	0.37	down	C18orf26
A_33_P3313532	0.0051393	0.118	0.37	down	ANKRD20A1
A_33_P3248992	0.0064748	0.131	0.37	down	ACADSB
A_32_P223777	0.0094393	0.154	0.37	down	IL6ST
A_33_P3321697	0.0101876	0.16	0.37	down	EPB41L4A
A_33_P3307049	0.0102449	0.16	0.37	down	LOC100129363
A_33_P3268487	0.0171634	0.2	0.37	down	ABAT
A_24_P332081	0.0189447	0.209	0.37	down	JAKMIP3

A_33_P3733417	0.0195214	0.211	0.37	down	DRD2
A_24_P408736	0.0198328	0.213	0.37	down	GALNT5
A_24_P381441	0.0215872	0.222	0.37	down	LMO3
A_32_P80850	0.0223411	0.225	0.37	down	COL14A1
A_32_P79434	0.0292094	0.252	0.37	down	PTPRN2
A_23_P251937	0.0329017	0.266	0.37	down	CPEB4
A_33_P3329974	0.0371834	0.28	0.37	down	CGN
A_33_P3216763	0.0376459	0.282	0.37	down	EYA1
A_24_P398130	0.03878	0.286	0.37	down	USP6NL
A_33_P3258593	0.0471425	0.311	0.37	down	PRB1
A_23_P145555	0.0498197	0.318	0.37	down	MLIP
A_21_P0004641	0.0208299	0.218	0.37	down	MUC22
A_23_P61447	0.0006998	0.0579	0.38	down	ETFDH
A_32_P52609	0.0010703	0.0665	0.38	down	LPIN1
A_23_P64919	0.0011205	0.0671	0.38	down	RERGL
A_23_P431268	0.0011916	0.0687	0.38	down	PLEKHA6
A_23_P86195	0.001266	0.0698	0.38	down	SLC44A3
A_23_P46812	0.0015828	0.0761	0.38	down	CPEB3
A_33_P3356070	0.0023222	0.086	0.38	down	LPIN1
A_23_P132910	0.0049251	0.116	0.38	down	RBM47
A_33_P3417328	0.0142317	0.184	0.38	down	SHROOM3
A_23_P42975	0.0143439	0.185	0.38	down	PRKAR2B
A_23_P23829	0.0173474	0.201	0.38	down	CD34
A_32_P125771	0.0220575	0.223	0.38	down	RGS22
A_33_P3319987	0.022543	0.226	0.38	down	SCML4
A_33_P3228558	0.0237772	0.231	0.38	down	ARHGAP27
A_33_P3313825	0.0260812	0.24	0.38	down	TGFBR2
A_23_P113111	0.036938	0.279	0.38	down	AR
A_33_P3302652	0.0014127	0.073	0.38	down	ENST00000415882
A_33_P3417472	0.0041344	0.109	0.38	down	ENST00000412492
A_23_P99853	0.001862	0.0795	0.39	down	KIAA1370
A_33_P3335498	0.0003803	0.0459	0.39	down	KLF8
A_33_P3335910	0.0004383	0.0484	0.39	down	SYNE1
A_24_P181055	0.0014064	0.0728	0.39	down	ST3GAL4
A_33_P3217700	0.0017729	0.0783	0.39	down	USP9Y
A_23_P120364	0.0032042	0.0975	0.39	down	PPDPF
A_33_P3423365	0.0045576	0.113	0.39	down	GSN
A_33_P3346766	0.0048139	0.115	0.39	down	UGT2A2
A_23_P352619	0.0075299	0.14	0.39	down	TIFA
A_33_P3226167	0.0084146	0.147	0.39	down	FAM129B
A_23_P94319	0.0109229	0.164	0.39	down	KBTBD11
A_33_P3712341	0.0109351	0.164	0.39	down	CXCL12
A_33_P3212729	0.0156478	0.193	0.39	down	PDZRN4
A_23_P163195	0.0161856	0.196	0.39	down	LRFN5

A_24_P67534	0.0174065	0.202	0.39	down	CXorf69
A_23_P409623	0.0212324	0.22	0.39	down	PPFIBP2
A_23_P324384	0.0219419	0.223	0.39	down	RPS4Y2
A_23_P106922	0.024989	0.236	0.39	down	CHST6
A_32_P216635	0.0261094	0.24	0.39	down	KSR2
A_23_P28246	0.0319876	0.263	0.39	down	SLC23A3
A_24_P657226	0.0375217	0.281	0.39	down	SLC9B1
A_33_P3423420	0.0487884	0.316	0.39	down	ZNF750
A_33_P3333364	0.0006261	0.0567	0.39	down	ENST00000390287
A_33_P3388016	0.0003041	0.0416	0.4	down	CCDC72
A_23_P85952	0.000388	0.046	0.4	down	DENND2D
A_23_P36753	0.0012588	0.0698	0.4	down	ALDH2
A_24_P945283	0.0031016	0.0958	0.4	down	DLG3
A_33_P3239242	0.0054034	0.121	0.4	down	SPATA6
A_23_P390148	0.0058485	0.125	0.4	down	RALGPS1
A_23_P407206	0.0085121	0.148	0.4	down	CLN8
A_33_P3799936	0.0087265	0.15	0.4	down	ARHGEF10L
A_23_P419107	0.0096669	0.156	0.4	down	TCP11L2
A_33_P3299487	0.0107748	0.163	0.4	down	AIM1L
A_33_P3342663	0.014671	0.187	0.4	down	ADAM33
A_23_P31073	0.0171974	0.201	0.4	down	MYB
A_33_P3209831	0.0176941	0.203	0.4	down	ZNF345
A_23_P34375	0.0194929	0.211	0.4	down	TCEA3
A_23_P19778	0.0258559	0.239	0.4	down	SLC13A4
A_23_P87791	0.0273232	0.245	0.4	down	ACACB
A_23_P215744	0.0284071	0.249	0.4	down	CTTNBP2
A_23_P145529	0.028555	0.249	0.4	down	PKIB
A_23_P30315	0.0386066	0.285	0.4	down	TRIM7
A_24_P11436	0.0398219	0.289	0.4	down	TTC22
A_23_P41476	0.0445641	0.303	0.4	down	SHISA3
A_33_P3338484	0.0269534	0.244	0.4	down	BX476374
A_32_P524014	0.0000577	0.0256	0.41	down	UTRN
A_23_P218086	0.0008715	0.0621	0.41	down	TPCN1
A_23_P325155	0.0011032	0.0668	0.41	down	CD200R1
A_23_P366812	0.0018297	0.0791	0.41	down	AQP5
A_24_P226108	0.0028988	0.0932	0.41	down	RBM47
A_23_P213518	0.0037937	0.104	0.41	down	CAST
A_24_P257579	0.0072779	0.138	0.41	down	EPB41L4A
A_33_P3379091	0.0092764	0.153	0.41	down	SYNGR1
A_33_P3241433	0.0104496	0.161	0.41	down	ZNF347
A_23_P409168	0.010999	0.164	0.41	down	NBEAL2
A_23_P434352	0.011386	0.166	0.41	down	CAST
A_23_P17695	0.0134936	0.18	0.41	down	SLC37A1
A_23_P105571	0.0143545	0.185	0.41	down	CHPT1

A_33_P3369520	0.0146021	0.187	0.41	down	MAGI3
A_33_P3350553	0.0153794	0.191	0.41	down	UXT
A_33_P3370570	0.018503	0.207	0.41	down	AQPEP
A_33_P3215575	0.0191021	0.21	0.41	down	ARHGEF10L
A_24_P185709	0.0224667	0.226	0.41	down	EPB41L1
A_33_P3300262	0.0250476	0.236	0.41	down	VIT
A_23_P114740	0.0271619	0.245	0.41	down	CFH
A_33_P3325700	0.0282651	0.249	0.41	down	DIO2
A_33_P3234202	0.0289962	0.251	0.41	down	DNASE1L3
A_23_P122068	0.031611	0.261	0.41	down	C1QTNF3
A_23_P327483	0.033949	0.27	0.41	down	SPATA6
A_23_P143902	0.0345074	0.271	0.41	down	P2RY12
A_23_P121614	0.0420116	0.295	0.41	down	MUC7
A_23_P13065	0.0442431	0.302	0.41	down	ZDHHC13
A_32_P42946	0.0448405	0.304	0.41	down	C1orf210
A_33_P3323959	0.045661	0.306	0.41	down	RELN
A_33_P3388651	0.0483084	0.314	0.41	down	ABLIM1
A_33_P3233869	0.0023174	0.086	0.41	down	ENST00000437267
A_23_P165061	0.0003668	0.0453	0.42	down	AES
A_23_P74229	0.0007508	0.0585	0.42	down	STK40
A_24_P100016	0.0008416	0.0607	0.42	down	ZNF486
A_33_P3366833	0.0011004	0.0668	0.42	down	CCDC73
A_23_P155417	0.0011162	0.067	0.42	down	ABHD14B
A_23_P212002	0.0015665	0.0757	0.42	down	NKTR
A_23_P18447	0.0017134	0.0776	0.42	down	PPARGC1A
A_33_P3297888	0.0019663	0.0814	0.42	down	CCDC125
A_33_P3387050	0.0020491	0.083	0.42	down	C8orf82
A_33_P3382835	0.0023446	0.0864	0.42	down	NYNRIN
A_23_P432591	0.0030491	0.0951	0.42	down	CCDC125
A_23_P392470	0.0038867	0.106	0.42	down	NR3C2
A_23_P50349	0.0053884	0.121	0.42	down	TRIP10
A_32_P61857	0.0066679	0.132	0.42	down	KIAA1468
A_33_P3229370	0.0067853	0.133	0.42	down	ID4
A_23_P339119	0.0068287	0.133	0.42	down	ACSS3
A_24_P333421	0.009059	0.152	0.42	down	ZNF862
A_24_P119577	0.0094558	0.154	0.42	down	PHACTR4
A_23_P333683	0.0097746	0.157	0.42	down	IGSF10
A_33_P3288942	0.0101937	0.16	0.42	down	FAM107B
A_33_P3217230	0.0117436	0.169	0.42	down	ZNF91
A_23_P92928	0.0124047	0.173	0.42	down	C6
A_23_P22350	0.0151199	0.19	0.42	down	GRAMD3
A_23_P8938	0.0186521	0.208	0.42	down	ADRA1A
A_24_P367079	0.025494	0.238	0.42	down	SFTA2
A_33_P3221989	0.0256009	0.238	0.42	down	CACNB4

A_23_P259314	0.0266618	0.243	0.42	down	RPS4Y1
A_33_P3334220	0.0272617	0.245	0.42	down	ACACB
A_23_P332399	0.0305035	0.257	0.42	down	GULP1
A_23_P116902	0.0329976	0.266	0.42	down	ART4
A_23_P45185	0.0351043	0.273	0.42	down	FIGF
A_23_P38735	0.043154	0.299	0.42	down	CDH19
A_33_P3310567	0.0230675	0.228	0.42	down	THC2670340
A_33_P3308137	0.0001339	0.0339	0.43	down	C7orf46
A_23_P56578	0.0003145	0.042	0.43	down	VIT
A_33_P3346444	0.0005242	0.0523	0.43	down	PCDH11Y
A_33_P3258600	0.0014874	0.0746	0.43	down	NYNRIN
A_24_P29401	0.0017075	0.0774	0.43	down	PIK3R1
A_23_P255884	0.0017713	0.0782	0.43	down	GSN
A_23_P10077	0.0019204	0.0806	0.43	down	PNPLA2
A_33_P3266730	0.0032676	0.0983	0.43	down	SYTL4
A_23_P256603	0.0036707	0.103	0.43	down	MLLT4
A_33_P3271001	0.0050021	0.116	0.43	down	NIPSNAP3B
A_23_P401106	0.007584	0.14	0.43	down	PDE2A
A_23_P140434	0.0084684	0.148	0.43	down	MYO5C
A_32_P60223	0.0103895	0.161	0.43	down	ING5
A_33_P3358731	0.0108682	0.164	0.43	down	PCSK5
A_33_P3285580	0.010981	0.164	0.43	down	GLYCTK
A_24_P375205	0.0111623	0.165	0.43	down	MKL2
A_33_P3252925	0.0120692	0.171	0.43	down	PHF17
A_23_P157109	0.0122288	0.172	0.43	down	ADCYAP1R1
A_33_P3331996	0.0141641	0.184	0.43	down	LOC100509860
A_24_P148590	0.0143578	0.185	0.43	down	TACR1
A_24_P40551	0.019209	0.21	0.43	down	BEX4
A_23_P414654	0.0206731	0.218	0.43	down	RAB37
A_23_P436353	0.0231907	0.229	0.43	down	MLLT4
A_23_P53884	0.0265119	0.242	0.43	down	MAB21L1
A_33_P3334205	0.02735	0.245	0.43	down	TMEM184A
A_23_P110712	0.0335939	0.269	0.43	down	DUSP1
A_23_P253012	0.0363524	0.278	0.43	down	GRAMD1C
A_23_P13822	0.0374244	0.281	0.43	down	STYK1
A_33_P3281028	0.0393302	0.287	0.43	down	MACROD2
A_33_P3284813	0.0465905	0.309	0.43	down	C10orf113
A_33_P3363500	0.0010848	0.0668	0.43	down	THC2723408
A_19_P00315633	0.0062697	0.129	0.43	down	ENST00000449573
A_24_P522786	0.0082347	0.146	0.43	down	ENST00000400831
A_33_P3336023	0.0407602	0.292	0.43	down	BC050023
A_33_P3321993	0.0437519	0.301	0.43	down	THC2550350
A_33_P3390122	0.0000025	0.0121	0.44	down	GIPC2
A_23_P155666	0.0001465	0.0355	0.44	down	NAAA

A_23_P316381	0.0002115	0.0379	0.44	down	ACOX3
A_23_P254353	0.0004367	0.0483	0.44	down	NOXA1
A_33_P3297217	0.000605	0.0558	0.44	down	NAAA
A_33_P3244083	0.0010734	0.0666	0.44	down	KIF13B
A_33_P3287939	0.0022878	0.0858	0.44	down	C7orf46
A_24_P310256	0.0026651	0.0903	0.44	down	LGI4
A_23_P127267	0.0028842	0.0931	0.44	down	LGI1
A_23_P53788	0.0044447	0.112	0.44	down	MTIF3
A_23_P502654	0.0077645	0.142	0.44	down	SHMT1
A_24_P929754	0.0094518	0.154	0.44	down	MKNK2
A_23_P399001	0.0123574	0.173	0.44	down	CXXC5
A_23_P128919	0.0136632	0.181	0.44	down	LGALS3
A_23_P171107	0.0138156	0.182	0.44	down	CCNB3
A_24_P80532	0.0139024	0.182	0.44	down	CCNG2
A_23_P207868	0.0167532	0.199	0.44	down	KRTAP9-4
A_23_P424513	0.0176768	0.203	0.44	down	RANBP9
A_23_P149975	0.0186885	0.208	0.44	down	FAM107B
A_33_P3725324	0.0187534	0.208	0.44	down	USP9Y
A_23_P143694	0.0235038	0.23	0.44	down	SOX10
A_23_P349083	0.0259584	0.24	0.44	down	FCHO2
A_23_P427023	0.030078	0.256	0.44	down	GIMAP1
A_23_P348749	0.0311595	0.26	0.44	down	AKD1
A_23_P44674	0.03395	0.27	0.44	down	CRIP1
A_24_P299423	0.0348918	0.273	0.44	down	IL16
A_33_P3394065	0.0396895	0.289	0.44	down	PIP4K2A
A_32_P53524	0.0399667	0.289	0.44	down	NTN1
A_24_P62800	0.0417253	0.295	0.44	down	KLHDC8A
A_23_P49448	0.0418633	0.295	0.44	down	FA2H
A_23_P7503	0.0454683	0.305	0.44	down	TIMD4
A_21_P0009481	0.0052669	0.119	0.44	down	RAB27B
A_21_P0013973	0.0071078	0.136	0.44	down	LOC100507161
A_19_P00317887	0.0197674	0.213	0.44	down	MPRIP
A_33_P3343101	0.0004132	0.047	0.45	down	ARGLU1
A_33_P3346635	0.0004901	0.0512	0.45	down	LOC100132244
A_33_P3363460	0.0005804	0.055	0.45	down	LOC100129186
A_23_P114689	0.0009225	0.0632	0.45	down	ASAP3
A_23_P123563	0.0012997	0.0705	0.45	down	RPS6
A_23_P49988	0.001663	0.0766	0.45	down	KRTAP4-2
A_23_P388433	0.0025007	0.0886	0.45	down	C4orf3
A_33_P3369058	0.0029036	0.0932	0.45	down	LRRK2
A_33_P3342942	0.0030662	0.0954	0.45	down	MEIS1
A_33_P3841621	0.0044405	0.112	0.45	down	MYO5B
A_33_P3279241	0.0049862	0.116	0.45	down	ZNF608
A_24_P382319	0.0057214	0.123	0.45	down	CEACAM1

A_32_P150632	0.0057669	0.124	0.45	down	ANXA11
A_33_P3290409	0.00673	0.133	0.45	down	LOC100128402
A_23_P133543	0.0077471	0.142	0.45	down	KLHL3
A_24_P272160	0.0124131	0.173	0.45	down	NBEAL2
A_23_P348227	0.0166819	0.198	0.45	down	ZNF135
A_23_P217570	0.0171404	0.2	0.45	down	CAPN6
A_23_P142878	0.0174149	0.202	0.45	down	ATOH8
A_33_P3236676	0.0175572	0.202	0.45	down	C9orf152
A_23_P96590	0.0204611	0.216	0.45	down	GPRASP1
A_33_P3347281	0.0227647	0.227	0.45	down	HNMT
A_23_P64837	0.022818	0.227	0.45	down	SMAGP
A_23_P169003	0.0259871	0.24	0.45	down	SH2D4A
A_24_P932939	0.0260886	0.24	0.45	down	LOC401052
A_23_P99275	0.0348327	0.273	0.45	down	KLRB1
A_33_P3372074	0.0462006	0.308	0.45	down	LLGL2
A_23_P101351	0.0481279	0.313	0.45	down	ZNF426
A_23_P139600	0.0493641	0.317	0.45	down	RASAL1
A_21_P0013997	0.0037397	0.103	0.45	down	LOC100509620
A_33_P3374117	0.0122039	0.172	0.45	down	ENST00000475369
A_33_P3393175	0.0003651	0.0452	0.46	down	CAPN7
A_23_P355536	0.0008156	0.0601	0.46	down	USP54
A_23_P147822	0.0012864	0.0703	0.46	down	EPS8L2
A_33_P3263061	0.0012914	0.0703	0.46	down	AK3
A_23_P130811	0.0038169	0.104	0.46	down	ZNF536
A_33_P3333272	0.0039226	0.106	0.46	down	TMEM235
A_23_P335957	0.0052361	0.119	0.46	down	TAS1R2
A_23_P81392	0.0055423	0.122	0.46	down	WWC1
A_24_P177568	0.0055984	0.123	0.46	down	ZNF431
A_33_P3397323	0.007331	0.138	0.46	down	ZNF441
A_33_P3241521	0.007527	0.14	0.46	down	EBF1
A_24_P238143	0.0084751	0.148	0.46	down	LRRC37A2
A_33_P3300747	0.0088382	0.15	0.46	down	ADHFE1
A_23_P200710	0.0094945	0.155	0.46	down	PIK3C2B
A_23_P105227	0.0161283	0.196	0.46	down	ME3
A_23_P32707	0.0171258	0.2	0.46	down	ESPL1
A_23_P137238	0.0175901	0.203	0.46	down	KDM5D
A_33_P3397288	0.017647	0.203	0.46	down	EDN3
A_24_P941831	0.0176886	0.203	0.46	down	TMEM237
A_33_P3273669	0.0216941	0.222	0.46	down	NRXN1
A_32_P225816	0.0250123	0.236	0.46	down	PRDM16
A_33_P3361337	0.0295851	0.254	0.46	down	TMEM184A
A_23_P202071	0.0337274	0.269	0.46	down	CELF2
A_33_P3402404	0.0338979	0.269	0.46	down	SCN3B
A_32_P383681	0.037891	0.282	0.46	down	DNAH6

A_33_P3256793	0.0426003	0.298	0.46	down	KIAA1324
A_24_P47988	0.043668	0.301	0.46	down	ELL3
A_32_P79190	0.0007821	0.0592	0.46	down	ENST00000456460
A_19_P00321063	0.0201362	0.215	0.46	down	Q5SRJ3
A_21_P0000188	0.0256495	0.238	0.46	down	ELF5
A_24_P365679	0.0320514	0.263	0.46	down	ENST00000357802
A_32_P352358	0.0000808	0.0294	0.47	down	LOC650293
A_33_P3335183	0.0007862	0.0592	0.47	down	LRRC37A3
A_23_P113471	0.0009375	0.0636	0.47	down	FAAH2
A_24_P305960	0.0028061	0.0918	0.47	down	LEMD2
A_23_P213699	0.0044634	0.112	0.47	down	NRG2
A_32_P128701	0.0049853	0.116	0.47	down	USP53
A_32_P148275	0.0056077	0.123	0.47	down	SPIC
A_33_P3230818	0.0092089	0.153	0.47	down	RCAN2
A_23_P257003	0.0103848	0.161	0.47	down	PCSK5
A_33_P3417339	0.0134489	0.18	0.47	down	SHROOM3
A_33_P3342633	0.0159485	0.195	0.47	down	DHRS12
A_33_P3369920	0.0161005	0.195	0.47	down	LOC100128402
A_23_P306307	0.0180157	0.205	0.47	down	OTOP1
A_23_P130444	0.0188506	0.209	0.47	down	ZNF701
A_24_P18270	0.0237245	0.231	0.47	down	UCMA
A_23_P258002	0.0238321	0.231	0.47	down	CDKN2AIP
A_23_P81280	0.0261689	0.241	0.47	down	BTNL9
A_33_P3892608	0.0319368	0.263	0.47	down	KCNT2
A_23_P112531	0.0355253	0.275	0.47	down	FAM102A
A_33_P3273324	0.0359241	0.276	0.47	down	ZNF880
A_23_P408249	0.0366941	0.279	0.47	down	PCK1
A_33_P3317937	0.0379584	0.282	0.47	down	KRTAP20-1
A_21_P0013987	0.0108234	0.164	0.47	down	LOC100509780
A_33_P3356022	0.0171491	0.2	0.47	down	ENST00000390462
A_21_P0013246	0.0368216	0.279	0.47	down	UPK3B
A_23_P160154	0.0001933	0.0372	0.48	down	GALE
A_24_P309645	0.0012635	0.0698	0.48	down	TPCN1
A_23_P18142	0.0017059	0.0774	0.48	down	RPL32
A_24_P476086	0.0020224	0.0826	0.48	down	KPNA5
A_23_P96761	0.0020451	0.0829	0.48	down	ACADM
A_33_P3408643	0.0021534	0.084	0.48	down	FAM82A1
A_33_P3215113	0.0022371	0.0853	0.48	down	LDOC1
A_24_P85258	0.0025442	0.0891	0.48	down	KIAA1751
A_24_P319736	0.00293	0.0935	0.48	down	MEIS1
A_24_P341504	0.0057706	0.124	0.48	down	RNPC3
A_23_P317756	0.0064419	0.131	0.48	down	ACSM3
A_33_P3372044	0.0095767	0.156	0.48	down	TPRG1
A_24_P350589	0.0096866	0.156	0.48	down	RNF150

A_23_P364414	0.0110077	0.164	0.48	down	PCDHGA9
A_33_P3410284	0.0118392	0.17	0.48	down	DOCK9
A_33_P3235321	0.0122943	0.173	0.48	down	SYTL1
A_23_P355394	0.0123627	0.173	0.48	down	STK32A
A_33_P3368139	0.0128311	0.176	0.48	down	MAP3K1
A_24_P412156	0.0149631	0.189	0.48	down	CXCL12
A_23_P22444	0.0241292	0.232	0.48	down	CFP
A_33_P3227990	0.0241554	0.232	0.48	down	MBP
A_23_P144476	0.0249212	0.236	0.48	down	SPRY1
A_33_P3379341	0.0250855	0.236	0.48	down	LOC391322
A_23_P214080	0.0283664	0.249	0.48	down	EGR1
A_33_P3374324	0.0339258	0.269	0.48	down	OR51I1
A_23_P433229	0.0428658	0.299	0.48	down	PHYHIP
A_23_P370684	0.0431301	0.299	0.48	down	TIGD4
A_23_P163467	0.0440199	0.302	0.48	down	C15orf52
A_23_P432545	0.0493944	0.317	0.48	down	EFCAB4A
A_21_P0010639	0.0005053	0.0518	0.48	down	RPSA
A_21_P0010756	0.0058571	0.125	0.48	down	CYP4Z1
A_21_P0012275	0.0159984	0.195	0.48	down	SLC9B1
A_33_P3287218	0.0000016	0.0112	0.49	down	GSTK1
A_33_P3252635	0.0005194	0.0521	0.49	down	ZNF254
A_33_P3267948	0.0008239	0.0603	0.49	down	SLC25A23
A_23_P332960	0.002673	0.0904	0.49	down	TMEM80
A_33_P3353732	0.0027578	0.0914	0.49	down	LOC100131581
A_33_P3221129	0.0033602	0.0994	0.49	down	LRRN4CL
A_33_P3234859	0.0048724	0.115	0.49	down	UTRN
A_23_P129005	0.0053573	0.12	0.49	down	NYNRIN
A_33_P3404899	0.0056311	0.123	0.49	down	LPHN2
A_23_P125748	0.0074509	0.139	0.49	down	ZMAT1
A_33_P3330453	0.0075732	0.14	0.49	down	PBRM1
A_24_P9090	0.0091807	0.152	0.49	down	HNRPDL
A_23_P107612	0.0103594	0.161	0.49	down	RAB27B
A_33_P3409746	0.010611	0.162	0.49	down	C11orf91
A_33_P3410454	0.0118131	0.17	0.49	down	GTF2IRD2B
A_23_P200203	0.013099	0.178	0.49	down	ECHDC2
A_23_P162766	0.0147951	0.188	0.49	down	DOCK9
A_23_P420610	0.0166962	0.198	0.49	down	FCHO2
A_33_P3364389	0.0170563	0.2	0.49	down	SYT8
A_33_P3300858	0.0183699	0.206	0.49	down	OR8H3
A_23_P153676	0.0236715	0.231	0.49	down	TLE2
A_24_P942743	0.0266182	0.242	0.49	down	ZFY
A_23_P78053	0.0271822	0.245	0.49	down	FAM117A
A_23_P18123	0.0299236	0.255	0.49	down	NLGN1
A_33_P3236020	0.0425586	0.297	0.49	down	TIMD4

A_23_P67529	0.0429578	0.299	0.49	down	KCNN4
A_33_P3215640	0.0454201	0.305	0.49	down	PI16
A_33_P3228435	0.0463169	0.308	0.49	down	FXYD1
A_21_P0000010	0.0016869	0.0771	0.49	down	FKBP5
A_23_P15123	0.0004133	0.047	2.01	up	UBFD1
A_33_P3364379	0.0006444	0.0568	2.01	up	YIF1A
A_33_P3266410	0.0018176	0.079	2.01	up	MAZ
A_23_P358221	0.0018998	0.0802	2.01	up	UBXN7
A_24_P219378	0.0034616	0.1	2.01	up	CASKIN1
A_33_P3398840	0.004858	0.115	2.01	up	ZBED1
A_33_P3287760	0.0074241	0.139	2.01	up	RASGEF1A
A_24_P336276	0.0086968	0.149	2.01	up	SLCO3A1
A_23_P10870	0.0102424	0.16	2.01	up	DOLK
A_24_P58337	0.0167442	0.199	2.01	up	FTH1
A_23_P149200	0.0192098	0.21	2.01	up	CDC20
A_33_P3340580	0.0227672	0.227	2.01	up	ZNF385C
A_23_P69437	0.0258663	0.239	2.01	up	YEATS2
A_33_P3239587	0.0308218	0.258	2.01	up	MXRA7
A_23_P80098	0.0398264	0.289	2.01	up	GART
A_23_P207850	0.0450932	0.304	2.01	up	TNS4
A_24_P214231	0.0495899	0.318	2.01	up	STIL
A_24_P12904	0.0022222	0.0852	2.02	up	C9orf5
A_23_P109470	0.0001287	0.0332	2.02	up	THOC5
A_23_P113005	0.0003464	0.0444	2.02	up	EFNA1
A_23_P142075	0.001867	0.0795	2.02	up	ACP5
A_24_P416346	0.0023017	0.086	2.02	up	ETV4
A_23_P8055	0.0024606	0.0881	2.02	up	NRM
A_23_P164000	0.003675	0.103	2.02	up	SENP3
A_23_P160025	0.0042862	0.11	2.02	up	IFI16
A_24_P58529	0.0081791	0.145	2.02	up	TUBA1C
A_23_P41804	0.0118774	0.17	2.02	up	NKD2
A_33_P3407524	0.0159709	0.195	2.02	up	SUV39H1
A_23_P48109	0.023218	0.229	2.02	up	NINJ2
A_23_P310911	0.0237957	0.231	2.02	up	BLMH
A_33_P3380098	0.032535	0.265	2.02	up	PXN
A_24_P945000	0.0373785	0.281	2.02	up	SKA2
A_23_P43255	0.0401752	0.29	2.02	up	FAM49B
A_23_P42198	0.0448738	0.304	2.02	up	HIST1H3G
A_33_P3366156	0.0479047	0.313	2.02	up	SPATA9
A_23_P89884	0.0002657	0.0403	2.03	up	TRIM28
A_23_P163258	0.000643	0.0568	2.03	up	PARP6
A_23_P137423	0.0011834	0.0684	2.03	up	IGSF8
A_23_P136232	0.0049514	0.116	2.03	up	IMPAD1
A_23_P79794	0.0057318	0.124	2.03	up	TGIF2

A_32_P6015	0.0063041	0.129	2.03	up	MNX1
A_23_P300600	0.0140683	0.183	2.03	up	NEFH
A_23_P26771	0.0219513	0.223	2.03	up	CD300C
A_33_P3373614	0.0225869	0.226	2.03	up	EFR3B
A_33_P3266646	0.044079	0.302	2.03	up	ATP8B3
A_24_P207139	0.0459074	0.307	2.03	up	PML
A_23_P148984	0.0471471	0.311	2.03	up	DARS2
A_23_P89460	0.0002378	0.0393	2.04	up	AATF
A_33_P3418597	0.0003798	0.0459	2.04	up	GAS2L1
A_24_P362317	0.0041674	0.109	2.04	up	ADAR
A_24_P231026	0.0068598	0.133	2.04	up	SCN8A
A_32_P47754	0.0083837	0.147	2.04	up	SLC2A14
A_23_P209904	0.008525	0.148	2.04	up	GPC1
A_33_P3259801	0.0239486	0.232	2.04	up	TMEM189
A_23_P152356	0.0267899	0.243	2.04	up	ZNF200
A_23_P168828	0.0340485	0.27	2.04	up	KLF10
A_23_P72077	0.0386022	0.285	2.04	up	IL12RB2
A_23_P255523	0.0443362	0.303	2.04	up	ALKBH4
A_33_P3361147	0.0361926	0.277	2.04	up	ENST00000371162
A_33_P3403400	0.002581	0.0893	2.05	up	C6orf127
A_33_P3390521	0.0000019	0.0116	2.05	up	UBE2Z
A_23_P168443	0.0000282	0.0207	2.05	up	EPHB4
A_33_P3371954	0.0001651	0.0364	2.05	up	IGSF8
A_23_P136238	0.0001935	0.0372	2.05	up	TMEM203
A_33_P3321034	0.000361	0.0449	2.05	up	PLXNA4
A_23_P129101	0.0004112	0.047	2.05	up	HEXA
A_23_P152272	0.0005632	0.0542	2.05	up	RNPS1
A_23_P5831	0.000639	0.0568	2.05	up	HPCAL1
A_33_P3251430	0.0008525	0.0613	2.05	up	NRIP1
A_32_P95729	0.0009924	0.0651	2.05	up	FANCI
A_23_P422794	0.0019471	0.081	2.05	up	NSMCE2
A_24_P220454	0.0036934	0.103	2.05	up	CUX1
A_33_P3350634	0.0041461	0.109	2.05	up	KIF22
A_23_P332789	0.0044381	0.112	2.05	up	CHRNB4
A_24_P292929	0.0050185	0.116	2.05	up	CAMKK1
A_33_P3367017	0.0091597	0.152	2.05	up	BRMS1
A_24_P115774	0.0091936	0.153	2.05	up	BIRC2
A_23_P207125	0.0135877	0.18	2.05	up	NLGN2
A_33_P3409392	0.0140299	0.183	2.05	up	FZD6
A_24_P101704	0.014374	0.185	2.05	up	ROR2
A_33_P3296366	0.0155727	0.192	2.05	up	PODXL2
A_33_P3318796	0.0173996	0.202	2.05	up	FSTL3
A_33_P3239569	0.0184934	0.207	2.05	up	RPS21
A_23_P258321	0.0211734	0.22	2.05	up	MRPS17

A_23_P69720	0.0292782	0.252	2.05	up	ANXA5
A_33_P3286208	0.0330527	0.266	2.05	up	LRR1
A_23_P69383	0.0351871	0.274	2.05	up	PARP9
A_24_P280762	0.0391471	0.287	2.05	up	KHDC1
A_23_P405129	0.0424934	0.297	2.05	up	LTBP2
A_23_P156390	0.0436268	0.301	2.05	up	JAKMIP2
A_32_P93852	0.0002319	0.0392	2.06	up	BOD1
A_23_P120776	0.0020705	0.0833	2.06	up	SLC25A1
A_33_P3240757	0.0022289	0.0852	2.06	up	IGSF3
A_24_P101402	0.0023789	0.087	2.06	up	NOP56
A_23_P137391	0.0038216	0.104	2.06	up	ENO1
A_23_P46309	0.005503	0.122	2.06	up	RCC1
A_23_P49924	0.0068227	0.133	2.06	up	NT5C3L
A_23_P109171	0.0077678	0.142	2.06	up	BFSP1
A_23_P159709	0.0079672	0.143	2.06	up	FOXP3
A_24_P21985	0.0110431	0.165	2.06	up	FOXJ2
A_23_P127948	0.0130298	0.177	2.06	up	ADM
A_23_P365418	0.0224504	0.226	2.06	up	ZNF783
A_23_P379054	0.031142	0.26	2.06	up	CLDN19
A_23_P388993	0.0316297	0.261	2.06	up	ZC3H12C
A_23_P11372	0.0380202	0.283	2.06	up	HPRT1
A_24_P78556	0.039585	0.288	2.06	up	RASSF8
A_21_P0012605	0.0118022	0.17	2.06	up	CEP170
A_23_P112260	0.000593	0.0553	2.07	up	GNG10
A_23_P37347	0.0006915	0.0579	2.07	up	SNW1
A_23_P210708	0.0014062	0.0728	2.07	up	SIRPA
A_23_P79628	0.0020014	0.0821	2.07	up	PSME4
A_23_P124024	0.0030536	0.0952	2.07	up	MED10
A_24_P935009	0.0034714	0.1	2.07	up	CRKL
A_23_P40174	0.0047788	0.115	2.07	up	MMP9
A_23_P407142	0.0049545	0.116	2.07	up	LUZP1
A_23_P11841	0.0053427	0.12	2.07	up	ATP2B4
A_23_P68106	0.0059078	0.125	2.07	up	TMSB10
A_23_P63178	0.0059171	0.126	2.07	up	TAF12
A_33_P3400477	0.0081316	0.145	2.07	up	STIL
A_24_P107859	0.0081807	0.145	2.07	up	SPRED1
A_23_P130359	0.0137379	0.181	2.07	up	ARHGAP28
A_23_P24031	0.0189508	0.209	2.07	up	TLX1
A_32_P155666	0.0191112	0.21	2.07	up	ECEL1
A_23_P101374	0.0280873	0.248	2.07	up	CYP2S1
A_33_P3379941	0.0331643	0.267	2.07	up	HMGXB3
A_23_P167479	0.0395253	0.288	2.07	up	IL17B
A_23_P46351	0.0410712	0.293	2.07	up	TDRKH
A_23_P500381	0.0496457	0.318	2.07	up	HTR7

A_32_P536872	0.0498851	0.318	2.07	up	TDRD5
A_19_P00808208	0.0169033	0.199	2.07	up	CDCA7L
A_24_P148811	0.0005543	0.0535	2.08	up	RUVBL1
A_23_P503233	0.0010172	0.0656	2.08	up	EDARADD
A_24_P53353	0.0021171	0.0838	2.08	up	RAB12
A_23_P23221	0.0027557	0.0914	2.08	up	GADD45A
A_23_P148410	0.0040166	0.107	2.08	up	FTHL17
A_23_P12992	0.0049539	0.116	2.08	up	TRMT112
A_23_P103877	0.0087859	0.15	2.08	up	LRRC38
A_23_P256956	0.0093187	0.153	2.08	up	KIF20A
A_23_P115792	0.0107039	0.163	2.08	up	PLEKHA1
A_23_P171117	0.0109529	0.164	2.08	up	MORC4
A_23_P126939	0.0137998	0.182	2.08	up	RAB7L1
A_23_P148057	0.0156132	0.193	2.08	up	ZDHHC9
A_33_P3256848	0.0187362	0.208	2.08	up	ADAM12
A_23_P389919	0.0255547	0.238	2.08	up	WHSC1
A_23_P251075	0.0258149	0.239	2.08	up	MAMLD1
A_33_P3349404	0.0259502	0.24	2.08	up	CECR2
A_33_P3277198	0.0301205	0.256	2.08	up	CHST10
A_23_P102060	0.0348707	0.273	2.08	up	SSFA2
A_23_P82478	0.0423187	0.297	2.08	up	PUS7
A_23_P12113	0.0427762	0.298	2.08	up	FLVCR1
A_23_P371865	0.0460031	0.307	2.08	up	CDYL2
A_23_P132378	0.0488939	0.316	2.08	up	CELSR1
A_23_P91468	0.0000063	0.0158	2.09	up	PSMA7
A_33_P3235217	0.0001737	0.0364	2.09	up	CTPS
A_23_P66158	0.0007526	0.0585	2.09	up	C16orf88
A_23_P77813	0.0011161	0.067	2.09	up	FN3KRP
A_33_P3876985	0.0022804	0.0858	2.09	up	PPPDE1
A_23_P121447	0.0025803	0.0893	2.09	up	ARPM1
A_23_P74887	0.0035446	0.101	2.09	up	SDC3
A_24_P284893	0.0046526	0.113	2.09	up	PSMB2
A_24_P89843	0.0086691	0.149	2.09	up	CYHR1
A_23_P413815	0.008672	0.149	2.09	up	VKORC1L1
A_24_P42501	0.011949	0.171	2.09	up	ACOT9
A_33_P3296582	0.012972	0.177	2.09	up	ARSJ
A_23_P47565	0.0157852	0.194	2.09	up	LDHA
A_32_P5480	0.01704	0.2	2.09	up	CERS6
A_23_P170959	0.0256556	0.238	2.09	up	LMLN
A_23_P302654	0.0279708	0.248	2.09	up	CEP72
A_33_P3364263	0.0288196	0.25	2.09	up	LBH
A_23_P429491	0.0416599	0.295	2.09	up	C11orf82
A_23_P331049	0.046282	0.308	2.09	up	DPYSL4
A_23_P15542	0.0483275	0.314	2.09	up	HSD17B1

A_19_P00812340	0.0247869	0.235	2.09	up	FTL
A_33_P3237359	0.000349	0.0444	2.1	up	HMGB3
A_23_P71415	0.0003519	0.0446	2.1	up	WDYHV1
A_24_P74160	0.0007523	0.0585	2.1	up	SNRPD2
A_33_P3399840	0.0015651	0.0757	2.1	up	USP39
A_23_P160631	0.0021471	0.084	2.1	up	CCT3
A_23_P102202	0.0021546	0.084	2.1	up	MSH6
A_23_P102404	0.0024091	0.0875	2.1	up	CCT7
A_23_P48886	0.0025897	0.0893	2.1	up	ADAM10
A_24_P364970	0.0047211	0.114	2.1	up	DHX33
A_23_P15394	0.0064157	0.13	2.1	up	CD68
A_23_P171223	0.0067488	0.133	2.1	up	CXorf56
A_23_P64129	0.0091722	0.152	2.1	up	HTATIP2
A_33_P3359047	0.0171445	0.2	2.1	up	LYPD6
A_33_P3317523	0.018975	0.209	2.1	up	STMN1
A_33_P3252800	0.0224474	0.226	2.1	up	PTPRR
A_23_P131202	0.0260637	0.24	2.1	up	HES6
A_23_P153037	0.0403189	0.29	2.1	up	ZNF624
A_33_P3323068	0.0438118	0.301	2.1	up	AGPAT4
A_23_P412515	0.04985	0.318	2.1	up	CLDN12
A_24_P371758	0.003028	0.0946	2.11	up	MRPL36
A_33_P3251144	0.0049854	0.116	2.11	up	CDCA7L
A_33_P3247788	0.0060545	0.127	2.11	up	LOC100128063
A_33_P3259775	0.0069148	0.134	2.11	up	DOCK5
A_23_P121702	0.014983	0.189	2.11	up	OCIAD2
A_23_P200443	0.0162826	0.197	2.11	up	SHC1
A_23_P65442	0.0189321	0.209	2.11	up	IRF9
A_33_P3216227	0.0473007	0.311	2.11	up	ITGB1BP1
A_23_P143274	0.0001168	0.0326	2.12	up	NRSN2
A_33_P3215953	0.0044548	0.112	2.12	up	MPZL1
A_23_P36076	0.00515	0.118	2.12	up	SSRP1
A_23_P210358	0.0078757	0.143	2.12	up	LIMS1
A_33_P3262012	0.0089454	0.151	2.12	up	CHPF
A_23_P212844	0.0110106	0.164	2.12	up	TACC3
A_23_P215566	0.0309705	0.259	2.12	up	AHR
A_33_P3221203	0.0393562	0.287	2.12	up	MMP13
A_23_P154962	0.0400212	0.289	2.12	up	RIMBP3
A_23_P6935	0.0449838	0.304	2.12	up	CD47
A_23_P313223	0.0002625	0.0403	2.13	up	C11orf84
A_23_P330908	0.0002744	0.0403	2.13	up	DERL1
A_23_P145408	0.0004566	0.0493	2.13	up	FUCA2
A_24_P319715	0.0016503	0.0766	2.13	up	PDIA6
A_33_P3228762	0.0029876	0.0941	2.13	up	SLC25A36
A_33_P3398251	0.0082005	0.145	2.13	up	FOXP3

A_33_P3423551	0.0171681	0.2	2.13	up	IER3
A_23_P717	0.0190071	0.209	2.13	up	TMEM206
A_24_P754817	0.0217153	0.222	2.13	up	EFTUD1
A_33_P3293675	0.0224731	0.226	2.13	up	SLC12A7
A_33_P3336287	0.0416392	0.295	2.13	up	SEC61A2
A_33_P3388391	0.0499802	0.318	2.13	up	GJB4
A_33_P3225313	0.0009676	0.0644	2.14	up	FAM91A1
A_23_P170058	0.0011392	0.0674	2.14	up	PSMB2
A_23_P166023	0.0046362	0.113	2.14	up	PFDN4
A_33_P3257708	0.0057953	0.124	2.14	up	APOA1BP
A_33_P3784253	0.006629	0.132	2.14	up	PAK1
A_33_P3400244	0.0066663	0.132	2.14	up	ZNF496
A_23_P368886	0.011927	0.17	2.14	up	CHSY3
A_24_P333663	0.0152563	0.191	2.14	up	MAPK6
A_33_P3282978	0.0153812	0.191	2.14	up	BAALC
A_23_P26687	0.0158724	0.194	2.14	up	TMEM186
A_23_P422212	0.024787	0.235	2.14	up	SLC35F3
A_23_P312174	0.0287391	0.25	2.14	up	ALMS1
A_24_P51855	0.0353085	0.274	2.14	up	DUSP7
A_33_P3392320	0.0452207	0.305	2.14	up	SLC6A15
A_23_P44581	0.0002125	0.0379	2.15	up	NOMO1
A_33_P3310070	0.000684	0.0579	2.15	up	FOXK2
A_33_P3380101	0.001043	0.066	2.15	up	MAP7D1
A_23_P393766	0.0011566	0.0676	2.15	up	WDR62
A_33_P3380346	0.0031942	0.0973	2.15	up	WHSC1
A_23_P125265	0.006859	0.133	2.15	up	KPNA2
A_33_P3321342	0.0085304	0.148	2.15	up	INSIG2
A_33_P3381684	0.0095833	0.156	2.15	up	TCHH
A_33_P3258612	0.0150644	0.189	2.15	up	PCNA
A_33_P3242883	0.0251083	0.236	2.15	up	DLX6
A_33_P3345319	0.0385543	0.285	2.15	up	ZFP64
A_23_P428184	0.0395595	0.288	2.15	up	HIST1H2AD
A_21_P0012282	0.0041115	0.109	2.15	up	GGT1
A_24_P109644	0.0157524	0.193	2.15	up	ENST00000441510
A_23_P210619	0.0002046	0.0376	2.16	up	C20orf43
A_32_P96134	0.0015338	0.0754	2.16	up	DPY19L1
A_23_P145197	0.0034312	0.1	2.16	up	BYSL
A_23_P46690	0.0036817	0.103	2.16	up	TMEM81
A_24_P47547	0.0048569	0.115	2.16	up	RAN
A_33_P3489737	0.0053498	0.12	2.16	up	NLN
A_24_P218265	0.0067157	0.133	2.16	up	TNFRSF10B
A_24_P321411	0.0142913	0.185	2.16	up	PRKRIR
A_23_P40527	0.0178178	0.204	2.16	up	TBX1
A_23_P137931	0.0263898	0.241	2.16	up	ADORA3

A_24_P205213	0.0297011	0.254	2.16	up	ARSB
A_33_P3236993	0.0304105	0.257	2.16	up	ARVCF
A_23_P7101	0.0338969	0.269	2.16	up	SLBP
A_23_P93258	0.0443853	0.303	2.16	up	HIST1H3B
A_33_P3367392	0.0453988	0.305	2.16	up	FAM167B
A_33_P3337182	0.0095783	0.156	2.16	up	ENST00000439955
A_33_P3352562	0.0009093	0.063	2.17	up	C19orf28
A_33_P3418010	0.0000111	0.0158	2.17	up	NUP62
A_23_P156049	0.0003475	0.0444	2.17	up	HEXB
A_32_P12610	0.0005858	0.0552	2.17	up	E2F6
A_23_P9603	0.0015402	0.0754	2.17	up	PRKDC
A_23_P40049	0.0021023	0.0837	2.17	up	CAD
A_23_P143120	0.0024743	0.0883	2.17	up	ADAM17
A_33_P3336233	0.0036803	0.103	2.17	up	UBL4A
A_24_P645765	0.0041862	0.11	2.17	up	KLHDC5
A_24_P210637	0.0052004	0.118	2.17	up	CHST14
A_23_P84929	0.0053026	0.12	2.17	up	SLC38A5
A_23_P422193	0.0081036	0.145	2.17	up	SUV39H1
A_24_P193592	0.0127777	0.176	2.17	up	CCNF
A_23_P69738	0.0128724	0.176	2.17	up	RASL11B
A_24_P289299	0.0247172	0.235	2.17	up	ARHGEF25
A_23_P334218	0.0266578	0.243	2.17	up	WDR67
A_23_P138881	0.0382312	0.283	2.17	up	ACTN3
A_33_P3691168	0.0429691	0.299	2.17	up	IL31RA
A_23_P87545	0.0455924	0.306	2.17	up	IFITM3
A_33_P3282434	0.0017489	0.0779	2.18	up	SLC16A1
A_23_P31315	0.0029981	0.0942	2.18	up	CBX3
A_24_P327815	0.003502	0.101	2.18	up	STIP1
A_33_P3240249	0.0049197	0.116	2.18	up	LOC100129125
A_33_P3213204	0.0055338	0.122	2.18	up	MAGEF1
A_24_P592012	0.0132039	0.179	2.18	up	ZBTB46
A_23_P73837	0.0175559	0.202	2.18	up	TLR8
A_23_P344531	0.0267325	0.243	2.18	up	SYNPO
A_23_P368779	0.032151	0.263	2.18	up	ZNF114
A_23_P99930	0.0416899	0.295	2.18	up	TIPIN
A_23_P336554	0.0481658	0.314	2.18	up	IL1RAP
A_32_P514599	0.0000733	0.0284	2.18	up	THC2537477
A_21_P0000609	0.0330627	0.266	2.18	up	TRPA1
A_23_P33433	0.0010749	0.0666	2.19	up	MAZ
A_32_P192823	0.001724	0.0777	2.19	up	PRPS1L1
A_32_P190049	0.002453	0.088	2.19	up	LRRC58
A_23_P102842	0.00319	0.0972	2.19	up	NFS1
A_24_P282237	0.0043357	0.111	2.19	up	PRIM2
A_23_P206059	0.0059111	0.125	2.19	up	PRC1

A_33_P3284951	0.0067527	0.133	2.19	up	MCM5
A_23_P316501	0.0174112	0.202	2.19	up	NKAIN2
A_23_P49862	0.0190356	0.21	2.19	up	TRPV3
A_23_P53467	0.0230489	0.228	2.19	up	IKBIP
A_23_P37983	0.0275697	0.246	2.19	up	MT1B
A_33_P3382137	0.0283617	0.249	2.19	up	LOC728671
A_33_P3382538	0.0339611	0.27	2.19	up	JRKL
A_24_P557479	0.0345401	0.272	2.19	up	XAF1
A_23_P12241	0.0454202	0.305	2.19	up	MCOLN3
A_24_P280029	0.0000816	0.0294	2.2	up	PDXP
A_33_P3671378	0.0003736	0.0456	2.2	up	CERCAM
A_23_P3368	0.0004694	0.0502	2.2	up	FURIN
A_23_P144622	0.0009994	0.0651	2.2	up	GNPDA1
A_33_P3304516	0.001366	0.0722	2.2	up	ZNFX1
A_33_P3401556	0.0219791	0.223	2.2	up	CTLA4
A_32_P175739	0.0279534	0.248	2.2	up	HK2
A_23_P309381	0.0337629	0.269	2.2	up	HIST2H2AA4
A_24_P345209	0.0364303	0.278	2.2	up	DYRK3
A_23_P127533	0.0411476	0.293	2.2	up	DCUN1D5
A_23_P378450	0.0440322	0.302	2.2	up	MBD3L2
A_33_P3220376	0.0017014	0.0773	2.2	up	THC2591311
A_24_P68585	0.0002178	0.0384	2.21	up	SHC1
A_23_P334021	0.0006569	0.057	2.21	up	IGF2R
A_23_P253571	0.0012213	0.0691	2.21	up	PHF5A
A_23_P104109	0.0023445	0.0864	2.21	up	RPS6KC1
A_23_P46215	0.0038404	0.105	2.21	up	TRIM46
A_23_P141636	0.0045165	0.113	2.21	up	EIF4A3
A_33_P3413671	0.0072521	0.137	2.21	up	ABL2
A_33_P3329769	0.0111163	0.165	2.21	up	BEND6
A_33_P3414912	0.0215757	0.222	2.21	up	NPL
A_23_P401380	0.0228621	0.228	2.21	up	DIP2B
A_23_P63459	0.0363149	0.278	2.21	up	C1orf31
A_32_P68504	0.0034116	0.0998	2.22	up	ZDBF2
A_33_P3386760	0.0096057	0.156	2.22	up	CHEK2
A_33_P3281695	0.0132436	0.179	2.22	up	NLRP3
A_24_P80776	0.0179257	0.204	2.22	up	C6orf228
A_23_P386478	0.0309133	0.259	2.22	up	TNIP3
A_23_P207940	0.0316842	0.262	2.22	up	AK091525
A_24_P181585	0.0000889	0.0302	2.23	up	LRRC59
A_24_P271527	0.00024	0.0395	2.23	up	JOSD1
A_23_P27515	0.0008185	0.0601	2.23	up	PLD3
A_33_P3259393	0.003507	0.101	2.23	up	HAPLN3
A_23_P26557	0.0040408	0.108	2.23	up	C16orf59
A_23_P25176	0.010635	0.163	2.23	up	TBX5

A_23_P142205	0.029195	0.252	2.23	up	LILRA2
A_23_P209735	0.0317324	0.262	2.23	up	ARMC9
A_23_P80839	0.0490262	0.316	2.23	up	MAP6D1
A_33_P3312549	0.0212436	0.22	2.23	up	THC2616877
A_23_P109895	0.0000124	0.0158	2.24	up	SLC26A6
A_23_P200199	0.0009084	0.063	2.24	up	TXNDC12
A_23_P161615	0.0009566	0.0642	2.24	up	POLA2
A_23_P120103	0.0011948	0.0687	2.24	up	KCNS3
A_23_P252462	0.0041677	0.109	2.24	up	GALNT9
A_23_P69249	0.0047629	0.114	2.24	up	ACTL6A
A_23_P53476	0.0089388	0.151	2.24	up	LDHB
A_23_P48676	0.0134757	0.18	2.24	up	PYGL
A_24_P357169	0.0173609	0.201	2.24	up	EPPK1
A_23_P99163	0.028515	0.249	2.24	up	DRAM1
A_33_P3832857	0.0404343	0.291	2.24	up	HAUS2
A_33_P3340040	0.0413035	0.294	2.24	up	GINS4
A_23_P217498	0.0459707	0.307	2.24	up	GDPD2
A_23_P89824	0.0152721	0.191	2.25	up	C18orf45
A_23_P62659	0.0001247	0.0329	2.25	up	PPT1
A_33_P3418516	0.0010457	0.066	2.25	up	E2F3
A_33_P3211520	0.0014847	0.0746	2.25	up	SNAP47
A_24_P376707	0.0017675	0.0782	2.25	up	HDGF
A_32_P204381	0.0061015	0.127	2.25	up	CIAPIN1
A_32_P83845	0.008836	0.15	2.25	up	HEY1
A_32_P116556	0.0177881	0.204	2.25	up	ZNF469
A_23_P361448	0.0209465	0.219	2.25	up	SESN3
A_23_P41942	0.0213346	0.22	2.25	up	POLR3G
A_23_P47967	0.0317574	0.262	2.25	up	LHX5
A_24_P702813	0.0445936	0.303	2.25	up	XPR1
A_23_P20480	0.0010066	0.0654	2.26	up	BRF2
A_33_P3254634	0.0017643	0.0782	2.26	up	PDIA5
A_24_P191067	0.0023236	0.086	2.26	up	CLSTN1
A_23_P252681	0.0030809	0.0955	2.26	up	PCYT1A
A_33_P3334404	0.0030924	0.0956	2.26	up	CA6
A_23_P28169	0.0079203	0.143	2.26	up	ARL6IP6
A_32_P104478	0.0170606	0.2	2.26	up	FGD6
A_24_P36944	0.0238404	0.231	2.26	up	CEP170
A_33_P3489675	0.0372763	0.28	2.26	up	ARHGAP8
A_24_P12401	0.0419481	0.295	2.26	up	VEGFA
A_33_P3237760	0.0042477	0.11	2.26	up	THC2552980
A_32_P133244	0.0000633	0.0267	2.27	up	PARN
A_23_P166248	0.0044165	0.112	2.27	up	RCAN1
A_33_P3212112	0.0090374	0.151	2.27	up	SLC12A6
A_23_P211007	0.0105875	0.162	2.27	up	NRIP1

A_33_P3880302	0.0155055	0.192	2.27	up	EPHB2
A_23_P364613	0.018651	0.208	2.27	up	HOGA1
A_32_P101031	0.019691	0.212	2.27	up	LYPD1
A_33_P3242388	0.0216416	0.222	2.27	up	PIGX
A_23_P406702	0.0229201	0.228	2.27	up	CAMSAP2
A_23_P99642	0.0241804	0.233	2.27	up	SLC7A7
A_33_P3267356	0.0451671	0.305	2.27	up	GLS
A_21_P0010536	0.0035288	0.101	2.27	up	GBP3
A_21_P0000161	0.0165362	0.198	2.27	up	C16orf57
A_23_P346421	0.0001671	0.0364	2.28	up	ZNF532
A_23_P63010	0.0014363	0.0736	2.28	up	CERS2
A_33_P3232193	0.0036653	0.103	2.28	up	CDH11
A_33_P3248903	0.0042029	0.11	2.28	up	WNT7B
A_23_P104318	0.0083037	0.146	2.28	up	DDIT4
A_23_P67367	0.0096806	0.156	2.28	up	DHDH
A_23_P145965	0.0127043	0.175	2.28	up	TPST1
A_24_P192805	0.0175177	0.202	2.28	up	CARD17
A_24_P339071	0.0362512	0.277	2.28	up	CDR2
A_33_P3262292	0.0001368	0.0344	2.29	up	UBXN7
A_23_P37191	0.0001593	0.0362	2.29	up	PSMB5
A_23_P119789	0.0012306	0.0692	2.29	up	TMEM185B
A_33_P3328653	0.0021498	0.084	2.29	up	CELSR1
A_23_P124486	0.0045571	0.113	2.29	up	PTPN9
A_23_P131723	0.0073569	0.138	2.29	up	YWHAQ
A_33_P3214501	0.0127327	0.175	2.29	up	AEN
A_32_P150891	0.0129867	0.177	2.29	up	DIAPH3
A_23_P151267	0.0411913	0.293	2.29	up	LIMA1
A_23_P5370	0.046162	0.308	2.29	up	RPRM
A_23_P361419	0.0473554	0.311	2.29	up	DEPDC1B
A_33_P3229672	0.0156829	0.193	2.29	up	THC2651904
A_23_P90612	0.001384	0.0725	2.3	up	MCM6
A_33_P3276918	0.0014525	0.0739	2.3	up	FAM64A
A_23_P158096	0.002506	0.0886	2.3	up	COL27A1
A_23_P120467	0.0025098	0.0886	2.3	up	ZFP64
A_24_P335305	0.0029797	0.094	2.3	up	OAS3
A_32_P155247	0.0146011	0.187	2.3	up	FTL
A_33_P3369317	0.0447643	0.304	2.3	up	DNAJB5
A_33_P3256660	0.0009081	0.063	2.3	up	THC2591158
A_21_P0011613	0.0245843	0.234	2.3	up	DNAH17
A_33_P3301010	0.0010224	0.0656	2.31	up	SPIRE1
A_33_P3217238	0.002656	0.0902	2.31	up	ATAD2
A_24_P187948	0.0030746	0.0954	2.31	up	BID
A_24_P346855	0.0136076	0.181	2.31	up	MKI67
A_23_P76386	0.0170487	0.2	2.31	up	SLC6A12

A_33_P3224105	0.0173427	0.201	2.31	up	C15orf23
A_23_P132845	0.0229375	0.228	2.31	up	CLCN2
A_23_P169909	0.0244516	0.234	2.31	up	DGKI
A_21_P0012311	0.0069093	0.134	2.31	up	CELSR1
A_33_P3236122	0.0182029	0.205	2.31	up	ENST00000425104
A_33_P3298387	0.0019611	0.0813	2.32	up	PLK1
A_23_P14473	0.0028227	0.092	2.32	up	CNIH
A_24_P143138	0.0070169	0.135	2.32	up	FGD1
A_33_P3214586	0.0073428	0.138	2.32	up	ARTN
A_33_P3263232	0.0120352	0.171	2.32	up	LRRC3
A_23_P344451	0.0246163	0.234	2.32	up	HDGFRP3
A_33_P3258056	0.0433241	0.3	2.32	up	PALM3
A_23_P143952	0.0450139	0.304	2.32	up	CEP97
A_33_P3404989	0.0455258	0.305	2.32	up	HIST1H3H
A_24_P911676	0.0005984	0.0555	2.33	up	SOX4
A_33_P3366987	0.0025921	0.0893	2.33	up	GPR125
A_23_P158596	0.0025973	0.0893	2.33	up	AGTRAP
A_24_P221883	0.0043434	0.111	2.33	up	PARL
A_33_P3662553	0.0073174	0.138	2.33	up	YWHAQ
A_24_P217572	0.0076367	0.141	2.33	up	EDNRA
A_23_P487	0.0094355	0.154	2.33	up	UCK2
A_24_P160401	0.0165109	0.198	2.33	up	CDCP1
A_23_P121533	0.0356545	0.275	2.33	up	SPON2
A_33_P3389133	0.0008037	0.0596	2.34	up	MINPP1
A_23_P23575	0.0008416	0.0607	2.34	up	SLC39A1
A_24_P305570	0.0011314	0.0673	2.34	up	RIN2
A_33_P3315320	0.0019342	0.0807	2.34	up	CNTD1
A_33_P3363245	0.0053659	0.12	2.34	up	NXPH4
A_23_P50504	0.0109688	0.164	2.34	up	FTL
A_23_P209430	0.0150687	0.189	2.34	up	ALS2
A_23_P120227	0.0190079	0.209	2.34	up	LBH
A_33_P3351955	0.0349073	0.273	2.34	up	EGFR
A_23_P200310	0.0406886	0.292	2.34	up	DEPDC1
A_21_P0010626	0.0271019	0.244	2.34	up	PSAT1
A_23_P129486	0.0019205	0.0806	2.35	up	SEPX1
A_33_P3775007	0.000134	0.0339	2.35	up	ASAP1
A_23_P218892	0.0013383	0.0714	2.35	up	EIF4G1
A_23_P91590	0.0016552	0.0766	2.35	up	RANBP1
A_33_P3377364	0.0034884	0.1	2.35	up	ITGB4
A_23_P58009	0.0051709	0.118	2.35	up	C3orf52
A_24_P261417	0.0061854	0.128	2.35	up	DKK3
A_33_P3251896	0.0063887	0.13	2.35	up	APBB2
A_33_P3260146	0.0123899	0.173	2.35	up	C9orf3
A_33_P3278211	0.0126915	0.175	2.35	up	MMRN2

A_33_P3210146	0.0274362	0.246	2.35	up	PRR9
A_33_P3271657	0.0283502	0.249	2.35	up	HHIPL1
A_24_P345002	0.0338701	0.269	2.35	up	NUDT11
A_33_P3267865	0.0113785	0.166	2.35	up	NP111779
A_33_P3346348	0.0012727	0.07	2.36	up	WDR67
A_33_P3319760	0.0017249	0.0777	2.36	up	TAGLN2
A_33_P3329597	0.0020529	0.083	2.36	up	BLOC1S3
A_33_P3353692	0.002438	0.0879	2.36	up	MYH9
A_23_P75786	0.0061069	0.127	2.36	up	SLC15A3
A_33_P3394105	0.0077717	0.142	2.36	up	SPATA2L
A_32_P171061	0.0081287	0.145	2.36	up	ASCL2
A_24_P277673	0.0112605	0.166	2.36	up	HIST1H4G
A_32_P16258	0.0219889	0.223	2.36	up	EXOC6B
A_23_P153524	0.0408819	0.292	2.36	up	C19orf73
A_24_P10226	0.0475451	0.312	2.36	up	SEMA6D
A_33_P3224660	0.0001717	0.0364	2.37	up	ABCD1
A_23_P110076	0.002434	0.0878	2.37	up	WDR53
A_23_P28120	0.0108717	0.164	2.37	up	SIX2
A_23_P6066	0.0110376	0.165	2.37	up	CPXM1
A_23_P70794	0.0112928	0.166	2.37	up	RAB23
A_24_P128163	0.0238554	0.231	2.37	up	ADAMTS4
A_23_P50108	0.0269314	0.244	2.37	up	NDC80
A_33_P3287879	0.0302019	0.256	2.37	up	HIST1H3H
A_33_P3220837	0.0452624	0.305	2.37	up	MAFB
A_32_P132477	0.0017651	0.0782	2.37	up	THC2539563
A_21_P0011816	0.0048848	0.115	2.37	up	IGSF3
A_33_P3373469	0.0005903	0.0553	2.38	up	PSMD2
A_32_P175539	0.0033245	0.099	2.38	up	RCN2
A_23_P34700	0.0085428	0.148	2.38	up	TNNT2
A_33_P3422897	0.010947	0.164	2.38	up	ABCA1
A_23_P10081	0.0242749	0.233	2.38	up	ARL13B
A_23_P7684	0.0373453	0.281	2.38	up	CCNJL
A_33_P3772937	0.0400235	0.289	2.38	up	KRT8P12
A_23_P152804	0.000307	0.0417	2.39	up	NME1
A_23_P334664	0.0011301	0.0673	2.39	up	PML
A_23_P7679	0.0013914	0.0727	2.39	up	NUP155
A_23_P18196	0.0014992	0.075	2.39	up	RFC4
A_23_P416112	0.0021331	0.0839	2.39	up	RNF168
A_23_P54622	0.0023952	0.0875	2.39	up	KIF22
A_33_P3260575	0.0031671	0.0969	2.39	up	CERCAM
A_23_P37676	0.0043541	0.111	2.39	up	GPR176
A_23_P75741	0.005833	0.125	2.39	up	UBE2L6
A_24_P48898	0.0073457	0.138	2.39	up	APOL2
A_24_P16913	0.0135298	0.18	2.39	up	ABCC4

A_23_P13740	0.0169379	0.199	2.39	up	NAV3
A_23_P65757	0.0181793	0.205	2.39	up	CCNB2
A_33_P3316800	0.0204294	0.216	2.39	up	AHR
A_24_P317762	0.0101929	0.16	2.4	up	LY6E
A_33_P3307197	0.0165006	0.198	2.4	up	PTGFRN
A_23_P50000	0.0208504	0.218	2.4	up	FAM57A
A_33_P3338634	0.0248399	0.235	2.4	up	SYT14
A_23_P353717	0.0262704	0.241	2.4	up	RMI2
A_23_P115805	0.0342198	0.27	2.4	up	SYCE1
A_19_P00324604	0.0217825	0.222	2.4	up	C17orf51
A_23_P167040	0.0003695	0.0453	2.41	up	PDIA5
A_24_P102880	0.0006376	0.0568	2.41	up	NAV1
A_23_P135164	0.0010374	0.0659	2.41	up	UAP1L1
A_23_P141894	0.0011644	0.0678	2.41	up	PVR
A_23_P17870	0.0013429	0.0715	2.41	up	TMEM184B
A_32_P92783	0.0015221	0.0754	2.41	up	STIP1
A_23_P340722	0.0002179	0.0384	2.42	up	XPOT
A_23_P137143	0.0039955	0.107	2.42	up	DKC1
A_23_P78871	0.0045885	0.113	2.42	up	KCTD15
A_23_P70398	0.0063578	0.13	2.42	up	VEGFA
A_24_P161018	0.0079811	0.143	2.42	up	PARP14
A_23_P80040	0.014692	0.187	2.42	up	PROCR
A_23_P302116	0.0254477	0.238	2.42	up	PPFIA1
A_23_P36985	0.034916	0.273	2.42	up	PCDH8
A_23_P208182	0.0383859	0.284	2.42	up	SIGLEC10
A_32_P54475	0.000634	0.0568	2.42	up	CU676483
A_33_P3407925	0.0065009	0.131	2.43	up	VMP1
A_33_P3337026	0.0096205	0.156	2.43	up	SLC6A8
A_24_P235429	0.0192507	0.21	2.43	up	ABCA1
A_23_P122863	0.0326103	0.265	2.43	up	GRB10
A_33_P3213645	0.0336141	0.269	2.43	up	ERN2
A_23_P132784	0.0015657	0.0757	2.44	up	FXR1
A_23_P141021	0.0056898	0.123	2.44	up	LPCAT2
A_23_P106602	0.0156897	0.193	2.44	up	CRISPLD2
A_23_P258136	0.0159537	0.195	2.44	up	MXRA5
A_23_P34744	0.0203203	0.216	2.44	up	CTSK
A_24_P162373	0.023929	0.232	2.44	up	ZNRF3
A_23_P133408	0.043965	0.302	2.44	up	CSF2
A_19_P00319591	0.0362485	0.277	2.44	up	BC034319
A_23_P343411	0.0007199	0.0585	2.45	up	AGRN
A_23_P107587	0.0008354	0.0607	2.45	up	NPC1
A_33_P3267186	0.0015741	0.0758	2.45	up	ATP2B1
A_23_P128706	0.0020884	0.0836	2.45	up	DYNC1H1
A_23_P104199	0.002554	0.0891	2.45	up	ITGB1

A_33_P3258223	0.0066475	0.132	2.45	up	MCM7
A_23_P39799	0.0141063	0.184	2.45	up	LOXL3
A_23_P30799	0.0266974	0.243	2.45	up	HIST1H3F
A_33_P3379962	0.0274833	0.246	2.45	up	HLA-A
A_23_P115872	0.0283071	0.249	2.45	up	CEP55
A_24_P321511	0.0336914	0.269	2.45	up	GOLT1B
A_23_P112026	0.0446539	0.303	2.45	up	IDO1
A_23_P138725	0.0003012	0.0416	2.46	up	MARVELD1
A_24_P418408	0.0009218	0.0632	2.46	up	FAM89A
A_24_P305764	0.0039834	0.107	2.46	up	SMS
A_24_P324314	0.0058983	0.125	2.46	up	MXRA7
A_23_P21706	0.0070629	0.135	2.46	up	CTPS
A_23_P215956	0.0107981	0.163	2.46	up	MYC
A_24_P69095	0.0146675	0.187	2.46	up	ENC1
A_23_P379794	0.0152672	0.191	2.46	up	PIGW
A_23_P135499	0.0155072	0.192	2.46	up	CLIC4
A_33_P3255384	0.016306	0.197	2.46	up	BPIFC
A_33_P3847514	0.0222582	0.225	2.46	up	C6orf141
A_23_P55649	0.0334483	0.268	2.46	up	FPR2
A_23_P251043	0.033799	0.269	2.46	up	SYNDIG1
A_33_P3364268	0.0341553	0.27	2.46	up	LBH
A_33_P3379967	0.0363634	0.278	2.46	up	HLA-F
A_23_P97141	0.0415175	0.294	2.46	up	RGS1
A_23_P152055	0.001271	0.07	2.47	up	EFTUD1
A_23_P319617	0.0031113	0.0958	2.47	up	CHST7
A_33_P3284557	0.0067048	0.132	2.47	up	ZAK
A_23_P80940	0.0106413	0.163	2.47	up	PPAT
A_23_P7827	0.0444158	0.303	2.47	up	FAM26F
A_33_P3276784	0.0000737	0.0284	2.47	up	ENST00000454671
A_23_P366366	0.0002313	0.0392	2.48	up	SCRN1
A_33_P3242458	0.0019141	0.0805	2.48	up	SLC41A3
A_23_P148556	0.0025493	0.0891	2.48	up	ABCD1
A_32_P24585	0.0039005	0.106	2.48	up	SH3PXD2B
A_24_P353794	0.0110854	0.165	2.48	up	GALNT2
A_23_P48029	0.0153466	0.191	2.48	up	CLEC4A
A_23_P94030	0.0164011	0.197	2.48	up	LAMB1
A_23_P40718	0.0204967	0.217	2.48	up	PARVB
A_24_P311926	0.0352012	0.274	2.48	up	HLA-G
A_23_P202104	0.0364672	0.278	2.48	up	PPIF
A_23_P40295	0.0085616	0.148	2.49	up	C20orf103
A_24_P276102	0.0022657	0.0858	2.49	up	RBL1
A_33_P3339375	0.0037851	0.104	2.49	up	ARHGAP11B
A_33_P3389306	0.0284273	0.249	2.49	up	CU692123
A_33_P3248108	0.000187	0.0372	2.5	up	ZNF618

A_24_P130962	0.003324	0.099	2.5	up	TOR3A
A_23_P97990	0.0115822	0.168	2.5	up	HTRA1
A_33_P3236297	0.0162262	0.196	2.5	up	SLCO1A2
A_23_P23346	0.0178549	0.204	2.5	up	MLLT11
A_33_P3289596	0.0353517	0.274	2.5	up	EFR3B
A_23_P334709	0.0007012	0.0579	2.51	up	FKBP9
A_23_P99927	0.0036109	0.102	2.51	up	PTPLAD1
A_24_P133488	0.01805	0.205	2.51	up	CDCA4
A_24_P122746	0.0255878	0.238	2.51	up	VWA1
A_32_P103291	0.032733	0.265	2.51	up	SMYD3
A_33_P3401981	0.0337181	0.269	2.51	up	SPRED3
A_23_P69452	0.0030171	0.0945	2.52	up	DBR1
A_23_P210763	0.0041495	0.109	2.52	up	JAG1
A_23_P130787	0.0093904	0.154	2.52	up	QPCTL
A_33_P3258346	0.0171185	0.2	2.52	up	XAF1
A_23_P124417	0.0195508	0.211	2.52	up	BUB1
A_23_P411806	0.0000518	0.025	2.53	up	SLC44A1
A_24_P391526	0.0005483	0.0534	2.53	up	MAGED1
A_24_P268123	0.0012196	0.0691	2.53	up	ST3GAL3
A_32_P151800	0.0013741	0.0723	2.53	up	FAM72D
A_24_P191312	0.0028965	0.0931	2.53	up	SLC1A4
A_23_P57588	0.0032312	0.0979	2.53	up	GTSE1
A_24_P17719	0.0049078	0.116	2.53	up	KLHL5
A_23_P394986	0.0070408	0.135	2.53	up	CREG2
A_33_P3225512	0.0252623	0.237	2.53	up	OAS2
A_21_P0010834	0.0000751	0.0285	2.53	up	ZNF532
A_24_P276932	0.0010645	0.0663	2.54	up	ATP6V1C2
A_23_P93690	0.0043321	0.111	2.54	up	MCM7
A_23_P120316	0.006925	0.134	2.54	up	MTHFD2
A_23_P206612	0.008187	0.145	2.54	up	USP31
A_23_P156289	0.0117568	0.169	2.54	up	OSMR
A_23_P200138	0.0118753	0.17	2.54	up	SLAMF8
A_23_P365817	0.0146866	0.187	2.54	up	PPP1R14B
A_23_P309837	0.0149634	0.189	2.54	up	STON2
A_23_P19226	0.0154366	0.191	2.54	up	DSE
A_24_P399888	0.0164269	0.197	2.54	up	CENPM
A_24_P38276	0.0189794	0.209	2.54	up	FZD1
A_33_P3339212	0.019444	0.211	2.54	up	TRIP13
A_24_P107941	0.0386275	0.285	2.54	up	C16orf57
A_33_P3393135	0.0371348	0.28	2.54	up	ENST00000358739
A_23_P89509	0.0004551	0.0493	2.55	up	SPAG5
A_33_P3296499	0.0005097	0.0519	2.55	up	PTPRK
A_33_P3384108	0.0017965	0.0787	2.55	up	SLC19A1
A_23_P57760	0.002446	0.0879	2.55	up	ACPL2

A_24_P391574	0.008	0.144	2.55	up	LDLRAD3
A_23_P19712	0.00867	0.149	2.55	up	GMNN
A_24_P109417	0.0115643	0.168	2.55	up	C1orf187
A_24_P131522	0.0118941	0.17	2.55	up	ANTXR1
A_23_P20196	0.0135293	0.18	2.55	up	ARPC1B
A_23_P159974	0.024464	0.234	2.55	up	KLHL13
A_33_P3303305	0.0350279	0.273	2.55	up	DSCAM
A_23_P139725	0.008809	0.15	2.55	up	ENST00000407384
A_24_P333857	0.001124	0.0672	2.56	up	SGIP1
A_23_P50907	0.0014714	0.0744	2.56	up	ITGAV
A_23_P303455	0.0032436	0.0982	2.56	up	GPR161
A_33_P3288805	0.0064084	0.13	2.56	up	KLC2
A_33_P3260669	0.0186673	0.208	2.56	up	STK3
A_24_P202558	0.0428285	0.298	2.56	up	SIPA1L3
A_33_P3233906	0.0456262	0.306	2.56	up	RAMP1
A_33_P3286988	0.0279756	0.248	2.56	up	BF106382
A_33_P3387300	0.0003942	0.0461	2.57	up	FXR1
A_23_P57370	0.0004334	0.0481	2.57	up	CECR5
A_32_P124746	0.0006344	0.0568	2.57	up	RHEB
A_23_P141362	0.0013163	0.0709	2.57	up	FZD2
A_23_P40782	0.001544	0.0755	2.57	up	COMMD2
A_24_P303480	0.0018126	0.079	2.57	up	RAB32
A_23_P68922	0.0045306	0.113	2.57	up	MICALL1
A_32_P206949	0.0048726	0.115	2.57	up	TMEM17
A_24_P925062	0.0202821	0.215	2.57	up	MXRA7
A_24_P221414	0.020509	0.217	2.57	up	DYNC1I1
A_23_P60259	0.0408123	0.292	2.57	up	TMEM38B
A_23_P151634	0.0002013	0.0374	2.58	up	SUPT16H
A_23_P119344	0.0006593	0.057	2.58	up	TEAD2
A_24_P149645	0.0009917	0.0651	2.58	up	PUF60
A_33_P3296479	0.0016689	0.0766	2.58	up	APP
A_23_P207400	0.0062228	0.128	2.58	up	BRCA1
A_32_P206698	0.006467	0.131	2.58	up	CKS1B
A_23_P21033	0.0089971	0.151	2.58	up	GMPS
A_33_P3261982	0.0096845	0.156	2.58	up	PRIM2
A_23_P385861	0.023777	0.231	2.58	up	CDCA2
A_23_P137935	0.0278772	0.247	2.58	up	MNDA
A_32_P62997	0.0346366	0.272	2.58	up	PBK
A_24_P137376	0.0001145	0.0326	2.59	up	ATP2C1
A_23_P92441	0.000617	0.0564	2.59	up	MAD2L1
A_33_P3372666	0.0008	0.0595	2.59	up	PDGFA
A_23_P67151	0.0044053	0.112	2.59	up	OLFM2
A_23_P55421	0.0067044	0.132	2.59	up	CBX8
A_23_P68007	0.0084614	0.148	2.59	up	ATP1B3

A_23_P58321	0.0273988	0.245	2.59	up	CCNA2
A_23_P163099	0.0368764	0.279	2.59	up	POLE2
A_33_P3408599	0.0005098	0.0519	2.59	up	AK126604
A_23_P207600	0.0009645	0.0644	2.6	up	PSMD11
A_24_P269062	0.0039221	0.106	2.6	up	SPRY4
A_23_P24716	0.0064397	0.131	2.6	up	TMEM132A
A_24_P89457	0.0182072	0.205	2.6	up	CDKN1A
A_21_P0014003	0.0030818	0.0955	2.6	up	LOC100652766
A_33_P3342365	0.0159922	0.195	2.6	up	BX114156
A_33_P3300733	0.0473962	0.311	2.6	up	DB514319
A_23_P209183	0.0000676	0.0279	2.61	up	GLT25D1
A_23_P107421	0.0001253	0.0329	2.61	up	TK1
A_24_P226278	0.0003401	0.0439	2.61	up	PHF15
A_33_P3231277	0.0016991	0.0773	2.61	up	HIF1A
A_24_P2648	0.0029132	0.0932	2.61	up	PTPN14
A_24_P810697	0.0036232	0.102	2.61	up	MXRA7
A_23_P311358	0.0166847	0.198	2.61	up	ZNF282
A_33_P3377750	0.047681	0.312	2.61	up	KLC3
A_21_P0013501	0.0028123	0.0919	2.61	up	CDK5
A_23_P43800	0.0002329	0.0392	2.62	up	BOP1
A_23_P144549	0.0002764	0.0405	2.62	up	IBSP
A_33_P3298861	0.0019798	0.0816	2.62	up	SLC12A8
A_33_P3217213	0.0088121	0.15	2.62	up	PDLIM7
A_24_P223124	0.0293089	0.253	2.62	up	FNDC3B
A_21_P0012337	0.0047248	0.114	2.62	up	IGSF3
A_23_P103070	0.0001697	0.0364	2.63	up	YWHAH
A_33_P3405966	0.0006941	0.0579	2.63	up	ST3GAL3
A_24_P210675	0.0013338	0.0714	2.63	up	NDE1
A_32_P34589	0.0032075	0.0975	2.63	up	RSRC1
A_23_P88522	0.0083301	0.146	2.63	up	NMB
A_32_P213831	0.0149662	0.189	2.63	up	FAM40B
A_23_P140373	0.0171438	0.2	2.63	up	FLVCR2
A_23_P390032	0.0181073	0.205	2.63	up	SLC35G1
A_23_P253752	0.0408053	0.292	2.63	up	FAM54A
A_21_P0000022	0.0159684	0.195	2.63	up	BRI3
A_23_P132277	0.0023962	0.0875	2.64	up	MCM5
A_23_P167276	0.011235	0.165	2.64	up	PAQR3
A_33_P3392192	0.0151507	0.19	2.64	up	NRG1
A_21_P0013088	0.0024074	0.0875	2.64	up	SMS
A_33_P3214199	0.0001255	0.0329	2.65	up	ZNF532
A_23_P43898	0.0004041	0.0467	2.65	up	EPHX4
A_24_P129417	0.0009944	0.0651	2.65	up	BMP1
A_33_P3211727	0.0092783	0.153	2.65	up	FAM176B
A_23_P311087	0.0111529	0.165	2.65	up	ZNF281

A_24_P419120	0.0182145	0.205	2.65	up	BICD2
A_23_P74349	0.0239571	0.232	2.65	up	NUF2
A_33_P3281191	0.0283554	0.249	2.65	up	NID1
A_32_P95739	0.0005498	0.0534	2.66	up	TPI1
A_33_P3212994	0.0009756	0.0645	2.66	up	ZWINT
A_33_P3394933	0.0010967	0.0668	2.66	up	P4HA2
A_32_P142818	0.0046858	0.114	2.66	up	DLX1
A_24_P933908	0.0118798	0.17	2.66	up	GPNMB
A_33_P3345717	0.034167	0.27	2.66	up	DCX
A_24_P148717	0.0426595	0.298	2.66	up	CCR1
A_19_P00321671	0.000737	0.0585	2.66	up	ENST00000508021
A_19_P00319028	0.0106516	0.163	2.66	up	RFX8
A_33_P3232516	0.001546	0.0755	2.67	up	KIF18B
A_23_P141965	0.0073934	0.138	2.67	up	HAUS8
A_23_P345460	0.0113774	0.166	2.67	up	PLEKHG4
A_32_P92505	0.0145628	0.187	2.67	up	LCLAT1
A_33_P3807062	0.0158939	0.194	2.67	up	HJURP
A_23_P149892	0.0190026	0.209	2.67	up	CSGALNACT2
A_33_P3293446	0.0260323	0.24	2.67	up	KIAA1462
A_23_P51085	0.0282617	0.249	2.67	up	SPC25
A_23_P210109	0.0362167	0.277	2.67	up	CYP26B1
A_24_P76675	0.0447717	0.304	2.67	up	MFAP3L
A_24_P306892	0.0471688	0.311	2.67	up	B4GALNT3
A_24_P235049	0.0000723	0.0284	2.68	up	MTHFD1L
A_24_P205589	0.001305	0.0707	2.68	up	ACOT7
A_33_P3227041	0.0019789	0.0816	2.68	up	BID
A_23_P45917	0.0029648	0.0936	2.68	up	CKS1B
A_23_P425880	0.0038486	0.105	2.68	up	TRIO
A_23_P161624	0.0048549	0.115	2.68	up	FOSL1
A_33_P3325262	0.0100728	0.159	2.68	up	SLC6A8
A_23_P52311	0.0123044	0.173	2.68	up	TAF5
A_24_P65616	0.0001798	0.0369	2.69	up	PVR
A_23_P399501	0.0014498	0.0739	2.69	up	PKM2
A_24_P113144	0.0017149	0.0776	2.69	up	ATAD5
A_23_P113393	0.0086797	0.149	2.69	up	APLN
A_33_P3405424	0.0228267	0.227	2.69	up	IL4I1
A_33_P3326210	0.0294125	0.253	2.69	up	ESCO2
A_33_P3221438	0.0007979	0.0595	2.7	up	XXYLT1
A_33_P3249046	0.0034877	0.1	2.7	up	CLDN2
A_23_P46606	0.0056803	0.123	2.7	up	LPGAT1
A_23_P47682	0.0063265	0.129	2.7	up	NRIP3
A_24_P921366	0.0112166	0.165	2.7	up	CALD1
A_23_P165937	0.0126566	0.175	2.7	up	DSN1
A_33_P3433873	0.0135147	0.18	2.7	up	ZWILCH

A_23_P163481	0.0272648	0.245	2.7	up	BUB1B
A_33_P3271930	0.0282087	0.249	2.7	up	PYCR1
A_24_P100234	0.0000197	0.0191	2.71	up	MORC2
A_23_P13772	0.001065	0.0663	2.71	up	TBX3
A_33_P3789894	0.00114	0.0674	2.71	up	SNORA75
A_33_P3315190	0.0033223	0.099	2.71	up	MICAL2
A_23_P388146	0.003813	0.104	2.71	up	ZNF587
A_23_P25873	0.0064581	0.131	2.71	up	WDHD1
A_33_P3393311	0.0073807	0.138	2.71	up	MCHR1
A_23_P5339	0.0084917	0.148	2.71	up	TMEM177
A_33_P3401428	0.0000062	0.0158	2.72	up	TMEM38B
A_23_P120056	0.0011258	0.0672	2.72	up	RTKN
A_24_P265832	0.0033605	0.0994	2.72	up	SUCNR1
A_23_P141345	0.004586	0.113	2.72	up	MPP3
A_23_P19673	0.0070893	0.136	2.72	up	SGK1
A_23_P218827	0.0075707	0.14	2.72	up	POLQ
A_23_P84576	0.0127294	0.175	2.72	up	ANTXR1
A_23_P402331	0.0485872	0.315	2.72	up	WFDC5
A_23_P393727	0.0001675	0.0364	2.73	up	NRP2
A_33_P3381191	0.000513	0.052	2.73	up	FOXRED2
A_23_P132417	0.0019712	0.0814	2.73	up	LSG1
A_24_P269779	0.0027102	0.0908	2.73	up	CHST3
A_23_P13753	0.0241486	0.232	2.73	up	NFE2
A_23_P15146	0.0450987	0.305	2.73	up	IL32
A_24_P342312	0.0004416	0.0486	2.74	up	ODZ4
A_24_P237804	0.0077487	0.142	2.74	up	POTED
A_33_P3666817	0.0115477	0.168	2.74	up	C10orf55
A_33_P3242649	0.0265723	0.242	2.74	up	KIF18A
A_33_P3319041	0.0475871	0.312	2.74	up	HMGB3
A_23_P24997	0.0001605	0.0362	2.75	up	CDK4
A_33_P3222917	0.0001678	0.0364	2.75	up	CD276
A_23_P410625	0.0007117	0.0584	2.75	up	ZNF367
A_32_P194312	0.004779	0.115	2.75	up	SDK2
A_23_P146849	0.008403	0.147	2.75	up	APBA2
A_33_P3377691	0.0130795	0.178	2.75	up	C4orf46
A_23_P121527	0.0132575	0.179	2.75	up	KLHL5
A_33_P3270445	0.0132999	0.179	2.75	up	BAI2
A_23_P45799	0.0304772	0.257	2.75	up	ORC1
A_24_P320545	0.0016476	0.0766	2.76	up	PTK7
A_23_P100344	0.0100182	0.158	2.76	up	ORC6
A_23_P164196	0.036448	0.278	2.76	up	DLX4
A_33_P3248231	0.0000352	0.0221	2.77	up	LOC283028
A_33_P3265549	0.0001512	0.0355	2.77	up	SLC19A1
A_33_P3234124	0.0001861	0.0372	2.77	up	FAM132B

A_23_P55749	0.0020535	0.083	2.77	up	COL5A3
A_23_P85716	0.0077006	0.141	2.77	up	FCGR2A
A_24_P158385	0.0079683	0.143	2.78	up	ZMYND19
A_23_P202245	0.0141626	0.184	2.78	up	RET
A_33_P3422810	0.0198164	0.213	2.78	up	ULBP1
A_33_P3227375	0.043971	0.302	2.78	up	THBS2
A_23_P103919	0.0001117	0.0323	2.79	up	B4GALT3
A_23_P57667	0.0015088	0.075	2.79	up	PLXNA1
A_23_P32078	0.0108327	0.164	2.79	up	SLC28A3
A_23_P214168	0.0280003	0.248	2.79	up	COL12A1
A_33_P3256680	0.000127	0.033	2.8	up	MFHAS1
A_23_P109072	0.0004281	0.0481	2.8	up	SALL4
A_24_P112447	0.0007355	0.0585	2.8	up	ENTPD7
A_23_P40240	0.0014079	0.0728	2.8	up	CTSZ
A_23_P129569	0.0024127	0.0875	2.8	up	PALB2
A_24_P138784	0.0034767	0.1	2.8	up	MGAT5B
A_33_P3394689	0.0037083	0.103	2.8	up	SLC6A2
A_33_P3402526	0.0053956	0.121	2.8	up	CSGALNACT2
A_33_P3399870	0.0120239	0.171	2.8	up	CERS6
A_23_P51231	0.0178278	0.204	2.8	up	RUNX3
A_23_P90407	0.0183403	0.206	2.8	up	CASP14
A_23_P401606	0.0499003	0.318	2.8	up	EDIL3
A_33_P3380056	0.004578	0.113	2.81	up	C9orf30
A_33_P3306192	0.0009908	0.0651	2.81	up	KBTBD13
A_23_P35219	0.0020051	0.0822	2.81	up	NEK2
A_23_P66432	0.0028484	0.0923	2.81	up	TTYH2
A_23_P163087	0.0061876	0.128	2.81	up	NID2
A_23_P10194	0.0245542	0.234	2.81	up	SEZ6L2
A_32_P154830	0.0379729	0.283	2.81	up	OSTM1
A_23_P146512	0.0428313	0.298	2.81	up	GOLM1
A_33_P3225327	0.000002	0.0116	2.82	up	ATP13A3
A_23_P420348	0.0039704	0.107	2.82	up	POTED
A_23_P215461	0.0040382	0.108	2.82	up	LIMK1
A_23_P119778	0.0049774	0.116	2.82	up	SLC39A10
A_23_P333951	0.0056384	0.123	2.82	up	DNAH14
A_24_P406334	0.009266	0.153	2.82	up	STEAP1
A_33_P3404601	0.0133228	0.179	2.82	up	C2
A_33_P3376140	0.0166861	0.198	2.82	up	KCNJ15
A_23_P159721	0.0170042	0.2	2.82	up	GPR50
A_23_P252642	0.0002224	0.0388	2.83	up	BBS5
A_23_P122815	0.0006471	0.0568	2.83	up	CALU
A_24_P401787	0.0008948	0.0626	2.83	up	TPH2
A_23_P67980	0.0018982	0.0802	2.83	up	KLF7
A_33_P3390539	0.001934	0.0807	2.83	up	GYLTL1B

A_33_P3358469	0.0037421	0.103	2.83	up	GLI2
A_33_P3261293	0.0042169	0.11	2.83	up	DKK3
A_23_P416395	0.005416	0.121	2.83	up	STC2
A_23_P94422	0.026269	0.241	2.83	up	MELK
A_23_P122197	0.0322041	0.264	2.83	up	CCNB1
A_23_P204087	0.0449931	0.304	2.83	up	OAS2
A_33_P3335966	0.0480243	0.313	2.83	up	TPM1
A_33_P3420762	0.0006442	0.0568	2.83	up	THC2577776
A_24_P314571	0.0006231	0.0566	2.84	up	SPC24
A_23_P215265	0.0011054	0.0668	2.84	up	GNA12
A_23_P102731	0.0034111	0.0998	2.84	up	SMOX
A_33_P3255829	0.004883	0.115	2.84	up	MXRA5
A_23_P368225	0.0019126	0.0805	2.85	up	EME1
A_23_P211428	0.0028062	0.0918	2.85	up	SMTN
A_32_P30649	0.0034101	0.0998	2.85	up	ETV5
A_23_P103775	0.0121568	0.172	2.85	up	LRRC8C
A_33_P3316790	0.0132494	0.179	2.85	up	ADAMTS15
A_23_P17307	0.000004	0.0151	2.86	up	C20orf20
A_23_P168868	0.000686	0.0579	2.86	up	PTDSS1
A_23_P16384	0.0024457	0.0879	2.86	up	NLRP7
A_33_P3364089	0.0153227	0.191	2.86	up	SLCO1B3
A_33_P3334423	0.0233094	0.229	2.86	up	SPRR2G
A_24_P348203	0.0260913	0.24	2.86	up	LRRC8E
A_23_P23457	0.0000563	0.0256	2.87	up	FBLIM1
A_23_P63789	0.0048815	0.115	2.87	up	ZWINT
A_23_P252740	0.0114295	0.167	2.87	up	DSCC1
A_23_P49499	0.0171076	0.2	2.87	up	ST6GALNAC2
A_33_P3371889	0.0479087	0.313	2.87	up	NUAK1
A_23_P44684	0.0002456	0.0398	2.88	up	ECT2
A_23_P137532	0.000254	0.0403	2.88	up	PLOD1
A_33_P3302586	0.0021475	0.084	2.88	up	GPR153
A_33_P3276615	0.0026	0.0893	2.88	up	APOL4
A_23_P304356	0.0056863	0.123	2.88	up	CLEC5A
A_33_P3393766	0.0131772	0.179	2.88	up	C17orf96
A_23_P75516	0.0221204	0.224	2.88	up	PPFIA1
A_24_P33895	0.0299046	0.255	2.88	up	ATF3
A_23_P211699	0.0004463	0.0488	2.88	up	THC2477815
A_33_P3242659	0.0000308	0.0214	2.89	up	KIF13A
A_23_P153197	0.0065059	0.131	2.89	up	TGIF1
A_23_P79221	0.0145853	0.187	2.89	up	ACVR1
A_33_P3347320	0.0157425	0.193	2.89	up	LOC100133190
A_23_P27584	0.0204684	0.216	2.89	up	MYADM
A_23_P143906	0.0257401	0.239	2.89	up	MLF1
A_32_P16007	0.0332399	0.267	2.89	up	POTEB

A_33_P3244279	0.0015745	0.0758	2.89	up	AL582488
A_33_P3345314	0.000472	0.0502	2.9	up	ZFP64
A_24_P255218	0.002445	0.0879	2.9	up	MYO5A
A_33_P3215043	0.0044796	0.112	2.9	up	HEPHL1
A_33_P3423718	0.0048015	0.115	2.9	up	ADAMTS7
A_23_P88691	0.0425726	0.297	2.9	up	CHRNA5
A_33_P3296482	0.0012012	0.0687	2.91	up	PTPRK
A_23_P144896	0.0025932	0.0893	2.91	up	PDLIM7
A_24_P475349	0.0202957	0.215	2.91	up	RAB6B
A_23_P169017	0.0387282	0.285	2.91	up	DEFB103B
A_23_P142750	0.000465	0.0499	2.92	up	EIF2AK2
A_24_P288722	0.0022468	0.0856	2.92	up	CASK
A_23_P48669	0.0147108	0.187	2.92	up	CDKN3
A_24_P180680	0.000686	0.0579	2.93	up	LAPTM4B
A_23_P69179	0.0012616	0.0698	2.93	up	LEPREL1
A_23_P28598	0.0050041	0.116	2.93	up	DLX2
A_23_P408353	0.006869	0.134	2.93	up	HLA-A
A_24_P225679	0.0136612	0.181	2.93	up	IRS1
A_23_P138507	0.0231408	0.228	2.93	up	CDK1
A_23_P100127	0.0276304	0.246	2.93	up	CASC5
A_33_P3285456	0.0029244	0.0934	2.94	up	C1orf68
A_23_P36562	0.0092482	0.153	2.94	up	ITGA5
A_23_P140928	0.0154743	0.192	2.94	up	TMC7
A_24_P203689	0.0182348	0.206	2.94	up	KIF7
A_32_P70158	0.0274587	0.246	2.94	up	LILRB3
A_21_P0011815	0.0020632	0.0832	2.94	up	IGSF3
A_21_P0011967	0.0312179	0.26	2.94	up	SFRP4
A_33_P3413821	0.0007758	0.0591	2.95	up	KIRREL
A_23_P53276	0.0103338	0.16	2.95	up	TIMELESS
A_23_P107744	0.0253235	0.237	2.95	up	S1PR5
A_33_P3352712	0.0029556	0.0935	2.96	up	CDR2L
A_23_P214144	0.0042734	0.11	2.96	up	COL10A1
A_33_P3230548	0.0046057	0.113	2.96	up	KIF14
A_23_P68072	0.0177017	0.203	2.96	up	WDR54
A_23_P92499	0.0251324	0.237	2.96	up	TLR2
A_32_P37867	0.043455	0.3	2.96	up	KIAA1644
A_23_P111804	0.0010465	0.066	2.97	up	PARP12
A_23_P401	0.0123464	0.173	2.97	up	CENPF
A_23_P211504	0.0140416	0.183	2.97	up	KDELR3
A_23_P215790	0.0231308	0.228	2.97	up	EGFR
A_21_P0011751	0.0208127	0.218	2.98	up	CD177
A_23_P120860	0.0003951	0.0461	2.99	up	NIPSNAP1
A_23_P337875	0.0015522	0.0755	2.99	up	PAQR3
A_24_P276888	0.0020286	0.0826	2.99	up	CENPO

A_33_P3508822	0.0041815	0.11	2.99	up	APP
A_23_P370989	0.0052882	0.12	2.99	up	MCM4
A_33_P3296940	0.0062553	0.129	2.99	up	FNDC3B
A_33_P3556532	0.0387533	0.285	2.99	up	DNAH17
A_32_P54553	0.0015311	0.0754	3	up	USP41
A_23_P137366	0.0151359	0.19	3	up	C1QB
A_23_P68211	0.0032636	0.0983	3.01	up	SPR
A_33_P3423941	0.0322365	0.264	3.01	up	IFITM1
A_23_P403443	0.0384413	0.284	3.01	up	B4GALNT3
A_24_P191664	0.0001663	0.0364	3.02	up	GOLIM4
A_23_P205778	0.0046032	0.113	3.02	up	GNB5
A_24_P413126	0.0184746	0.207	3.02	up	PMEPA1
A_24_P944222	0.0002474	0.0399	3.03	up	TMTC3
A_23_P23303	0.0010366	0.0659	3.03	up	EXO1
A_23_P156310	0.0020657	0.0833	3.03	up	SKP2
A_23_P215341	0.0033941	0.0997	3.03	up	FKBP14
A_23_P388812	0.0099106	0.158	3.03	up	CKAP2L
A_23_P41470	0.0371125	0.28	3.03	up	DDX60
A_33_P3379939	0.0064823	0.131	3.04	up	HLA-F
A_33_P3306894	0.0075868	0.14	3.04	up	C11orf41
A_23_P324754	0.0097387	0.157	3.04	up	KIAA1199
A_33_P3406030	0.0229449	0.228	3.04	up	LRRC8C
A_33_P3314579	0.0001846	0.0372	3.05	up	RAB34
A_23_P115885	0.001875	0.0797	3.05	up	MINPP1
A_24_P411121	0.0115382	0.168	3.05	up	TNFRSF18
A_24_P918266	0.0003884	0.046	3.06	up	TMTC3
A_33_P3262665	0.0012945	0.0704	3.06	up	MAP7D3
A_23_P1552	0.0020179	0.0825	3.06	up	CTSC
A_33_P3763846	0.0052117	0.118	3.06	up	MAPK12
A_24_P126628	0.0077671	0.142	3.06	up	TMEM194A
A_33_P3354464	0.0089207	0.151	3.06	up	LOXL1
A_23_P101054	0.009843	0.157	3.06	up	KRT34
A_23_P39931	0.0250016	0.236	3.06	up	DYSF
A_33_P3422103	0.0004034	0.0467	3.06	up	THC2517032
A_33_P3360665	0.0006836	0.0579	3.07	up	ACVR1
A_24_P297078	0.0009939	0.0651	3.07	up	C20orf3
A_23_P134041	0.0074737	0.139	3.07	up	TBX18
A_23_P129075	0.0367616	0.279	3.07	up	WDR76
A_33_P3248787	0.0009474	0.0638	3.07	up	DB221055
A_33_P3407256	0.0013275	0.0712	3.08	up	TRIP13
A_23_P327519	0.0100196	0.158	3.08	up	STARD4
A_24_P208081	0.02964	0.254	3.09	up	PLA2G2F
A_33_P3219475	0.0018053	0.079	3.1	up	C9orf30-TMEFF1
A_33_P3408711	0.0019461	0.081	3.1	up	FLNA

A_23_P207537	0.0045497	0.113	3.1	up	DUSP14
A_33_P3415280	0.0295714	0.254	3.1	up	CABYR
A_23_P433855	0.0328914	0.266	3.1	up	RGS4
A_21_P0013306	0.0000217	0.0195	3.1	up	FKBP9
A_23_P165840	0.0059849	0.126	3.11	up	ODC1
A_24_P802145	0.0073978	0.138	3.11	up	IRS1
A_23_P159937	0.0146131	0.187	3.11	up	SLC6A8
A_33_P3358104	0.0323232	0.264	3.11	up	CD300E
A_24_P127691	0.0454234	0.305	3.11	up	DNAH14
A_23_P394448	0.0001208	0.0328	3.12	up	DPY19L1
A_23_P55020	0.0001888	0.0372	3.12	up	CD300LF
A_23_P209954	0.0027639	0.0915	3.12	up	GNLY
A_24_P227415	0.0120049	0.171	3.12	up	CLEC7A
A_33_P3348782	0.0232635	0.229	3.12	up	CYP2S1
A_24_P510377	0.0097005	0.156	3.12	up	BC031250
A_24_P276628	0.0000264	0.0206	3.13	up	PPT1
A_33_P3376116	0.0009912	0.0651	3.14	up	SPC24
A_23_P130182	0.0137428	0.181	3.14	up	AURKB
A_23_P88404	0.0006299	0.0567	3.15	up	TGFB3
A_23_P205031	0.0041322	0.109	3.15	up	COL4A2
A_33_P3289025	0.0339061	0.269	3.15	up	FAM176A
A_23_P106024	0.000285	0.0408	3.16	up	JAG2
A_23_P42353	0.0017478	0.0779	3.16	up	ETV7
A_24_P12413	0.009531	0.155	3.16	up	TRAM2
A_33_P3403576	0.0161454	0.196	3.16	up	FCGR2A
A_24_P184799	0.0245114	0.234	3.16	up	COCH
A_23_P420692	0.0404458	0.291	3.16	up	PPFIA4
A_23_P59388	0.0018615	0.0795	3.17	up	DST
A_23_P110196	0.0026039	0.0893	3.17	up	HERC5
A_23_P416434	0.0036067	0.102	3.17	up	PHF15
A_33_P3413168	0.0046731	0.114	3.17	up	COL27A1
A_23_P118174	0.0066235	0.132	3.17	up	PLK1
A_24_P65060	0.0245406	0.234	3.17	up	MEX3B
A_33_P3351120	0.0263716	0.241	3.17	up	TXNRD1
A_23_P66732	0.0047262	0.114	3.18	up	GSG2
A_23_P73097	0.0214777	0.221	3.18	up	RGS20
A_24_P81947	0.0008761	0.0621	3.19	up	CORO1C
A_23_P110802	0.0015886	0.0762	3.19	up	CENPH
A_23_P153571	0.0027304	0.0913	3.19	up	IGFL2
A_23_P78037	0.0094918	0.155	3.19	up	CCL7
A_24_P228130	0.0138219	0.182	3.19	up	CCL3L3
A_33_P3369401	0.0494753	0.317	3.19	up	CYP24A1
A_33_P3257330	0.0004544	0.0493	3.2	up	DCBLD1
A_23_P64611	0.0079227	0.143	3.2	up	P2RY6

A_23_P134454	0.0286496	0.25	3.2	up	CAV1
A_23_P133956	0.0319336	0.263	3.2	up	KIFC1
A_33_P3397198	0.0020712	0.0833	3.2	up	ENST00000455707
A_23_P45871	0.0264567	0.242	3.21	up	IFI44L
A_23_P148609	0.0327487	0.265	3.21	up	PLAC1
A_24_P57993	0.037791	0.282	3.21	up	KIAA1644
A_23_P126120	0.0113432	0.166	3.22	up	CENPL
A_24_P323598	0.013688	0.181	3.22	up	ESCO2
A_24_P156049	0.0000278	0.0207	3.23	up	SLC39A6
A_23_P156970	0.0084585	0.148	3.23	up	MEST
A_23_P139919	0.0234988	0.23	3.23	up	CHST11
A_23_P206018	0.0236289	0.231	3.23	up	TPM1
A_23_P345707	0.0353967	0.274	3.23	up	C15orf42
A_23_P28815	0.0086343	0.149	3.24	up	CYP24A1
A_23_P748	0.0160709	0.195	3.24	up	IRF6
A_33_P3297415	0.0001231	0.0329	3.25	up	NRP2
A_33_P3373364	0.0005269	0.0524	3.25	up	CLIC4
A_23_P113553	0.0247226	0.235	3.25	up	MAGEA11
A_33_P3224858	0.0010809	0.0667	3.26	up	EIF2AK2
A_24_P125469	0.0016887	0.0771	3.26	up	LIPG
A_24_P84396	0.0028045	0.0918	3.26	up	KIAA1199
A_33_P3298206	0.0256458	0.238	3.26	up	PPP4R4
A_32_P122754	0.0001186	0.0326	3.27	up	C9orf30
A_24_P108262	0.0016251	0.0766	3.27	up	SDK2
A_23_P104651	0.0081453	0.145	3.27	up	CDCA5
A_23_P380881	0.0012079	0.0688	3.28	up	ANKRD13B
A_23_P165608	0.00182	0.079	3.28	up	SEMA4F
A_23_P29773	0.0022556	0.0858	3.28	up	LAMP3
A_33_P3318581	0.0023284	0.0861	3.28	up	PLOD2
A_23_P127565	0.0107061	0.163	3.28	up	LAYN
A_24_P825874	0.001172	0.0681	3.29	up	POTEE
A_23_P113777	0.0446015	0.303	3.29	up	ITGBL1
A_23_P259189	0.0006414	0.0568	3.3	up	CLIC4
A_23_P354297	0.0049783	0.116	3.3	up	CHTF18
A_23_P80974	0.0366098	0.279	3.3	up	TDO2
A_33_P3421338	0.0027902	0.0918	3.31	up	KIF18B
A_33_P3243454	0.0073753	0.138	3.31	up	IGFL3
A_23_P7727	0.0389744	0.286	3.31	up	HAPLN1
A_24_P287941	0.0003856	0.046	3.32	up	PSMC3IP
A_23_P166459	0.0034733	0.1	3.32	up	LGALS1
A_23_P258190	0.0315916	0.261	3.32	up	AKR1B1
A_33_P3316273	0.0371225	0.28	3.32	up	CCL3
A_24_P137434	0.0009153	0.063	3.33	up	DCBLD2
A_23_P379614	0.0023651	0.0868	3.33	up	OIP5

A_23_P202004	0.0033212	0.099	3.33	up	PRTFDC1
A_24_P383609	0.0061959	0.128	3.34	up	NANOS1
A_23_P94412	0.0213329	0.22	3.34	up	PDCD1LG2
A_24_P406006	0.0276289	0.246	3.34	up	LPCAT1
A_33_P3366120	0.0012455	0.0695	3.35	up	FLNA
A_33_P3392391	0.0024022	0.0875	3.35	up	CPT1C
A_24_P383356	0.0042986	0.11	3.35	up	SLC39A14
A_23_P74290	0.0068178	0.133	3.35	up	GBP5
A_33_P3423949	0.005728	0.123	3.36	up	CBX2
A_33_P3279629	0.0000779	0.0289	3.37	up	UCN2
A_24_P319613	0.0078147	0.142	3.37	up	NEK2
A_23_P118246	0.0179574	0.205	3.37	up	GINS2
A_33_P3257678	0.0200738	0.214	3.37	up	HIST2H3A
A_24_P225616	0.0328882	0.266	3.37	up	RRM2
A_19_P00319398	0.020761	0.218	3.37	up	ERVMER34-1
A_32_P117464	0.0128548	0.176	3.38	up	MB21D2
A_23_P134100	0.0197743	0.213	3.38	up	ULBP3
A_33_P3221489	0.0004984	0.0515	3.39	up	KIRREL
A_23_P50990	0.0025707	0.0892	3.39	up	CENPO
A_33_P3232692	0.0282405	0.249	3.39	up	IL24
A_23_P45524	0.0002592	0.0403	3.4	up	NGFRAP1
A_24_P227831	0.0016683	0.0766	3.4	up	ABCC1
A_23_P109143	0.0266607	0.243	3.4	up	PRNP
A_24_P365506	0.0129711	0.177	3.41	up	FERMT1
A_23_P169039	0.000229	0.0392	3.42	up	SNAI2
A_24_P190168	0.0040788	0.108	3.42	up	TMEM97
A_23_P92727	0.0054659	0.121	3.42	up	RAI14
A_24_P253003	0.0126111	0.175	3.42	up	WNT11
A_24_P354689	0.0220377	0.223	3.42	up	SPOCK1
A_33_P3283944	0.032876	0.266	3.42	up	THC2497807
A_23_P59005	0.0030052	0.0942	3.44	up	TAP1
A_23_P210690	0.0040158	0.107	3.44	up	TRIB3
A_33_P3340025	0.0191161	0.21	3.44	up	GINS1
A_33_P3253596	0.0022952	0.0859	3.45	up	KIF4A
A_32_P55241	0.00312	0.0959	3.45	up	SHISA2
A_23_P126917	0.0010439	0.066	3.46	up	IGSF3
A_23_P68610	0.0008954	0.0626	3.47	up	TPX2
A_33_P3313283	0.0014169	0.0731	3.47	up	CILP2
A_23_P34142	0.000023	0.0195	3.48	up	WBP5
A_23_P77993	0.0006458	0.0568	3.48	up	C1QL1
A_23_P92261	0.0042857	0.11	3.48	up	ECE2
A_23_P218047	0.0318747	0.262	3.48	up	KRT5
A_23_P409462	0.0003689	0.0453	3.49	up	DCBLD1
A_23_P258410	0.0005122	0.052	3.49	up	WNT7A

A_33_P3402868	0.0349329	0.273	3.49	up	GRIN2D
A_33_P3405068	0.000024	0.0199	3.5	up	NAV1
A_23_P12147	0.0102341	0.16	3.5	up	C1orf74
A_23_P144959	0.0172369	0.201	3.5	up	VCAN
A_24_P96780	0.0048851	0.115	3.52	up	CENPF
A_33_P3231953	0.0156327	0.193	3.52	up	COL12A1
A_24_P166663	0.0187862	0.208	3.52	up	CDK6
A_33_P3257030	0.0000275	0.0207	3.54	up	LEPREL4
A_23_P331895	0.0001578	0.036	3.54	up	TTYH3
A_24_P51061	0.0002964	0.0414	3.54	up	DCBLD2
A_33_P3357658	0.0053087	0.12	3.54	up	HMGA2
A_23_P106362	0.0034204	0.0999	3.55	up	AQP9
A_23_P49155	0.0049413	0.116	3.55	up	CDH3
A_23_P43107	0.0001644	0.0364	3.56	up	TM7SF4
A_33_P3349536	0.0039058	0.106	3.56	up	CHEK1
A_23_P88731	0.0059625	0.126	3.56	up	RAD51
A_23_P259586	0.0133847	0.18	3.56	up	TTK
A_24_P42681	0.0000013	0.0112	3.57	up	PSMD2
A_33_P3224324	0.0078113	0.142	3.57	up	NOX4
A_33_P3370404	0.0012747	0.0701	3.58	up	PANX1
A_24_P169343	0.0219373	0.223	3.59	up	C9orf21
A_23_P69012	0.0000366	0.0226	3.59	up	CCR8
A_33_P3379326	0.0065332	0.131	3.59	up	MSR1
A_23_P60079	0.0076039	0.14	3.59	up	ANGPT2
A_32_P210202	0.0006011	0.0556	3.6	up	E2F7
A_23_P156687	0.0123891	0.173	3.6	up	CFB
A_23_P50919	0.0126639	0.175	3.6	up	SERPINE2
A_24_P50801	0.0000212	0.0194	3.61	up	NRP2
A_23_P65651	0.0007617	0.0586	3.61	up	WARS
A_24_P319113	0.0017996	0.0788	3.61	up	P2RX7
A_24_P304071	0.0041098	0.109	3.61	up	IFIT2
A_24_P365807	0.0026954	0.0905	3.62	up	EFNB1
A_23_P115482	0.0042642	0.11	3.62	up	UBE2T
A_23_P210726	0.0043023	0.11	3.62	up	CDC25B
A_32_P780862	0.0201385	0.215	3.62	up	BMP8B
A_23_P157865	0.0266378	0.242	3.62	up	TNC
A_33_P3402489	0.0013237	0.0712	3.63	up	OAS3
A_23_P397341	0.0054153	0.121	3.63	up	PAQR4
A_23_P376449	0.0007516	0.0585	3.64	up	THC2532340
A_23_P110052	0.0026077	0.0893	3.65	up	FOXL2
A_23_P73589	0.0015046	0.075	3.66	up	MSN
A_33_P3242952	0.0032939	0.0988	3.66	up	FAM72A
A_33_P3214670	0.016984	0.2	3.66	up	HK2
A_33_P3265376	0.0001191	0.0326	3.67	up	HOMER3

A_32_P75792	0.0084886	0.148	3.67	up	FAM132A
A_23_P29594	0.0106485	0.163	3.67	up	RPL39L
A_33_P3310649	0.0087637	0.15	3.67	up	ENST00000432413
A_23_P255104	0.0000718	0.0284	3.68	up	LHFPL2
A_24_P79054	0.0034047	0.0998	3.68	up	TGFB1
A_23_P35309	0.0069044	0.134	3.68	up	TAF5L
A_23_P94533	0.0011833	0.0684	3.69	up	CTSL1
A_33_P3374205	0.0016539	0.0766	3.7	up	MKI67
A_24_P237175	0.0021696	0.0841	3.7	up	CST2
A_23_P4679	0.0033064	0.0989	3.7	up	ERF
A_23_P51126	0.0175306	0.202	3.7	up	IL1RL1
A_23_P133236	0.0006593	0.057	3.71	up	PCDHB14
A_23_P212042	0.0047706	0.114	3.71	up	MFI2
A_33_P3258660	0.004938	0.116	3.71	up	SCD5
A_23_P141092	0.00664	0.132	3.71	up	TFAP4
A_23_P205489	0.0285089	0.249	3.71	up	SLC7A8
A_23_P117852	0.0112907	0.166	3.72	up	KIAA0101
A_21_P0012447	0.0390195	0.286	3.72	up	MYLK
A_24_P82106	0.0007	0.0579	3.73	up	MMP14
A_33_P3310301	0.0025579	0.0891	3.73	up	SLC7A8
A_32_P405759	0.0021629	0.084	3.74	up	COL22A1
A_23_P153745	0.0023167	0.086	3.74	up	IFI30
A_23_P62115	0.0318743	0.262	3.74	up	TIMP1
A_23_P98205	0.0086078	0.149	3.75	up	CHST1
A_23_P62377	0.0167118	0.199	3.76	up	PNCK
A_23_P52266	0.0120474	0.171	3.77	up	IFIT1
A_23_P105794	0.0163629	0.197	3.77	up	EPSTI1
A_23_P39465	0.0188363	0.209	3.77	up	BST2
A_33_P3332596	0.0219562	0.223	3.78	up	BX471465
A_23_P164596	0.0167866	0.199	3.79	up	SIGLEC12
A_33_P3266255	0.0021516	0.084	3.8	up	CSGALNACT2
A_33_P3308512	0.0037667	0.104	3.8	up	SLC16A10
A_33_P3390172	0.0035061	0.101	3.81	up	ADAMDEC1
A_32_P19716	0.0063565	0.13	3.81	up	ZNF697
A_24_P157926	0.0176286	0.203	3.81	up	TNFAIP3
A_24_P219552	0.0002143	0.0381	3.82	up	NFE2L1
A_23_P144337	0.0003917	0.0461	3.82	up	CCRN4L
A_32_P163089	0.005609	0.123	3.82	up	C12orf75
A_33_P3238166	0.0169078	0.199	3.82	up	PXDN
A_33_P3462856	0.0397822	0.289	3.82	up	AK023309
A_23_P209246	0.0002025	0.0375	3.83	up	GLI2
A_23_P63390	0.0118054	0.17	3.83	up	FCGR1B
A_33_P3286953	0.0167548	0.199	3.83	up	ADAMTS6
A_24_P304051	0.0000348	0.022	3.84	up	GSTO1

A_33_P3339650	0.0007388	0.0585	3.84	up	DST
A_24_P288890	0.0030298	0.0946	3.84	up	FAM101A
A_23_P132956	0.0349504	0.273	3.85	up	UCHL1
A_24_P84898	0.0018861	0.0799	3.86	up	FEN1
A_23_P67339	0.0025235	0.0887	3.86	up	RCN3
A_24_P385134	0.0072382	0.137	3.86	up	SCD5
A_33_P3287158	0.0172497	0.201	3.86	up	NLGN4X
A_24_P923251	0.0195369	0.211	3.86	up	TGM2
A_33_P3296230	0.0044982	0.112	3.87	up	ENST00000538113
A_23_P310	0.0032261	0.0979	3.88	up	MARCKSL1
A_23_P216579	0.0134081	0.18	3.88	up	PALM2
A_23_P256473	0.0172544	0.201	3.88	up	SEMA3C
A_23_P91829	0.000823	0.0603	3.89	up	DCBLD2
A_23_P94571	0.0391193	0.287	3.89	up	ELAVL2
A_23_P216429	0.0092682	0.153	3.9	up	ASPN
A_23_P45011	0.0120203	0.171	3.9	up	PPP1R14C
A_32_P34920	0.0005191	0.0521	3.91	up	FOXD1
A_23_P213584	0.0191248	0.21	3.91	up	HK3
A_33_P3389689	0.0010355	0.0659	3.92	up	LEPRE1
A_24_P290286	0.0094988	0.155	3.95	up	P4HA3
A_33_P3235147	0.037329	0.28	3.95	up	DLX5
A_23_P75811	0.0001738	0.0364	3.96	up	SLC3A2
A_23_P10542	0.0018848	0.0799	3.96	up	HTRA3
A_32_P62863	0.0026828	0.0905	3.96	up	SCHIP1
A_23_P153562	0.0084252	0.147	3.96	up	C5AR1
A_33_P3237977	0.0232208	0.229	3.96	up	LARP6
A_33_P3247042	0.003581	0.102	3.98	up	FPR3
A_23_P54055	0.0258025	0.239	3.98	up	AJUBA
A_33_P3349693	0.0000539	0.0253	3.99	up	LCLAT1
A_33_P3245575	0.0000064	0.0158	4.01	up	GLT25D1
A_23_P380857	0.0000627	0.0266	4.01	up	APOL4
A_33_P3243028	0.0010216	0.0656	4.01	up	DSPP
A_23_P353005	0.0039036	0.106	4.02	up	RNF217
A_23_P45365	0.0104681	0.161	4.02	up	COL4A5
A_23_P131935	0.0308557	0.259	4.02	up	FERMT1
A_33_P3243230	0.0367059	0.279	4.03	up	IL8
A_24_P414999	0.000033	0.0218	4.04	up	LAPTM4B
A_33_P3358824	0.000103	0.0307	4.04	up	PLOD1
A_24_P48248	0.0012736	0.07	4.04	up	C17orf53
A_23_P134419	0.0029661	0.0936	4.05	up	ZP3
A_23_P212617	0.001823	0.079	4.06	up	TFRC
A_33_P3391496	0.0033814	0.0996	4.06	up	SLIT3
A_23_P113701	0.0115515	0.168	4.06	up	PDGFA
A_24_P117410	0.0202911	0.215	4.08	up	KLHDC7B

A_24_P99175	0.0135557	0.18	4.09	up	ZDHHC22
A_23_P153320	0.0162008	0.196	4.09	up	ICAM1
A_23_P44421	0.0013909	0.0727	4.11	up	HTRA4
A_33_P3335177	0.0028072	0.0918	4.11	up	SFRP4
A_23_P25030	0.0114731	0.167	4.11	up	HSD17B6
A_23_P24129	0.0228807	0.228	4.11	up	DKK1
A_32_P300427	0.0417512	0.295	4.11	up	APCDD1L
A_23_P101093	0.0000955	0.0302	4.13	up	COPZ2
A_23_P321501	0.0005226	0.0522	4.13	up	DHRS2
A_23_P51397	0.0037405	0.103	4.13	up	ENAH
A_23_P352799	0.0046339	0.113	4.13	up	NPW
A_23_P501010	0.038447	0.284	4.13	up	COL17A1
A_33_P3285893	0.0007285	0.0585	4.14	up	SCRN1
A_33_P3269203	0.0024239	0.0877	4.14	up	SERPINH1
A_23_P370682	0.0027874	0.0918	4.16	up	BATF2
A_24_P98109	0.008716	0.149	4.16	up	SNX10
A_24_P81900	0.0366515	0.279	4.16	up	SLC2A3
A_33_P3527721	0.0023481	0.0865	4.18	up	LOC284219
A_23_P99063	0.0036575	0.103	4.18	up	LUM
A_23_P157299	0.0053005	0.12	4.18	up	AEBP1
A_23_P103310	0.0428427	0.299	4.19	up	S100A7
A_23_P338479	0.0001529	0.0355	4.2	up	CD274
A_23_P59950	0.0023861	0.0872	4.2	up	SLC39A14
A_33_P3293888	0.0416962	0.295	4.2	up	AFF2
A_23_P207456	0.0328734	0.266	4.24	up	CCL8
A_33_P3318661	0.0004173	0.0471	4.25	up	E2F7
A_23_P99292	0.0044821	0.112	4.25	up	RAD51AP1
A_23_P151506	0.0400145	0.289	4.25	up	PLEK2
A_23_P250156	0.0005389	0.0528	4.27	up	IGF2BP2
A_24_P277367	0.0362604	0.277	4.27	up	CXCL5
A_33_P3212456	0.0005108	0.0519	4.29	up	STX1A
A_33_P3213169	0.0142565	0.184	4.29	up	ITGAX
A_32_P195065	0.0025308	0.0888	4.33	up	SEMA4F
A_23_P126212	0.0003557	0.0447	4.34	up	CLSPN
A_33_P3289536	0.0021385	0.0839	4.35	up	SNX10
A_23_P56746	0.0070506	0.135	4.35	up	FAP
A_24_P413884	0.0066731	0.132	4.37	up	CENPA
A_33_P3344831	0.0387668	0.286	4.37	up	TMEM45A
A_33_P3395314	0.0071754	0.137	4.37	up	DB335107
A_23_P3681	0.0060449	0.126	4.39	up	NETO2
A_24_P12626	0.00909	0.152	4.39	up	CAV1
A_23_P417383	0.0284792	0.249	4.39	up	ASPRV1
A_24_P322354	0.0022082	0.0849	4.4	up	SKA1
A_23_P202881	0.0001912	0.0372	4.43	up	FEZ1

A_33_P3216059	0.0006568	0.057	4.44	up	ASPN
A_23_P393034	0.0013854	0.0726	4.44	up	HAS3
A_23_P148475	0.0020812	0.0834	4.44	up	KIF4A
A_33_P3311755	0.0027867	0.0918	4.44	up	KIF23
A_24_P236799	0.0001022	0.0307	4.46	up	RAB31
A_24_P216361	0.0117976	0.17	4.46	up	PRAME
A_23_P24104	0.0024171	0.0876	4.47	up	PLAU
A_24_P274270	0.0008661	0.0619	4.49	up	STAT1
A_23_P216355	0.0012616	0.0698	4.5	up	TONSL
A_23_P47148	0.0144672	0.186	4.5	up	NOX4
A_23_P165624	0.0045745	0.113	4.52	up	TNFAIP6
A_23_P210176	0.003619	0.102	4.53	up	ITGA6
A_32_P486693	0.0002311	0.0392	4.54	up	NRIP3
A_24_P411561	0.0017288	0.0778	4.55	up	HAVCR2
A_23_P134426	0.0023758	0.087	4.55	up	GPNMB
A_23_P118834	0.0028402	0.0923	4.56	up	TOP2A
A_33_P3264657	0.009855	0.157	4.57	up	CYP27C1
A_33_P3369158	0.0006551	0.057	4.6	up	KIF3C
A_23_P105957	0.0009267	0.0632	4.61	up	ACTN1
A_23_P80032	0.0020735	0.0833	4.62	up	E2F1
A_24_P250922	0.0057092	0.123	4.64	up	PTGS2
A_23_P159907	0.0002435	0.0396	4.65	up	MAGED4B
A_23_P10385	0.0054039	0.121	4.66	up	DTL
A_23_P15727	0.0016188	0.0765	4.67	up	FKBP10
A_33_P3267532	0.0030898	0.0956	4.68	up	KCNJ15
A_23_P139418	0.005343	0.12	4.68	up	GALNTL4
A_24_P55295	0.0015467	0.0755	4.69	up	GJA1
A_23_P14083	0.0372048	0.28	4.69	up	AMIGO2
A_23_P123071	0.0003082	0.0417	4.7	up	CAV2
A_23_P59261	0.0005193	0.0521	4.7	up	TPBG
A_24_P236091	0.0050837	0.117	4.71	up	ENO2
A_23_P150693	0.0065411	0.131	4.72	up	FJX1
A_24_P7143	0.0001605	0.0362	4.76	up	MYO1B
A_33_P3256920	0.001145	0.0675	4.77	up	WNT7B
A_23_P337934	0.0000112	0.0158	4.78	up	FBLIM1
A_24_P419132	0.0016694	0.0766	4.78	up	CENPI
A_23_P62890	0.0002219	0.0388	4.79	up	GBP1
A_23_P502336	0.0003202	0.0424	4.79	up	EMR2
A_33_P3268472	0.0008877	0.0625	4.79	up	CTSC
A_23_P257129	0.0067433	0.133	4.79	up	PAEP
A_23_P111995	0.0025782	0.0893	4.8	up	LOXL2
A_33_P3382565	0.000463	0.0498	4.81	up	KIF26B
A_32_P86763	0.0194898	0.211	4.82	up	TGM2
A_33_P3367396	0.0335638	0.268	4.83	up	FAM177B

A_33_P3237150	0.0085463	0.148	4.84	up	BMP2
A_33_P3271455	0.0141481	0.184	4.88	up	PXDN
A_33_P3391290	0.0002329	0.0392	4.89	up	C5orf13
A_33_P3357678	0.0004706	0.0502	4.9	up	LCTL
A_23_P126836	0.0020807	0.0834	4.9	up	TNFSF4
A_33_P3381513	0.0008931	0.0626	4.91	up	CD274
A_23_P141730	0.0012903	0.0703	4.94	up	DSG2
A_23_P303210	0.0056156	0.123	4.94	up	IKBIP
A_23_P151150	0.0007632	0.0586	4.95	up	FOXM1
A_33_P3411075	0.0017018	0.0773	4.95	up	FSCN1
A_23_P131866	0.0003005	0.0416	4.99	up	AURKA
A_21_P0011610	0.0020703	0.0833	4.99	up	DNAH17
A_23_P49338	0.0007641	0.0586	5	up	TNFRSF12A
A_23_P213562	0.0021052	0.0837	5.02	up	F2R
A_33_P3379396	0.0483462	0.314	5.05	up	KRT1
A_23_P139669	0.0152362	0.19	5.07	up	SLC2A3
A_23_P153480	0.0429805	0.299	5.09	up	KLK5
A_33_P3341299	0.005446	0.121	5.1	up	CACNG4
A_23_P359245	0.0024753	0.0883	5.11	up	MET
A_23_P92909	0.0194705	0.211	5.14	up	SPINK6
A_23_P201459	0.0011517	0.0676	5.15	up	IFI6
A_33_P3219601	0.0008183	0.0601	5.16	up	ABL2
A_24_P273756	0.0041741	0.109	5.16	up	TP63
A_24_P149124	0.0004068	0.0468	5.17	up	C5orf13
A_33_P3317603	0.0018384	0.0791	5.18	up	B4GALNT4
A_33_P3280845	0.0082514	0.146	5.18	up	THY1
A_23_P10206	0.0089928	0.151	5.18	up	HAS2
A_23_P43273	0.0000944	0.0302	5.24	up	EXT1
A_23_P40847	0.0002061	0.0376	5.26	up	CHST2
A_33_P3332547	0.00106	0.0663	5.26	up	IQCJ-SCHIP1
A_24_P911607	0.0074086	0.138	5.29	up	WNT7B
A_24_P44462	0.0023213	0.086	5.3	up	TPM1
A_23_P38696	0.0314867	0.261	5.31	up	DSC1
A_23_P21976	0.0195289	0.211	5.35	up	CSPG4
A_33_P3270776	0.001961	0.0813	5.36	up	HTRA3
A_23_P571	0.0109092	0.164	5.36	up	SLC2A1
A_23_P118815	0.0001166	0.0326	5.41	up	BIRC5
A_23_P69310	0.0007474	0.0585	5.41	up	CCRL2
A_23_P407840	0.0016096	0.0765	5.42	up	FNDC1
A_23_P44648	0.0000308	0.0214	5.43	up	ADAMTS12
A_32_P103633	0.0000421	0.0229	5.43	up	MCM2
A_33_P3265374	0.0000223	0.0195	5.45	up	HOMER3
A_33_P3284129	0.0001422	0.0349	5.46	up	LYPD1
A_24_P109214	0.0001039	0.0307	5.48	up	APOC1

A_33_P3355508	0.0008634	0.0619	5.5	up	FOXL2
A_23_P68436	0.0183596	0.206	5.5	up	WFDC12
A_33_P3237850	0.0017703	0.0782	5.5	up	ENST00000383038
A_23_P200728	0.0019813	0.0816	5.51	up	FCGR3A
A_21_P0000160	0.0019273	0.0807	5.54	up	TM4SF19
A_33_P3234222	0.0003586	0.0448	5.58	up	TSPO2
A_33_P3291831	0.0021493	0.084	5.59	up	CEP55
A_23_P419947	0.0039709	0.107	5.59	up	MLF1
A_24_P87036	0.0049126	0.116	5.65	up	ANO1
A_33_P3290780	0.0233934	0.229	5.67	up	IL24
A_23_P356684	0.0016404	0.0766	5.68	up	ANLN
A_23_P110531	0.0015831	0.0761	5.69	up	FST
A_24_P4170	0.0034668	0.1	5.69	up	CPA6
A_23_P85922	0.0017472	0.0779	5.74	up	BMP8A
A_24_P125335	0.0128986	0.176	5.78	up	CCL13
A_23_P55251	0.0011828	0.0684	5.79	up	ITGA3
A_23_P388168	0.0059662	0.126	5.79	up	RAB3B
A_33_P3409649	0.0004835	0.0507	5.81	up	ADAMTS2
A_23_P148255	0.0362924	0.277	5.83	up	MAGEA2B
A_23_P39364	0.0001023	0.0307	5.86	up	HOMER3
A_33_P3368895	0.0008212	0.0602	5.86	up	ENST00000427050
A_33_P3290354	0.0004706	0.0502	5.87	up	FNDC1
A_23_P253301	0.0037589	0.103	5.91	up	PFN2
A_23_P43197	0.0256528	0.238	5.97	up	CALB1
A_23_P34126	0.0031295	0.0961	5.99	up	BGN
A_24_P335620	0.0029512	0.0935	6.03	up	SLC7A5
A_23_P118571	0.0087574	0.15	6.04	up	SOST
A_23_P390700	0.0083226	0.146	6.06	up	CNTN1
A_24_P12397	0.000722	0.0585	6.15	up	TREM2
A_33_P3283044	0.000435	0.0482	6.22	up	CCDC165
A_33_P3304655	0.0015996	0.0764	6.27	up	LTBP1
A_23_P145096	0.000719	0.0585	6.31	up	PLA2G7
A_24_P931443	0.0134412	0.18	6.31	up	GPR68
A_33_P3806965	0.0026385	0.0898	6.4	up	FLJ13744
A_33_P3217393	0.0000103	0.0158	6.41	up	CD276
A_23_P1029	0.0000131	0.0158	6.42	up	MFAP2
A_23_P363275	0.011341	0.166	6.44	up	WDR66
A_33_P3391339	0.000059	0.0257	6.47	up	ENST00000383040
A_33_P3417640	0.0067848	0.133	6.56	up	KLK14
A_23_P22134	0.0098849	0.157	6.57	up	BNC1
A_23_P321307	0.0003024	0.0416	6.58	up	ADAMTS2
A_24_P192994	0.0028849	0.0931	6.63	up	FADS1
A_23_P56404	0.0002109	0.0379	6.65	up	EN1
A_33_P3420466	0.0002114	0.0379	6.65	up	MATN3

A_33_P3288832	0.0005236	0.0523	6.68	up	GPRIN1
A_23_P43810	0.0014477	0.0738	6.69	up	LTBP1
A_33_P3283611	0.0024621	0.0881	6.71	up	IFIT3
A_23_P77493	0.0123476	0.173	6.85	up	TUBB3
A_33_P3231447	0.0005015	0.0517	6.86	up	ITGA6
A_32_P60065	0.0008928	0.0626	7.07	up	F2RL2
A_33_P3210488	0.0008685	0.062	7.16	up	COL6A3
A_33_P3846177	0.0001513	0.0355	7.19	up	B4GALNT1
A_24_P923381	0.0000732	0.0284	7.21	up	L26245
A_23_P152305	0.0002789	0.0407	7.28	up	CDH11
A_33_P3286066	0.0002041	0.0376	7.34	up	PHLDB2
A_33_P3317005	0.0009598	0.0643	7.41	up	SLC11A1
A_33_P3244956	0.0001505	0.0355	7.48	up	NRIP3
A_23_P36397	0.0060144	0.126	7.64	up	CYP27B1
A_23_P819	0.0000517	0.025	7.66	up	ISG15
A_23_P360754	0.0029074	0.0932	7.69	up	ADAMTS4
A_24_P211565	0.0008119	0.06	7.73	up	C1QTNF6
A_23_P43164	0.0010424	0.066	7.73	up	SULF1
A_33_P3292886	0.0271791	0.245	7.78	up	KRT6A
A_23_P122216	0.0017469	0.0779	7.82	up	LOX
A_23_P150979	0.0043526	0.111	7.84	up	MUCL1
A_23_P64854	0.0178994	0.204	7.96	up	KRT75
A_33_P3221293	0.0046448	0.113	8.08	up	DFNA5
A_23_P256948	0.0002998	0.0415	8.19	up	MSC
A_23_P169097	0.0006274	0.0567	8.19	up	WISP1
A_23_P16469	0.0000514	0.025	8.22	up	PLAUR
A_24_P299474	0.0008258	0.0603	8.24	up	ODZ2
A_23_P217379	0.0026417	0.0899	8.26	up	COL4A6
A_33_P3411165	0.0033595	0.0994	8.35	up	MAGEA2B
A_23_P18452	0.0005342	0.0528	8.45	up	CXCL9
A_24_P277934	0.0001868	0.0372	8.63	up	COL1A2
A_23_P122924	0.0006135	0.0562	8.65	up	INHBA
A_23_P139786	0.0175935	0.203	8.8	up	OASL
A_23_P19333	0.0051271	0.117	8.84	up	TREM1
A_24_P179504	0.0031715	0.0969	8.85	up	WDR66
A_23_P19030	0.0000771	0.0287	8.97	up	ARSI
A_24_P299685	0.000157	0.0359	9.08	up	PDPN
A_24_P827037	0.0000412	0.0229	9.1	up	LRRC15
A_23_P89780	0.0046974	0.114	9.17	up	LAMA3
A_23_P144071	0.0000049	0.0151	9.21	up	COL7A1
A_23_P83818	0.000275	0.0403	9.3	up	COL5A1
A_23_P69030	0.0005689	0.0545	9.34	up	COL8A1
A_33_P3365735	0.0013002	0.0705	9.55	up	THBS2
A_33_P3284345	0.0001152	0.0326	9.69	up	NRG1

A_23_P65240	0.000863	0.0619	9.89	up	COL4A1
A_21_P0011633	0.0364401	0.278	10.16	up	KRT14
A_33_P3245218	0.0000649	0.0271	10.28	up	ODZ2
A_24_P280274	0.0098248	0.157	10.71	up	S100A7A
A_24_P28722	0.0009863	0.065	10.73	up	RSAD2
A_23_P156327	0.000091	0.0302	10.76	up	TGFBI
A_23_P315815	0.0006475	0.0568	10.87	up	NRG1
A_21_P0011517	0.0051887	0.118	10.95	up	KRT14
A_33_P3242973	0.0002967	0.0414	10.99	up	IGF2BP2
A_23_P137665	0.0014748	0.0744	11	up	CHI3L1
A_24_P935491	0.0001479	0.0355	11.11	up	COL3A1
A_23_P33196	0.0001011	0.0307	11.44	up	COL5A2
A_24_P158089	0.0003661	0.0453	11.44	up	SERPINE1
A_23_P342641	0.0027896	0.0918	11.48	up	SLC44A5
A_24_P334130	0.0010804	0.0667	12.06	up	FN1
A_23_P8906	0.0000023	0.0118	12.07	up	LRP12
A_23_P215634	0.0000388	0.0226	12.26	up	IGFBP3
A_23_P214026	0.0001094	0.032	13.13	up	FBN2
A_23_P95930	0.0007775	0.0592	13.16	up	HMGA2
A_33_P3338121	0.0001488	0.0355	13.2	up	LAMB3
A_24_P185945	0.008074	0.144	13.26	up	MAGEA4
A_23_P369899	0.000026	0.0205	13.27	up	TMEM158
A_33_P3306146	0.0001254	0.0329	14.11	up	PLAU
A_33_P3345534	0.0009248	0.0632	14.33	up	KRT14
A_24_P160466	0.0000031	0.0141	14.62	up	GPRIN1
A_23_P57417	0.0000086	0.0158	15.09	up	MMP11
A_23_P392942	0.0000762	0.0287	15.25	up	MSR1
A_33_P3398727	0.0000299	0.0214	15.27	up	ENST00000447898
A_23_P38537	0.0087483	0.15	15.66	up	KRT16
A_33_P3343175	0.0001696	0.0364	15.96	up	CXCL10
A_23_P161698	0.0044579	0.112	15.98	up	MMP3
A_23_P157793	0.0019518	0.081	16.19	up	CA9
A_23_P52761	0.0016442	0.0766	16.22	up	MMP7
A_23_P372946	0.0003796	0.0459	16.74	up	TM4SF19
A_23_P76249	0.0074028	0.138	17.02	up	KRT6B
A_23_P2271	0.0003201	0.0424	17.28	up	PTHLH
A_23_P150316	0.0013065	0.0707	18.06	up	MMP12
A_23_P112859	0.0000021	0.0116	19.26	up	CST1
A_33_P3511265	0.0000387	0.0226	19.43	up	POSTN
A_24_P882732	0.0002292	0.0392	20.38	up	ENST00000399211
A_23_P96158	0.0001099	0.032	21.36	up	KRT17
A_23_P160968	0.0003312	0.0434	22.77	up	LAMC2
A_24_P303091	0.0001111	0.0322	23.12	up	CXCL10
A_33_P3279119	0.0000141	0.0165	25.58	up	DR007930

A_33_P3304668	0.0000214	0.0194	26.3	up	COL1A1
A_23_P111888	0.0000179	0.0179	26.96	up	CTHRC1
A_23_P13094	0.0092572	0.153	27.31	up	MMP10
A_23_P125278	0.0003305	0.0434	27.37	up	CXCL11
A_23_P7313	0.0002631	0.0403	29.15	up	SPP1
A_23_P1691	0.0003702	0.0453	29.34	up	MMP1
