# Supplementary material for: Integrated Analysis of Long Noncoding RNA and mRNA Expression Profile in Advanced Laryngeal Squamous Cell Carcinoma
Source: PLoS One. 2016 Dec 29;11(12):e0169232. doi: 10.1371/journal.pone.0169232 (PMC5199101; doi:10.1371/journal.pone.0169232)
Supplement: S5 Table — (PDF) [file pone.0169232.s005.pdf]

miRNA	ceRNA	type	binding site
hsa-miR-128-3p	AF049885	noncoding	5
	AK096049	noncoding	1
	AX746517	noncoding	1
	AY562502	noncoding	1
	AY946017	noncoding	2
	BC033124	noncoding	1
	DA714598	noncoding	1
	ENST00000372173	noncoding	2
	ENST00000400178	noncoding	14
	ENST00000411775	noncoding	1
	ENST00000411795	noncoding	8
	ENST00000415330	noncoding	1
	ENST00000416395	noncoding	4
	ENST00000419640	noncoding	1
	ENST00000419952	noncoding	8
	ENST00000423764	noncoding	2
	ENST00000427168	noncoding	2
	ENST00000427491	noncoding	2
	ENST00000428669	noncoding	4
	ENST00000430699	noncoding	2
	ENST00000437249	noncoding	1
	ENST00000439302	noncoding	1
	ENST00000444754	noncoding	1
	ENST00000445438	noncoding	2
	ENST00000445461	noncoding	10
	ENST00000450314	noncoding	3
	ENST00000456280	noncoding	1
	ENST00000456327	noncoding	3
	ENST00000456342	noncoding	14
	ENST00000458377	noncoding	5
	ENST00000458468	noncoding	14
	ENST00000494791	noncoding	1
	ENST00000497440	noncoding	3
	ENST00000500162	noncoding	6
	ENST00000500955	noncoding	3
	ENST00000504820	noncoding	6
	ENST00000507072	noncoding	1
	ENST00000507508	noncoding	2
	ENST00000509491	noncoding	3
	ENST00000518605	noncoding	1
	ENST00000518902	noncoding	1
	ENST00000524346	noncoding	13
	ENST00000524517	noncoding	6
	ENST00000527543	noncoding	1
	ENST00000539229	noncoding	2
	ENST00000547824	noncoding	1

ENST00000552502	noncoding	1
ENST00000553682	noncoding	2
ENST00000553909	noncoding	1
ENST00000555864	noncoding	1
ENST00000557691	noncoding	2
ENST00000563477	noncoding	4
ENST00000565979	noncoding	2
ENST00000568091	noncoding	1
Hs.130180	noncoding	2
Hs.653095	noncoding	4
Hs.722974	noncoding	4
Hs.728856	noncoding	1
Hs.731140	noncoding	2
M61870	noncoding	3
M76743	noncoding	1
M97723	noncoding	3
NR_001543	noncoding	5
NR_002788	noncoding	3
NR_002806	noncoding	2
NR_002940_3	noncoding	2
NR_003191	noncoding	3
NR_003366	noncoding	3
NR_003662	noncoding	3
NR_015446	noncoding	2
NR_023388	noncoding	1
NR_024420	noncoding	2
NR_024441	noncoding	1
NR_024559	noncoding	6
NR_024602	noncoding	1
NR_026558	noncoding	2
NR_026677	noncoding	1
NR_026756	noncoding	2
NR_027270	noncoding	1
NR_027399	noncoding	4
NR_027469	noncoding	4
NR_027647	noncoding	2
NR_027663	noncoding	6
NR_027791	noncoding	10
NR_027995	noncoding	1
NR_028049	noncoding	1
NR_028080	noncoding	19
NR_028343	noncoding	1
NR_030732	noncoding	1
NR_033234	noncoding	7
NR_033701	noncoding	7
NR_033798	noncoding	4
NR_033815	noncoding	1

NR_033872	noncoding	9
NR_034112	noncoding	1
NR_036468	noncoding	3
NR_036489	noncoding	2
NR_036522	noncoding	1
NR_036627	noncoding	6
NR_036634	noncoding	15
NR_037853_5	noncoding	1
NR_037918	noncoding	7
NR_038225	noncoding	6
NR_038226	noncoding	6
NR_038433	noncoding	1
NR_038894	noncoding	2
NR_040091	noncoding	9
NR_040113	noncoding	2
NR_045012	noncoding	2
NR_045066	noncoding	4
NR_045128	noncoding	3
NR_045129	noncoding	2
NR_045211	noncoding	3
NR_045405	noncoding	4
NR_045563	noncoding	2
NR_047662	noncoding	6
NR_047680	noncoding	2
NR_047683	noncoding	2
NR_072979	noncoding	4
OTTHUMT00000047656	noncoding	1
OTTHUMT00000047661	noncoding	1
OTTHUMT00000157918	noncoding	2
OTTHUMT00000314912	noncoding	2
OTTHUMT00000323603	noncoding	1
OTTHUMT00000326022	noncoding	4
PIK3R1	coding	1
S68588	noncoding	1
S73490	noncoding	1
TCONS_00000692	noncoding	5
TCONS_00000879	noncoding	1
TCONS_00005789	noncoding	1
TCONS_00005921	noncoding	3
TCONS_00007856	noncoding	9
TCONS_00007857	noncoding	4
TCONS_00009804	noncoding	1
TCONS_00011242	noncoding	1
TCONS_00012852	noncoding	2
TCONS_00014788	noncoding	1
TCONS_00016964	noncoding	5
TCONS_00017606	noncoding	1

TCONS_00017615	noncoding	5
TCONS_00017649	noncoding	2
TCONS_00021496	noncoding	1
TCONS_00023459	noncoding	1
TCONS_00024633	noncoding	1
TCONS_00024652	noncoding	1
TCONS_00025471	noncoding	1
TCONS_00025714	noncoding	1
TCONS_00029196	noncoding	2
TCONS_00029359	noncoding	1
TCONS_l2_00000030	noncoding	1
TCONS_l2_00000550	noncoding	1
TCONS_l2_00000551	noncoding	1
TCONS_l2_00000563	noncoding	1
TCONS_l2_00001496	noncoding	1
TCONS_l2_00001497	noncoding	1
TCONS_l2_00001498	noncoding	1
TCONS_l2_00002156	noncoding	1
TCONS_l2_00002157	noncoding	2
TCONS_l2_00002159	noncoding	1
TCONS_l2_00002161	noncoding	1
TCONS_l2_00002491	noncoding	1
TCONS_l2_00002629	noncoding	1
TCONS_l2_00002637	noncoding	1
TCONS_l2_00002638	noncoding	1
TCONS_l2_00003113	noncoding	6
TCONS_l2_00003491	noncoding	2
TCONS_l2_00007044	noncoding	2
TCONS_l2_00007045	noncoding	2
TCONS_l2_00007081	noncoding	2
TCONS_l2_00009425	noncoding	1
TCONS_l2_00011784	noncoding	1
TCONS_l2_00012388	noncoding	2
TCONS_l2_00012876	noncoding	1
TCONS_l2_00013074	noncoding	2
TCONS_l2_00014470	noncoding	1
TCONS_l2_00015866	noncoding	1
TCONS_l2_00016247	noncoding	3
TCONS_l2_00016249	noncoding	2
TCONS_l2_00017132	noncoding	2
TCONS_l2_00017135	noncoding	2
TCONS_l2_00017137	noncoding	2
TCONS_l2_00017424	noncoding	1
TCONS_l2_00019716	noncoding	2
TCONS_l2_00022091	noncoding	1
TCONS_l2_00022661	noncoding	2
TCONS_l2_00022666	noncoding	1

	TCONS_l2_00022670	noncoding	1
	TCONS_l2_00028814	noncoding	1
	TCONS_l2_00029285	noncoding	1
	TCONS_l2_00029290	noncoding	1
	TCONS_l2_00029291	noncoding	1
	TCONS_l2_00029292	noncoding	1
	TCONS_l2_00029376	noncoding	3
	TCONS_l2_00029378	noncoding	2
	TCONS_l2_00029722	noncoding	1
	TCONS_l2_00029723	noncoding	1
	TCONS_l2_00029938	noncoding	1
	U52701	noncoding	1
	uc001cqo.1	noncoding	1
	uc001ejc.2	noncoding	1
	uc001hgr.2	noncoding	2
	uc001mmy.1	noncoding	1
	uc001zgs.1	noncoding	1
	uc002aeh.2	noncoding	3
	uc002ktb.2	noncoding	2
	uc002qng.2	noncoding	1
	uc002sth.1	noncoding	1
	uc002sux.1	noncoding	1
	uc002yjf.2	noncoding	2
	uc002yji.2	noncoding	1
	uc002ykc.2	noncoding	10
	uc003ehd.2	noncoding	3
	uc003ttk.1	noncoding	1
	uc004acq.3	noncoding	2
	uc010rpo.1	noncoding	1
	uc010tcj.1	noncoding	1
	XR_109954	noncoding	1
hsa-miR-181d-5p	AF049885	noncoding	8
	AF131786	noncoding	1
	AF429306	noncoding	6
	AK055738	noncoding	1
	AK096049	noncoding	2
	AK123264	noncoding	1
	AK126763	noncoding	1
	AL050227	noncoding	1
	AX746755	noncoding	1
	BC015458	noncoding	1
	CR608789	noncoding	1
	DA714598	noncoding	5
	DQ925687	noncoding	1
	ENST00000372173	noncoding	4
	ENST00000400178	noncoding	18
	ENST00000411775	noncoding	1

ENST00000411795	noncoding	2
ENST00000413004	noncoding	1
ENST00000413221	noncoding	1
ENST00000415121	noncoding	1
ENST00000416351	noncoding	1
ENST00000416395	noncoding	1
ENST00000419952	noncoding	11
ENST00000427168	noncoding	1
ENST00000428669	noncoding	5
ENST00000430699	noncoding	4
ENST00000437249	noncoding	1
ENST00000439302	noncoding	4
ENST00000442663	noncoding	1
ENST00000444754	noncoding	1
ENST00000445461	noncoding	15
ENST00000450314	noncoding	1
ENST00000456342	noncoding	18
ENST00000456587	noncoding	4
ENST00000458377	noncoding	1
ENST00000458468	noncoding	18
ENST00000490162	noncoding	1
ENST00000494791	noncoding	1
ENST00000497440	noncoding	6
ENST00000500162	noncoding	4
ENST00000500955	noncoding	2
ENST00000504820	noncoding	4
ENST00000505448	noncoding	1
ENST00000509491	noncoding	1
ENST00000517640	noncoding	1
ENST00000518902	noncoding	1
ENST00000523913	noncoding	1
ENST00000524346	noncoding	17
ENST00000524517	noncoding	4
ENST00000526206	noncoding	1
ENST00000553267	noncoding	1
ENST00000553909	noncoding	1
ENST00000557691	noncoding	1
ENST00000563477	noncoding	3
ENST00000565979	noncoding	4
Hs.653095	noncoding	2
Hs.722974	noncoding	2
Hs.728856	noncoding	3
Hs.731140	noncoding	2
Hs.98895	noncoding	4
M61870	noncoding	1
NR_001284	noncoding	1
NR_001284_5	noncoding	1

NR_001543	noncoding	3
NR_001544	noncoding	1
NR_002330	noncoding	1
NR_002788	noncoding	2
NR_002925	noncoding	1
NR_003191	noncoding	1
NR_003366	noncoding	8
NR_015446	noncoding	2
NR_024014	noncoding	1
NR_024420	noncoding	1
NR_024559	noncoding	6
NR_024602	noncoding	1
NR_026700	noncoding	1
NR_027270	noncoding	1
NR_027399	noncoding	2
NR_027469	noncoding	1
NR_027647	noncoding	2
NR_027663	noncoding	1
NR_027791	noncoding	15
NR_027995	noncoding	1
NR_028049	noncoding	1
NR_028080	noncoding	21
NR_028343	noncoding	6
NR_033234	noncoding	5
NR_033798	noncoding	1
NR_033807	noncoding	3
NR_033810	noncoding	3
NR_033811	noncoding	3
NR_033872	noncoding	4
NR_036489	noncoding	5
NR_036581	noncoding	1
NR_036627	noncoding	5
NR_036634	noncoding	8
NR_037918	noncoding	11
NR_038225	noncoding	3
NR_038226	noncoding	3
NR_040091	noncoding	6
NR_040113	noncoding	1
NR_045012	noncoding	3
NR_045066	noncoding	7
NR_045211	noncoding	1
NR_045405	noncoding	1
NR_045563	noncoding	16
NR_047662	noncoding	4
NR_047680	noncoding	4
NR_047683	noncoding	4
NR_072979	noncoding	2

OTTHUMT00000280614	noncoding	2
OTTHUMT00000326022	noncoding	2
PBX1	coding	3
TCONS_00000692	noncoding	2
TCONS_00000879	noncoding	1
TCONS_00002573	noncoding	1
TCONS_00002831	noncoding	2
TCONS_00003850	noncoding	1
TCONS_00005789	noncoding	1
TCONS_00005921	noncoding	2
TCONS_00006708	noncoding	1
TCONS_00007856	noncoding	6
TCONS_00007857	noncoding	4
TCONS_00009804	noncoding	1
TCONS_00012852	noncoding	2
TCONS_00016964	noncoding	3
TCONS_00017590	noncoding	1
TCONS_00017606	noncoding	4
TCONS_00017615	noncoding	5
TCONS_00017649	noncoding	3
TCONS_00020005	noncoding	2
TCONS_00020048	noncoding	1
TCONS_00022863	noncoding	2
TCONS_00022864	noncoding	2
TCONS_00023208	noncoding	2
TCONS_00023441	noncoding	1
TCONS_00024652	noncoding	1
TCONS_00026032	noncoding	1
TCONS_00027238	noncoding	1
TCONS_00028829	noncoding	2
TCONS_00029196	noncoding	1
TCONS_l2_00000550	noncoding	1
TCONS_l2_00000551	noncoding	1
TCONS_l2_00001496	noncoding	4
TCONS_l2_00001497	noncoding	3
TCONS_l2_00001498	noncoding	3
TCONS_l2_00002156	noncoding	4
TCONS_l2_00002157	noncoding	4
TCONS_l2_00002159	noncoding	2
TCONS_l2_00002161	noncoding	1
TCONS_l2_00002491	noncoding	2
TCONS_l2_00002636	noncoding	1
TCONS_l2_00002637	noncoding	3
TCONS_l2_00002638	noncoding	3
TCONS_l2_00003113	noncoding	3
TCONS_l2_00003491	noncoding	3
TCONS_l2_00009425	noncoding	3

	TCONS_l2_00010129	noncoding	2
	TCONS_l2_00011787	noncoding	1
	TCONS_l2_00012876	noncoding	2
	TCONS_l2_00013919	noncoding	1
	TCONS_l2_00015025	noncoding	6
	TCONS_l2_00016247	noncoding	2
	TCONS_l2_00017143	noncoding	1
	TCONS_l2_00017424	noncoding	1
	TCONS_l2_00017545	noncoding	4
	TCONS_l2_00017547	noncoding	3
	TCONS_l2_00017556	noncoding	3
	TCONS_l2_00017559	noncoding	1
	TCONS_l2_00019086	noncoding	1
	TCONS_l2_00022091	noncoding	1
	TCONS_l2_00022661	noncoding	1
	TCONS_l2_00024745	noncoding	1
	TCONS_l2_00028814	noncoding	2
	TCONS_l2_00029368	noncoding	2
	TCONS_l2_00029376	noncoding	2
	TGFBR2	coding	1
	uc001cqo.1	noncoding	2
	uc001ejc.2	noncoding	3
	uc001hgr.2	noncoding	3
	uc001zgs.1	noncoding	3
	uc002aeh.2	noncoding	2
	uc002sth.1	noncoding	1
	uc002wry.2	noncoding	1
	uc002yji.2	noncoding	1
	uc002ykc.2	noncoding	15
	uc003ehd.2	noncoding	3
	uc003ttk.1	noncoding	1
	uc009yzx.1	noncoding	1
	uc010rpo.1	noncoding	1
	uc010tcj.1	noncoding	1
	XR_109954	noncoding	1
	XR_110606	noncoding	1
hsa-miR-18a-3p	AF049885	noncoding	8
	AF202879	noncoding	1
	AF539739	noncoding	4
	AK056119	noncoding	1
	AK096049	noncoding	9
	AK124033	noncoding	1
	AX746755	noncoding	1
	AY294627	noncoding	1
	AY462278	noncoding	1
	AY495952	noncoding	1
	CR623805	noncoding	1

DA714598	noncoding	2
DQ925687	noncoding	1
ENST00000400178	noncoding	13
ENST00000407889	noncoding	1
ENST00000411759	noncoding	1
ENST00000411775	noncoding	1
ENST00000411795	noncoding	2
ENST00000416395	noncoding	3
ENST00000419640	noncoding	1
ENST00000419952	noncoding	8
ENST00000426185	noncoding	1
ENST00000427168	noncoding	4
ENST00000427491	noncoding	1
ENST00000428669	noncoding	5
ENST00000430699	noncoding	3
ENST00000437249	noncoding	1
ENST00000439302	noncoding	4
ENST00000442663	noncoding	1
ENST00000444754	noncoding	1
ENST00000445438	noncoding	2
ENST00000445461	noncoding	9
ENST00000447039	noncoding	1
ENST00000448927	noncoding	1
ENST00000449955	noncoding	1
ENST00000450314	noncoding	3
ENST00000453998	noncoding	3
ENST00000456327	noncoding	1
ENST00000456342	noncoding	13
ENST00000456587	noncoding	3
ENST00000458377	noncoding	4
ENST00000458468	noncoding	13
ENST00000478815	noncoding	1
ENST00000497440	noncoding	11
ENST00000500162	noncoding	10
ENST00000500955	noncoding	5
ENST00000505448	noncoding	1
ENST00000507508	noncoding	2
ENST00000509491	noncoding	4
ENST00000515455	noncoding	1
ENST00000518172	noncoding	1
ENST00000518605	noncoding	3
ENST00000518902	noncoding	3
ENST00000524346	noncoding	11
ENST00000524517	noncoding	8
ENST00000539229	noncoding	7
ENST00000555442	noncoding	1
ENST00000555864	noncoding	1

ENST00000557602	noncoding	1
ENST00000557691	noncoding	2
ENST00000563477	noncoding	4
ENST00000565979	noncoding	1
ENST00000566475	noncoding	1
ENST00000568091	noncoding	1
ENST00000568752	noncoding	1
ENST00000570223	noncoding	1
ENST00000573260	noncoding	3
EU039832	noncoding	1
Hs.130180	noncoding	5
Hs.653095	noncoding	5
Hs.722974	noncoding	4
Hs.728856	noncoding	2
Hs.731140	noncoding	1
Hs.98895	noncoding	1
M61870	noncoding	3
M76743	noncoding	1
M97723	noncoding	1
NR_001543	noncoding	7
NR_002788	noncoding	3
NR_002817	noncoding	2
NR_003191	noncoding	3
NR_003260	noncoding	3
NR_003366	noncoding	8
NR_003662	noncoding	1
NR_003948	noncoding	1
NR_024046	noncoding	1
NR_024420	noncoding	1
NR_024441	noncoding	2
NR_024559	noncoding	6
NR_024602	noncoding	2
NR_026558	noncoding	2
NR_026700	noncoding	1
NR_026756	noncoding	2
NR_026759	noncoding	3
NR_027270	noncoding	2
NR_027399	noncoding	6
NR_027469	noncoding	1
NR_027647	noncoding	2
NR_027663	noncoding	4
NR_027706	noncoding	1
NR_027791	noncoding	9
NR_027995	noncoding	1
NR_028049	noncoding	1
NR_028080	noncoding	12
NR_028343	noncoding	1

NR_028439	noncoding	1
NR_033234	noncoding	7
NR_033701	noncoding	4
NR_033798	noncoding	2
NR_033815	noncoding	3
NR_033828	noncoding	2
NR_033872	noncoding	10
NR_034112	noncoding	1
NR_036442	noncoding	1
NR_036468	noncoding	1
NR_036489	noncoding	1
NR_036522	noncoding	1
NR_036581	noncoding	2
NR_036627	noncoding	1
NR_036634	noncoding	20
NR_037853_5	noncoding	2
NR_037918	noncoding	13
NR_038225	noncoding	6
NR_038226	noncoding	6
NR_038433	noncoding	4
NR_038877	noncoding	1
NR_038894	noncoding	1
NR_040091	noncoding	5
NR_040113	noncoding	2
NR_045012	noncoding	1
NR_045066	noncoding	8
NR_045128	noncoding	1
NR_045129	noncoding	1
NR_045211	noncoding	3
NR_045405	noncoding	1
NR_045563	noncoding	12
NR_046422	noncoding	1
NR_047662	noncoding	10
NR_047680	noncoding	5
NR_047683	noncoding	5
NR_072979	noncoding	15
OTTHUMT00000157918	noncoding	3
OTTHUMT00000280614	noncoding	1
OTTHUMT00000314912	noncoding	3
OTTHUMT00000326022	noncoding	5
PPAP2B	coding	1
TCONS_00000692	noncoding	9
TCONS_00002831	noncoding	3
TCONS_00003850	noncoding	1
TCONS_00004435	noncoding	2
TCONS_00005921	noncoding	1
TCONS_00007856	noncoding	8

TCONS_00007857	noncoding	6
TCONS_00010517	noncoding	1
TCONS_00012852	noncoding	5
TCONS_00012947	noncoding	1
TCONS_00015993	noncoding	2
TCONS_00016964	noncoding	4
TCONS_00017606	noncoding	3
TCONS_00017649	noncoding	2
TCONS_00022475	noncoding	2
TCONS_00023287	noncoding	3
TCONS_00023441	noncoding	1
TCONS_00024633	noncoding	3
TCONS_00024652	noncoding	2
TCONS_00026032	noncoding	1
TCONS_00029196	noncoding	2
TCONS_00029986	noncoding	1
TCONS_00030010	noncoding	1
TCONS_00040336_HOT	noncoding	3
TCONS_l2_00000549	noncoding	1
TCONS_l2_00000563	noncoding	2
TCONS_l2_00001212	noncoding	1
TCONS_l2_00001496	noncoding	4
TCONS_l2_00001497	noncoding	3
TCONS_l2_00001498	noncoding	3
TCONS_l2_00002156	noncoding	3
TCONS_l2_00002157	noncoding	3
TCONS_l2_00002159	noncoding	3
TCONS_l2_00002161	noncoding	1
TCONS_l2_00002491	noncoding	2
TCONS_l2_00002629	noncoding	1
TCONS_l2_00002636	noncoding	1
TCONS_l2_00002637	noncoding	3
TCONS_l2_00002638	noncoding	3
TCONS_l2_00003113	noncoding	6
TCONS_l2_00003491	noncoding	2
TCONS_l2_00007044	noncoding	1
TCONS_l2_00007045	noncoding	1
TCONS_l2_00007081	noncoding	1
TCONS_l2_00010129	noncoding	1
TCONS_l2_00011787	noncoding	1
TCONS_l2_00011988	noncoding	2
TCONS_l2_00012563	noncoding	2
TCONS_l2_00012876	noncoding	1
TCONS_l2_00013919	noncoding	2
TCONS_l2_00014470	noncoding	3
TCONS_l2_00016247	noncoding	1
TCONS_l2_00017135	noncoding	2

	TCONS_l2_00017137	noncoding	4
	TCONS_l2_00017143	noncoding	1
	TCONS_l2_00017418	noncoding	2
	TCONS_l2_00017424	noncoding	2
	TCONS_l2_00019086	noncoding	1
	TCONS_l2_00019716	noncoding	2
	TCONS_l2_00022661	noncoding	3
	TCONS_l2_00028763	noncoding	1
	TCONS_l2_00028814	noncoding	6
	TCONS_l2_00029053	noncoding	1
	TCONS_l2_00029290	noncoding	1
	TCONS_l2_00029291	noncoding	1
	TCONS_l2_00029327	noncoding	1
	TCONS_l2_00029366	noncoding	1
	TCONS_l2_00029368	noncoding	2
	TCONS_l2_00029376	noncoding	2
	TCONS_l2_00029378	noncoding	3
	U52699	noncoding	1
	U52701	noncoding	1
	uc001cqo.1	noncoding	3
	uc001ejc.2	noncoding	1
	uc001hgr.2	noncoding	6
	uc002ktb.2	noncoding	2
	uc002qng.2	noncoding	1
	uc002sth.1	noncoding	2
	uc002yjf.2	noncoding	2
	uc002yji.2	noncoding	2
	uc002ykc.2	noncoding	9
	uc003ehd.2	noncoding	11
	uc004acq.3	noncoding	2
	uc010lrv.1	noncoding	1
	uc010rpo.1	noncoding	7
	uc010tcj.1	noncoding	1
	XR_110606	noncoding	1
hsa-miR-196a-5p	AF049885	noncoding	3
	AF429306	noncoding	1
	AK026822	noncoding	1
	AK056075	noncoding	1
	BC071777	noncoding	1
	CR627122	noncoding	1
	DA714598	noncoding	2
	ENST00000400178	noncoding	13
	ENST00000411795	noncoding	4
	ENST00000416395	noncoding	2
	ENST00000419640	noncoding	2
	ENST00000419952	noncoding	9
	ENST00000427168	noncoding	3

ENST00000436211	noncoding	1
ENST00000445438	noncoding	2
ENST00000445461	noncoding	9
ENST00000447039	noncoding	1
ENST00000448844	noncoding	1
ENST00000450314	noncoding	4
ENST00000456327	noncoding	1
ENST00000456342	noncoding	13
ENST00000456587	noncoding	5
ENST00000457658	noncoding	1
ENST00000458155	noncoding	1
ENST00000458468	noncoding	13
ENST00000490162	noncoding	2
ENST00000494791	noncoding	2
ENST00000497440	noncoding	5
ENST00000500162	noncoding	1
ENST00000504820	noncoding	2
ENST00000518605	noncoding	2
ENST00000524346	noncoding	11
ENST00000524517	noncoding	2
ENST00000533992	noncoding	1
ENST00000561977	noncoding	1
ENST00000563477	noncoding	2
ENST00000565979	noncoding	3
ENST00000573260	noncoding	1
Hs.130180	noncoding	2
Hs.520664	noncoding	1
Hs.570567	noncoding	1
Hs.59203	noncoding	1
Hs.653095	noncoding	2
Hs.712206	noncoding	1
Hs.98895	noncoding	2
M61867	noncoding	1
M61870	noncoding	1
M76743	noncoding	1
NR_001543	noncoding	2
NR_003191	noncoding	3
NR_003366	noncoding	4
NR_003662	noncoding	1
NR_015446	noncoding	5
NR_024559	noncoding	2
NR_026558	noncoding	1
NR_027270	noncoding	3
NR_027469	noncoding	3
NR_027647	noncoding	1
NR_027791	noncoding	9
NR_028049	noncoding	2

NR_028080	noncoding	22
NR_028343	noncoding	1
NR_033234	noncoding	2
NR_033872	noncoding	2
NR_036489	noncoding	3
NR_036581	noncoding	1
NR_036634	noncoding	6
NR_037918	noncoding	5
NR_038225	noncoding	2
NR_038226	noncoding	2
NR_038846	noncoding	1
NR_038877	noncoding	1
NR_040113	noncoding	1
NR_045012	noncoding	1
NR_045066	noncoding	12
NR_045211	noncoding	3
NR_045405	noncoding	1
NR_045563	noncoding	12
NR_047662	noncoding	1
NR_047680	noncoding	5
NR_047683	noncoding	5
NR_072979	noncoding	3
OTTHUMT00000314912	noncoding	1
OTTHUMT00000326022	noncoding	1
PBX1	coding	5
TCONS_00000692	noncoding	8
TCONS_00002831	noncoding	1
TCONS_00003850	noncoding	1
TCONS_00005778	noncoding	1
TCONS_00005790	noncoding	1
TCONS_00007856	noncoding	9
TCONS_00007857	noncoding	6
TCONS_00009804	noncoding	1
TCONS_00012852	noncoding	2
TCONS_00012947	noncoding	1
TCONS_00016964	noncoding	2
TCONS_00017606	noncoding	2
TCONS_00017649	noncoding	1
TCONS_00023208	noncoding	1
TCONS_00026032	noncoding	1
TCONS_l2_00000560	noncoding	1
TCONS_l2_00000562	noncoding	1
TCONS_l2_00000563	noncoding	2
TCONS_l2_00002162	noncoding	2
TCONS_l2_00002167	noncoding	1
TCONS_l2_00002168	noncoding	1
TCONS_l2_00002629	noncoding	2

	TCONS_l2_00002630	noncoding	1
	TCONS_l2_00002631	noncoding	1
	TCONS_l2_00002632	noncoding	1
	TCONS_l2_00003113	noncoding	2
	TCONS_l2_00007047	noncoding	2
	TCONS_l2_00009425	noncoding	1
	TCONS_l2_00011786	noncoding	3
	TCONS_l2_00012876	noncoding	1
	TCONS_l2_00015025	noncoding	2
	TCONS_l2_00016247	noncoding	1
	TCONS_l2_00016250	noncoding	1
	TCONS_l2_00017139	noncoding	3
	TCONS_l2_00017140	noncoding	2
	TCONS_l2_00017143	noncoding	2
	TCONS_l2_00017144	noncoding	1
	TCONS_l2_00017419	noncoding	5
	TCONS_l2_00017424	noncoding	2
	TCONS_l2_00017545	noncoding	1
	TCONS_l2_00017547	noncoding	1
	TCONS_l2_00017556	noncoding	1
	TCONS_l2_00022091	noncoding	1
	TCONS_l2_00022661	noncoding	1
	TCONS_l2_00025701	noncoding	1
	TCONS_l2_00027065	noncoding	1
	TCONS_l2_00029290	noncoding	1
	TCONS_l2_00029291	noncoding	1
	TCONS_l2_00029293	noncoding	2
	TCONS_l2_00029368	noncoding	2
	TCONS_l2_00029376	noncoding	2
	TCONS_l2_00029378	noncoding	2
	TCONS_l2_00029508	noncoding	1
	TCONS_l2_00029722	noncoding	2
	TCONS_l2_00029723	noncoding	3
	TCONS_l2_00029938	noncoding	2
	uc001zgs.1	noncoding	1
	uc002yji.2	noncoding	3
	uc002ykc.2	noncoding	9
	uc003ehd.2	noncoding	5
	uc003ttk.1	noncoding	1
	uc004acq.3	noncoding	1
	uc010rpo.1	noncoding	3
hsa-miR-196b-5p	AF049885	noncoding	6
	AK026822	noncoding	1
	BC043233	noncoding	1
	DA714598	noncoding	1
	ENST00000400178	noncoding	12
	ENST00000411795	noncoding	2

ENST00000416395	noncoding	2
ENST00000419640	noncoding	2
ENST00000419952	noncoding	10
ENST00000423764	noncoding	1
ENST00000427168	noncoding	3
ENST00000428669	noncoding	1
ENST00000436211	noncoding	1
ENST00000445438	noncoding	1
ENST00000445461	noncoding	10
ENST00000447039	noncoding	1
ENST00000448844	noncoding	1
ENST00000448927	noncoding	1
ENST00000450314	noncoding	2
ENST00000456327	noncoding	1
ENST00000456342	noncoding	12
ENST00000456587	noncoding	6
ENST00000457658	noncoding	1
ENST00000458155	noncoding	1
ENST00000458468	noncoding	12
ENST00000490162	noncoding	1
ENST00000497440	noncoding	3
ENST00000500162	noncoding	1
ENST00000504820	noncoding	2
ENST00000507508	noncoding	1
ENST00000518605	noncoding	1
ENST00000524346	noncoding	12
ENST00000524517	noncoding	1
ENST00000533992	noncoding	1
ENST00000555864	noncoding	2
ENST00000561977	noncoding	1
ENST00000563477	noncoding	2
ENST00000565979	noncoding	4
ENST00000573260	noncoding	1
Hs.130180	noncoding	2
Hs.570567	noncoding	1
Hs.653095	noncoding	2
Hs.712206	noncoding	1
Hs.722974	noncoding	1
Hs.728856	noncoding	2
Hs.731140	noncoding	1
Hs.98895	noncoding	1
M61867	noncoding	1
M61870	noncoding	1
M76743	noncoding	1
M97723	noncoding	1
NR_001543	noncoding	2
NR_002817	noncoding	2

NR_003191	noncoding	2
NR_003366	noncoding	2
NR_003662	noncoding	1
NR_015446	noncoding	3
NR_024559	noncoding	1
NR_024602	noncoding	1
NR_026558	noncoding	3
NR_027270	noncoding	1
NR_027469	noncoding	1
NR_027647	noncoding	1
NR_027663	noncoding	1
NR_027791	noncoding	10
NR_028080	noncoding	19
NR_033234	noncoding	2
NR_033807	noncoding	1
NR_033810	noncoding	1
NR_033811	noncoding	1
NR_033872	noncoding	2
NR_036489	noncoding	4
NR_036581	noncoding	1
NR_036627	noncoding	1
NR_036634	noncoding	4
NR_037918	noncoding	4
NR_038225	noncoding	1
NR_038226	noncoding	1
NR_038846	noncoding	1
NR_038877	noncoding	1
NR_040091	noncoding	1
NR_045012	noncoding	1
NR_045066	noncoding	11
NR_045211	noncoding	2
NR_045405	noncoding	1
NR_045563	noncoding	10
NR_047662	noncoding	1
NR_047680	noncoding	2
NR_047683	noncoding	2
NR_072979	noncoding	4
OTTHUMT00000314912	noncoding	1
OTTHUMT00000326022	noncoding	1
PBX1	coding	3
TCONS_00000692	noncoding	8
TCONS_00002831	noncoding	1
TCONS_00003850	noncoding	1
TCONS_00005778	noncoding	1
TCONS_00005790	noncoding	1
TCONS_00007856	noncoding	8
TCONS_00007857	noncoding	6

TCONS_00009804	noncoding	1
TCONS_00012852	noncoding	1
TCONS_00012947	noncoding	1
TCONS_00017606	noncoding	1
TCONS_00022478	noncoding	1
TCONS_00023208	noncoding	1
TCONS_00026032	noncoding	1
TCONS_l2_00000560	noncoding	1
TCONS_l2_00000562	noncoding	1
TCONS_l2_00000563	noncoding	2
TCONS_l2_00002162	noncoding	2
TCONS_l2_00002167	noncoding	1
TCONS_l2_00002168	noncoding	1
TCONS_l2_00002629	noncoding	2
TCONS_l2_00002630	noncoding	1
TCONS_l2_00002631	noncoding	1
TCONS_l2_00002632	noncoding	1
TCONS_l2_00003113	noncoding	1
TCONS_l2_00007047	noncoding	2
TCONS_l2_00009425	noncoding	2
TCONS_l2_00011786	noncoding	2
TCONS_l2_00015025	noncoding	2
TCONS_l2_00016247	noncoding	1
TCONS_l2_00016250	noncoding	1
TCONS_l2_00017139	noncoding	2
TCONS_l2_00017140	noncoding	1
TCONS_l2_00017143	noncoding	1
TCONS_l2_00017419	noncoding	4
TCONS_l2_00017424	noncoding	1
TCONS_l2_00017545	noncoding	1
TCONS_l2_00017547	noncoding	1
TCONS_l2_00017556	noncoding	1
TCONS_l2_00022661	noncoding	1
TCONS_l2_00027468	noncoding	2
TCONS_l2_00028763	noncoding	2
TCONS_l2_00029290	noncoding	1
TCONS_l2_00029291	noncoding	1
TCONS_l2_00029293	noncoding	2
TCONS_l2_00029327	noncoding	2
TCONS_l2_00029368	noncoding	5
TCONS_l2_00029376	noncoding	1
TCONS_l2_00029378	noncoding	3
TCONS_l2_00029508	noncoding	1
TCONS_l2_00029722	noncoding	2
TCONS_l2_00029723	noncoding	3
TCONS_l2_00029938	noncoding	2
uc001zgs.1	noncoding	2

	uc002sth.1	noncoding	1
	uc002yji.2	noncoding	1
	uc002ykc.2	noncoding	10
	uc003ehd.2	noncoding	2
	uc004acq.3	noncoding	3
hsa-miR-1972	AF049885	noncoding	7
	AF429306	noncoding	2
	AK055580	noncoding	1
	AK055738	noncoding	1
	AK056173	noncoding	3
	AK096049	noncoding	10
	AL833334	noncoding	1
	AY294627	noncoding	1
	AY462278	noncoding	1
	AY495952	noncoding	1
	BC040307	noncoding	1
	BC043233	noncoding	1
	CR623805	noncoding	1
	DA714598	noncoding	6
	DQ645738	noncoding	4
	DQ925687	noncoding	3
	ENST00000372173	noncoding	3
	ENST00000400178	noncoding	18
	ENST00000411795	noncoding	7
	ENST00000413004	noncoding	2
	ENST00000415330	noncoding	1
	ENST00000416395	noncoding	9
	ENST00000419952	noncoding	10
	ENST00000420195	noncoding	1
	ENST00000421996	noncoding	1
	ENST00000423764	noncoding	4
	ENST00000424774	noncoding	1
	ENST00000426185	noncoding	1
	ENST00000427168	noncoding	3
	ENST00000427491	noncoding	3
	ENST00000428669	noncoding	11
	ENST00000430699	noncoding	1
	ENST00000436211	noncoding	1
	ENST00000437249	noncoding	1
	ENST00000439302	noncoding	4
	ENST00000444754	noncoding	1
	ENST00000445438	noncoding	2
	ENST00000445461	noncoding	16
	ENST00000447956	noncoding	1
	ENST00000448844	noncoding	1
	ENST00000449955	noncoding	1
	ENST00000450314	noncoding	7

ENST00000453660	noncoding	2
ENST00000456280	noncoding	2
ENST00000456327	noncoding	3
ENST00000456342	noncoding	18
ENST00000456587	noncoding	6
ENST00000457658	noncoding	4
ENST00000458377	noncoding	6
ENST00000458468	noncoding	18
ENST00000490162	noncoding	1
ENST00000494791	noncoding	2
ENST00000497440	noncoding	11
ENST00000500162	noncoding	9
ENST00000500955	noncoding	4
ENST00000504820	noncoding	4
ENST00000507072	noncoding	3
ENST00000507508	noncoding	2
ENST00000509491	noncoding	4
ENST00000515455	noncoding	1
ENST00000517640	noncoding	1
ENST00000518172	noncoding	2
ENST00000518605	noncoding	2
ENST00000518902	noncoding	4
ENST00000524346	noncoding	19
ENST00000524517	noncoding	11
ENST00000527543	noncoding	2
ENST00000533992	noncoding	1
ENST00000539229	noncoding	3
ENST00000547824	noncoding	2
ENST00000548702	noncoding	1
ENST00000552502	noncoding	2
ENST00000553267	noncoding	1
ENST00000553909	noncoding	1
ENST00000555442	noncoding	2
ENST00000555862	noncoding	1
ENST00000555864	noncoding	1
ENST00000557602	noncoding	2
ENST00000557691	noncoding	1
ENST00000563477	noncoding	14
ENST00000563931	noncoding	2
ENST00000566475	noncoding	1
ENST00000568752	noncoding	8
ENST00000573260	noncoding	3
ENST00000573312	noncoding	1
ENST00000573494	noncoding	1
EPB41L1	coding	1
EU039832	noncoding	3
Hs.130180	noncoding	4

Hs.570567	noncoding	3
Hs.653095	noncoding	1
Hs.722974	noncoding	4
Hs.728856	noncoding	1
M26638	noncoding	1
M61870	noncoding	8
M76743	noncoding	2
M97723	noncoding	6
NR_001284	noncoding	1
NR_001543	noncoding	12
NR_002788	noncoding	3
NR_002806	noncoding	2
NR_002817	noncoding	4
NR_002925	noncoding	2
NR_003191	noncoding	6
NR_003260	noncoding	3
NR_003366	noncoding	14
NR_003662	noncoding	6
NR_003948	noncoding	1
NR_023388	noncoding	2
NR_024420	noncoding	3
NR_024461	noncoding	2
NR_024559	noncoding	8
NR_026558	noncoding	4
NR_026578	noncoding	2
NR_026677	noncoding	1
NR_026700	noncoding	2
NR_026759	noncoding	1
NR_026779	noncoding	1
NR_027270	noncoding	4
NR_027399	noncoding	9
NR_027469	noncoding	1
NR_027647	noncoding	4
NR_027663	noncoding	19
NR_027791	noncoding	16
NR_027995	noncoding	5
NR_028049	noncoding	2
NR_028080	noncoding	25
NR_028343	noncoding	3
NR_028439	noncoding	1
NR_028508	noncoding	1
NR_033234	noncoding	18
NR_033701	noncoding	2
NR_033798	noncoding	4
NR_033815	noncoding	7
NR_033828	noncoding	1
NR_033872	noncoding	21

NR_034112	noncoding	1
NR_036442	noncoding	2
NR_036468	noncoding	4
NR_036522	noncoding	1
NR_036581	noncoding	1
NR_036627	noncoding	1
NR_036634	noncoding	26
NR_037853_5	noncoding	1
NR_037918	noncoding	12
NR_038225	noncoding	6
NR_038226	noncoding	6
NR_038846	noncoding	1
NR_038877	noncoding	2
NR_038894	noncoding	3
NR_040091	noncoding	9
NR_040113	noncoding	6
NR_045012	noncoding	1
NR_045066	noncoding	5
NR_045128	noncoding	3
NR_045129	noncoding	2
NR_045211	noncoding	6
NR_045405	noncoding	5
NR_045563	noncoding	12
NR_046422	noncoding	2
NR_047651	noncoding	3
NR_047662	noncoding	9
NR_047680	noncoding	5
NR_047683	noncoding	5
NR_072979	noncoding	11
OTTHUMT00000134074	noncoding	1
OTTHUMT00000323603	noncoding	1
OTTHUMT00000326022	noncoding	6
TCONS_00000692	noncoding	7
TCONS_00000879	noncoding	1
TCONS_00002573	noncoding	1
TCONS_00002831	noncoding	1
TCONS_00003850	noncoding	1
TCONS_00005778	noncoding	1
TCONS_00005789	noncoding	1
TCONS_00005790	noncoding	3
TCONS_00005921	noncoding	4
TCONS_00007856	noncoding	18
TCONS_00007857	noncoding	11
TCONS_00009804	noncoding	1
TCONS_00011286	noncoding	1
TCONS_00012852	noncoding	4
TCONS_00015993	noncoding	1

TCONS_00016964	noncoding	7
TCONS_00017000	noncoding	1
TCONS_00017606	noncoding	7
TCONS_00017615	noncoding	6
TCONS_00019178	noncoding	1
TCONS_00020005	noncoding	3
TCONS_00020048	noncoding	1
TCONS_00021496	noncoding	1
TCONS_00022737	noncoding	1
TCONS_00023208	noncoding	7
TCONS_00023287	noncoding	2
TCONS_00023441	noncoding	2
TCONS_00023831	noncoding	1
TCONS_00024633	noncoding	3
TCONS_00024652	noncoding	2
TCONS_00026032	noncoding	1
TCONS_00027894	noncoding	1
TCONS_00029196	noncoding	4
TCONS_00029986	noncoding	1
TCONS_00030010	noncoding	1
TCONS_l2_00000030	noncoding	2
TCONS_l2_00000550	noncoding	1
TCONS_l2_00000551	noncoding	1
TCONS_l2_00000560	noncoding	1
TCONS_l2_00000563	noncoding	1
TCONS_l2_00001483	noncoding	2
TCONS_l2_00001485	noncoding	1
TCONS_l2_00001496	noncoding	3
TCONS_l2_00001497	noncoding	3
TCONS_l2_00001498	noncoding	3
TCONS_l2_00002157	noncoding	1
TCONS_l2_00002159	noncoding	1
TCONS_l2_00002162	noncoding	1
TCONS_l2_00002168	noncoding	1
TCONS_l2_00002491	noncoding	2
TCONS_l2_00002629	noncoding	1
TCONS_l2_00002630	noncoding	1
TCONS_l2_00002636	noncoding	1
TCONS_l2_00003113	noncoding	6
TCONS_l2_00003491	noncoding	1
TCONS_l2_00007044	noncoding	3
TCONS_l2_00007045	noncoding	1
TCONS_l2_00007081	noncoding	5
TCONS_l2_00007083	noncoding	1
TCONS_l2_00007497	noncoding	2
TCONS_l2_00009425	noncoding	3
TCONS_l2_00011031	noncoding	2

TCONS_l2_00011784	noncoding	1
TCONS_l2_00011786	noncoding	2
TCONS_l2_00012388	noncoding	3
TCONS_l2_00012563	noncoding	1
TCONS_l2_00012876	noncoding	5
TCONS_l2_00013073	noncoding	1
TCONS_l2_00013074	noncoding	1
TCONS_l2_00013919	noncoding	1
TCONS_l2_00014470	noncoding	1
TCONS_l2_00015025	noncoding	1
TCONS_l2_00015866	noncoding	1
TCONS_l2_00016247	noncoding	6
TCONS_l2_00016249	noncoding	1
TCONS_l2_00017135	noncoding	1
TCONS_l2_00017137	noncoding	3
TCONS_l2_00017139	noncoding	1
TCONS_l2_00017144	noncoding	1
TCONS_l2_00017419	noncoding	1
TCONS_l2_00017424	noncoding	3
TCONS_l2_00017545	noncoding	1
TCONS_l2_00017547	noncoding	1
TCONS_l2_00017556	noncoding	1
TCONS_l2_00019086	noncoding	14
TCONS_l2_00019088	noncoding	1
TCONS_l2_00019716	noncoding	1
TCONS_l2_00022091	noncoding	3
TCONS_l2_00022661	noncoding	7
TCONS_l2_00022666	noncoding	1
TCONS_l2_00028763	noncoding	4
TCONS_l2_00028814	noncoding	4
TCONS_l2_00028815	noncoding	1
TCONS_l2_00029290	noncoding	1
TCONS_l2_00029294	noncoding	2
TCONS_l2_00029327	noncoding	4
TCONS_l2_00029330	noncoding	2
TCONS_l2_00029368	noncoding	1
TCONS_l2_00029376	noncoding	1
TCONS_l2_00029378	noncoding	4
TCONS_l2_00029508	noncoding	1
TCONS_l2_00029722	noncoding	2
TCONS_l2_00029723	noncoding	2
TCONS_l2_00029938	noncoding	2
U11872	noncoding	3
U52699	noncoding	1
U52701	noncoding	4
uc001cqo.1	noncoding	2
uc001ejc.2	noncoding	1

	uc001hgr.2	noncoding	2
	uc001mmy.1	noncoding	5
	uc001zgs.1	noncoding	3
	uc002aeh.2	noncoding	2
	uc002qng.2	noncoding	1
	uc002sth.1	noncoding	2
	uc002yji.2	noncoding	4
	uc002ykc.2	noncoding	16
	uc003ehd.2	noncoding	9
	uc003hrl.1	noncoding	1
	uc003ttk.1	noncoding	1
	uc004acq.3	noncoding	4
	uc009yzx.1	noncoding	1
	uc010rpo.1	noncoding	12
	uc010tcj.1	noncoding	5
hsa-miR-200c-5p	AF049885	noncoding	2
	AF429306	noncoding	2
	ASAP3	coding	1
	DA714598	noncoding	1
	ENST00000400178	noncoding	7
	ENST00000415330	noncoding	1
	ENST00000416395	noncoding	1
	ENST00000419952	noncoding	4
	ENST00000426185	noncoding	1
	ENST00000427491	noncoding	1
	ENST00000428669	noncoding	4
	ENST00000445461	noncoding	5
	ENST00000453660	noncoding	1
	ENST00000456327	noncoding	1
	ENST00000456342	noncoding	7
	ENST00000456587	noncoding	1
	ENST00000458377	noncoding	1
	ENST00000458468	noncoding	7
	ENST00000497440	noncoding	4
	ENST00000504820	noncoding	2
	ENST00000507508	noncoding	1
	ENST00000509491	noncoding	1
	ENST00000517640	noncoding	1
	ENST00000524346	noncoding	6
	ENST00000526206	noncoding	1
	ENST00000553909	noncoding	1
	ENST00000563477	noncoding	1
	ENST00000566475	noncoding	1
	Hs.570567	noncoding	1
	Hs.653095	noncoding	1
	Hs.731140	noncoding	1
	M76743	noncoding	1

M97723	noncoding	3
NR_001543	noncoding	2
NR_002817	noncoding	1
NR_002925	noncoding	1
NR_003191	noncoding	1
NR_003366	noncoding	1
NR_003673	noncoding	1
NR_003948	noncoding	1
NR_024420	noncoding	1
NR_026558	noncoding	1
NR_027270	noncoding	1
NR_027647	noncoding	1
NR_027791	noncoding	5
NR_028080	noncoding	6
NR_033234	noncoding	2
NR_033798	noncoding	2
NR_033807	noncoding	1
NR_033810	noncoding	1
NR_033811	noncoding	1
NR_033872	noncoding	1
NR_034112	noncoding	1
NR_036468	noncoding	1
NR_036627	noncoding	1
NR_036634	noncoding	1
NR_037853_5	noncoding	1
NR_037918	noncoding	1
NR_038225	noncoding	1
NR_038226	noncoding	1
NR_038894	noncoding	1
NR_045012	noncoding	1
NR_045066	noncoding	1
NR_045211	noncoding	1
NR_045405	noncoding	1
NR_045563	noncoding	2
NR_046422	noncoding	1
OTTHUMT0000013407	noncoding	1
TCONS_00000692	noncoding	3
TCONS_00005790	noncoding	1
TCONS_00007856	noncoding	1
TCONS_00007857	noncoding	1
TCONS_00012852	noncoding	1
TCONS_00015820	noncoding	2
TCONS_00016964	noncoding	2
TCONS_00017606	noncoding	1
TCONS_00019178	noncoding	1
TCONS_00022863	noncoding	1
TCONS_00022864	noncoding	1

	TCONS_00023208	noncoding	2
	TCONS_00029196	noncoding	1
	TCONS_00029986	noncoding	1
	TCONS_00030010	noncoding	1
	TCONS_l2_00000030	noncoding	1
	TCONS_l2_00003113	noncoding	1
	TCONS_l2_00009425	noncoding	1
	TCONS_l2_00017144	noncoding	1
	TCONS_l2_00017424	noncoding	1
	TCONS_l2_00019716	noncoding	1
	TCONS_l2_00028763	noncoding	1
	TCONS_l2_00029327	noncoding	1
	TCONS_l2_00029378	noncoding	1
	U52701	noncoding	2
	uc001hgr.2	noncoding	1
	uc001zgs.1	noncoding	1
	uc002yji.2	noncoding	1
	uc002ykc.2	noncoding	5
	uc004acq.3	noncoding	1
hsa-miR-205-5p	ACACB	coding	1
	AF049885	noncoding	8
	AF539739	noncoding	1
	AK055738	noncoding	1
	AK056173	noncoding	1
	AK096049	noncoding	2
	AK123664	noncoding	2
	AL833334	noncoding	1
	AMOT	coding	1
	BC043233	noncoding	1
	BC128192	noncoding	1
	CR623805	noncoding	2
	DA714598	noncoding	2
	DQ645738	noncoding	1
	ENST00000400178	noncoding	19
	ENST00000411795	noncoding	1
	ENST00000419640	noncoding	1
	ENST00000419952	noncoding	12
	ENST00000427168	noncoding	1
	ENST00000427491	noncoding	2
	ENST00000428669	noncoding	11
	ENST00000437249	noncoding	2
	ENST00000445461	noncoding	18
	ENST00000450314	noncoding	2
	ENST00000456327	noncoding	1
	ENST00000456342	noncoding	19
	ENST00000456587	noncoding	2
	ENST00000457348	noncoding	1

ENST00000458468	noncoding	19
ENST00000497440	noncoding	8
ENST00000500162	noncoding	1
ENST00000500955	noncoding	1
ENST00000507508	noncoding	1
ENST00000509491	noncoding	1
ENST00000517640	noncoding	1
ENST00000518902	noncoding	1
ENST00000524346	noncoding	6
ENST00000524517	noncoding	6
ENST00000533992	noncoding	1
ENST00000539229	noncoding	2
ENST00000553682	noncoding	1
ENST00000555864	noncoding	1
ENST00000561507	noncoding	1
ENST00000563477	noncoding	1
ENST00000565979	noncoding	2
Hs.130180	noncoding	1
Hs.570567	noncoding	1
Hs.653095	noncoding	5
Hs.728856	noncoding	1
Hs.98895	noncoding	1
M61870	noncoding	1
M97723	noncoding	1
MAGI3	coding	2
NM_006735	noncoding	1
NR_001284	noncoding	1
NR_001543	noncoding	5
NR_003191	noncoding	2
NR_003662	noncoding	3
NR_024420	noncoding	1
NR_024559	noncoding	2
NR_027399	noncoding	2
NR_027469	noncoding	1
NR_027663	noncoding	2
NR_027791	noncoding	18
NR_028080	noncoding	14
NR_028343	noncoding	1
NR_033234	noncoding	6
NR_033701	noncoding	1
NR_033798	noncoding	2
NR_033807	noncoding	1
NR_033810	noncoding	1
NR_033811	noncoding	1
NR_033872	noncoding	3
NR_034112	noncoding	1
NR_036468	noncoding	1

NR_036489	noncoding	2
NR_036627	noncoding	2
NR_036634	noncoding	5
NR_037853_5	noncoding	1
NR_037918	noncoding	7
NR_038225	noncoding	3
NR_038226	noncoding	3
NR_038846	noncoding	1
NR_038877	noncoding	3
NR_038894	noncoding	2
NR_040091	noncoding	1
NR_045012	noncoding	1
NR_045066	noncoding	5
NR_045211	noncoding	2
NR_045563	noncoding	4
NR_047662	noncoding	1
NR_047680	noncoding	1
NR_047683	noncoding	1
NR_072979	noncoding	1
OTTHUMT00000326022	noncoding	1
TCONS_00000692	noncoding	8
TCONS_00000879	noncoding	1
TCONS_00002831	noncoding	1
TCONS_00005790	noncoding	1
TCONS_00007856	noncoding	4
TCONS_00007857	noncoding	3
TCONS_00009804	noncoding	4
TCONS_00012852	noncoding	3
TCONS_00014014	noncoding	1
TCONS_00016964	noncoding	3
TCONS_00017615	noncoding	3
TCONS_00023208	noncoding	2
TCONS_00023831	noncoding	1
TCONS_00027238	noncoding	1
TCONS_00029196	noncoding	4
TCONS_l2_00003113	noncoding	3
TCONS_l2_00007081	noncoding	1
TCONS_l2_00009425	noncoding	1
TCONS_l2_00011786	noncoding	1
TCONS_l2_00012388	noncoding	2
TCONS_l2_00012688	noncoding	1
TCONS_l2_00013779	noncoding	1
TCONS_l2_00013783	noncoding	1
TCONS_l2_00013919	noncoding	2
TCONS_l2_00016247	noncoding	3
TCONS_l2_00016249	noncoding	2
TCONS_l2_00017545	noncoding	1

	TCONS_l2_00017547	noncoding	1
	TCONS_l2_00017556	noncoding	1
	TCONS_l2_00019716	noncoding	1
	TCONS_l2_00022091	noncoding	1
	TCONS_l2_00029053	noncoding	1
	TCONS_l2_00029368	noncoding	3
	U52699	noncoding	1
	U52701	noncoding	1
	uc001hgr.2	noncoding	2
	uc001zgs.1	noncoding	1
	uc002aeh.2	noncoding	1
	uc002ykc.2	noncoding	18
	uc003ehd.2	noncoding	3
	uc003ttk.1	noncoding	2
	uc010lrv.1	noncoding	1
	uc010rpo.1	noncoding	3
hsa-miR-27a-5p	AF049885	noncoding	2
	AF429306	noncoding	3
	AK123664	noncoding	1
	AK123786	noncoding	1
	AK124319	noncoding	1
	BC017958	noncoding	1
	DLG2	coding	1
	ENST00000400178	noncoding	8
	ENST00000419952	noncoding	4
	ENST00000424774	noncoding	1
	ENST00000428669	noncoding	2
	ENST00000439302	noncoding	1
	ENST00000445461	noncoding	5
	ENST00000447478	noncoding	1
	ENST00000447956	noncoding	1
	ENST00000450314	noncoding	1
	ENST00000453660	noncoding	1
	ENST00000453998	noncoding	1
	ENST00000456327	noncoding	1
	ENST00000456342	noncoding	8
	ENST00000456587	noncoding	1
	ENST00000458468	noncoding	9
	ENST00000490162	noncoding	2
	ENST00000494791	noncoding	1
	ENST00000497440	noncoding	4
	ENST00000500162	noncoding	4
	ENST00000500955	noncoding	2
	ENST00000507508	noncoding	2
	ENST00000515455	noncoding	1
	ENST00000524346	noncoding	2
	ENST00000524517	noncoding	3

ENST00000526206	noncoding	1
ENST00000533992	noncoding	1
ENST00000548702	noncoding	1
ENST00000552502	noncoding	1
ENST00000553682	noncoding	1
ENST00000555442	noncoding	1
ENST00000555864	noncoding	1
ENST00000557602	noncoding	1
ENST00000563477	noncoding	1
EU039832	noncoding	1
Hs.130180	noncoding	1
Hs.569669	noncoding	1
Hs.570567	noncoding	1
Hs.653095	noncoding	1
Hs.722974	noncoding	2
M61870	noncoding	1
M76743	noncoding	1
M97723	noncoding	1
NR_001543	noncoding	2
NR_002788	noncoding	1
NR_002806	noncoding	1
NR_002817	noncoding	2
NR_003191	noncoding	1
NR_003366	noncoding	2
NR_003662	noncoding	2
NR_024014	noncoding	2
NR_024420	noncoding	1
NR_024441	noncoding	2
NR_024461	noncoding	1
NR_026558	noncoding	1
NR_026700	noncoding	1
NR_026759	noncoding	1
NR_027270	noncoding	1
NR_027647	noncoding	5
NR_027791	noncoding	6
NR_027995	noncoding	2
NR_028080	noncoding	6
NR_033701	noncoding	6
NR_033872	noncoding	3
NR_036522	noncoding	1
NR_036581	noncoding	2
NR_036627	noncoding	2
NR_036634	noncoding	6
NR_037918	noncoding	11
NR_038225	noncoding	3
NR_038226	noncoding	3
NR_038926	noncoding	1

NR_040091	noncoding	6
NR_040113	noncoding	1
NR_045012	noncoding	2
NR_045066	noncoding	5
NR_045128	noncoding	2
NR_045129	noncoding	2
NR_045211	noncoding	1
NR_045405	noncoding	3
NR_045563	noncoding	2
NR_047662	noncoding	4
NR_047680	noncoding	4
NR_047683	noncoding	4
NR_072979	noncoding	2
OTTHUMT00000157918	noncoding	2
OTTHUMT00000323603	noncoding	1
TCONS_00000692	noncoding	4
TCONS_00002831	noncoding	1
TCONS_00005778	noncoding	1
TCONS_00005789	noncoding	1
TCONS_00005790	noncoding	1
TCONS_00007856	noncoding	2
TCONS_00007857	noncoding	2
TCONS_00011286	noncoding	1
TCONS_00014014	noncoding	1
TCONS_00014788	noncoding	2
TCONS_00016964	noncoding	3
TCONS_00017000	noncoding	1
TCONS_00017606	noncoding	1
TCONS_00017649	noncoding	1
TCONS_00022863	noncoding	1
TCONS_00022864	noncoding	1
TCONS_00023208	noncoding	1
TCONS_00026032	noncoding	1
TCONS_00027894	noncoding	1
TCONS_00029196	noncoding	1
TCONS_l2_00001496	noncoding	1
TCONS_l2_00001497	noncoding	1
TCONS_l2_00001498	noncoding	1
TCONS_l2_00003113	noncoding	3
TCONS_l2_00003491	noncoding	1
TCONS_l2_00016247	noncoding	1
TCONS_l2_00016249	noncoding	1
TCONS_l2_00016250	noncoding	1
TCONS_l2_00017137	noncoding	1
TCONS_l2_00017326	noncoding	1
TCONS_l2_00017424	noncoding	1
TCONS_l2_00022091	noncoding	1

	TCONS_l2_00028763	noncoding	2
	TCONS_l2_00028814	noncoding	1
	TCONS_l2_00029327	noncoding	2
	TCONS_l2_00029330	noncoding	1
	TCONS_l2_00029376	noncoding	1
	TCONS_l2_00029378	noncoding	1
	TCONS_l2_00029508	noncoding	1
	U52699	noncoding	2
	U52701	noncoding	2
	uc002qng.2	noncoding	1
	uc002yji.2	noncoding	1
	uc002ykc.2	noncoding	6
	uc003ehd.2	noncoding	2
	uc004acq.3	noncoding	1
	uc010tcj.1	noncoding	2
hsa-miR-301a-3p	AF049885	noncoding	6
	AF429306	noncoding	1
	AK096049	noncoding	1
	ATP1A2	coding	1
	BC043233	noncoding	1
	ENST00000400178	noncoding	29
	ENST00000411795	noncoding	3
	ENST00000416395	noncoding	8
	ENST00000419640	noncoding	1
	ENST00000419952	noncoding	14
	ENST00000426185	noncoding	1
	ENST00000427168	noncoding	2
	ENST00000427491	noncoding	1
	ENST00000428669	noncoding	11
	ENST00000430699	noncoding	2
	ENST00000439302	noncoding	1
	ENST00000445438	noncoding	1
	ENST00000445461	noncoding	21
	ENST00000447956	noncoding	1
	ENST00000450314	noncoding	3
	ENST00000456327	noncoding	1
	ENST00000456342	noncoding	29
	ENST00000456587	noncoding	6
	ENST00000457658	noncoding	1
	ENST00000458377	noncoding	1
	ENST00000458468	noncoding	29
	ENST00000490162	noncoding	1
	ENST00000494791	noncoding	1
	ENST00000497440	noncoding	7
	ENST00000500162	noncoding	4
	ENST00000500955	noncoding	1
	ENST00000504820	noncoding	5

ENST00000507508	noncoding	3
ENST00000517640	noncoding	1
ENST00000518046	noncoding	1
ENST00000518605	noncoding	1
ENST00000518902	noncoding	1
ENST00000524346	noncoding	15
ENST00000524517	noncoding	3
ENST00000527543	noncoding	1
ENST00000533992	noncoding	1
ENST00000545818	noncoding	1
ENST00000553909	noncoding	1
ENST00000555442	noncoding	2
ENST00000555864	noncoding	1
ENST00000557602	noncoding	1
ENST00000561977	noncoding	1
ENST00000563477	noncoding	4
ENST00000565979	noncoding	1
ENST00000566475	noncoding	1
ENST00000568091	noncoding	1
EU039832	noncoding	2
GPT2	coding	1
Hs.130180	noncoding	1
Hs.570567	noncoding	2
Hs.653095	noncoding	3
Hs.728856	noncoding	3
M33328	noncoding	1
M76743	noncoding	2
M97723	noncoding	1
NR_001543	noncoding	5
NR_002788	noncoding	3
NR_002817	noncoding	1
NR_002940_3	noncoding	1
NR_003191	noncoding	1
NR_003260	noncoding	1
NR_003366	noncoding	1
NR_003662	noncoding	2
NR_003673	noncoding	1
NR_003948	noncoding	1
NR_024461	noncoding	1
NR_024602	noncoding	2
NR_026558	noncoding	2
NR_026578	noncoding	2
NR_026829	noncoding	1
NR_027270	noncoding	1
NR_027399	noncoding	2
NR_027647	noncoding	1
NR_027663	noncoding	5

NR_027791	noncoding	21
NR_027995	noncoding	1
NR_028049	noncoding	1
NR_028080	noncoding	23
NR_028439	noncoding	1
NR_030732	noncoding	1
NR_033234	noncoding	4
NR_033798	noncoding	1
NR_033807	noncoding	1
NR_033810	noncoding	1
NR_033811	noncoding	1
NR_033872	noncoding	10
NR_036468	noncoding	1
NR_036489	noncoding	1
NR_036627	noncoding	4
NR_036634	noncoding	5
NR_037853_5	noncoding	1
NR_037918	noncoding	20
NR_038225	noncoding	3
NR_038226	noncoding	3
NR_038433	noncoding	1
NR_038877	noncoding	2
NR_038894	noncoding	1
NR_040091	noncoding	8
NR_040113	noncoding	1
NR_045066	noncoding	7
NR_045128	noncoding	2
NR_045129	noncoding	2
NR_045211	noncoding	1
NR_045405	noncoding	2
NR_045563	noncoding	8
NR_046422	noncoding	1
NR_047662	noncoding	4
NR_047680	noncoding	4
NR_047683	noncoding	4
NR_072979	noncoding	2
NR3C2	coding	1
OTTHUMT0000028061[illegible]	noncoding	1
OTTHUMT0000031491[illegible]	noncoding	1
OTTHUMT0000032360[illegible]	noncoding	1
TCONS_00000692	noncoding	4
TCONS_00000879	noncoding	1
TCONS_00003049	noncoding	1
TCONS_00004435	noncoding	2
TCONS_00005790	noncoding	2
TCONS_00005921	noncoding	5
TCONS_00007856	noncoding	8

TCONS_00007857	noncoding	2
TCONS_00012852	noncoding	1
TCONS_00016354	noncoding	1
TCONS_00016964	noncoding	5
TCONS_00017606	noncoding	2
TCONS_00017615	noncoding	1
TCONS_00017649	noncoding	1
TCONS_00022478	noncoding	1
TCONS_00022737	noncoding	1
TCONS_00022863	noncoding	1
TCONS_00022864	noncoding	1
TCONS_00023208	noncoding	3
TCONS_00025714	noncoding	1
TCONS_00027894	noncoding	1
TCONS_00028829	noncoding	1
TCONS_00029196	noncoding	3
TCONS_00030010	noncoding	2
TCONS_l2_00000549	noncoding	1
TCONS_l2_00001496	noncoding	1
TCONS_l2_00001497	noncoding	1
TCONS_l2_00001498	noncoding	1
TCONS_l2_00002156	noncoding	2
TCONS_l2_00002157	noncoding	2
TCONS_l2_00002159	noncoding	1
TCONS_l2_00002491	noncoding	1
TCONS_l2_00002637	noncoding	1
TCONS_l2_00002638	noncoding	1
TCONS_l2_00003113	noncoding	3
TCONS_l2_00003491	noncoding	1
TCONS_l2_00007081	noncoding	1
TCONS_l2_00007497	noncoding	1
TCONS_l2_00012388	noncoding	2
TCONS_l2_00012688	noncoding	1
TCONS_l2_00013919	noncoding	1
TCONS_l2_00014470	noncoding	1
TCONS_l2_00015025	noncoding	2
TCONS_l2_00016247	noncoding	1
TCONS_l2_00016249	noncoding	1
TCONS_l2_00017144	noncoding	1
TCONS_l2_00017545	noncoding	4
TCONS_l2_00017547	noncoding	3
TCONS_l2_00017556	noncoding	3
TCONS_l2_00017559	noncoding	1
TCONS_l2_00019716	noncoding	1
TCONS_l2_00022091	noncoding	1
TCONS_l2_00028763	noncoding	1
TCONS_l2_00029327	noncoding	1

	TCONS_l2_00029376	noncoding	1
	TCONS_l2_00029378	noncoding	1
	TCONS_l2_00029508	noncoding	1
	TGFBR2	coding	1
	uc001cqo.1	noncoding	1
	uc001ejc.2	noncoding	1
	uc001mmy.1	noncoding	1
	uc002aeh.2	noncoding	1
	uc002yji.2	noncoding	1
	uc002ykc.2	noncoding	21
	uc003ehd.2	noncoding	3
	uc003isk.1	noncoding	1
	uc004acq.3	noncoding	2
	uc010rpo.1	noncoding	3
	uc010tcj.1	noncoding	1
hsa-miR-30a-5p	ACVR1	coding	1
	AF086566	noncoding	1
	AF195420	noncoding	4
	AK023033	noncoding	1
	AK075059	noncoding	10
	AK094884	noncoding	2
	AK130538	noncoding	1
	AY493738	noncoding	3
	AY665469	noncoding	1
	BC035585	noncoding	4
	BC035645	noncoding	8
	BC044613	noncoding	1
	BC062758	noncoding	1
	CHST1	coding	1
	CR611979	noncoding	3
	CR621930	noncoding	1
	D83986	noncoding	1
	D90187	noncoding	2
	DDIT4	coding	1
	DQ004397	noncoding	5
	EDNRA	coding	1
	ENST00000383038	noncoding	1
	ENST00000409139	noncoding	1
	ENST00000409898	noncoding	1
	ENST00000412017	noncoding	1
	ENST00000418499	noncoding	1
	ENST00000426452	noncoding	1
	ENST00000427303	noncoding	2
	ENST00000428865	noncoding	1
	ENST00000431094	noncoding	1
	ENST00000432001	noncoding	1
	ENST00000437541	noncoding	1

ENST00000437597	noncoding	1
ENST00000438128	noncoding	1
ENST00000439070	noncoding	2
ENST00000439362	noncoding	2
ENST00000441500	noncoding	1
ENST00000444301	noncoding	1
ENST00000445673	noncoding	4
ENST00000452397	noncoding	1
ENST00000453665	noncoding	1
ENST00000455088	noncoding	1
ENST00000466034	noncoding	1
ENST00000493038	noncoding	3
ENST00000509144	noncoding	2
ENST00000513815	noncoding	1
ENST00000513868	noncoding	3
ENST00000513899	noncoding	1
ENST00000518528	noncoding	1
ENST00000518932	noncoding	2
ENST00000520433	noncoding	2
ENST00000520594	noncoding	2
ENST00000520598	noncoding	1
ENST00000521122	noncoding	1
ENST00000524045	noncoding	2
ENST00000525302	noncoding	1
ENST00000526061	noncoding	1
ENST00000526220	noncoding	1
ENST00000530690	noncoding	1
ENST00000547285	noncoding	1
ENST00000548271	noncoding	1
ENST00000548475	noncoding	1
ENST00000549813	noncoding	1
ENST00000550717	noncoding	6
ENST00000553119	noncoding	3
ENST00000553153	noncoding	1
ENST00000555937	noncoding	1
ENST00000564705	noncoding	1
ENST00000570945	noncoding	1
ENST00000571639	noncoding	1
ENST00000574246	noncoding	1
EPHB2	coding	1
FZD2	coding	1
GALNT2	coding	1
GJA1	coding	1
GNG10	coding	2
Hs.190748	noncoding	1
Hs.537370	noncoding	1
Hs.571424	noncoding	1

Hs.616703	noncoding	4
Hs.715338	noncoding	1
M90464	noncoding	8
NR_003105	noncoding	1
NR_003135	noncoding	2
NR_003149	noncoding	11
NR_003367	noncoding	3
NR_015395	noncoding	1
NR_023391	noncoding	1
NR_026758	noncoding	2
NR_026899	noncoding	1
NR_026975	noncoding	2
NR_027140	noncoding	1
NR_027458	noncoding	8
NR_027633	noncoding	1
NR_027676	noncoding	1
NR_030728	noncoding	2
NR_033344	noncoding	2
NR_033652	noncoding	1
NR_034147	noncoding	2
NR_036488	noncoding	2
NR_036680	noncoding	5
NR_037857	noncoding	1
NR_037944	noncoding	1
NR_038219	noncoding	1
NR_038306	noncoding	1
NR_045721	noncoding	1
NR_045864	noncoding	2
NR_046318	noncoding	2
NR_046337	noncoding	1
NR_047513	noncoding	1
NR_047584	noncoding	2
NR_049767	noncoding	1
NR_052852	noncoding	1
NR_073017	noncoding	2
NR_073018	noncoding	2
S67859	noncoding	5
S69184	noncoding	1
TCONS_00002272	noncoding	1
TCONS_00021491	noncoding	2
TCONS_00022699	noncoding	1
TCONS_00023067	noncoding	1
TCONS_00027897	noncoding	2
TCONS_00029375	noncoding	1
TCONS_l2_00003977	noncoding	1
TCONS_l2_00007759	noncoding	1
TCONS_l2_00007970	noncoding	1

TCONS_l2_00008291	noncoding	1
TCONS_l2_00008295	noncoding	1
TCONS_l2_00008296	noncoding	1
TCONS_l2_00013787	noncoding	1
TCONS_l2_00014788	noncoding	2
TCONS_l2_00014792	noncoding	2
TCONS_l2_00014793	noncoding	1
TCONS_l2_00014795	noncoding	1
TCONS_l2_00014801	noncoding	1
TCONS_l2_00014802	noncoding	1
TCONS_l2_00014889	noncoding	2
TCONS_l2_00015810	noncoding	2
TCONS_l2_00015911	noncoding	2
TCONS_l2_00016214	noncoding	1
TCONS_l2_00017150	noncoding	1
TCONS_l2_00017478	noncoding	2
TCONS_l2_00017848	noncoding	1
TCONS_l2_00017850	noncoding	1
TCONS_l2_00018290	noncoding	3
TCONS_l2_00018291	noncoding	1
TCONS_l2_00018292	noncoding	1
TCONS_l2_00018293	noncoding	1
TCONS_l2_00018977	noncoding	1
TCONS_l2_00018981	noncoding	2
TCONS_l2_00019959	noncoding	2
TCONS_l2_00020648	noncoding	1
TCONS_l2_00020697	noncoding	1
TCONS_l2_00023705	noncoding	2
TCONS_l2_00023843	noncoding	1
TCONS_l2_00023844	noncoding	1
TCONS_l2_00024448	noncoding	2
TCONS_l2_00030598	noncoding	2
U31851	noncoding	1
uc001koj.3	noncoding	1
uc001veg.1	noncoding	4
uc001vvq.1	noncoding	3
uc002snv.3	noncoding	1
uc002tru.1	noncoding	2
uc002twe.2	noncoding	4
uc003hlf.2	noncoding	1
uc004ewj.1	noncoding	2
uc010ado.1	noncoding	4
uc010amc.1	noncoding	8
uc010jpp.1	noncoding	3
uc010tgn.1	noncoding	4
X05562	noncoding	3
XR_132779	noncoding	2

	Z50167	noncoding	1
hsa-miR-3613-5p	AB073212	noncoding	1
	AF055459	noncoding	2
	AF090428	noncoding	1
	AF195420	noncoding	10
	AK023033	noncoding	1
	AK054973	noncoding	1
	AK056476	noncoding	1
	AK075059	noncoding	7
	AK094884	noncoding	2
	AY425955	noncoding	1
	AY493738	noncoding	3
	AY665469	noncoding	1
	BC017481	noncoding	2
	BC035585	noncoding	2
	BC035645	noncoding	3
	BC036851	noncoding	1
	BC048192	noncoding	1
	BC057832	noncoding	2
	BC062758	noncoding	1
	BX248289	noncoding	2
	CDK6	coding	1
	CR611979	noncoding	1
	CR615536	noncoding	2
	D83986	noncoding	1
	D84212	noncoding	1
	D90187	noncoding	1
	DQ004397	noncoding	2
	ENST00000304425	noncoding	4
	ENST00000383038	noncoding	2
	ENST00000409139	noncoding	1
	ENST00000409898	noncoding	1
	ENST00000412017	noncoding	2
	ENST00000417691	noncoding	1
	ENST00000418499	noncoding	2
	ENST00000431094	noncoding	2
	ENST00000432001	noncoding	2
	ENST00000434244	noncoding	2
	ENST00000434951	noncoding	1
	ENST00000438128	noncoding	2
	ENST00000439070	noncoding	2
	ENST00000439362	noncoding	3
	ENST00000441500	noncoding	2
	ENST00000442293	noncoding	1
	ENST00000444301	noncoding	2
	ENST00000444872	noncoding	2
	ENST00000445673	noncoding	1

ENST00000453665	noncoding	1
ENST00000455088	noncoding	2
ENST00000493038	noncoding	3
ENST00000504081	noncoding	1
ENST00000509144	noncoding	5
ENST00000513868	noncoding	3
ENST00000513899	noncoding	1
ENST00000518932	noncoding	1
ENST00000520422	noncoding	1
ENST00000520433	noncoding	5
ENST00000520594	noncoding	5
ENST00000521252	noncoding	1
ENST00000521666	noncoding	1
ENST00000524045	noncoding	5
ENST00000525302	noncoding	3
ENST00000526061	noncoding	4
ENST00000526220	noncoding	1
ENST00000547285	noncoding	2
ENST00000548057	noncoding	1
ENST00000548271	noncoding	2
ENST00000548475	noncoding	2
ENST00000549813	noncoding	2
ENST00000550717	noncoding	6
ENST00000551881	noncoding	1
ENST00000553119	noncoding	3
ENST00000553153	noncoding	2
ENST00000555937	noncoding	1
ENST00000557790	noncoding	1
ENST00000558297	noncoding	1
ENST00000559979	noncoding	1
ENST00000560732	noncoding	1
ENST00000560800	noncoding	8
ENST00000564705	noncoding	1
ENST00000569678	noncoding	1
ENST00000575838	noncoding	1
Hs.537370	noncoding	2
Hs.571424	noncoding	1
Hs.616703	noncoding	3
L08437	noncoding	1
M90464	noncoding	3
NM_002148	noncoding	1
NM_006884	noncoding	1
NR_003105	noncoding	1
NR_003149	noncoding	4
NR_003367	noncoding	3
NR_015395	noncoding	2
NR_015404	noncoding	1

NR_026975	noncoding	9
NR_027053	noncoding	1
NR_027140	noncoding	1
NR_027458	noncoding	2
NR_027645	noncoding	1
NR_027646	noncoding	1
NR_027676	noncoding	5
NR_027889	noncoding	1
NR_027917	noncoding	1
NR_028444	noncoding	3
NR_033321	noncoding	1
NR_033344	noncoding	3
NR_033404	noncoding	2
NR_033652	noncoding	1
NR_036680	noncoding	3
NR_037857	noncoding	1
NR_037944	noncoding	1
NR_045721	noncoding	1
NR_045864	noncoding	2
NR_046337	noncoding	2
NR_047513	noncoding	1
NR_047584	noncoding	1
NR_047648	noncoding	1
NR_073017	noncoding	4
NR_073018	noncoding	4
OTTHUMT00000320637	noncoding	1
S46006	noncoding	1
TCONS_00000563	noncoding	1
TCONS_00003298	noncoding	1
TCONS_00006729	noncoding	1
TCONS_00007547	noncoding	1
TCONS_00013157	noncoding	2
TCONS_00014975	noncoding	1
TCONS_00015427	noncoding	1
TCONS_00015888	noncoding	1
TCONS_00017041	noncoding	1
TCONS_00020222	noncoding	1
TCONS_00025465	noncoding	1
TCONS_00029375	noncoding	1
TCONS_l2_00003977	noncoding	1
TCONS_l2_00007970	noncoding	2
TCONS_l2_00008291	noncoding	2
TCONS_l2_00008295	noncoding	2
TCONS_l2_00008296	noncoding	2
TCONS_l2_00008531	noncoding	1
TCONS_l2_00014788	noncoding	3
TCONS_l2_00014792	noncoding	3

	TCONS_l2_00014793	noncoding	1
	TCONS_l2_00014795	noncoding	1
	TCONS_l2_00014801	noncoding	2
	TCONS_l2_00014802	noncoding	2
	TCONS_l2_00015810	noncoding	1
	TCONS_l2_00015911	noncoding	3
	TCONS_l2_00015996	noncoding	1
	TCONS_l2_00017478	noncoding	4
	TCONS_l2_00017848	noncoding	2
	TCONS_l2_00017850	noncoding	2
	TCONS_l2_00018290	noncoding	3
	TCONS_l2_00018291	noncoding	2
	TCONS_l2_00018292	noncoding	2
	TCONS_l2_00018293	noncoding	2
	TCONS_l2_00018313	noncoding	1
	TCONS_l2_00018977	noncoding	1
	TCONS_l2_00018981	noncoding	1
	TCONS_l2_00019959	noncoding	8
	TCONS_l2_00023705	noncoding	6
	TCONS_l2_00023706	noncoding	1
	TCONS_l2_00030598	noncoding	3
	U31851	noncoding	3
	uc001flf.2	noncoding	2
	uc001veg.1	noncoding	3
	uc001vvq.1	noncoding	3
	uc002snv.3	noncoding	1
	uc002tte.2	noncoding	1
	uc003qeq.1	noncoding	1
	uc004ewj.1	noncoding	6
	uc010ado.1	noncoding	3
	uc010amc.1	noncoding	7
	uc010jpp.1	noncoding	8
	uc010tgn.1	noncoding	3
	X05562	noncoding	3
	XR_109374	noncoding	1
	XR_132779	noncoding	2
hsa-miR-421	AF049885	noncoding	5
	AF086436	noncoding	1
	AF429306	noncoding	4
	AK096049	noncoding	2
	AK123264	noncoding	2
	AK128778	noncoding	1
	AY462278	noncoding	1
	AY495952	noncoding	1
	BC033124	noncoding	1
	BC043233	noncoding	1
	BC071777	noncoding	1

DA714598	noncoding	1
ENST00000372173	noncoding	1
ENST00000400178	noncoding	17
ENST00000411775	noncoding	1
ENST00000413004	noncoding	1
ENST00000416395	noncoding	3
ENST00000419952	noncoding	10
ENST00000420195	noncoding	1
ENST00000427168	noncoding	2
ENST00000428669	noncoding	6
ENST00000430699	noncoding	1
ENST00000437249	noncoding	1
ENST00000439302	noncoding	1
ENST00000444754	noncoding	1
ENST00000445461	noncoding	14
ENST00000447956	noncoding	1
ENST00000448844	noncoding	2
ENST00000450314	noncoding	1
ENST00000452738	noncoding	1
ENST00000456327	noncoding	1
ENST00000456342	noncoding	17
ENST00000456587	noncoding	4
ENST00000457348	noncoding	1
ENST00000457658	noncoding	2
ENST00000458155	noncoding	2
ENST00000458468	noncoding	18
ENST00000490162	noncoding	1
ENST00000497440	noncoding	3
ENST00000500162	noncoding	4
ENST00000500955	noncoding	3
ENST00000504820	noncoding	1
ENST00000507508	noncoding	3
ENST00000509491	noncoding	2
ENST00000515455	noncoding	1
ENST00000518605	noncoding	1
ENST00000524346	noncoding	20
ENST00000524517	noncoding	1
ENST00000533992	noncoding	1
ENST00000534849	noncoding	1
ENST00000553682	noncoding	1
ENST00000555864	noncoding	1
ENST00000557691	noncoding	1
ENST00000560740	noncoding	1
ENST00000565979	noncoding	1
EU039832	noncoding	1
Hs.570567	noncoding	1
Hs.592473	noncoding	1

Hs.712206	noncoding	3
Hs.722974	noncoding	1
Hs.728856	noncoding	1
Hs.98895	noncoding	1
M61867	noncoding	1
M61870	noncoding	2
NR_001284_5	noncoding	1
NR_001543	noncoding	3
NR_001544	noncoding	2
NR_002788	noncoding	4
NR_002806	noncoding	1
NR_002817	noncoding	1
NR_002925	noncoding	4
NR_003191	noncoding	2
NR_003260	noncoding	1
NR_003366	noncoding	2
NR_003662	noncoding	1
NR_024559	noncoding	1
NR_024602	noncoding	1
NR_026558	noncoding	1
NR_026578	noncoding	1
NR_027469	noncoding	1
NR_027647	noncoding	1
NR_027663	noncoding	3
NR_027791	noncoding	15
NR_027995	noncoding	2
NR_028049	noncoding	1
NR_028080	noncoding	23
NR_033234	noncoding	6
NR_033798	noncoding	2
NR_033815	noncoding	2
NR_033828	noncoding	1
NR_033872	noncoding	9
NR_036489	noncoding	1
NR_036627	noncoding	3
NR_036634	noncoding	10
NR_037918	noncoding	7
NR_038225	noncoding	1
NR_038226	noncoding	1
NR_038433	noncoding	1
NR_038877	noncoding	2
NR_040091	noncoding	7
NR_040113	noncoding	2
NR_045012	noncoding	1
NR_045066	noncoding	14
NR_045128	noncoding	3
NR_045129	noncoding	1

NR_045211	noncoding	2
NR_045405	noncoding	6
NR_045563	noncoding	9
NR_047662	noncoding	4
NR_047680	noncoding	2
NR_047683	noncoding	2
NR_072979	noncoding	3
OTTHUMT00000047661	noncoding	1
OTTHUMT00000326022	noncoding	1
SLC4A4	coding	2
TCONS_00000692	noncoding	4
TCONS_00002831	noncoding	1
TCONS_00005778	noncoding	2
TCONS_00005790	noncoding	1
TCONS_00005921	noncoding	1
TCONS_00007856	noncoding	6
TCONS_00007857	noncoding	4
TCONS_00009804	noncoding	1
TCONS_00012852	noncoding	3
TCONS_00015993	noncoding	1
TCONS_00016354	noncoding	2
TCONS_00016964	noncoding	6
TCONS_00017590	noncoding	2
TCONS_00017606	noncoding	4
TCONS_00017649	noncoding	2
TCONS_00018105	noncoding	1
TCONS_00020048	noncoding	1
TCONS_00024633	noncoding	1
TCONS_00024652	noncoding	2
TCONS_00027894	noncoding	1
TCONS_00029196	noncoding	1
TCONS_00029197	noncoding	1
TCONS_l2_00000550	noncoding	2
TCONS_l2_00000551	noncoding	1
TCONS_l2_00000560	noncoding	2
TCONS_l2_00000562	noncoding	2
TCONS_l2_00000563	noncoding	2
TCONS_l2_00001483	noncoding	1
TCONS_l2_00001485	noncoding	1
TCONS_l2_00001496	noncoding	1
TCONS_l2_00002157	noncoding	1
TCONS_l2_00002159	noncoding	2
TCONS_l2_00002161	noncoding	1
TCONS_l2_00002162	noncoding	3
TCONS_l2_00002165	noncoding	1
TCONS_l2_00002167	noncoding	2
TCONS_l2_00002168	noncoding	2

TCONS_l2_00002491	noncoding	3
TCONS_l2_00002629	noncoding	2
TCONS_l2_00002630	noncoding	2
TCONS_l2_00002631	noncoding	2
TCONS_l2_00002632	noncoding	2
TCONS_l2_00002634	noncoding	1
TCONS_l2_00002636	noncoding	1
TCONS_l2_00002637	noncoding	1
TCONS_l2_00003113	noncoding	1
TCONS_l2_00007081	noncoding	1
TCONS_l2_00010129	noncoding	2
TCONS_l2_00011784	noncoding	2
TCONS_l2_00011786	noncoding	1
TCONS_l2_00011787	noncoding	1
TCONS_l2_00012388	noncoding	1
TCONS_l2_00013919	noncoding	1
TCONS_l2_00016247	noncoding	1
TCONS_l2_00017139	noncoding	2
TCONS_l2_00017142	noncoding	1
TCONS_l2_00017143	noncoding	1
TCONS_l2_00017419	noncoding	2
TCONS_l2_00017422	noncoding	1
TCONS_l2_00017545	noncoding	2
TCONS_l2_00017547	noncoding	2
TCONS_l2_00017556	noncoding	2
TCONS_l2_00019088	noncoding	1
TCONS_l2_00019716	noncoding	2
TCONS_l2_00021132	noncoding	1
TCONS_l2_00022091	noncoding	1
TCONS_l2_00022661	noncoding	1
TCONS_l2_00028763	noncoding	1
TCONS_l2_00029293	noncoding	1
TCONS_l2_00029327	noncoding	1
TCONS_l2_00029330	noncoding	1
TCONS_l2_00029339	noncoding	1
TCONS_l2_00029378	noncoding	1
TCONS_l2_00029508	noncoding	3
TCONS_l2_00029723	noncoding	1
uc.254+	noncoding	1
uc001cqo.1	noncoding	3
uc001ejc.2	noncoding	2
uc001hgr.2	noncoding	3
uc002aeh.2	noncoding	1
uc002sth.1	noncoding	1
uc002ykc.2	noncoding	15
uc003ehd.2	noncoding	10
uc004acq.3	noncoding	1

	uc010rpo.1	noncoding	1
	uc010tcj.1	noncoding	2
	XR_110606	noncoding	1
hsa-miR-429	AF049885	noncoding	4
	AF202879	noncoding	1
	AK097064	noncoding	1
	AK124319	noncoding	1
	AY462278	noncoding	1
	AY495952	noncoding	1
	CR542200	noncoding	1
	CR627122	noncoding	1
	ENST00000400178	noncoding	10
	ENST00000419952	noncoding	3
	ENST00000428669	noncoding	2
	ENST00000430699	noncoding	1
	ENST00000439302	noncoding	2
	ENST00000445461	noncoding	4
	ENST00000447039	noncoding	1
	ENST00000450314	noncoding	2
	ENST00000456342	noncoding	10
	ENST00000456587	noncoding	1
	ENST00000458377	noncoding	1
	ENST00000458468	noncoding	10
	ENST00000497440	noncoding	4
	ENST00000500162	noncoding	5
	ENST00000500955	noncoding	2
	ENST00000504820	noncoding	1
	ENST00000509491	noncoding	1
	ENST00000524346	noncoding	8
	ENST00000561977	noncoding	1
	ENST00000573260	noncoding	1
	EU039832	noncoding	1
	Hs.570567	noncoding	1
	Hs.592473	noncoding	1
	Hs.653095	noncoding	1
	Hs.722974	noncoding	1
	K03207	noncoding	1
	LPIN1	coding	1
	M61870	noncoding	1
	NR_001543	noncoding	2
	NR_002788	noncoding	2
	NR_002925	noncoding	2
	NR_003366	noncoding	1
	NR_003662	noncoding	1
	NR_024420	noncoding	1
	NR_027399	noncoding	2
	NR_027663	noncoding	1

NR_027791	noncoding	4
NR_028080	noncoding	8
NR_033234	noncoding	2
NR_033798	noncoding	1
NR_036627	noncoding	1
NR_036634	noncoding	4
NR_037918	noncoding	3
NR_040091	noncoding	3
NR_045066	noncoding	5
NR_045128	noncoding	4
NR_045129	noncoding	2
NR_045405	noncoding	2
NR_045563	noncoding	7
NR_047662	noncoding	5
NR_047680	noncoding	1
NR_047683	noncoding	1
OTTHUMT00000314912	noncoding	1
TCONS_00000692	noncoding	1
TCONS_00005790	noncoding	1
TCONS_00005921	noncoding	1
TCONS_00007856	noncoding	4
TCONS_00007857	noncoding	4
TCONS_00012852	noncoding	1
TCONS_00012947	noncoding	1
TCONS_00016964	noncoding	1
TCONS_00017000	noncoding	1
TCONS_00017606	noncoding	3
TCONS_00017615	noncoding	2
TCONS_00020232	noncoding	1
TCONS_00023208	noncoding	2
TCONS_00029196	noncoding	1
TCONS_l2_00001496	noncoding	2
TCONS_l2_00001497	noncoding	1
TCONS_l2_00001498	noncoding	1
TCONS_l2_00002156	noncoding	1
TCONS_l2_00002157	noncoding	1
TCONS_l2_00002159	noncoding	1
TCONS_l2_00002491	noncoding	1
TCONS_l2_00003491	noncoding	1
TCONS_l2_00011988	noncoding	1
TCONS_l2_00012388	noncoding	1
TCONS_l2_00015025	noncoding	1
TCONS_l2_00017545	noncoding	2
TCONS_l2_00017547	noncoding	2
TCONS_l2_00017556	noncoding	2
TCONS_l2_00022091	noncoding	1
TCONS_l2_00029376	noncoding	1

	U11872	noncoding	1
	uc001cqo.1	noncoding	1
	uc001mmy.1	noncoding	1
	uc002aeh.2	noncoding	3
	uc002ykc.2	noncoding	4
	uc003ehd.2	noncoding	2
hsa-miR-4317	AF049885	noncoding	1
	AF429306	noncoding	1
	AK126763	noncoding	1
	CR623805	noncoding	1
	CR627122	noncoding	1
	DA714598	noncoding	1
	ENST00000400178	noncoding	6
	ENST00000411775	noncoding	1
	ENST00000411795	noncoding	1
	ENST00000419952	noncoding	3
	ENST00000427168	noncoding	1
	ENST00000427491	noncoding	1
	ENST00000428669	noncoding	2
	ENST00000430699	noncoding	1
	ENST00000435892	noncoding	1
	ENST00000437249	noncoding	1
	ENST00000439302	noncoding	2
	ENST00000442663	noncoding	1
	ENST00000444754	noncoding	1
	ENST00000445461	noncoding	3
	ENST00000450314	noncoding	1
	ENST00000456342	noncoding	6
	ENST00000456587	noncoding	2
	ENST00000457348	noncoding	1
	ENST00000458377	noncoding	3
	ENST00000458468	noncoding	6
	ENST00000497440	noncoding	3
	ENST00000500162	noncoding	4
	ENST00000500955	noncoding	1
	ENST00000518605	noncoding	1
	ENST00000524346	noncoding	2
	ENST00000524517	noncoding	1
	ENST00000553909	noncoding	1
	EU039832	noncoding	1
	Hs.130180	noncoding	2
	Hs.98895	noncoding	1
	M61870	noncoding	2
	NR_001284	noncoding	1
	NR_002788	noncoding	3
	NR_003366	noncoding	2
	NR_024420	noncoding	1

NR_024559	noncoding	1
NR_024602	noncoding	1
NR_026779	noncoding	1
NR_027270	noncoding	1
NR_027469	noncoding	1
NR_027791	noncoding	3
NR_027995	noncoding	1
NR_028080	noncoding	3
NR_033872	noncoding	2
NR_036581	noncoding	1
NR_036634	noncoding	1
NR_037918	noncoding	5
NR_038225	noncoding	1
NR_038226	noncoding	1
NR_038894	noncoding	1
NR_040091	noncoding	1
NR_040113	noncoding	1
NR_045012	noncoding	2
NR_045066	noncoding	3
NR_045563	noncoding	2
NR_047662	noncoding	4
NR_047680	noncoding	1
NR_047683	noncoding	1
OTTHUMT00000047661	noncoding	1
OTTHUMT00000157918	noncoding	3
OTTHUMT00000314912	noncoding	1
PIK3R1	coding	1
TCONS_00000692	noncoding	3
TCONS_00000879	noncoding	1
TCONS_00005921	noncoding	2
TCONS_00007856	noncoding	7
TCONS_00007857	noncoding	6
TCONS_00012852	noncoding	1
TCONS_00016964	noncoding	3
TCONS_00017606	noncoding	3
TCONS_00017615	noncoding	1
TCONS_00017649	noncoding	1
TCONS_00023208	noncoding	1
TCONS_00023287	noncoding	1
TCONS_00024652	noncoding	2
TCONS_00029196	noncoding	1
TCONS_l2_00001496	noncoding	2
TCONS_l2_00001497	noncoding	1
TCONS_l2_00001498	noncoding	1
TCONS_l2_00002156	noncoding	1
TCONS_l2_00002157	noncoding	1
TCONS_l2_00002159	noncoding	1

	TCONS_l2_00002161	noncoding	1
	TCONS_l2_00002491	noncoding	1
	TCONS_l2_00002636	noncoding	1
	TCONS_l2_00002637	noncoding	1
	TCONS_l2_00002638	noncoding	1
	TCONS_l2_00003113	noncoding	1
	TCONS_l2_00015025	noncoding	1
	TCONS_l2_00016247	noncoding	2
	TCONS_l2_00016249	noncoding	2
	TCONS_l2_00017143	noncoding	1
	TCONS_l2_00017424	noncoding	1
	TCONS_l2_00022091	noncoding	1
	TCONS_l2_00023048	noncoding	1
	TCONS_l2_00028814	noncoding	1
	TCONS_l2_00029366	noncoding	1
	TCONS_l2_00029368	noncoding	2
	TCONS_l2_00029376	noncoding	1
	TCONS_l2_00029378	noncoding	1
	U11872	noncoding	1
	U52701	noncoding	1
	uc001cqo.1	noncoding	1
	uc001ejc.2	noncoding	1
	uc001mmy.1	noncoding	1
	uc002aeh.2	noncoding	1
	uc002yji.2	noncoding	1
	uc002ykc.2	noncoding	3
	uc003ehd.2	noncoding	1
	uc010tcj.1	noncoding	1
hsa-miR-452-5p	AB019573	noncoding	1
	AF049885	noncoding	5
	AF429306	noncoding	1
	AK096049	noncoding	1
	AK124319	noncoding	1
	AK128778	noncoding	1
	DA714598	noncoding	2
	DQ645738	noncoding	1
	ENST00000372173	noncoding	1
	ENST00000400178	noncoding	20
	ENST00000411795	noncoding	2
	ENST00000419640	noncoding	1
	ENST00000419952	noncoding	10
	ENST00000428669	noncoding	9
	ENST00000437249	noncoding	1
	ENST00000445438	noncoding	1
	ENST00000445461	noncoding	13
	ENST00000450314	noncoding	2
	ENST00000453660	noncoding	1

ENST00000456327	noncoding	1
ENST00000456342	noncoding	20
ENST00000456587	noncoding	4
ENST00000457658	noncoding	1
ENST00000458377	noncoding	2
ENST00000458468	noncoding	20
ENST00000497440	noncoding	3
ENST00000500162	noncoding	1
ENST00000500955	noncoding	1
ENST00000504820	noncoding	3
ENST00000507072	noncoding	1
ENST00000518172	noncoding	1
ENST00000524346	noncoding	6
ENST00000524517	noncoding	2
ENST00000533992	noncoding	1
ENST00000553682	noncoding	1
ENST00000553909	noncoding	2
ENST00000555442	noncoding	1
ENST00000555864	noncoding	1
ENST00000557602	noncoding	1
ENST00000563477	noncoding	1
ENST00000565058	noncoding	1
ENST00000565979	noncoding	1
ENST00000566551	noncoding	1
ENST00000573312	noncoding	1
Hs.653095	noncoding	1
Hs.722974	noncoding	1
Hs.728856	noncoding	1
Hs.731140	noncoding	1
M76743	noncoding	1
M97723	noncoding	1
MEIS1	coding	1
NR_001543	noncoding	4
NR_002788	noncoding	3
NR_002925	noncoding	1
NR_003191	noncoding	1
NR_003366	noncoding	8
NR_003662	noncoding	2
NR_015446	noncoding	3
NR_024420	noncoding	2
NR_024559	noncoding	2
NR_026558	noncoding	1
NR_027469	noncoding	1
NR_027791	noncoding	13
NR_027906	noncoding	1
NR_028080	noncoding	24
NR_033234	noncoding	4

NR_033701	noncoding	1
NR_033798	noncoding	1
NR_033815	noncoding	1
NR_033828	noncoding	1
NR_034112	noncoding	1
NR_036489	noncoding	1
NR_036522	noncoding	1
NR_036627	noncoding	2
NR_036634	noncoding	7
NR_037918	noncoding	11
NR_038225	noncoding	3
NR_038226	noncoding	3
NR_038846	noncoding	1
NR_038877	noncoding	1
NR_040013	noncoding	1
NR_040091	noncoding	2
NR_040113	noncoding	1
NR_045066	noncoding	8
NR_045211	noncoding	1
NR_045405	noncoding	4
NR_045563	noncoding	9
NR_046422	noncoding	1
NR_047662	noncoding	1
NR_047680	noncoding	4
NR_047683	noncoding	4
NR_072979	noncoding	4
OTTHUMT0000032602[illegible]	noncoding	1
TCONS_00000692	noncoding	2
TCONS_00002831	noncoding	1
TCONS_00005921	noncoding	1
TCONS_00007624	noncoding	1
TCONS_00007856	noncoding	6
TCONS_00007857	noncoding	5
TCONS_00009804	noncoding	2
TCONS_00011145	noncoding	1
TCONS_00016354	noncoding	1
TCONS_00016964	noncoding	6
TCONS_00017000	noncoding	1
TCONS_00017606	noncoding	4
TCONS_00017615	noncoding	1
TCONS_00019178	noncoding	1
TCONS_00020005	noncoding	1
TCONS_00022863	noncoding	2
TCONS_00022864	noncoding	2
TCONS_00023208	noncoding	1
TCONS_00023441	noncoding	1
TCONS_00024652	noncoding	3

	TCONS_00029196	noncoding	3
	TCONS_l2_00002491	noncoding	2
	TCONS_l2_00003113	noncoding	3
	TCONS_l2_00009425	noncoding	1
	TCONS_l2_00011031	noncoding	1
	TCONS_l2_00015025	noncoding	2
	TCONS_l2_00017137	noncoding	1
	TCONS_l2_00022091	noncoding	2
	TCONS_l2_00022661	noncoding	2
	TCONS_l2_00022666	noncoding	1
	TCONS_l2_00028814	noncoding	7
	TCONS_l2_00029293	noncoding	1
	TCONS_l2_00029376	noncoding	1
	TCONS_l2_00029508	noncoding	1
	TCONS_l2_00029722	noncoding	1
	TCONS_l2_00029723	noncoding	1
	TCONS_l2_00029938	noncoding	1
	U52699	noncoding	2
	U52701	noncoding	2
	uc001cqo.1	noncoding	2
	uc001hgr.2	noncoding	1
	uc001mmy.1	noncoding	2
	uc001zgs.1	noncoding	1
	uc002aeh.2	noncoding	1
	uc002qng.2	noncoding	1
	uc002ykc.2	noncoding	13
	uc004acq.3	noncoding	1
	uc010rpo.1	noncoding	1
hsa-miR-501-5p	AF049885	noncoding	5
	AF429306	noncoding	1
	AK001941	noncoding	1
	AK026822	noncoding	1
	AK056173	noncoding	1
	AK096049	noncoding	2
	BC033124	noncoding	1
	CR599621	noncoding	1
	CR624679	noncoding	1
	DQ925687	noncoding	1
	ENST00000400178	noncoding	15
	ENST00000415330	noncoding	1
	ENST00000416395	noncoding	1
	ENST00000419952	noncoding	10
	ENST00000427168	noncoding	1
	ENST00000427491	noncoding	2
	ENST00000428669	noncoding	9
	ENST00000435892	noncoding	1
	ENST00000437249	noncoding	2

ENST00000439302	noncoding	3
ENST00000445461	noncoding	14
ENST00000453660	noncoding	1
ENST00000456280	noncoding	1
ENST00000456327	noncoding	3
ENST00000456342	noncoding	15
ENST00000456587	noncoding	5
ENST00000458377	noncoding	4
ENST00000458468	noncoding	15
ENST00000490162	noncoding	2
ENST00000497440	noncoding	9
ENST00000500162	noncoding	4
ENST00000500955	noncoding	2
ENST00000504820	noncoding	4
ENST00000507508	noncoding	2
ENST00000514420	noncoding	1
ENST00000515455	noncoding	1
ENST00000518172	noncoding	1
ENST00000518902	noncoding	2
ENST00000524346	noncoding	13
ENST00000524517	noncoding	1
ENST00000526206	noncoding	2
ENST00000539229	noncoding	1
ENST00000553682	noncoding	3
ENST00000555864	noncoding	1
ENST00000561507	noncoding	1
ENST00000563931	noncoding	1
ENST00000565979	noncoding	1
ENST00000570183	noncoding	1
ENST00000573260	noncoding	1
ENST00000573312	noncoding	1
EU039832	noncoding	1
Hs.569669	noncoding	1
Hs.570567	noncoding	2
Hs.629644	noncoding	1
Hs.653095	noncoding	2
Hs.722974	noncoding	4
Hs.728856	noncoding	1
LPIN1	coding	1
M97723	noncoding	2
NR_001284	noncoding	1
NR_001543	noncoding	3
NR_001544	noncoding	1
NR_002788	noncoding	2
NR_003191	noncoding	2
NR_003366	noncoding	1
NR_003662	noncoding	1

NR_015446	noncoding	3
NR_023388	noncoding	1
NR_024420	noncoding	2
NR_024559	noncoding	5
NR_024602	noncoding	1
NR_026558	noncoding	1
NR_026578	noncoding	1
NR_026700	noncoding	1
NR_027399	noncoding	1
NR_027469	noncoding	1
NR_027647	noncoding	1
NR_027663	noncoding	2
NR_027791	noncoding	14
NR_028049	noncoding	1
NR_028080	noncoding	16
NR_028343	noncoding	1
NR_033234	noncoding	9
NR_033798	noncoding	1
NR_033872	noncoding	4
NR_036489	noncoding	2
NR_036627	noncoding	1
NR_036634	noncoding	8
NR_037918	noncoding	11
NR_038225	noncoding	1
NR_038226	noncoding	1
NR_038433	noncoding	2
NR_038846	noncoding	1
NR_038894	noncoding	2
NR_038926	noncoding	1
NR_040091	noncoding	3
NR_045012	noncoding	1
NR_045066	noncoding	15
NR_045128	noncoding	1
NR_045129	noncoding	1
NR_045211	noncoding	2
NR_045405	noncoding	3
NR_045563	noncoding	20
NR_046422	noncoding	2
NR_047662	noncoding	4
NR_047680	noncoding	5
NR_047683	noncoding	5
NR_072979	noncoding	2
OTTHUMT0000015791[illegible]	noncoding	3
OTTHUMT0000031491[illegible]	noncoding	1
TCONS_00000692	noncoding	7
TCONS_00002831	noncoding	1
TCONS_00003049	noncoding	1

	TCONS_00003850	noncoding	1
	TCONS_00004435	noncoding	1
	TCONS_00005790	noncoding	2
	TCONS_00005921	noncoding	1
	TCONS_00007856	noncoding	11
	TCONS_00007857	noncoding	5
	TCONS_00009804	noncoding	3
	TCONS_00012852	noncoding	1
	TCONS_00014234	noncoding	1
	TCONS_00016964	noncoding	6
	TCONS_00017590	noncoding	1
	TCONS_00017606	noncoding	2
	TCONS_00017615	noncoding	2
	TCONS_00017649	noncoding	2
	TCONS_00021470	noncoding	1
	TCONS_00021496	noncoding	1
	TCONS_00021543	noncoding	1
	TCONS_00022863	noncoding	1
	TCONS_00022864	noncoding	1
	TCONS_00029196	noncoding	2
	TCONS_l2_00001496	noncoding	3
	TCONS_l2_00001497	noncoding	3
	TCONS_l2_00001498	noncoding	3
	TCONS_l2_00002491	noncoding	2
	TCONS_l2_00003113	noncoding	1
	TCONS_l2_00011031	noncoding	1
	TCONS_l2_00013919	noncoding	2
	TCONS_l2_00015025	noncoding	2
	TCONS_l2_00016247	noncoding	1
	TCONS_l2_00016249	noncoding	1
	TCONS_l2_00021898	noncoding	1
	TCONS_l2_00029290	noncoding	1
	TCONS_l2_00029291	noncoding	1
	TCONS_l2_00029292	noncoding	1
	TCONS_l2_00029368	noncoding	1
	TCONS_l2_00029376	noncoding	1
	TCONS_l2_00029378	noncoding	1
	uc001cqo.1	noncoding	2
	uc001hgr.2	noncoding	1
	uc002aeh.2	noncoding	1
	uc002ykc.2	noncoding	14
	uc003ehd.2	noncoding	5
	uc003ttk.1	noncoding	2
	uc004acq.3	noncoding	1
	uc010rpo.1	noncoding	6
