# Supplementary figures and images for: Integrated Analysis of Long Noncoding RNA and mRNA Expression Profile in Advanced Laryngeal Squamous Cell Carcinoma
Source: PLoS One. 2016 Dec 29;11(12):e0169232. doi: 10.1371/journal.pone.0169232 (PMC5199101; doi:10.1371/journal.pone.0169232)

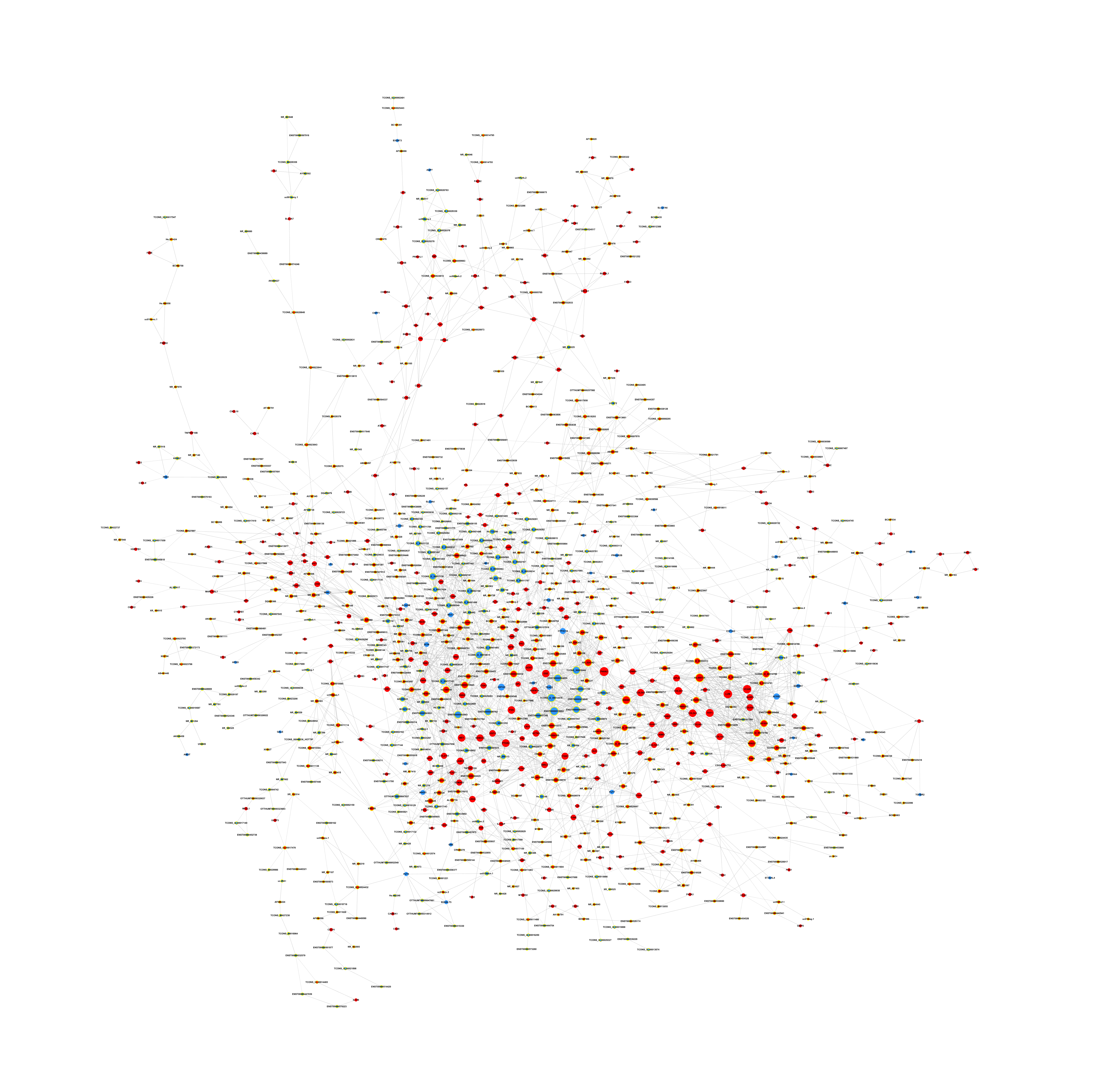

Supplement: S2 Fig — (PNG) [file pone.0169232.s007.png]

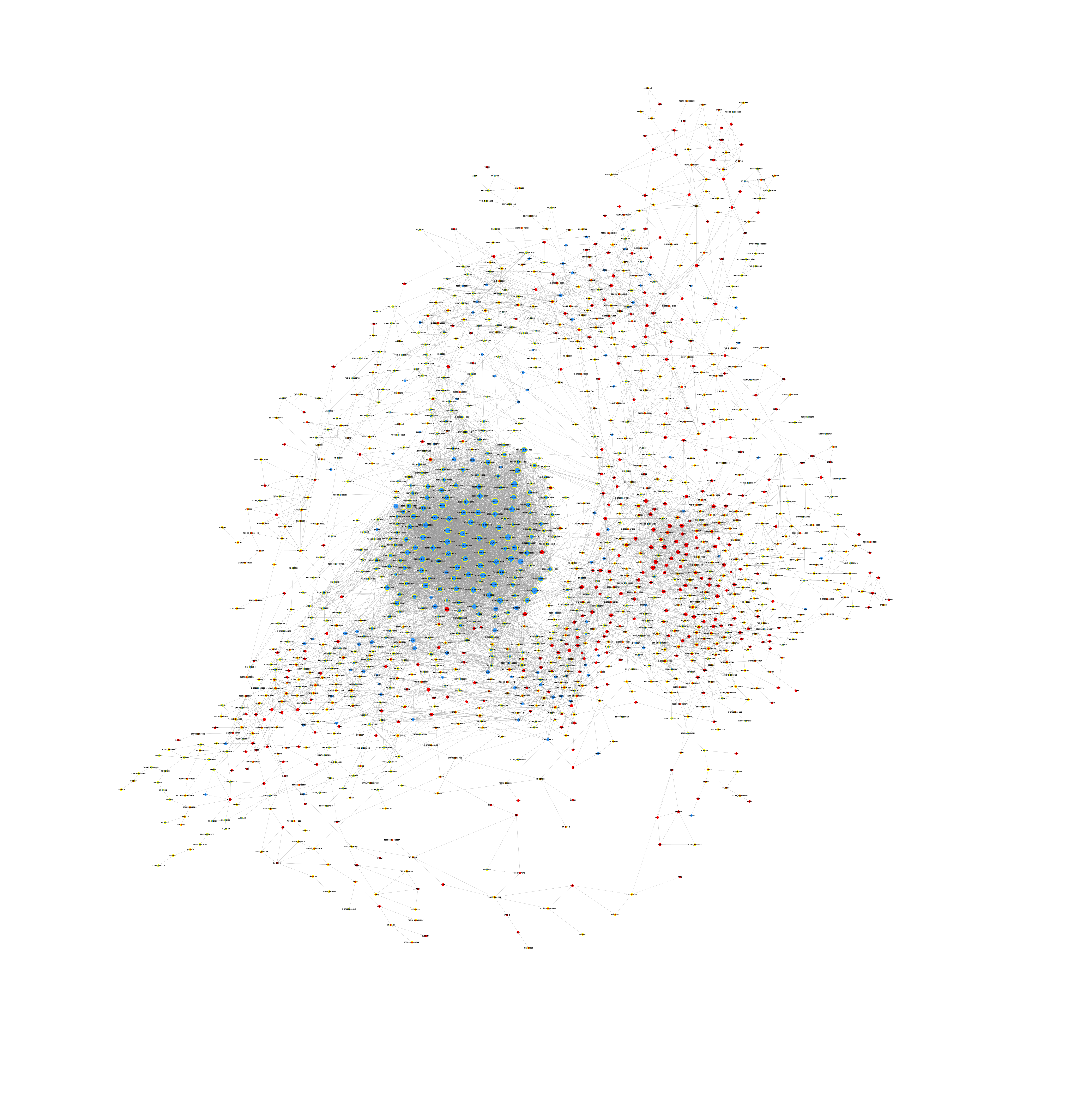

Supplement: S3 Fig — (PNG) [file pone.0169232.s008.png]
