# Supplementary material for: Integrated Analysis of Long Noncoding RNA and mRNA Expression Profile in Advanced Laryngeal Squamous Cell Carcinoma
Source: PLoS One. 2016 Dec 29;11(12):e0169232. doi: 10.1371/journal.pone.0169232 (PMC5199101; doi:10.1371/journal.pone.0169232)
Supplement: S1 Fig — In the network, a node represents a gene, red color indicates up-regulation and blue color indicates down-regulation, the size of the node’s area represents the value of betweenness centrality. The nodes connect by an edge. The indicators a, b, c, p, u, m, inh, ex, dep, ind are abbreviation of activation, binding, compound, phosphorylation, ubiquitination, missing interaction, inhibition, expression, dephosphorylation, indirect effect respectively. (PDF) [file pone.0169232.s006.pdf]

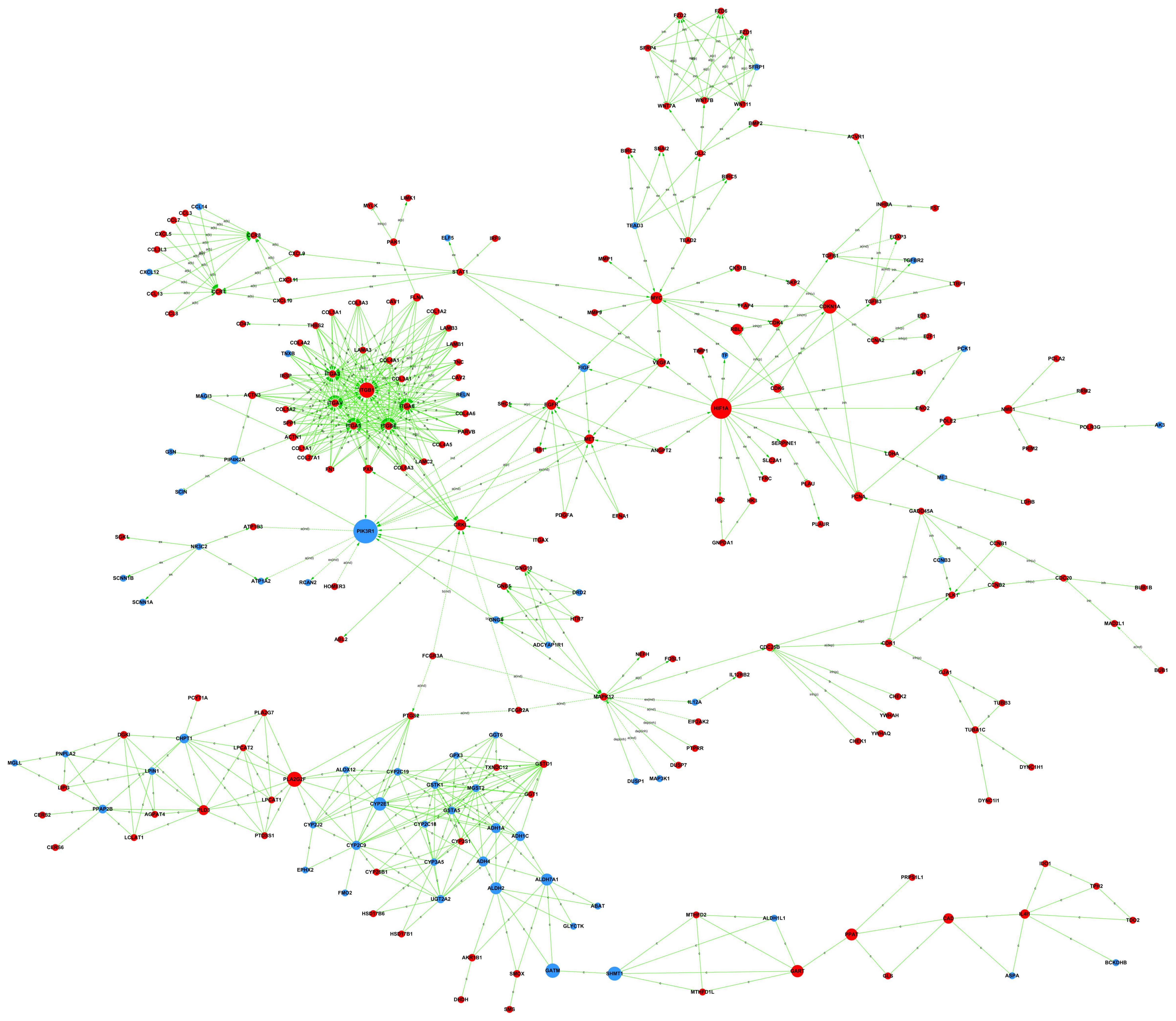
PIK3R1
HIF1A
CDKN1A
GADD45A
MAPK12
SHMT1
GATM
ALDH7A1
ALDH2
ALDH1L1
CYP2C19
CYP2C9
CYP2C18
CYP2J2
CYP3A5
GSTA5
GSTK1
MGST2
ADH1A
ADH1C
ADH4
EPHX2
FMO2
TXNDC12
GPX3
GGT5
ALOX12
PNPLA2
MGLL
LPIN1
PPAP2B
AGPAT4
LCLAT1
CHPT1
TEAD3
TGFBR2
NR3C2
SCNN1B
SCNN1A
PIP4K2A
ADCYAP1R1
ARAT
GLYCTK
BCKDHB
ABAT
